# Electrochemical Vicinal Difluorination of Alkenes: Scalable and Amenable to Electron‐Rich Substrates†

**DOI:** 10.1002/anie.201912119

**Published:** 2019-11-28

**Authors:** Sayad Doobary, Alexi T. Sedikides, Henry P. Caldora, Darren L. Poole, Alastair J. J. Lennox

**Affiliations:** ^1^ School of Chemistry University of Bristol Cantock's Close Bristol BS8 1TS UK; ^2^ Medicines Design GSK Medicines Research Centre Gunnels Wood Rd Stevenage SG1 2NY UK

**Keywords:** electrochemistry, fluorination, green chemistry, hypervalent iodine, oxidation

## Abstract

Fluorinated alkyl groups are important motifs in bioactive compounds, positively influencing pharmacokinetics, potency and conformation. The oxidative difluorination of alkenes represents an important strategy for their preparation, yet current methods are limited in their alkene‐types and tolerance of electron‐rich, readily oxidized functionalities, as well as in their safety and scalability. Herein, we report a method for the difluorination of a number of unactivated alkene‐types that is tolerant of electron‐rich functionality, giving products that are otherwise unattainable. Key to success is the electrochemical generation of a hypervalent iodine mediator using an “ex‐cell” approach, which avoids oxidative substrate decomposition. The more sustainable conditions give good to excellent yields in up to decagram scales.

## Graphical abstract

**Fluorination for the nation**: A method for the difluorination of unactivated alkenes with electron‐rich functionalities is described. Key to success is the electrochemical generation of a hypervalent iodine mediator using an “ex‐cell” approach, which avoids oxidative substrate decomposition.

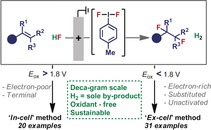

The inclusion of fluorine in bioactive compounds is becoming more important:^1^ since 2006, prevalence has increased from 6 % to 31 % in the top 100 best‐selling small‐molecule drugs.^2^ Fluorinating alkyl groups can increase potency, lipophilicity and metabolic stability,^3^ while reducing the basicity of neighbouring amines,^4^ all of which can improve bioactivity and pharmacokinetics. The vicinal difluoroethane unit has attracted recent attention, particularly as a bioisostere of ethyl or trifluoromethyl groups^5^ (Figure 1 A) and for its unique propensity to adopt a gauche conformation in solution.^6^ Exploiting this stereo‐electronic effect is an emerging strategy for molecular design,^7^ and has found application in, for example, organocatalysis^8^ and peptide mimics.^9^

**Figure 1:**
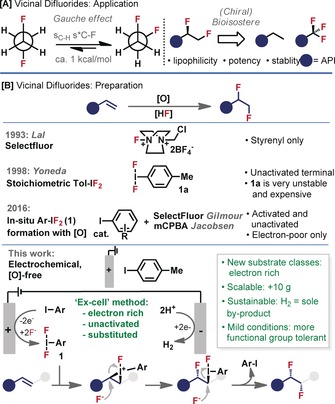
A) Significance and application of vicinal difluorides. B) Methods to prepare vicinal difluorides.

Simple vicinal difluorides have been prepared from alkenes with the use of ambiphilic fluoride reagents, F_2_ and XeF_2_.^10^ However, their high reactivity, toxicity and high cost render their use impractical. While the required oxidizing equivalents are self‐contained in these reagents, subsequent methods have relied on the combination of HF‐salts and oxidants (Figure 1 B). Oxidation with the use of electrochemistry^11^ or Selectfluor^12^ provided the earliest confirmations of this strategy. However, in both cases the product selectivity is poor and the alkene‐types amenable to the reaction are limited, with success demonstrated on only very simple substrates. Yoneda employed *p*‐tolyl difluoro λ^3^‐iodane (**1 a**) as the oxidant,^13^, ^14^ which improved product selectivity, however **1 a** is light‐ and temperature‐sensitive, highly hygroscopic and expensive.^15^ Thus, the in situ formation of **1 a** using aryl‐iodide, HF‐salts and Selectfluor or mCPBA, was the subject of elegant work by both Gilmour^16^ and Jacobsen.^17^ The use of these stoichiometric oxidants then permits the use of sub‐stoichiometric quantities of the aryl‐iodide catalyst.

While these examples represent great advances in accessing vicinal difluorides, there is no general method for accessing these motifs in compounds containing electron‐rich moieties; due to competitive substrate oxidation, resulting in either decomposition or unselective fluorination. Moreover, owing to the potential of fluorinated alkyl groups in high‐value bioactive compounds, a method that is readily scaled, and therefore is safe, inexpensive and does not produce much waste, is still required.

To address these shortfalls, we sought to access the λ^3^‐iodane **1** mediators^18^ using electrochemical^19^ oxidation. The unique spatial control of redox events, along with the control of potential and rate, should facilitate the expansion of substrate classes. As well as the inherent safety and scalability of electrochemistry,^20^ the addition of a chemical oxidant is not required, as protons can ultimately accept electrons at the cathode to form H_2_ as the sole by‐product, thereby rendering the process more atom‐economical.^21^

The electrochemical generation of many hypervalent λ^3^‐iodane species from aryl‐iodides is known.^22^ Difluoro λ^3^‐iodanes (**1**) has been comparatively less explored,^23^ with Waldvogel recently reporting the only example in the presence of alkenes,23e which are liable to preferentially oxidise. A number of additional problems are reported to occur when **1** is generated at an electrode,^24^ which include dimerization, benzylic fluorination and the formation of “many other complicated products”.23d The application of high potentials with HF‐salts also causes anode passivation, where a non‐conducting polymer coating forms on the electrode surface that suppresses faradaic current and can attenuate reaction.^25^ Thus, our primary objective was to address these issues in our optimisations.

We started by examining different aryl‐iodide mediators for the electrochemical difluorination of allylbenzene (**2 a**) (Figure 2 A). No conversion to difluorinated alkane **3 a** was observed with the use of 4‐iodo‐anisole (*R*=4‐OMe), the most readily oxidized derivative we tested. However, we observed the formation of **3 a** using aryl‐iodides with higher oxidation potentials. Further increases in potential beyond R=Me (tolyl) led to a subsequent decline in yield, as direct substrate oxidation out‐competes aryl‐iodide oxidation (ca. *E*_ox_=1.9 V). Although iodotoluene gave the highest yield of **3 a**, substantial quantities of benzylic fluorination (to **4**) were observed (<18 %) (Figure 2 B). We confirmed that **4** itself is a very poor mediator, as only 7 % of **3 a** was returned when using **4** in place of **1 a**.^26^ As this side reaction requires deprotonation, we reasoned that it should be attenuated by reducing the availability of basic fluoride by increasing the proportion of HF to amine (in mixes of commercially available 3 HF⋅NEt_3_ and 9HF⋅py (amine=py or NEt_3_). Indeed, by increasing this ratio from 3 to 5.6, benzylic fluorination decreased with an accompanying increase in product **3 a** (Figure 2 B). This trend also mirrors the enhanced activation of **1 a** with acid expected from the presence of more HF. Further increases beyond 5.6 maintained the lack of **4** formation, but led to a decline in product **3 a**, possibly reflecting a decrease in fluoride activity. This sweet‐spot demonstrates the delicate balance between activation of **1 a** and deactivation of fluoride.

**Figure 2:**
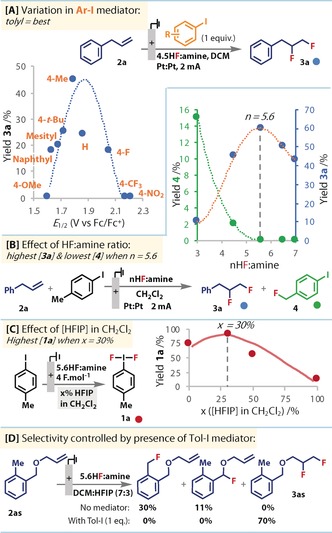
Reaction optimisation: electrolyses in undivided cell, ^19^F NMR yields. A) Yields of **3 a** from **2 a** (25 mm) in the presence of different aryl‐iodides (**1**) 4 F mol^−1^. *E*_1/2_ vs. Fc/Fc^+^. B) Yields of **4** (blue) and **3 a** (green) from **1 a** and **2 a** (both 25 mm) in different HF:amine ratios after 4 F mol^−1^. “Amine”=NEt_3_ or py. C) Yields of **1 a** after 4 F mol^−1^, 12 mA (Pt∥Pt) in different amounts of HFIP in CH_2_Cl_2_. D) Fluorination of **2 as** with and without Tol‐I. 4 F mol^−1^ passed at 12 mA (Pt∥Pt).

The influence of solvent was then examined. MeCN performed worse than CH_2_Cl_2_, which was unsurprising considering anode passivation is particularly predominant under these conditions.^25^ This insulating effect was reflected by a large rise in cell potential during reaction compared to other solvents tested,^26^ none of which improved the yield beyond that of CH_2_Cl_2_. However, when hexafluoro‐isopropanol (HFIP) was added as a co‐solvent, higher yields of **3 a** were observed, as also noted in other halogenation^27^ and electrochemical reactions.^28^ 30 % HFIP in CH_2_Cl_2_ led to the greatest enhancement.^26^ Control reactions in the absence of alkene revealed that the concentration of HFIP determined the amount of **1 a** formation (Figure 2 C) after the same amount of charge was passed. The addition of allylpentafluorobenzene (**2 e**) to these mixtures confirmed its more efficient transformation to **3 e** in the presence of more **1 a**.^26^ To rationalise the downward trend of **1 a** (Figure 2 C), we observed a reduced solubility of iodotoluene with increased HFIP. CV studies revealed that the oxidation potential of iodotoluene under the reaction conditions decreases with more HFIP present,^26^, ^29^ resulting in a milder oxidizing environment, which should contribute to improved functional group tolerance. Finally, ^19^F NMR analysis of a genuine sample of **1 a**, supported our assumption that **1 a** was formed under the optimized conditions.^26^

The importance of the iodotoluene mediator to product selectivity was confirmed by control reactions in its absence (Figure 2 D). Without iodotoluene, direct oxidation of **2 as** led to benzylic, rather than alkene, fluorination with low mass balance. The use of sub‐stoichiometric quantities of the mediator led to a decline in yield of **3 a**,^26^ reflecting a mismatch of reaction‐rates in the electrochemical and chemical steps (EC mechanism). We could drop the loading of iodotoluene to 20 mol % by applying 5 cycles of 0.7 F mol^−1^ with 6 h stirring in between each cycle.^26^ However, the vastly increased reaction time was deemed to be an inferior adjustment to the conditions than using an equivalent of iodotoluene and running the reaction in one go. Moreover, we are able to recover pure iodotoluene in greater than 70 % yields, and thus recycle it for use in subsequent reactions.

With this “in‐cell” optimised method now in hand, we proceeded to explore the substrate scope (Figure 3). Previously published protocols do not report substrate classes that contain electron‐rich moieties, therefore, we initially avoided these substrates. Indeed, electron‐poor allyl arenes were well tolerated (**2 a**–**e**) and gave difluorinated products in good to excellent yields. Long‐chain alkenes, **2 f**–**h**, returned excellent yields, demonstrating that a proximal aromatic ring is not necessary for reactivity. Allylic ethers and amines (**2 i**–**t**) were both tolerated if containing electron poor (hetero)aromatics. Ester (**2 h**), alcohol (**2 f**), sulfonate (**2 g**) and halide (Br, Cl, e.g., **2 l** and **2 q**) functional groups were all untouched, providing useful products for further derivatization. While acid sensitive functionality, such as boc groups, was not tolerated,^26^ a cyclopropyl ring (**2 t**) avoided competing oxidative ring opening.^30^

**Figure 3:**
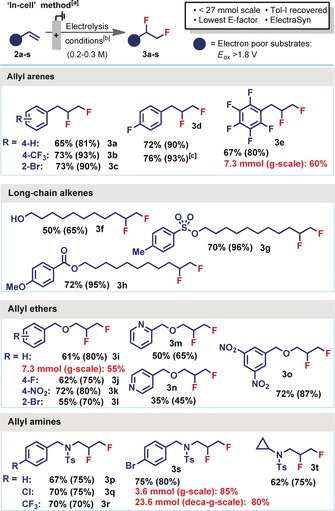
[a] Isolated yields (NMR yields in parentheses). [b] **2** (1.2 mmol (0.3 m) or 0.8 mmol (0.2 m)) and iodotoluene (1 equiv.) in 3 parts 5.6 HF:amine (where amine=py or NEt_3_) and 1 part CH_2_Cl_2_:HFIP (7:3); electrolysis: 8 mA (0.2 m) or 12 mA (0.3 m), 3.5 F mol^−1^, Pt∥Pt, undivided cell, then stirring (12 h). [c] Reaction performed with Electrasyn 2.0 in a PTFE vial.

The scalability of the method was demonstrated by yielding gram‐scale quantities of products **3 e**, **3 i** and **3 s** and a deca‐gram‐scale quantity of **3 s**. In each of these cases, 70+% of pure iodotoluene was recovered. The commercially available ElectraSyn 2.0 set‐up was also tested‐in combination with a PTFE vial‐and was found to give product **3 d** in a comparable yield to our set‐up, validating the robustness of the conditions.

These “in‐cell” conditions performed worse with the more electron‐rich allyltoluene (**2 u**) (Figure 4), returning only a moderate yield of difluorinated product **3 u**. This reactivity is consistent with previous methods that also struggle with readily oxidized substrate classes.^16^ Therefore, a new approach was sought to specifically gain access to products containing electron‐rich moieties. Analysis of a range of electron‐rich substrates by cyclic voltammetry revealed their preferential oxidation to iodotoluene,^26^ thereby eluding the vital formation of λ^3^‐iodane **1 a**. To avoid this problem, an “ex‐cell” method was devised that spatially and temporally separates the electrochemical oxidation and fluorination steps, thereby avoiding competitive direct oxidation of the substrates. Thus, conditions were re‐optimized for the initial formation of **1 a** in a divided cell, followed by the subsequent addition of substrate **2 u**.^26^ With this approach the yield of **3 u** was raised from 45 % to 73 % (Figure 4).

**Figure 4:**
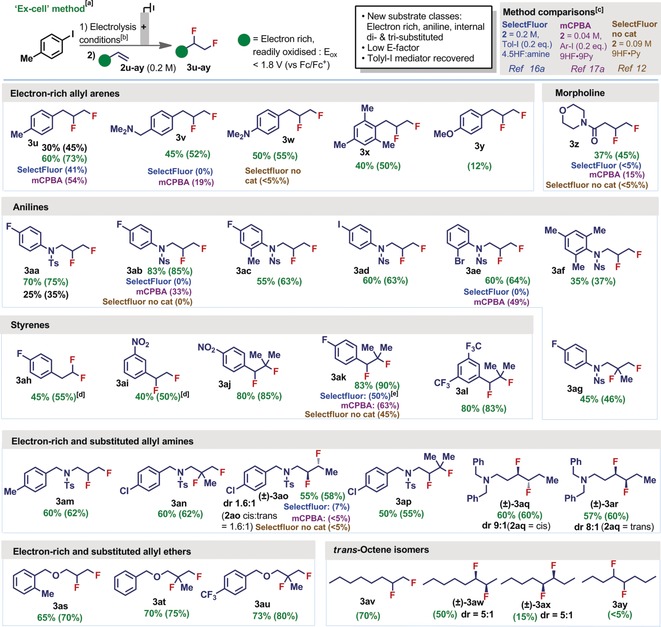
[a] Isolated yields with NMR yields in parentheses, black=“in‐cell”, green=“ex‐cell”. [b] Electrolysis of iodotoluene (1.2 mmol, 0.2 m) in 3 parts 5.6 HF:amine and 1 part CH_2_Cl_2_:HFIP (7:3): 12 mA, 3.0 F mol^−1^, Pt∥Pt, divided cell, then addition of 2 (1.2 mmol), stir (12 h). [c] Selectfluor + Tol‐I conditions from Ref. 16a; mCPBA + Ar‐I conditions from Ref. 17a; Selectfluor conditions from Ref. 12. [d] CHCl_3_ used instead of CH_2_Cl_2_ with 4.5HF:amine. [e] Styrene difluorination conditions from Ref. 16b. Selectfluor conditions from Ref. 16a generated only (17 %) **3 ap**.

The scope of electron‐rich or easily oxidised substrates was now tested with this “ex‐cell” method (Figure 4). Electron‐rich allyl arenes (**2 u**–**x**) were now tolerated, returning moderate to very good yields of product. Success was achieved with the very electron‐rich dimethylaniline **2 w**, however, the anisole derivative **2 y** was less well tolerated, which may be due to the known C−H activation pathway of these arenes‐types to generate diaryliodonium species.^31^ A pharmaceutically relevant morpholine amide (**2 z**) was also tolerated.

Anilines are a substrate class that have also not previously been demonstrated, as they are very readily oxidized. Aniline **2 aa** posed problems using the “in‐cell” method, cf. only 25 % **3 aa** was isolated. However, by adopting the “ex‐cell” method, this was increased to 70 %. The greater electron withdrawing effect of nosyl (**3 ab**) vs. tosyl (**3 aa**) translated into greater yields. Good to excellent yields of other difluorinated anilines containing electron rich and poor rings were generated (**3 ac**–**ag**), including mesityl. Other terminal alkenes containing electron‐rich moieties were tolerated, including an allyl amine (**2 am**) and ether (**2 as**). Styrenes were tolerated if they were electron poor, such as **2 ai**, otherwise geminal fluorination occurred (**2 ah**), which is a process that has previously been observed with this substrate class.^32^ Substituted styrenes (**2 aj**–**al**) were also well tolerated.

Substituted alkenes that are unactivated are problematic substrates for other methods, and so we were pleased to discover that our “ex‐cell” conditions readily translate electron‐rich di‐ and tri‐substituted terminal and internal alkenes (**2 ag**, **2 an**–**ar, 2 at**–**au**). Good to excellent yields were produced in each case. Both *cis* and *trans* isomers of internal alkenes were viable substrates, and their stereochemical information was translated to their products with a diastereoselectivity between 5–9:1. Readily oxidised trialkylamines (**2 aq**–**ar**) were again tolerated functionality. To ascertain the effect of steric bulk or hydrophobic shielding on internal alkenes, 4 regio‐isomers of *trans*‐octene were tested in the reaction (**2 av**–**ay**). It was found that yields improved as the bulk either side of the alkene decreased.

The difluorination of several electron‐rich substrates using our electrochemical method was compared to methods in the literature, including those that employ Selectfluor and mCPBA with aryl‐iodide, and Selectfluor alone. In each of the examples tested, the electrochemical method gave superior yields (Figure 4), thereby validating the importance of the “ex‐cell” approach. The sustainability of the electrochemical method is reflected in the low E‐factor^33^ (ratio of total waste to product) calculated for the reaction,^34^ which was consistently lower than the Selectfluor or mCPBA methods. The main improvement to waste reduction originates from the lack of a stoichiometric oxidant, which will also contribute to enhanced safety^35^ and lower cost^26^ on‐scale.

In summary, an electrochemical vicinal 1,2‐difluorination of alkenes has been described, using a simple and user‐friendly 2‐electrode setup with nucleophilic fluoride and iodotoluene as a mediator. Moderate to excellent yields of fluorinated products are demonstrated in a wide substrate scope. The “ex‐cell” method allows access to new substrate classes that have otherwise remained unattainable, including electron rich moieties, anilines and substituted internal alkenes. The method is sustainable (lower E‐factor), safe, and high‐yielding gram and decagram scale reactions demonstrate the practicality of the process. We therefore expect this method to facilitate access to this important motif in a wider variety of compounds and contexts.

## Conflict of interest

The authors declare no conflict of interest.

## Supporting information

As a service to our authors and readers, this journal provides supporting information supplied by the authors. Such materials are peer reviewed and may be re‐organized for online delivery, but are not copy‐edited or typeset. Technical support issues arising from supporting information (other than missing files) should be addressed to the authors.

Supplementary
