# Supplementary material for: Electrochemical Vicinal Difluorination of Alkenes: Scalable and Amenable to Electron‐Rich Substrates
Source: Angew Chem Int Ed Engl. 2019 Nov 28;59(3):1155–60. doi: 10.1002/anie.201912119 (PMC6973232; doi:10.1002/anie.201912119)
Supplement: Supplementary file 1 — Supplementary [file ANIE-59-1155-s001.pdf]

Angewandte Chemie
Eine Zeitschrift der Gesellschaft Deutscher Chemiker

Supporting Information

Electrochemical Vicinal Difluorination of Alkenes: Scalable and Amenable to Electron-rich Substrates**

*Sayad Doobary, Alexi T. Sedikides, Henry P. Caldora, Darren L. Poole, and Alastair J. J. Lennox**

anie_201912119_sm_miscellaneous_information.pdf

Contents

General Experimental details	**S3**
Optimisation studies	**S6**
Formation of p-tolyl difluoro λ3-iodane, **1a**	**S18**
Stability of p-tolyl difluoro λ3-iodane, **1a**, under reaction conditions	**S19**
Investigation into the role of HFIP	**S20**
Control reactions	**S22**
General synthetic procedures of substrates **2**	**S24**
Characterisation of substrates	**S28**
Synthesis of iodoarene catalysts for benchmarking experiments	**S53**
Electrochemical setup	**S54**
General procedures for electrochemical 1,2 difluorination of alkenes **2** to **3**	**S59**
Characterisation of products **3**	**S63**
Faradaic efficiency calculations	**S113**
Benchmarking experiments	**S114**
E-factor analysis	**S117**
Price of oxidant calculations	**S121**
Unsuccessful substrates	**S124**
NMR spectra of substrates	**S125**
NMR spectra of products	**S144**
References	**S218**

General experimental details:

Techniques

Manipulations involving air and moisture sensitive materials were conducted employing standard Schlenk-line and glovebox techniques, using vacuum lines attached to a double manifold with greaseless J. Youngs valves equipped with an oil pump (0.1mmHg) under an atmosphere of dry nitrogen. All glassware was dried overnight before use, in a 180º oven and then allowed to cool under vacuum at 0.1 mmHg. The removal of solvents in vacuo was achieved using a Büchi rotary evaporator (bath temperatures up to 40 °C) at a pressure of 15 mmHg (diaphragm pump), or at 0.1 mmHg (oil pump) on a vacuum line at room temperature. The addition of liquids, with the exception of HF solutions, were added using a Gilson PIPETMAN p20.

Solvents

THF (tetrahydrofuran), CH_2Cl_2, CH_3CN and Et_2O was dried using an Anhydrous Engineering alumina column drying system situated in the University of Bristol’s chemistry department. All solvents were collected using Strauss flasks using a gas-tight J. Youngs valve. Anhydrous 1,1,1,2,2,2 hexafluoroisopropanol (HFIP) was collected by distillation after stirring in $MgSO_4$ overnight. Deuterated solvents for NMR analysis were purchased from *Sigma Aldrich*.

Chromatography

TLC analysis was performed on Merck Silica gel 60F_{254} glass backed plates. Visualisation was achieved by UV fluorescence (254 nm). Flash column chromatography was conducted using Fluorochem 60 silica: 230-400 mesh (40-63 µm).

Reagents

Allylbenzene, 1-allyl-4-trifluoromethylbenzene, 4-Allylanisole, Allylpentafluorobenzene, 4-allyltoluene, 1-Allyl-2-Bromo benzene, Allyl benzyl ether, 1-allyl-4-fluorobenzene, 3-nitrostyrene, 3,5-bis(trifluoromethyl)benzaldehyde, 4-nitrobenzaldehyde, 4-fluorobenzaldehyde, 2-methylbenzylalcohol, 2-octene, crotyl alcohol, prenyl alcohol, 10-undencen-1-ol, 3,5-dinitrobenzyl alcohol, 2-bromobenzyl

alcohol, 2-pyridinemethanol, 4-pyridinemethanol, 3-butenoic acid, N-(3-dimethylaminopropyl)-N'-ethylcarbodiimide hydrochloride, magnesium, sodium hydride in mineral oil, cyclopropylamine, triphenylphosphine were all purchased from Sigma Aldrich. Octene, 4-chlorobenzylamine, 4-trifluomethyl benzyl alcohol, 4-fluorobenzyl alcohol, 4-iodobenzyl alcohol, 4-bromobenzyl alcohol, 4-trifluoromethylbenzyl amine, 4-Bromo-N,N-dimethylaniline, 4-fluoro aniline, 2-bromo aniline, 4-nitrobenzenesulfonyl chloride, diisopropyl azodicarboxylate were all purchased from Fluorochem. Morpholine, 2,4,6-trimethyl aniline, 4-Fluoro-2-methyl aniline, 4-iodo aniline, trans-3-octene, trans-4-octene, 1-chloro-2-methylpropene, allylbromide, 4-methoxybenzoyl chloride, 4-Bromo-N,N-dimethylaniline, triethylamine, pyridine, potassium carbonate, 4-(dimethylamino)pyridine were all purchased from Alfa-Aesar. Bromomesitylene, 4-toluenesulfonyl chloride, isopropyltriphenylphosphonium iodide were all purchased from Acros. Benzyl alcohol was purchased from Honeywell.

Analysis

NMR spectra were recorded on Bruker Nano 400 or Bruker Advance III HD 500 cryo spectrometers. Chemical shifts (δ) are quoted in parts per million (ppm), referenced to the residual solvent peak (^{1}H and ^{13}C NMR) and coupling constants (J) are given in Hz. Multiplicities are abbreviated as: s (singlet), d (doublet), t (triplet), q (quartet), m (multiplet) or combinations thereof. NMR shifts for novel compounds have been assigned with the use of the appropriate 2D NMR experiments, such as COSY, HSQC and HMBC. Infrared spectra were recorded using a Perkin Elmer Spectrum Two FT-IR spectrometer.

Electrochemical techniques

All cyclic voltammetric (CV) and chronopotentiometric measurements were performed at room temperature using an Autolab M101 or a Tenma PSU (purchased from Farnell) and the ElectraSyn 2.0 (purchased from IKA). CV experiments were carried out with a working electrode (GC = glassy carbon, Pt, Au, 1-3 mm diameter), a counter electrode (platinum wire) and a Ag/$AgNO_3$ reference electrode. Cyclic voltammetric measurements of solutions containing HF were carried out using a silver wire reference electrode. All working electrodes were polished before each experiment.

After each CV, the solution was stirred for approximately 10 seconds, whilst being degassed by a stream of N_2.

Warning: Use of HF reagents

The hazards of hydrogen fluoride solutions are well categorised and can be viewed in this SDS (https://www.sigmaaldrich.com/catalog/product/aldrich/184225). Therefore, personal protection is of utmost importance. It is advised to wear two pairs of nitrile gloves when handling, and if the gloves come into contact with HF, they are removed **immediately,** the area affected washed thoroughly with Hexafluorine solution^{TM} (http://www.medicalcare.se/diphoterine-and-hexafluorine), calcium glucanoate gel is applied to the area and medical attention is sought. It is advised that Hexafluorine solution^{TM} and calcium glucanoate gel is kept nearby for personal use.

Optimisation studies

General procedure

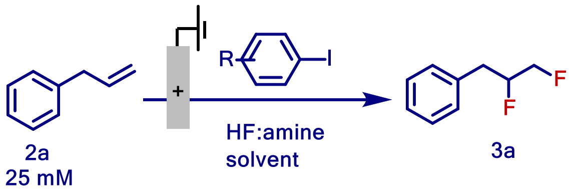

To a 20 mL HDPE vial, equipped with a magnetic stirrer bar, was added iodotoluene (1 eq., 0.1 mmol, 21.8 mg), solvent (1 mL) and HF stock solution (3 mL). Allylbenzene (1 eq., 0.1 mmol, 13.3 µL) was then added in one portion via pipette and the vial was capped with the suba-seal equipped with platinum anode and cathode. The reaction was then subjected to electrolysis (4 F/mol, 3 mA, 4 hrs). The electrodes were then removed, and the vial was capped with an HDPE cap and the reaction was stirred overnight. The reaction solution was then quenched with 50 mL of cold saturated aqueous $CaCO_3$ solution. This was stirred for 30 mins and the mixture was extracted into CH_2Cl_2, dried with Na_2SO_4 and evaporated *en vacuo*. To the mixture was then added $CDCl_3$ (2 mL) and 4,4'-difluorobiphenyl (1 eq., 0.1 mmol, 19.1 mg) at which point the ^{19}F NMR was then measured. The NMR yield was then measured by comparing the integration of the fluorine signal of the product (δ = -231.97 ppm) to that of the internal standard (δ = -115.76 ppm).

Preparation of HF:amine stock solutions

HF: amine stock solutions were made prior to every reaction and were cooled at 2ºC for up to 30 mins before use.

1 mL of a 5.6 HF: amine stock solution (0.44 mL of Py.9HF and 0.56 mL of NEt_3-3HF)

1 mL of a 4.5 HF: amine stock solution (0.32 mL of Py.9HF and 0.68 mL of NEt_3-3HF)

Optimisation of volume of 4.5 HFamine

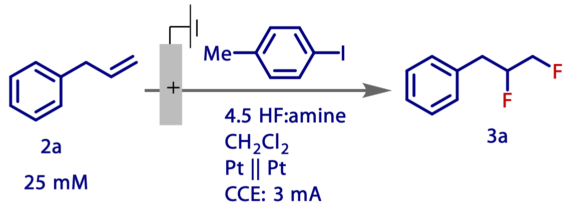

Entry	4.5 HF:amine/ mL	Yield 3a/ %
1	1	30
2	2	37
**3**	**3**	**42**
4	5	37
5	4 (No CH_2Cl_2)	<5

Table S1: Optimisation of iodoarene. Reaction conditions: 1 eq. of iodotoluene, 1 eq. of allylbenzene, 4.5 HF:amine (x mL), CH_2Cl_2 (1 mL) 3 mA, 4 F/mol, undivided cell, Pt || Pt, 12 hours of stirring post electrolysis.

The most optimum (HF:amine):CH_2Cl_2 ratio was found to be 3:1 (**entry 3, table S1**), whilst a decrease or increase led to diminished yield. Using no co-solvent whatsoever, led to decreased yield (**entry 5, table S1**).

Optimisation of iodoarene mediators

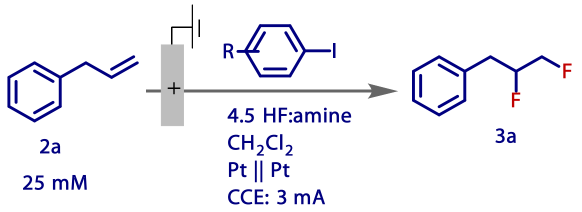

Entry	R	3a/ %
1	4-NO_2	0
2	4-CF_3	0
3	4-F	18
4	4-H	24
5	***4-Me***	***42***
6	4-^{t}Bu	25
7	Mes	20
8	2-Napth	18
9	4-OMe	0

Table S2: Optimisation of iodoarene. Reaction conditions: 1 eq. of iodoarene, 1 eq. of allylbenzene, HF:amine (4.5:1), solvent:CH_2Cl_2, (HF:amine):solvent (3:1), 3 mA, 4 F/mol, undivided cell, Pt || Pt, 12 hours of stirring post electrolysis.

The mass-balance for these reactions was generally low, and the rest of the material detected was either un-reacted starting material or unidentified side-products.

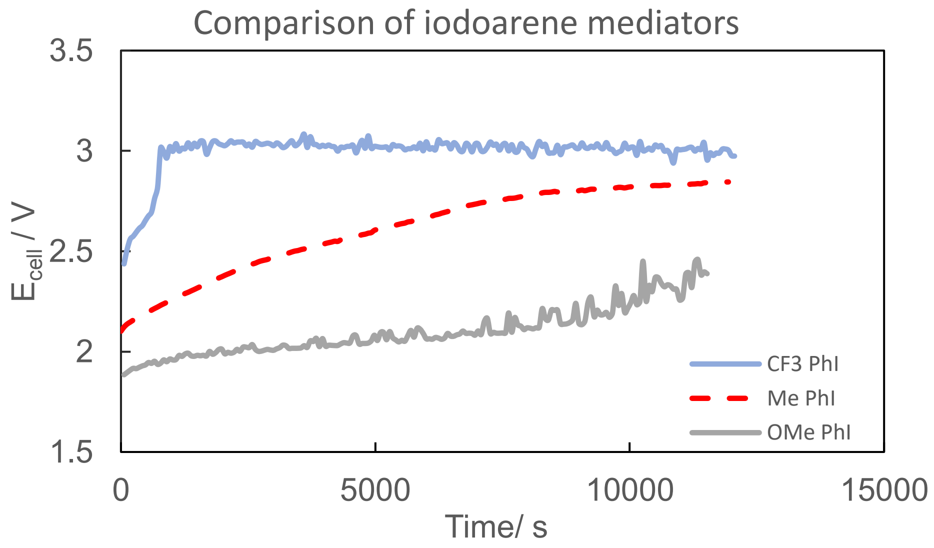

Figure S1: Bulk electrolyses traces of above reaction using para-trifluormethyliodobenzene (**entry 2,** blue line) vs para-methyl iodobenzene (**entry 5**, red, dotted line) vs para methoxy iodobenzene (**entry 9** grey line).

The cell potential, shown in bulk electrolysis trace (**Figure S1**) using para trifluoromethyl iodobenzene rises up to 3 V after ~0.2 F was passed. The use of iodotoluene depicts a steady incline in potential throughout the reaction time. Para methoxy iodobenzene sustains a much lower E_{cell}. Iodotoluene provided the greatest yield, Table S2.

Optimisation of HF:amine ratios

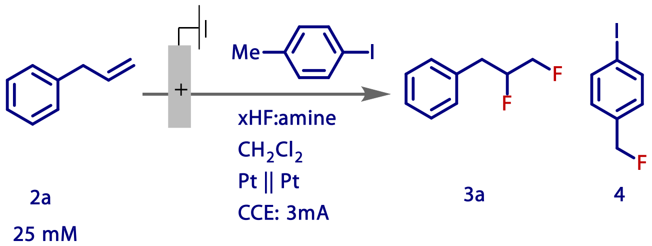

Entry	HF:amine	Yield 3a/ %	Yield 4/ %
1	3	0	18
2	4.5	40	7
3	5	45	5
4	5 [a]	40	7
5	***5.6***	***55***	***<5***
6	5.6 [b]	<5	5
7	6.5	50	<5
8	7	43	<5
9	9	10	<5
10	$^nBu_4N.HF_2$ [c]	0	0
11	$^nBu_4NF.3HF$ [d]	0	<5

Table S3: Reaction conditions: 1 eq. of iodoarene, 1 eq. of allylbenzene, solvent:CH_2Cl_2, (HF:amine):solvent (3:1), 3 mA, 4 F/mol, undivided cell, Pt || Pt, 12 hours of stirring post electrolysis. [a] NEt_3.5HF was used; [b] 5.6HF:amine stock solution prepared using Py.9HF and diluting with pyridine. [c] 100 eq. of $^nBu_4N.HF_2$ used instead of HF:amine. [d] $^nBu_4NF.3HF$ made up by mixing $^nBu_4N.HF_2$ and Py.9HF (3 mL).

The ratios of triethylamine NEt_3•3HF and Py•9HF were varied to prepare a range of HF:amine solutions. The most optimum HF:amine was 5.6 (**entry 4, table S3**) which produced the difluorinated product in 55% yield. Any deviation from this ratio was detrimental to the yield. Interestingly, when utilising a 5.6HF:amine solution, composed of Py•9HF diluted down with pyirinde, a reduced difluorinated product, **3a**, yield was observed. A second trend was observed as lower HF:amine ratios (**entries 1-3, table S3**) produced a significant amount of the benzylic fluorination product of iodotoluene, **4** (5-18%). Additionally, it was shown that this benzylic fluorinated product was not an adequate mediator for the reaction (see Control reaction with the use of benzylic fluorinated iodoarene mediator). Using $^nBu_4N.HF_2$ (**entry 10, table S3**) or a mixture of $^nBu_4N.HF_2$ and Py•9HF (**entry 11, table S3**) to make $^nBu_4NF.3HF$ led to 0% yield of product, **3a**.

Optimisation of solvent

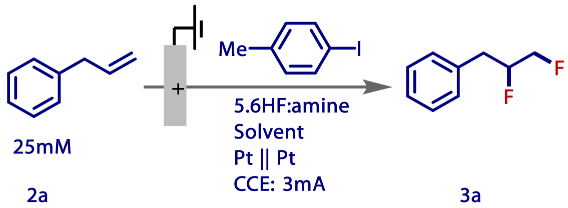

Entry	Solvent	Yield /%
1	CH_2Cl_2	55
2	$CHCl_3$	17
3	DCE	25
4	CH_2Cl_2:HFIP (9:1)	58
5	***CH_2Cl_2:HFIP (7:3)***	***65***
6	CH_2Cl_2:HFIP (1:1)	45
7	DCE:HFIP (7:3)	45
8	HFIP	33

Table S4: Reaction conditions: 1 eq. of iodoarene, 1 eq. of allylbenzene, HF:amine (5.6:1), (HF:amine):solvent (3:1), 3 mA, 4 F/mol, undivided cell, Pt || Pt, 12 hours of stirring post electrolysis.

When optimising the solvent, CH_2Cl_2 (**entry 1, table S4**) was found to perform the best, whilst CH_3CN lead to poor yields. The chronopotentiometry trace from a MeCN reaction (**figure S2**) depicts a continual rise in applied potential, which is typical of electrical insulation from electrode passivation. Varying the ratio of CH_2Cl_2:HFIP (**entries 1-3**, **table S4**) to 7:3 led to the most optimum yield of 65%.

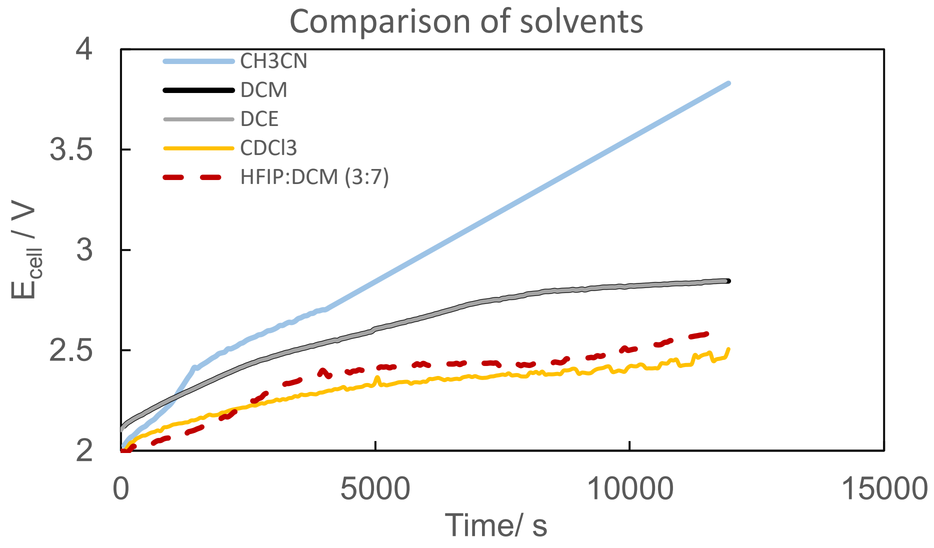

Figure S2: Bulk electrolysis traces of reaction using CH_3CN (orange), CH_2Cl_2 (black), DCE (grey), $CDCl_3$ (yellow) and HFIP:DCM (3:7).

Optimisation of substrate concentration

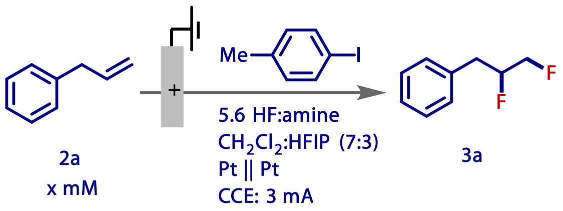

Entry	Conc/ mM	Yield 3a/%
1	25	65
2	100	68
3	200	73
**4**	**300**	**75**
5	400	65

Table S5: Reaction conditions: 1 eq. of iodoarene, 1 eq. of allylbenzene, HF:amine (5.6:1), solvent: CH_2Cl_2:HFIP (7:3), (HF:amine):solvent (3:1), 3 mA, 4 F/mol, undivided cell, Pt || Pt, 12 hours of stirring post electrolysis.

Increasing the concentration from 25 mM to 300 mM effected a steady increase in yield (**entries 1-4, table S5**) to 75%.

Optimisation of applied current

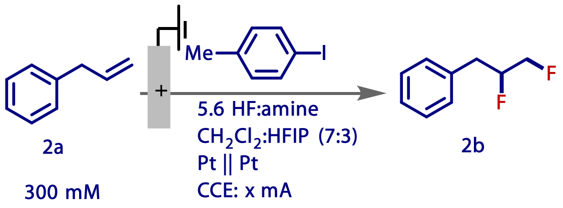

Entry	Current	Yield 2b/%
1	8	73
2	10	75
**3**	**12**	**81**
4	14	77
5	16	53
6	3.8[a]	45

Table S6: Reaction conditions: 1 eq. of iodotoluene, 1 eq. of allylbenzene, HF:amine (5.6:1), (HF:amine):solvent (3:1), x mA, 3.5 F/mol, undivided cell, Pt || Pt, 12 hours of stirring post electrolysis. [a] 0.2 eq. of iodotoluene. Pulse electrolysis conditions: 5 cycles of 6 hours on / 6 hours off. Each cycle = 0.7 F / mol. Total reaction time = 70 hours.

Increasing the current applied from 9 mA to 13 mA (***entries 1-3, table S6***), produced incremental increases in yield up to 81% (most optimized conditions).

Using a pulse electrolysis protocol (5 cycles of 6 hours on / 6 hours off), allowed the use of 0.2 equivalents of iodotoluene which resulted in a 45% yield of **2b**.

Optimisation of anodic material

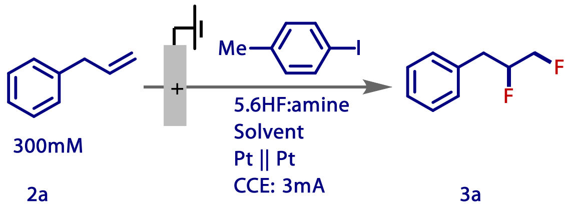

Entry	Anode material	Yield 3a/ %
**1**	**Pt**	**81**
2	Graphite	15
3	RVC	27

Table S7: Reaction conditions: 1 eq. of iodotoluene, 1 eq. of allylbenzene, HF:amine (5.6:1), (HF:amine):solvent (3:1), 12 mA, 3.5 F/mol, undivided cell, Pt cathode use, 12 hours of stirring post electrolysis.

The use of carbon anodes (**entries 2 and 3**, **table S5**) led to decreased yield.

Optimisation of ‘ex-cell’ method

CV studies revealed that the oxidation potential of 4-allyltoluene is lower than that of the 4-iodotoluene mediator, (**Figure S3**). Therefore, the oxidation of iodotoluene was performed first before addition of substrate.

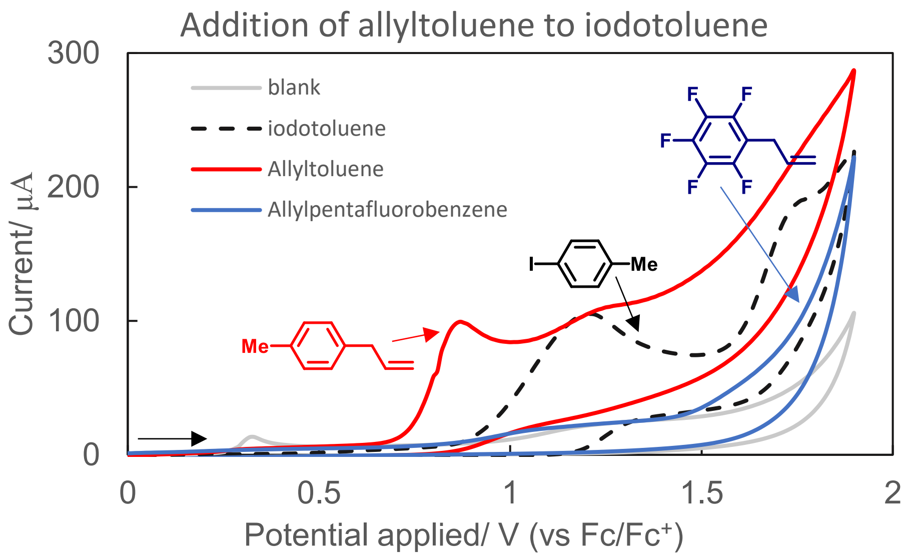

Figure S3: CV of iodotoluene (5 mM, dashed, black line), 4-allyltoluene (5 mM, red line) and allylpentafluorobenzene (5 mM, blue line) to this solution. CV conditions are the same as the reaction conditions: HFIP:DCM (3:7) = solvent, HF:amine (5.6:1), (HF:amine):solvent (3:1), Pt working electrode, Pt counter electrode, silver wire as reference electrode, 0.1 V/s.

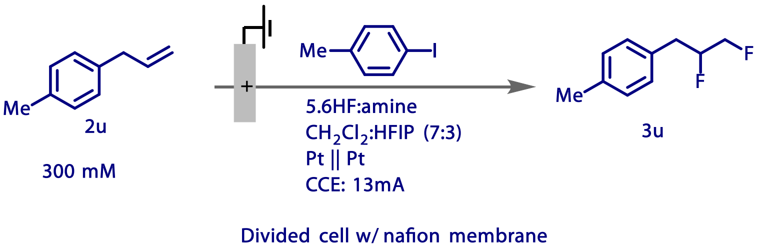

Entry	*Deviation from standard conditions*	*Yield 3t/ %*
1	**none**	**73**
2	2 equivalents of iodotoluene	60
3	Para-CF_3 iodobenzene instead of iodotoluene	15
4	Iodo-mesitylene instead of iodotoluene	20
5	No HFIP instead of CH_2Cl_2:HFIP (7:3)	15
6	Undivided cell with allytoluene added at the start	45
7	Undivided cell instead of divided cell	15

Table S8: Optimisation table for ex-cell method. Reaction conditions: 1 eq. of iodotoluene, 1 eq. of allyltoluene (1.2 mmol), HF:amine (5.6:1), (HF:amine):solvent (3:1), 13.4 mA, 3F/mol, divided cell, Pt || Pt. After electrolysis addition of allyltoluene, and then 12 hours of stirring. Yields were determined by ^{19}F NMR spectroscopy using 4,4'-difluorobiphenyl as the internal standard.

Note, for the best yields and reproducibility a fresh piece of nafion was used for each reaction, as repeated use led to decreased yields.

Formation of *p*-tolyl difluoro λ3-iodane, 1a

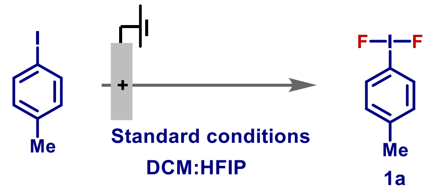

In order to establish the nature of the hypervalent iodine species produced during electrolysis, an aliquot of the reaction mixture from an ex-cell method reaction was compared to a genuine sample of *p*-tolyl difluoro λ3-iodane, **1a** (p-TolIF$_2$) under reaction conditions.

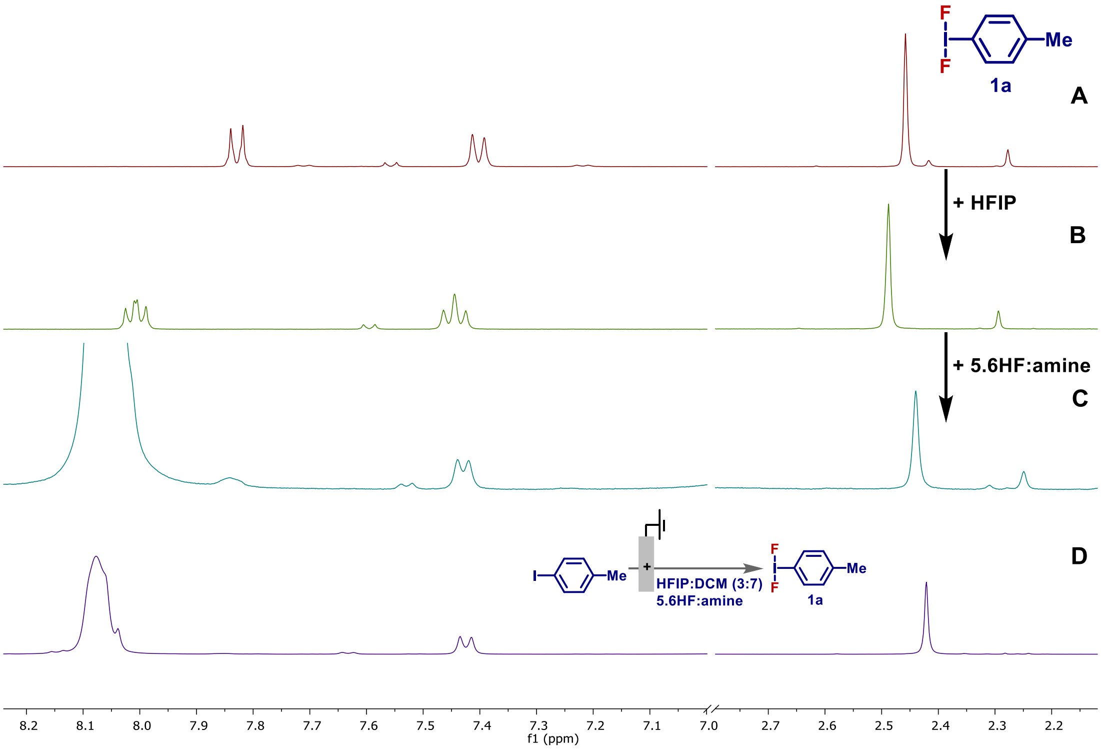

Figure S4. **A** = p-TolIF$_2$ in CH$_2$Cl$_2$, **B** = p-TolIF$_2$ in CH$_2$Cl$_2$/ HFIP (7:3), **C** = p-TolIF$_2$ in CH$_2$Cl$_2$/ HFIP (7:3) and 5.6HF:amine (3:1) (i.e reaction conditions). **D** = aliquot from post ex-cell method: 1 eq. of iodotoluene, HF:amine (5.6:1), (HF:amine):solvent (3:1), 13.4 mA, 3F/mol, undivided cell, Pt || Pt.

A sample of p-TolIF$_2$ (**1a**) in CH$_2$Cl$_2$ (A) shows 2 apparent doublets (2 x C$_{Ar}$ -H), but when HFIP (B) is added to this solution, multiplicities change to exhibit two apparent triplets and the downfield signal shifts further downfield from 7.82 to 8.00 ppm. Addition of 5.6HF:amine to this solution (C) reverts the apparent triplets back to apparent doublets. Finally, an aliquot from the electrochemical ex-cell method (D) is comparable to **1a** under the reaction conditions (C).

Stability of *p*-tolyl difluoro λ^3-iodane, 1a, under reaction conditions

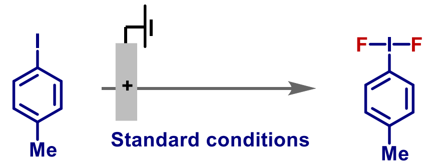

1a

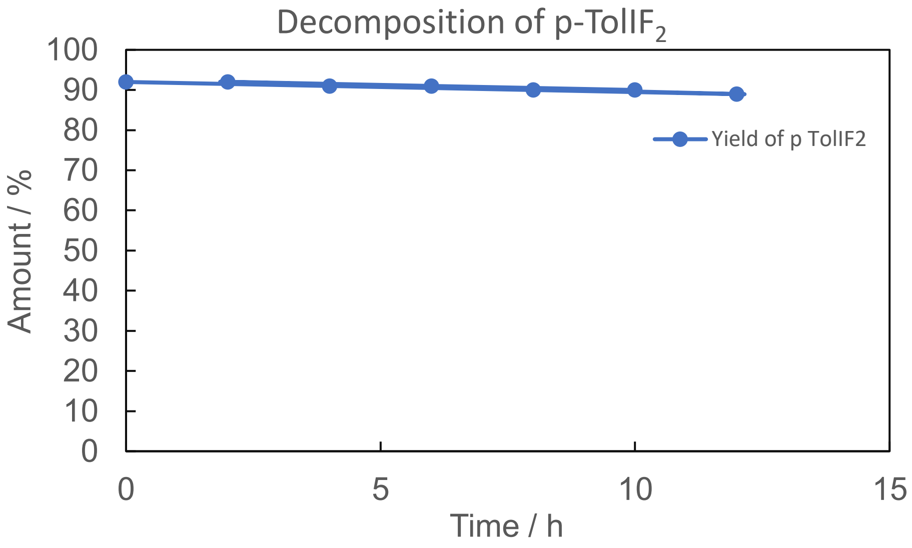

Figure S5. Decomposition of *p*-tolyl difluoro λ^3-iodane, 1a, (p-TolIF$_2$) post electrolysis of an ex-cell method reaction.

Next the stability of p-tolyl difluoro λ^3-iodane, **1a** was investigated. An aliquot of a reaction mixture (ex-cell) post electrolysis was taken and an NMR spectrum every 2 hours was measured. Using an internal standard (ethyl fluoroacetate) the amount of **1a** was calculated. Under the reaction mixture conditions, there was a 3% decrease in the amount of p-TolIF$_2$ post electrolysis.

Investigation into the role of HFIP

In order to ascertain the beneficial role of HFIP, NMR and CV studies were carried out. The oxidation of iodotoluene was performed in various HFIP:DCM ratios in a divided cell. The NMR yields of *p*-tolyl difluoro λ^3-iodane (**1a** $ArIF_2$) were recorded (against an internal standard, ethyl fluoro acetate: ^{1}H signal = 4.62 ppm) (**Table S9**). We observed that utilising a ratio of HFIP:DCM (3:7) produced the largest amount of **1a** (92%). Despite the air-sensitivity of this species, it was found to be stable in solution under the reaction conditions for the time period of the experiment. Allylpentafluorobenzene **1e** (1 eq.) was then added to the solutions, and we observed that HFIP:DCM (3:7) produced the highest yield of difluorination product. Furthermore, we observed that mixtures with more HFIP present, led to higher conversions of **1a** to difluorination product **3e:** 100% HFIP led to 100% conversion of **1a** to **3e**, whereas 30% HFIP led to 65% conversion of **1a** to **3e**. Thus, HFIP promotes difluorination from **1a**, but a dilution of it is important (HFIP:DCM (3:7)) to achieve the highest level of **1a** formation.

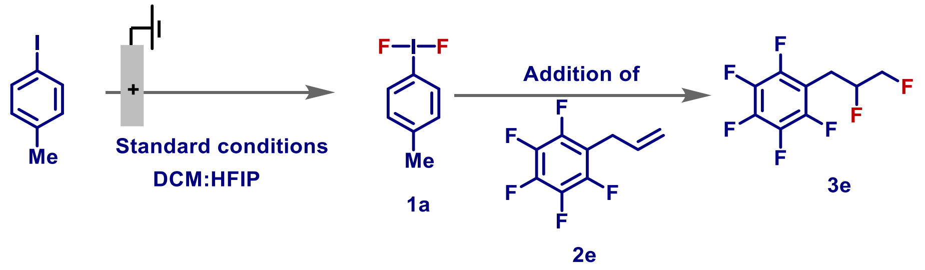

DCM:HFIP	Yield $ArIF_2$ 1a/ %	Yield, difluorination 3e/ %	Conversion of 1a to product 3e/ %
1:0	74	49	66
7:3	92	60	65
1:1	55	50	91
0:1	13	13	100

Table S9: Standard conditions: 1 eq. of iodotoluene, 1 eq. of allyltoluene (1.2 mmol), HF:amine (5.6:1), HF/amine:solvent (3:1), 12 mA, 3F/mol, undivided cell, Pt || Pt. Oxidation potentials of iodotoluene with varying HFIP:DCM ratios and NMR yield of $ArIF_2$ (**1a**) after electrolysis before addition of substrate using trifluoro ethyl acetate as internal standard.

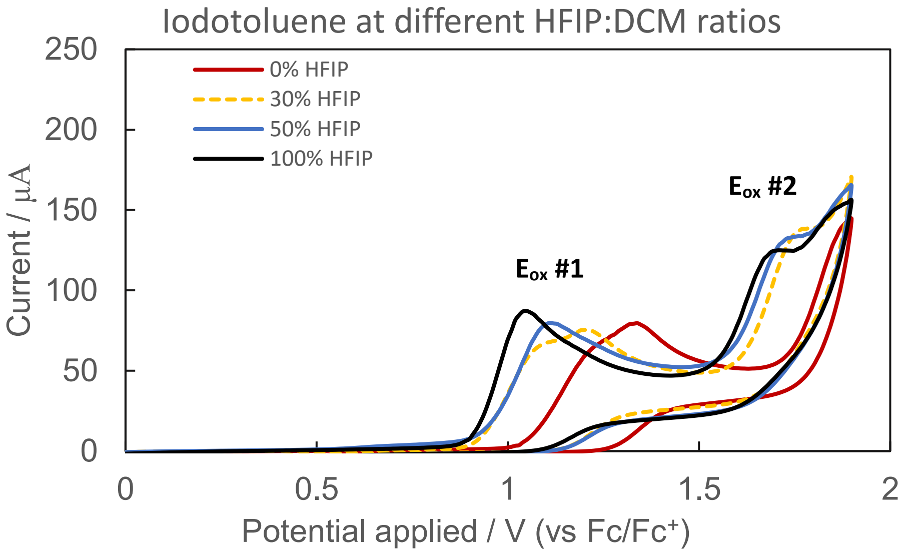

Figure S6. CV studies of iodotoluene at different CH_2Cl_2:HFIP ratios. 0% HFIP (red line), 30% HFIP (orange, dashed line), 50% HFIP (blue line) and 100% HFIP (black line).

CV studies of iodotoluene under reaction conditions using varying HFIP:CH_2Cl_2 ratios were conducted. As shown, the oxidation potential of both E_{ox} 1 and 2, decreases as more HFIP is included in the reaction.

Control reactions

Use of Benzylic fluorinated iodoarene (4) as mediator

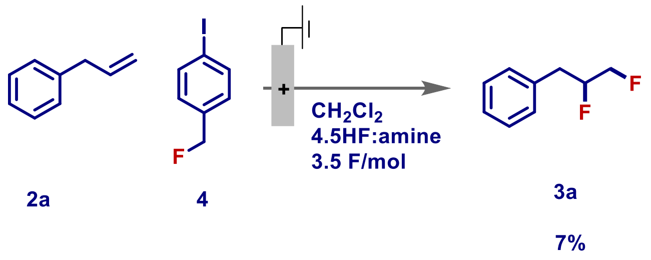

To a 20 mL HDPE vial, equipped with a magnetic stirrer bar, was added benzylic fluorinated iodotoluene (**4**) (1 eq., 0.1 mmol, 23.6 mg), CH_2Cl_2 (0.7 mL), HFIP (0.3 mL) and 5.6HF:amine stock solution (3 mL). Allylbenzene (1 eq., 0.1 mmol, 13.5 µL) was added and the vial was capped with a suba-seal equipped with platinum anode and cathode. The reaction was then subjected to electrolysis (3.5 F/mol, 13 mA, 6 hrs). The electrodes were then removed, and the vial was capped with an HDPE cap and the reaction was stirred overnight. The reaction solution was then quenched with 50 mL of cold (0 ºC) saturated aqueous $CaCO_3$ solution. This stirred for 1 hour until the aqueous layer measured pH 7. The mixture was extracted into CH_2Cl_2, dried with Na_2SO_4 and evaporated *en vacuo*.

Without the use of iodotoluene mediator

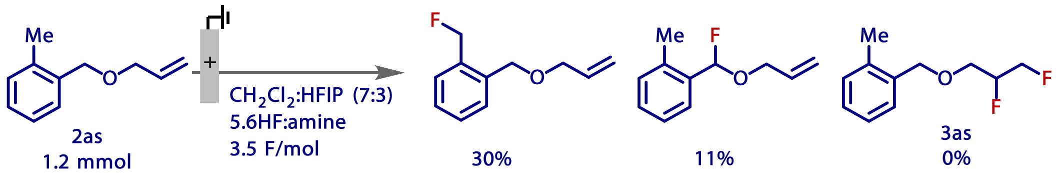

To a 20 mL HDPE vial, equipped with a magnetic stirrer bar, was added substrate (1 eq., 1.2 mmol, 195 mg), CH_2Cl_2 (0.7 mL), HFIP (0.3 mL) and 5.6HF:amine stock solution (3 mL). The vial was capped with a suba-seal equipped with platinum anode and cathode. The reaction was then subjected to electrolysis (3.5 F/mol, 13 mA, 6 hrs). The electrodes were then removed, and the vial was capped with an HDPE cap and the reaction was stirred overnight. The reaction solution was then quenched with

50 mL of cold (0 ºC) saturated aqueous $CaCO_3$ solution. This stirred for 1 hour until the aqueous layer measured pH 7. The mixture was extracted into CH_2Cl_2, dried with Na_2SO_4 and evaporated *en vacuo*.

General Synthetic Procedures for Substrates

A: Allylation Procedure 1

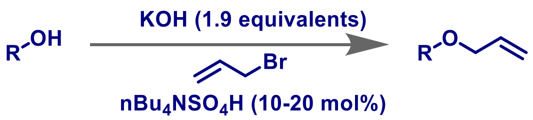

To a solution of alcohol (1 eq.of alcohol, 0.5 M) in allyl bromide (1 eq.) was added KOH (1.9 equivalents), and tetrabutyl ammonium bisulfate (10-20 mol %). The mixture was stirred at room temperature until the reaction was shown to be complete by TLC analysis. Water (20 mL) was then added, and the aqueous layer was extracted with diethyl ether (10 mL, 3-5 times). The combined organic layers were washed once with water (5 mL), and once with brine (5 mL) before being dried over magnesium sulfate, filtered, and concentrated *in-vacuo* to afford the crude product. The crude product was purified by silica gel flash column chromatography.

B: Allylation Procedure 2

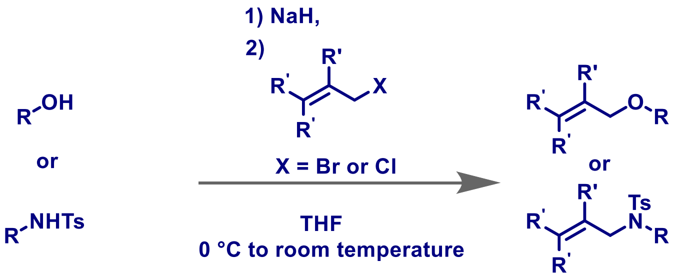

To a two-neck flame-dried round-bottomed flask, under nitrogen, was added sodium hydride (60% in mineral oil, 1.4-2 equivalents) and dry THF (sufficient volume to be 0.3 M concentration of alcohol or amine), and the mixture was cooled to 0 °C. The solution was stirred as the corresponding alcohol or amine (1 equivalent) was added drop-wise. The solution was then warmed to room temperature and left to stir for one hour. The reaction mixture was cooled again to 0 °C and the corresponding allylic bromide or chloride substrate (1.4-2 equivalents) was added drop-wise. The reaction was warmed to room temperature, stirred and monitored by TLC. Upon complete conversion of the alcohol or amine, the reaction mixture was cooled to 0 °C and quenched by drop-wise addition of water. The aqueous and organic layers were separated, and the aqueous layer was extracted with diethyl ether (20 mL x 3). The combined organic layers were washed once with water and once with brine before

being dried over magnesium sulfate, filtered, and concentrated *in-vacuo* to afford the crude product. The crude product was purified by silica gel flash column chromatography.

C: Allylation procedure 3

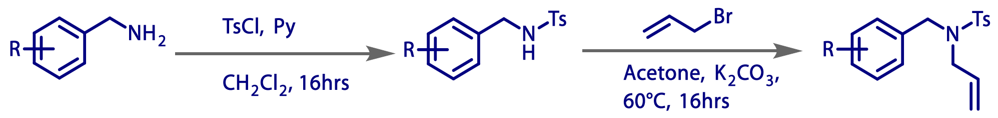

To a flame-dried 2-neck round bottom flask was added benzylamine (1 eq.), anhydrous CH_2Cl_2 (0.2 M) and pyridine (3 eq.). This mixture stirred for 5 minutes and then 4-methylbenzenesulfonyl chloride (1.2 eq.) was added in portions. After stirring at room temperature for 4 hours, 1 M HCl (10 mL) was added. The organic layer was extracted with EtOAc (2 x 20 mL) and dried with $NaSO_4$. The filtrate was dried *en vacuo* and brought forward to the next step.

To a single neck round bottom flask was added the crude from the previous step (5 mmol), K_2CO_3 (3 eq.), acetone (15 mL) and allyl bromide (1.5 eq.). The reaction mixture was then stirred overnight at reflux. The reaction mixture was then filtered, solvent evaporated *en vacuo*. CH_2Cl_2 (20 mL) and water (20mL) was added to the filtrate. The organic layer was extracted with CH_2Cl_2 (2 x 20mL), dried with Na_2SO_4 and then dried *en vacuo*.

D: Grignard procedure

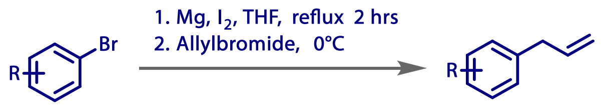

A dry round bottomed flask was charged with a stir bar and Mg (1.2 equiv.) under a N_2 atmosphere. Aryl bromide (1 equiv.) and an iodide crystal were dissolved in THF in a dry Schlenk tube (under nitrogen). Part of the solution (~2 mL) was added to the flask and stirred. After the colour of the mixture suddenly faded, the rest of the solution was added dropwise via a dropping funnel. The mixture was then stirred at reflux for 2 h. The reaction mixture was cooled to 0 ^{o}C and allyl bromide (1.5 equiv.) was carefully added and stirred until complete conversion of the alcohol was evident by TLC analysis. The reaction mixture was cooled to 0 ^{o}C and aqueous saturated NH_4Cl solution was slowly added. The aqueous phase and organic layers were separated,

and the aqueous phase was extracted with Et_2O. The combined organic phases were dried with $MgSO_4$ and concentrated under reduced pressure. The crude product was purified by silica gel flash column chromatography (unless stated otherwise).

E: Allylation Procedure 4

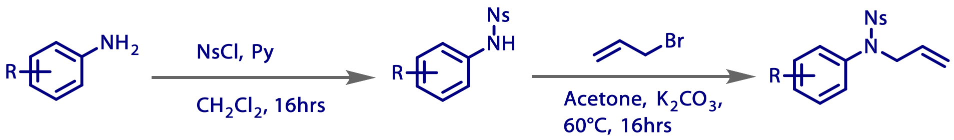

To a flame-dried 2-neck round bottom flask was added aniline (1 eq.), anhydrous CH_2Cl_2 (0.2 M) and pyridine (3 eq.). This mixture stirred for 5 minutes and then 4-nitrobenzenesulfonyl chloride (2 eq.) was added in portions. After stirring at room temperature for 4 hours, $HCl_{(aq)}$ (1 M, 10 mL) was added. The organic layer was extracted (2 x 20 mL) and dried with $NaSO_4$. The filtrate was dried *en vacuo* and brought forward to the next step.

To a single neck round bottom flask was added the crude from the previous step (5 mmol), K_2CO_3 (3 eq.), acetone (15 mL) and ally bromide (1.5 eq.). The reaction mixture was then heated overnight at reflux. The reaction mixture was then filtered, and water was added to the filtrate. The organic layer was extracted (2 x 20 mL), dried with Na_2SO_4 and then dried *en vacuo*.

F: Wittig procedure

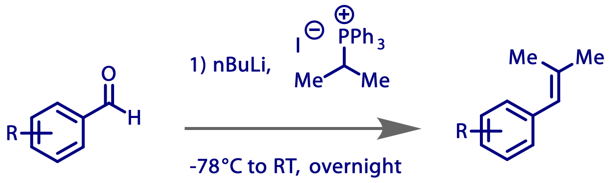

To a flame-dried flask, under nitrogen, at -78 °C, was added isopropyltriphenylphosphonium iodide (1.2 eq.) and THF (40 mL). To the stirred solution was added nBuLi (1.4 eq., 7 mmol, 4.58 mL, 1.53 M solution in hexane) drop-wise. The reaction was stirred for one hour at room temperature before being cooled to -78 °C. Benzaldehyde (1 eq.) was then added drop-wise. The flask was left to stir at room temperature until TLC analysis indicated full consumption of the aldehyde. The reaction was quenched by the addition of brine. The aqueous and organic layers were separated. The aqueous layer was extracted with diethyl ether (5 x 15 mL). The

combined organic layers were washed with water (20 mL) and brine (20 mL) before being dried over magnesium sulfate, filtered, and concentrated *in-vacuo* to afford the crude product. The crude product was purified by silica gel flash column chromatography.

G: Mitsunobu procedure

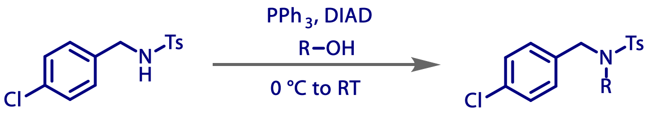

Under a nitrogen atmosphere to a Schlenk tube, was added triphenylphosphine (1 eq., 5 mmol, 1.31 g), THF (20 mL), N-(4-chlorobenzyl)-4-methylbenzenesulfonamide (1 eq., 5 mmol, 2.94 g) and alcohol (1 eq. 5 mmol). After this was cooled to 0 ºC, DIAD (1 eq., 5 mmol, 983 µL) was added dropwise until the solution turns yellow and the reaction was left to stir overnight. The mixture was then dried *en vacuo*, distilled water (20 mL) was added and then extracted into CH_2Cl_2 (3 x 25 mL). The combined organic phases were dried with $MgSO_4$ and concentrated *en vacuo*. The crude product was purified by silica gel flash chromatography.

H: Mesylation procedure 1

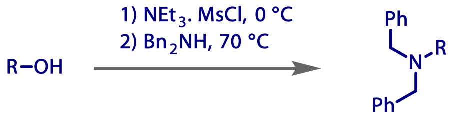

To a round bottom flask was added alcohol (10 mmol, 1 eq.) and CH_2Cl_2 (10 mL). At 0 ºC, triethylamine (20 mmol, 0.6 mL, 2 eq.) and methysulfonic chloride (1.5 eq., 15 mmol, 0.64 mL) were added. After stirring for 1 hour, the reaction mixture was washed with 1 M HCl (25 mL) and extracted into CH_2Cl_2, dried and evaporated *en vacuo*. The resultant oil was then dissolved in ethanol (20 mL), and dibenzylamine (2.5 eq., 25 mmol, 2.65 mL) was added. The resultant mixture was allowed to stir at 70 ºC for 16 hours, and then extracted into CH_2Cl_2 (25 mL). The organic extract was washed with saturated aqueous $NaHCO_3$ (50 mL), saturated NH_4Cl, saturated aqueous $NaHCO_3$, dried and evaporated *en vacuo*.

Characterisation of substrates

Undec-10-en-1-yl 4-methylbenzenesulfonate, 2g

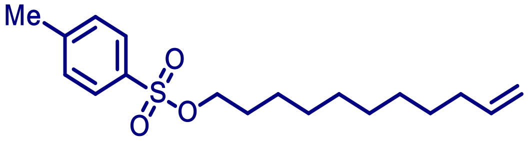

Synthesised according to literature procedure [2] using undec-en-1-ol (5 mmol) and was purified by silica gel flash column chromatography (10 % EtOAc:Hexane) to yield a colourless oil (736 mg, 45 %).

R_f = 0.25 (10 % EtOAc:Hexane)

^{1}H NMR (300 MHz, $CDCl_3$): δ = 7.79 (2H, d, *J* = 8.3 Hz), 7.34 (2H, d, *J* = 8.5 Hz), 5.80 (1H, ddt, *J* = 16.9, 10.1, 6.7 Hz), 5.03 – 4.90 (2H, m), 4.01 (2H, t, *J* = 6.5 Hz), 2.45 (3H, s), 2.03 (2H, q, *J* = 6.9 Hz), 1.69 – 1.57 (12H, m), 1.22 (4H, m)

^{13}C NMR (101 MHz, $CDCl_3$): δ = 144.7, 129.9, 128.0, 114.3, 70.8, 33.9, 29.4, 29.1, 29.0-28.9 (overlapping peaks) 25.4, 21.7

These data are consistent with that previously reported.[2]

Dodec-11-en-1-yl 4-methoxybenzoate, 2h

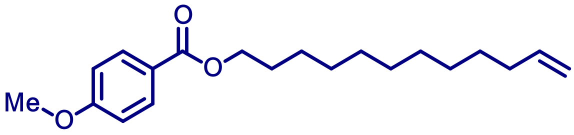

Synthesised according to literature procedure [2] using undec-en-1-ol (5 mmol) and was purified by silica gel flash column chromatography (5 % EtOAc:Hexane) to yield a colourless oil (1.09 g, 45 %).

R_f = 0.20 (5 % EtOAc:Hexane)

^{1}H NMR (400 MHz, $CDCl_3$): δ = 8.02 – 7.97 (2H, m), 6.95 – 6.88 (2H, m), 5.81 (1H, ddt, *J* = 17.1, 10.3, 6.7 Hz), 4.99 (1H, ddt, *J* = 17.1, 2.3, 1.6 Hz), 4.93 (1H, ddt, *J* = 10.1, 2.2, 1.2 Hz), 4.28 (2H, t, *J* = 6.7 Hz), 3.86 (3H, s), 2.08 – 2.00 (12H, m), 1.75 (2H, dq, *J* = 8.1, 6.8 Hz), 1.49 – 1.24 (2H, m).

^{13}C NMR (101 MHz, CDCl$_3$): δ = 166.6, 163.3, 139.3, 131.6, 123.1, 114.2, 113.6, 64.9, 55.5, 33.9, 29.5, 29.5, 29.4, 29.2, 29.0, 28.9, 26.1.

These data are consistent with that previously reported.[2]

1-((Allyloxy)methyl)-4-fluorobenzene, 2j

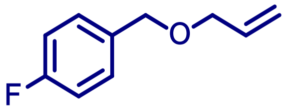

This substrate was synthesised from 4-Fluorobenzyl alcohol (20 mmol) using **allylation procedure 1** and was purified by silica gel column chromatography (5% EtOAc:Hexane) to yield a colourless oil (1.16 g, 35 %).

^{1}H NMR (400 MHz, CDCl$_3$): δ = 7.32 – 7.28 (2H, m), 7.03 – 6.98 (2H, m), 5.95 (1H,ddt, *J* = 16.1, 10.6, 5.6 Hz), 5.31 (1H, dt, *J* = 17.2, 1.7 Hz), 5.22 (1H, dq, *J* = 10.3, 1.4 Hz), 4.47 (2H, s),4.02 (2H, dt, *J* = 5.6, 1.4 Hz).

^{13}C NMR (101 MHz, CDCl$_3$): δ = 162.4 (d, *J* = 245.5 Hz), 134.7, 134.1 (d, *J* = 3.2 Hz), 129.5 (d, *J* = 8.1 Hz), 117.3, 115.3 (d, *J* = 21.4 Hz), 71.5, 71.2.

These data are consistent with that previously reported.[3]

1-((Allyloxy)methyl)-4-nitrobenzene, 2k

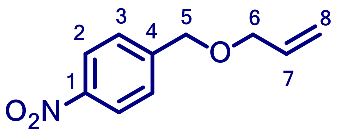

This substrate was synthesised from 4-nitro benzyl alcohol (10 mmol) using **allylation procedure 1** and was purified by silica gel column chromatography (10% EtOAc:Hexane) to yield an orange oil (621 mg, 32 %).

R_f = 0.5 (10% EtOAc:Hexane)

^{1}H NMR (400 MHz, CDCl$_3$): δ = 8.21 (2H, d, *J* = 8.7 Hz, *H^2*), 7.51 (2H, d, *J* = 8.6 Hz, *H^3*), 5.96 (1H, ddt, *J* = 16.1, 10.8, 5.6 Hz, *H^7*), 5.34 (1H, ddt, *J* = 17.2, 1.6, 1.3 Hz, *H^8*), 5.25 (1H, dd, *J* = 10.2, 1.2 Hz, *H^8*), 4.62 (2H, s, *H^5*), 4.09 (2H, dt, *J* = 5.6, 1.2 Hz, *H^6*).

^{13}C NMR (101 MHz, CDCl$_3$): δ = 147.5 (1C, *C^1*), 146.2 (1C, *C^4*), 134.2 (1C, *C^7*), 127.8 (2C, *C^3*) , 123.8 (2C, *C^2*), 117.9 (1C, *C^8*), 71.9 (1C, *C^6*), 70.9 (1C, *C^5*)

HRMS (APCI) calc: $[M+H]^+$ ($C_{10}H_{11}NO_3$) 194.0812; measured: 194.0804 = 4.12 ppm difference

IR (neat) ν_{max}/ cm^{-1}: 3080, 2855, 1604, 1516, 1341, 1084, 1014, 989, 928, 841, 737, 698

1-((Allyloxy)methyl)-2-bromobenzene, 2l

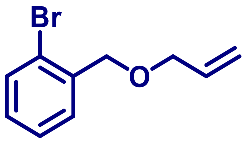

This substrate was synthesised from (2-bromophenyl)methanol (20 mmol) using **allylation procedure 1** was purified by silica gel column chromatography (100% Hexane) to yield a colourless oil (3.54 g, 78 %).

R_f = 0.3 (Hexane)

^{1}H NMR (400 MHz, CDCl$_3$): δ = 7.52 (1H, dd, *J* = 8.0, 1.2 Hz), 7.51 – 7.46 (1H, m), 7.34 – 7.27 (1H, m), 7.16 – 7.10 (1H, m), 6.04 – 5.92 (1H, ddt *J* = 5.56 Hz, 10.4 Hz, 25.22 Hz), 5.34 (1H, dq, *J* = 17.2, 1.7 Hz), 5.22 (1H, ddt, 10.4 Hz, 1.68 Hz, 1.28 Hz), 4.58 (2H, s), 4.10 (2H, dt, *J* = 5.6, 1.4 Hz)

^{13}C NMR (101 MHz, CDCl$_3$): δ = 137.8, 134.6, 132.6, 129.1, 129.0, 127.5, 122.7, 117.4, 71.8, 71.5.

These data are consistent with that previously reported.[4]

2-((Allyloxy)methyl)pyridine, 2m

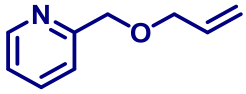

This substrate was synthesised from pyridin-2-ylmethanol (15 mmol) using **allylation procedure 1** and was purified by silica gel column chromatography (30% EtOAc:Hexane) to yield an orange oil (1.08 g, 25 %).

^{1}H NMR (400 MHz, CDCl$_3$): δ = 8.54-8.52 (1H, m), 7.67 (1H, td, *J*= 7.6, 1.8 Hz), 7.44 (1H, d, *J*= 8.1 Hz), 7.17 (1H, m), 6.05–5.89 (1H, m), 5.32 (1H, dp, *J* = 17.3, 1.6Hz), 5.21 (1H, ddq, *J*= 10.4, 3.0, 1.7 Hz), 4.63 (2H, s), 4.11 (2H, dt, *J*= 5.64, 1.28 Hz)

^{13}C NMR (101 MHz, CDCl$_3$): δ = 158.7, 149.2, 136.8, 134.6, 122.6, 121.5, 117.5, 73.2, 72.0

These data are consistent with that previously reported.[5]

4-((Allyloxy)methyl)pyridine, 2n

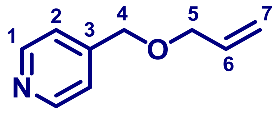

This substrate was synthesised from 4-(bromomethyl)pyridine hydrobromide (5.81 mmol) and allyl alcohol (6.97 mmol) using **allylation procedure 2** to yield an orange oil (639 mg, 74 %).

R$_f$ = 0.4 (5% MeOH in DCM)

^{1}H NMR (400 MHz, CDCl$_3$): δ = 8.59 – 8.56 (2H, m, *H^1*), 7.28 – 7.25 (2H, m, *H^2*), 5.96 (1H, ddtd, *J* = 16.9, 10.4, 5.6, 0.8 Hz, *H^6*), 5.33 (1H, dqd, *J* = 17.2, 1.6, 0.9 Hz, *H^7*), 6.01 – 5.90 (1H, m, *H^7*), 4.54 (2H, d, *J* = 0.7 Hz, *H^4*), 4.07 (2H, dt, *J* = 5.6, 1.5 Hz, *H^5*).

^{13}C NMR (101 MHz, CDCl$_3$): δ = 149.9 (2C, *C^1*), 147.7 (1C, *C^3*), 134.3 (1C, *C^6*), 121.9 (2C, *C^2*), 117.7 (1C, *C^7*), 71.8 (1C, *C^5*), 70.4 (1C, *C^4*).

HRMS (ESI$^+$) calc: [M+H]$^+$ ($C_9H_{11}NO$) 150.0906; measured: 150.0913 = 4.60 ppm difference

IR (neat) v_{max}/ cm^{-1}: 2854, 1604, 1562, 1350, 1414, 1219, 1097, 992, 926, 796

1-((Allyloxy)methyl)-3,5-dinitrobenzene, 2o

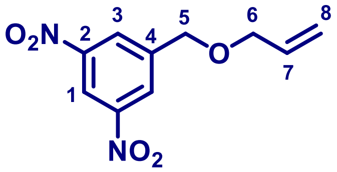

This substrate was synthesised from (3,5-dinitrophenyl)methanol (5 mmol) using **allylation procedure 1** and was purified by silica gel column chromatography (15% EtOAc:Hexane) to yield a dark orange oil (520 mg, 43 %).

R_f = 0.25 (20% Et_2O:Hexane)

^{1}H NMR (400 MHz, $CDCl_3$): δ = 8.94 (1H, td, *J* = 2.1, 0.6 Hz, H^1), 8.54 (2H, dt, *J* = 2.1, 0.8 Hz, H^3), 5.97 (1H, ddt, *J* = 17.2, 10.4, 5.7 Hz, H^7), 5.36 (1H, dq, *J* = 17.2, 1.6 Hz, H^8), 5.29 (1H, dq, *J* = 10.4, 1.2 Hz, H^8), 4.71 (2H, q, *J* = 0.8 Hz, H^5), 4.16 (2H, dt, *J* = 5.7, 1.4 Hz, H^6).

^{13}C NMR (101 MHz, $CDCl_3$): δ = 148.7 (2C, C^2), 143.5 (1C, C^4), 133.7 (1C, C^7), 127.2 (2C, C^3), 118.5 (1C, C^8), 117.9 (1C, C^1), 72.3 (1C, C^6), 69.8 (1C, C^5).

HRMS (APCI) calc: $[M+H]^+$ ($C_{10}H_{10}N_2O_5$) 239.0668; measured: 239.0656 = 3.68 ppm difference

IR (neat) v_{max}/ cm^{-1} = 1541, 1344, 729

N-allyl-N-benzyl-4-methylbenzenesulfonamide, 2p

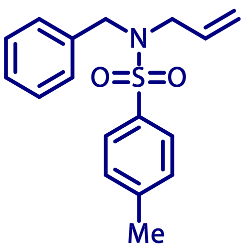

This substrate was synthesised from benzylamine (5.5 mmol) using **allylation procedure 3** and was purified by silica gel column chromatography (10% Et_2O:Hexane) yield an off-white solid (1.1 g, 65 %).

R_f = 0.4 (10% Et_2O:Hexane)

^{1}H NMR (500 MHz, $CDCl_3$): δ = 7.74 (2H, d, *J* = 8.3 Hz,), 7.33 – 7.24 (7H, m), 5.47 (1h, ddt, *J* = 16.8, 10.1, 6.6 Hz,), 5.08 – 4.97 (2H, m), 4.34 (2H, s), 3.75 (2H, dt, *J* = 6.6, 1.3 Hz), 2.44 (3H, s).

^{13}C NMR (151 MHz, $CDCl_3$): δ = 143.4, 137.7, 136.1, 132.3, 129.9, 128.7, 128.6, 127.8, 127.4, 50.3, 49.6, 21.7.

These data are consistent with that previously reported.[5]

N-Allyl-N-(4-chlorobenzyl)-4-methylbenzenesulfonamide, 2q

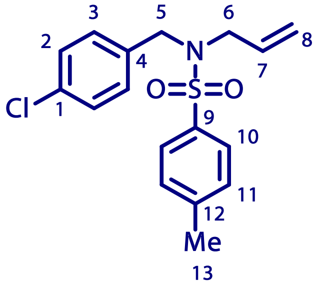

This substrate, **2p**, was synthesised from 4-chlorobenzylamine (5 mmol) using **allylation procedure 3** and was purified by silica-gel flash column chromatography (10 % ethyl acetate: Hexane) **3** to yield a yellow oil (415 mg, 47 %).

R_f = 0.19 (10 % EtOAc:Hexane)

^{1}H NMR (400 MHz, CDCl$_3$): δ = 7.73 (2H, d, *J* = 8.4 Hz, *H^{10}*), 7.32 (2H, d, *J* = 8.4 Hz, *H^{11}*), 7.28 (2H, d, *J* = 8.2 Hz, *H^2*), 7.20 (2H, d, *J* = 8.2 Hz, *H^3*), 5.45 (1H, ddt, *J* = 16.8, 10.2, 6.5 Hz, *H^{12}*), 5.06 (1H, dt, *J* = 10.2, 1.4 Hz, *H^8*), 4.99 (1H, dt, *J* = 17.0, 1.4 Hz, *H^8*), 4.29 (2H, s, *H^5*), 3.74 (2H, dd, *J* = 6.6, 1.4 Hz, *H^6*), 2.45 (3H, s, *H^{13}*).

^{13}C NMR (101 MHz, CDCl$_3$): δ = 143.6 (1C, *C^{12}*), 137.4 (1C, *C^1*), 134.8 (1C, *C^4*), 133.6 (1C, *C^9*), 132.2 (1C, *C^8*), 129.9 (2C, *C^{10}*), 129.9 (2C, *C^2*) 128.8 (2C, *C^3*), 127.2 (2C, *C^{11}*), 119.7 (1C, *C^7*), 49.9 (1C, *C^6*), 49.7 (1C, *C^5*), 21.6 (1C, *C^{13}*).

HRMS (ESI) calc: $[M+H]^+$ ($C_{17}H_{18}ClNO_2S$) 336.0820; measured: 336.0820 = 0.00 ppm difference

IR (neat) $ν_{max}$/ cm^{-1}: 2922, 2853, 1457, 1160, 664, 547

N-allyl-4-methyl-N-(4-(trifluoromethyl)benzyl)benzenesulfonamide, 2r

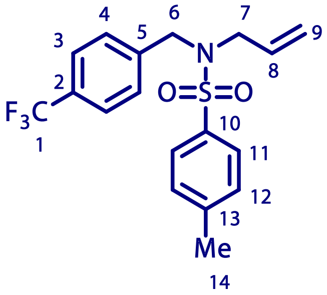

This substrate, **2q**, was synthesised from 4-trifluoromethylbenzylamine (5 mmol) using **allylation procedure 3** and was purified by silica-gel flash column chromatography (15 % ethyl acetate:Hexane) to yield a yellow oil (550 mg, 20 %).

R_f = 0.3 (15 % Et_2O: Hexane)

^{1}H NMR (500 MHz, CDCl$_3$): δ 7.73 (2H, d, *J* = 8.2 Hz, *H^{11}*), 7.55 (2H, d, *J* = 8.0 Hz, *H^3*), 7.39 (2H, d, *J* = 8.0 Hz, *H^4*), 7.32 (2H, d, *J* = 8.0 Hz, *H^{12}*), 5.45 (1H, ddt, *J* = 16.8, 10.1, 6.6 Hz, *H^8*), 5.06 (1H, d, *J* = 14.3 Hz, *H^{9b}*), 4.99 (1H, d, *J* = 16.7 Hz, *H^{9a}*), 4.37 (2H, s, *H^6*), 3.76 (2H, d, *J* = 6.6 Hz, *H^7*), 2.43 (3H, s, *H^{14}*).

^{13}C NMR (151 MHz, CDCl$_3$): δ 143.7 (1C, *C^{13}*), 140.6 (1C, *C^5*), 137.1 (1C, *C^{10}*), 132.0 (1C, *C^8*), 130.0 (1C, q, *J* = 31.9 Hz, *C^2*), 129.9 (2C, *C^{12}*), 128.6 (2C, *C^4*), 127.2 (2C,

C^{11}), 125.6 (2C, q, *J* = *3.8* Hz, C^3), 124.1 (1C, q, *J* = 272.0 Hz, C^1), 119.9 (1C, C^9), 50.3 (1C, C^7), 49.9 (1C, C^6), 21.6 (1C, C^{14}).

19F NMR (376 MHz, CDCl3): δ -62.37 (3F, s, F^1)

HRMS (ESI) calc: [M-H$^+$] ($C_{18}H_{18}F_3NO_2S$) 370.1083; measured: 370.1082 = 0.28 ppm difference

IR (neat) V$_{max}$/ cm^{-1} = 1321, 1150, 1108, 908, 662, 545

N-Allyl-N-(4-chlorobenzyl)-4-methylbenzenesulfonamide, 2s

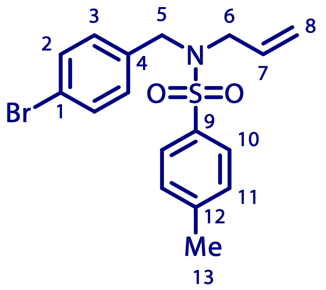

This substrate, **2r**, was synthesised from 4-bromobenzylamine (43 mmol) using **allylation procedure 3 a**nd was purified by silica-gel flash column chromatography (20 % Et_2O:Hexane) to yield a yellow oil (11 g, 68 %)

R*f* = 0.4 (20 % Et_2O: Hexane)

^{1}H NMR (400 MHz, CDCl$_3$): δ = 7.73 (2H, d, *J* = 8.5 Hz, H^{10}), 7.43 (2H, d, *J* = 8.2 Hz, H^2), 7.32 (2H, d, *J* = 8.5 Hz, H^{11}), 7.14 (2H, d, *J* = 8.2 Hz, H^3), 5.44 (1H, ddt, *J* = 16.8, 10.2, 6.6 Hz, H^7), 5.04 (1H dq, *J* = 10.1, 1.2 Hz, H^8), 5.01 (1H, dq, *J* = 17.0, 1.4 Hz, H^8), 4.27 (2H, s, H^5), 3.74 (2H, dd, *J* = 6.6, 1.4 Hz, H^6), 2.44 (3H, s, H^{13}).

^{13}C NMR (101 MHz, CDCl$_3$): δ = 143.6 (1C, C^{12}), 137.5 (1C, C^4), 135.3 (1C, C^9), 132.2 (1C, C^7), 131.8 (2C, C^2), 130.2 (2C, C^3) 129.9 (2C, C^{10}), 127.3 (2C, C^{11}), 121.8 (1C, C^1) 119.7 (1C, C^8), 49.9 (1C, C^6), 49.8 (1C, C^5), 21.7 (1C, C^{13}).

HRMS (ESI) calc: [M+H]$^+$ ($C_{17}H_{18}BrNO_2S$) 380.0314; measured: 380.0316 = 0.52 ppm difference

IR (neat) ν$_{max}$/ cm^{-1}: 2921, 2852, 1347, 1159, 1088, 1011, 894, 808, 665

N-allyl-N-cyclopropyl-4-methylbenzenesulfonamide, 2t

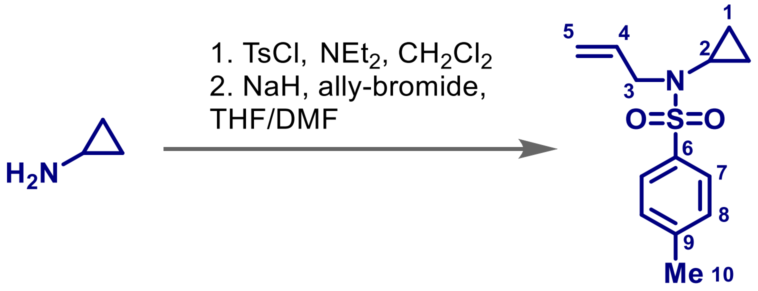

Under a nitrogen atmosphere, cyclopropylamine (55.0 mmol, 3.81 mL) and CH_2Cl_2 were added to a two-neck round bottom flask and cooled to 0 °C. Triethylamine (55.0 mmol, 7.67 mL) was added and then *p*-toluenesulfonyl chloride (50.0 mmol, 9.53 g) portion wise. After stirring overnight, the reaction was quenched with water, and extracted into CH_2Cl_2. The organic extract was washed with brine, dried with $MgSO_4$ and dried *en vacuo* to produce white crystalline solid (10.54 g, 99 % yield). The crude product was used directly without further purification.

Under a nitrogen atmosphere, sodium hydride (60 % in mineral oil, 2.2 mmol, 88 mg), THF (6 mL) and DMF (8 mL) were added to a two-neck round bottom flask. To this, a solution of *N*-cyclopropyl-4-methylbenzenesulfonamide (2.2 mmol, 464 mg) in THF (6 mL) was added dropwise. After stirring for 1 hour, allyl bromide (2.0 mmol, 172 µL) was added dropwise and the reaction mixture was stirred at 80 °C for 15.5 hrs. After the reaction mixture cooled to rt, it was quenched with 2 M HCl. The crude product was extracted with Et_2O, and the combined organic layers were then washed with brine, dried over $MgSO_4$ and dried *en vacuo* to produce a yellow oil. Purification by silica gel column chromatography (10 % EtOAc:Hexane) gave **2s** as an off-white solid (556 mg, 90 % yield).

^{1}H NMR (400 MHz, $CDCl_3$) δ = 7.75 (2H, d, *J* = 8.2 Hz, H^7), 7.31 (2H, d, *J* = 8.2 Hz, H^8), 5.73 (1H, ddt, J= 16.7, 10.1, 6.4 Hz, H^4), 5.24 – 5.07 (2H, m, H^5), 3.84 (2H, dt, *J* = 6.5, 1.3 Hz, H^3), 2.44 (3H, s, H^{10}), 2.03 (1H, tt, *J* = 6.9, 3.3 H^2), 0.88 – 0.81 (2H, m, H^1), 0.69 – 0.63 (2H, m, H^1).

^{13}C NMR (101 MHz, CDCl$_3$) δ_C = 143.5 (1C, *C*^{9}), 136.0 (1C, *C*^{4}), 133.5 (1C, *C*^{6}), 129.6 (2C, *C*^{7}), 127.9 (2C, *C*^{8}), 118.5 (1C, *C*^{5}), 53.8 (1C, *C*^{3}), 30.2 (1C, *C*^{2}), 21.7 (1C, *C*^{1}), 7.6 (1C, *C*^{1}).

HRMS (ESI) calc: [M+H]$^+$ ($C_{13}H_{18}NO_2S$) 252.1058; measured: 252.1061 = 0.78 ppm difference

IR (neat) ν_{max}/ cm^{-1}: 1650, 1485, 1357, 1156, 1081, 1016

1-(4-Allylphenyl)-N,N-dimethylmethanamine, 2v

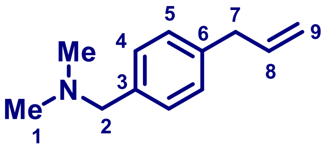

This substrate was synthesised according to the **Grignard procedure** using 1-(4-Bromophenyl)-*N,N*-dimethylmethanamine (10 mmol) and purified by silica gel flash column chromatography (10% IPA:CH_2Cl_2) to yield a colourless oil (400 mg, 33 %).

R*f* = 0.15 (30% Ethyl acetate in hexane)

^{1}H NMR (400 MHz, CDCl$_3$): δ = 7.25 – 7.21 (2H, m, *H*^{4}), 7.16 – 7.13 (2H, m, *H*^{5}), 6.03 – 5.92 (1H, m, *H*^{8}), 5.11 – 5.04 (2H, m, *H*^{9}), 3.43 – 3.36 (4H, m, *H*^{2,7}), 2.23 (6H, s, *H*^{1})

^{13}C NMR (101 MHz, CDCl$_3$): δ = 138.9 (1C, *C*^{6}), 137.6 (1C, *C*^{8}), 136.7 (1C, *C*^{3}), 129.3 (2C, *C*^{4}), 128.6 (2C, *C*^{5}), 115.8 (1C, *C*^{8}), 64.2 (1C, *C*^{2}), 45.5 (2C, *C*^{1}), 40.0 (1C, *C*^{7}).

IR (neat) ν_{max}/ cm^{-1}: 2976, 2941, 2813, 2765, 1638, 1512, 1455, 1432, 1362, 1019, 912, 856, 797

HRMS (ESI): calc [M+H$^+$] ($C_{11}H_{15}N$) 176.1439; measured: 176.1442 = 0.71 ppm difference

4-Allyl-*N,N*-dimethylaniline, 2w

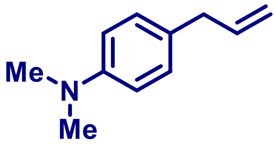

This substrate was synthesised according to the **Grignard procedure**[1] using 4-Bromo-N,N-dimethylaniline (15 mmol) and purified by silica gel flash column chromatography (5% EtOAc:Hexane) to yield a colourless oil (1.41 g, 58 %).

R_f = 0.4 (5% EtOAc:Hexane)

^{1}H NMR (400 MHz,CDCl$_3$): δ = 7.09 (2H, d, *J* = 8.4 Hz), 6.73 (2H, d, *J* = 8.6 Hz), 5.98 (1H, ddt, *J* = 16.8, 10.1, 6.7 Hz), 5.12 – 4.96 (2H, m), 3.32 (2H, d, *J* = 6.8 Hz), 2.93 (6H, s).

^{13}C NMR (101 MHz, CDCl$_3$): δ = 149.4, 138.5, 129.3, 128.3, 115.1, 113.2, 77.2, 41.1, 39.4.

These data are consistent with that previously reported.[5]

2-Allyl-1,3,5-trimethylbenzene, 2x

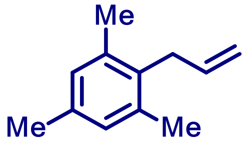

This substrate was synthesised according to the **Grignard procedure**[1] using 2-bromomesitylene (15 mmol) and purified by silica gel flash column chromatography (100% Hexane) to yield a colourless oil (1.35 g, 56 %).

R_f = 0.83 (Hexane)

^{1}H NMR (400 MHz, CDCl$_3$): δ = 6.98 – 6.87 (2H, m), 6.10 – 5.88 (1H, m), 5.20 – 4.92 (2H, m), 3.49 (2H, m), 2.39 (9H, m).

^{13}C NMR (101 MHz, CDCl$_3$): δ = 136.6, 135.6, 135.4, 133., 128.91, 114.8, 33.4, 21.0, 19.8.

These data are consistent with that previously reported.[5]

1-morpholinobut-3-en-1-one, 2z

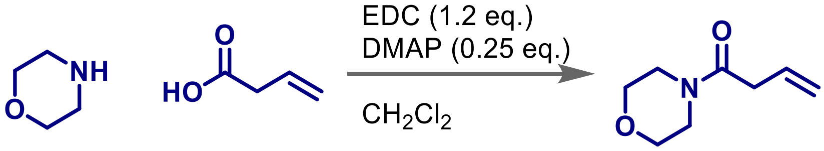

Under an inert atmosphere, but-3-enoic acid (1 eq., 15 mmol, 1.17 mL) and morpholine (1 eq., 15 mmol, 1.31 mL) were dissolved in CH_2Cl_2 (40 mL) and then EDC (1.1 eq., 16.5 mmol, 3.15 g) and DMAP (0.25 eq., 3.75 mmol, 0.42 g) were added. The solution was stirred overnight and then the reaction mixture was diluted with DCM (10 mL) and washed with H_2O (2 x 20 mL), $NaHCO_3$ (2 x 20 mL) and brine (10 mL). The organic layer was collected and dried using $MgSO_4$. After being dried *en vacuo* the crude mixture was purified using silica gel chromatography (10% MeOH:DCM) to yield a pale yellow oil (1.80 g, 77 %).

R_f = 0.5 (10% MeOH:DCM)

^{1}H NMR (400 MHz, $CDCl_3$) δ = 6.03 – 5.85 (1H, m), 5.22 – 5.11 (2H, m), 3.74 – 3.35 (8H, m), 3.15 (2H, dt, *J* = 6.5, 1.6 Hz).

^{13}C NMR (101 MHz, $CDCl_3$) δ = 169.7, 131.3, 118.1, 67.0, 66.7, 46.3, 42.1, 38.7

These data are consistent with that previously reported.[6]

N-allyl-N-(4-fluorophenyl)-4-methylbenzenesulfonamide, 2aa

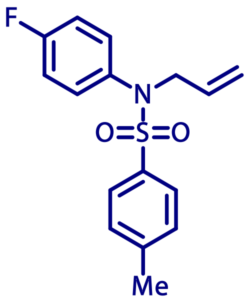

This substrate was synthesised from 4-fluoroaniline (5 mmol) and p-toluenesulfonyl chloride (instead of nitrobenzenesulfonyl chloride) using **allylation procedure 4** and was isolated by silica gel column chromatography (25% EtOAc:Hexane) to yield fluffy, colourless crystals (1.1 g, 70 %).

R_f = 0.4 (25% EtOAc:Hexane)

^{1}H NMR (500 MHz, $CDCl_3$): δ = 7.49 (2H, d, *J* = 8.6 Hz), 7.26 (2H, d, *J* = 8.6 Hz), 7.02 – 6.94 (4H, m), 5.72 (1H, ddt, *J* = 17.3, 9.9, 6.3 Hz), 5.01 – 5.13 (2H, m), 4.14 (2H, dt, *J* = 6.4, 1.3 Hz).

^{13}C NMR (151 MHz, $CDCl_3$): δ = 161.7 (d, *J* = 248.0 Hz), 143.6, 134.9, 134.9 (d, *J* = 3.1 Hz), 132.3, 130.5 (d, *J* = 8.7 Hz), 129.9, 127.1, 118.9, 115.8 (d, *J* = 22.6 Hz), 53.8, 21.5.

^{19}F NMR (376 MHz, $CDCl_3$): δ = -113.15 - -113.22 (1F, m, *F^1*)

These data are consistent with that previously reported.[7]

N-allyl-N-(4-fluorophenyl)-4-nitrobenzenesulfonamide, 2ab

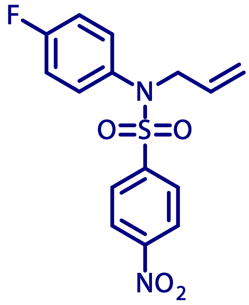

This substrate was synthesised from 4-fluoroaniline (5 mmol) using **allylation procedure 4** and was purified by silica gel column chromatography (30% EtOAc:Hexane) to yield colourless crystals (900 mg, 54 %).

R_f = 0.2 (30% EtOAc:Hexane)

^{1}H NMR (500 MHz, $CDCl_3$): δ = 8.33 (2H, d, *J* = 9.0 Hz), 7.80 (2H, d, *J* = 9.1 Hz), 7.04 – 6.97 (4H, m), 5.75 (1H, ddt, *J* = 16.7, 10.3, 6.4 Hz), 5.21 – 5.07 (2H, m), 4.26 (2H, dt, *J* = 6.4, 1.3 Hz)

^{13}C NMR (151 MHz, $CDCl_3$): δ = 162.2 (d, 247.9 Hz), 150.3, 144.3, 134.1 (d, *J* = 3.4 Hz), 132.0, 130.9 (d, *J* = 8.9 Hz), 128.9, 124.3, 120.1, 116.4 (d, *J* = 22.7 Hz), 54.4.

^{19}F NMR (376 MHz, $CDCl_3$): δ = -111.70 - -111.77 (m)

These data are consistent with that previously reported.[8]

N-allyl-N-(4-fluoro-2-methylphenyl)-4-nitrobenzenesulfonamide, 2ac

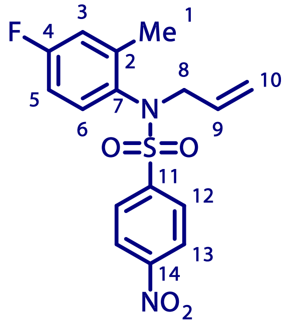

This substrate was synthesised from N-(4-fluoro-2-methylphenyl)-4-nitrobenzenesulfonamide (10 mmol) using **allylation procedure 4** and was purified by silica gel column chromatography (30% EtOAc:Hexane) to yield a colourless powder (0.87 g, 35 %).

R_f = 0.4 (30% EtOAc:Hexane)

^{1}H NMR (400 MHz, $CDCl_3$): δ = 8.38 – 8.32 (2H, m, H^{12}), 7.90 – 7.86 (2H, m, H^{13}), 6.99 (1H, dd, J = 9.1, 3.0 Hz, H^3), 6.76 (1H, td, J = 8.2, 3.0 Hz, H^5), 6.51 (1H, dd, J = 8.7, 5.2 Hz, H^6), 5.73 (1H, ddt, J = 17.0, 10.1, 6.9 Hz, H^9), 5.08 (1H, dd, J = 10.0, 1.1 Hz, H^{10}), 5.03 (1H, dq, J = 16.9, 1.2 Hz, H^{10}), 4.29 (1H, ddd, J = 15.3, 6.4 Hz, H^8), 3.96 (1H, ddd, J = 14.1, 7.1 Hz, H^8), 2.33 (3H, s, H^1).

^{13}C NMR (101 MHz, $CDCl_3$): δ = 162.2 (1C, d, J = 249.2 Hz, C^4), 150.3 (1C, s, C^{14}), 144.9 (1C, C^{11}), 142.7 (d, J = 8.7 Hz, C^2), 133.0 (1C, d, J = 3.0 Hz, C^7), 131.5 (1C, C^9), 130.1 (1C, d, J = 9.2 Hz C^6), 129.1 (2C, C^{13}), 124.4 (2C, C^{12}), 120.6 (1C, C^{10}), 118.4 (1C, d, J = 22.1 Hz, C^3), 113.6 (d, J = 22.6 Hz, C^5), 55.2 (1C, C^8), 18.9 (1C, s, C^1)

^{19}F NMR (376 MHz, $CDCl_3$): δ -111.79– -111.91 (1F, m, F^1)

HRMS (APCI) calc: [M+H$^+$] ($C_{16}H_{15}N_2O_4FS$) 351.0809; measured: 351.0807 = 0.57 ppm difference

IR (neat) V_{max}/ cm^{-1} = 2924, 2856, 1526, 1495, 1349, 1167, 1262, 1089, 852, 738

N-allyl-N-(4-iodophenyl)-4-nitrobenzenesulfonamide, 2ad

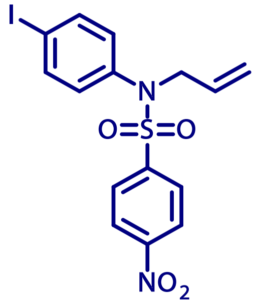

This substrate was synthesised from 4-iodoaniline (5 mmol) using **allylation procedure 4** was purified by silica gel column chromatography (30% EtOAc:Hexane) to yield a brown-orange solid (0.9 g, 41 %).

R$_f$ = 0.2 (30% EtOAc:Hexane)

^{1}H NMR (500 MHz, CDCl$_3$): δ = 8.33 (2H, d, *J* = 8.6 Hz), 7.81 (2H, d, *J* = 8.6 Hz), 7.65 (2H, d, *J* = 8.1 Hz), 6.78 (2H, d, *J* = 8.1 Hz), 5.71 (1H, ddt, *J* = 16.8, 10.3, 6.4 Hz), 5.20 – 5.00 (2H, m), 4.21 (2H, dt, *J* = 6.4, 1.3 Hz)

^{13}C NMR (151 MHz, CDCl$_3$): δ = 150.3, 144.1, 138.7, 138.1, 131.8, 130.7, 128.9, 124.4, 120.2, 94.2, 54.0.

These data are consistent with that previously reported.[8]

N-allyl-N-(2-bromophenyl)-4-nitrobenzenesulfonamide, 2ae

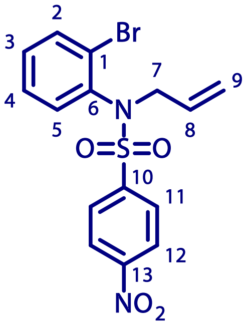

This substrate was synthesised from 2-bromoaniline (5 mmol) using **allylation procedure 4** and was purified by silica gel column chromatography (30% EtOAc:Hexane) to yield a brown-orange solid (1.5 g, 76 %).

R$_f$ = 0.2 (30% EtOAc:Hexane)

^{1}H NMR (500 MHz, CDCl$_3$): δ = 8.33 (2H, d, 8.9 Hz, *H^{12}*), 7.93 (2H, d, *J* = 9.0 Hz, *H^{11}*), 7.60 – 7.57 (1H, m, *H^2*), 7.35 – 7.30 (1H, m, *H^5*), 7.26 – 7.20 (2H, m, *$H^{3,4}$*), 5.84 (1H, ddt, *J* = 16.9, 10.1, 6.7 Hz, *H^8*), 5.14 – 5.03 (2H, m, *$H^{9a,b}$*), 4.47 – 4.39 (1H, m, , *H^{7a}*), 4.13 – 4.04 (1H, m, *H^{7b}*).

^{13}C NMR (151 MHz, CDCl$_3$): δ = 150.2 (1C, *C^{13}*), 145.9 (1C, *C^{103}*), 136.6 (1C, *C^6*), 134.2 (1C, *C^4*), 133.6 (1C, *C^3*), 132.1 (1C, *C^8*), 130.6 (1C, *C^2*), 129.1 (2C, *C^{12}*), 128.3 (1C, *C^5*), 124.9 (1C, *C^1*), 124.3 (2C, *C^{11}*), 120.3 (1C, *C^9*), 54.2 (1C, *C^7*).

IR (neat) ν_{max}/ cm^{-1}: 1526, 1471, 1352, 1154, 1087, 1044, 939, 853, 736, 602

HRMS (ESI$^+$) calc: [M+H$^+$] ($C_{15}H_{14}BrN_2O_2S$) 395.9779; measured: 395.9781 = 0.8 ppm difference.

N-allyl-N-mesityl-4-nitrobenzenesulfonamide, 2af

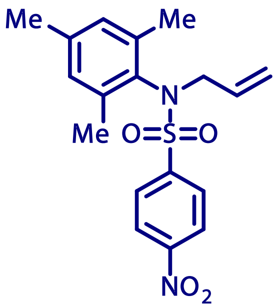

This substrate was synthesised from mesityl aniline (10 mmol) using **allylation procedure 4** yield an off-white solid (2.13 g, 59 %).

R$_f$ = 0.6 (20% EtOAc:Hexane)

^{1}H NMR (400 MHz, CDCl$_3$) δ = 8.33 (2H, d, *J* = 9.0 Hz), 7.96 (2H, d, *J* = 9.1 Hz), 6.85 (2H, s), 5.86 (1H, ddt, *J* = 17.7, 9.3, 7.0 Hz), 5.10 – 5.03 (2H, m), 4.12 (2H, d, *J* = 6.9 Hz), 2.25 (6H, s), 1.97 (1H, s).

^{13}C NMR (101 MHz, CDCl$_3$): δ = 150.0, 146.9, 138.7, 138.5, 133.5, 132.5, 130.1, 128.7, 124.4, 120.0, 54.6, 21.1, 19.2.

These data are consistent with that previously reported.[8]

N-(4-fluorophenyl)-N-(2-methylallyl)-4-nitrobenzenesulfonamide, 2ag

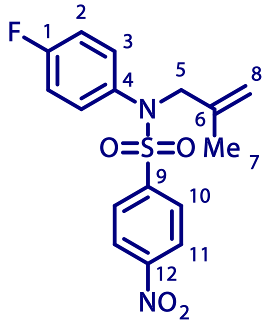

This substrate was synthesised from N-(4-fluorophenyl)-4-nitrobenzenesulfonamide (3 mmol) using **allylation procedure 4** to yield a brown solid (583 mg, 56%).

R_f = 0.6 (20% EtOAc:Hexane)

^{1}H NMR (400 MHz, $CDCl_3$): δ = 8.32 (2H, d, *J* = 9.0 Hz, H^{11}), 7.76 (2H, d, *J* = 9.0 Hz, H^{10}), 7.03 – 6.98 (4H, m, $H^{2,3}$), 4.75 (2H, d, *J* = 40.3 Hz, H^{8}), 4.11 (2H, s, H^{5}), 1.76 (3H, s, H^{7}).

^{13}C NMR (101 MHz, $CDCl_3$): δ = 162.1 (1C, d, *J* = 249.3 Hz, C^{1}), 150.3 (1C, C^{12}), 143.9 (1C, $C^{9,}$), 138.9 (1C, C^{6}), 133.9 (1C, d, *J* = 3.2 Hz, C^{4}), 130.4 (2C, d, *J* = 8.7 Hz, C^{3}), 128.9 (2C, C^{11}), 124.3 (2C, C^{10}), 116.3 (1C, C^{8}), 116.3 (2C, d, *J* = 22.8 Hz, C^{2}), 57.6 (1C, C^{5}), 19.9 (1C, C^{7}).

^{19}F NMR (376 MHz, $CDCl_3$): δ = -122.02 – 122.11 (1F, m, F^{1}).

HRMS (APCI) calc: [M+H^{+}] ($C_{16}H_{15}FN_2O_4S$) 351.0809; measured: 351.0796, = 3.70 ppm difference.

IR (neat) v_{max}/ cm^{-1}: 1606, 1530, 1505, 1351, 1169, 1092, 854, 737, 686, 606

1-(2-methylprop-1-en-1-yl)-4-nitrobenzene, 2aj

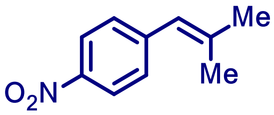

This substrate was prepared from 4-nitrobenzaldehyde (5 mmol) using the witting procedure and was purified by silica gel column chromatography (10% EtOAc:Hexane) to yield a pale yellow oil (560 mg, 78 %).

R_f = 0.3 (10% EtOAc:Hexane)

^{1}H NMR (500 MHz, CDCl$_3$): δ = 8.17 (2H, d, *J* = 8.8 Hz), 7.35 (2H, d, *J* = 8.8 Hz), 6.31 (1H, s), 1.96 (3H, dd, *J* = 1.4 Hz,), 1.91 (3H, dd, *J* = 1.4 Hz)

^{13}C NMR (151 MHz, CDCl$_3$): δ = 145.6, 140.3, 133.9, 133.8, 129.4, 123.8, 27.4, 19.9.

These data are consistent with that previously reported.[9]

1-fluoro-4-(2-methylprop-1-en-1-yl)benzene, 2ak

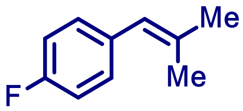

This substrate was synthesised from 4-fluorobenzaldehyde (5 mmol) using the witting procedure and was purified by silica gel flash column chromatography (100% Hexane) to afford a volatile, colourless oil (300 mg, 60 %).

R_f = 0.8 (Hexane)

^{1}H NMR (400 MHz, CDCl$_3$): δ = 7.20 – 7.14 (2H, m), 7.02 – 6.96 (2H, m), 6.22 (1H, s), 1.89 (3H, s), 1.83 (3H, s).

^{13}C NMR (101 MHz, CDCl$_3$): δ = 162.5 (d, *J* = 249.3 Hz), 140.3 (s), 135.6 (d, *J* = 8.3Hz), 130.3 (d, *J* = 2.6 Hz), 125.3 (s), 116.3 (d, *J* = 22.8 Hz), 26.9 (s), 19.4 (s).

^{19}F NMR (376 MHz, CDCl$_3$): δ = -116.82 - -116.91 (m).

These data are consistent with that previously reported.[13]

1-(2-methylprop-1-en-1-yl)-3,5-bis(trifluoromethyl)benzene, 2al

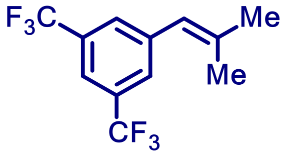

This substrate was synthesised from 3,5 bis(trifluoromethylbenzaldehyde) (5 mmol) using the witting procedure and and was purified by silica gel flash column chromatography (10% EtOAc:Hexane) to afford a colourless oil (575 mg, 43 %).

R*f* = 0.6 (10% EtOAc:Hexane)

^{1}H NMR (400 MHz, CDCl$_3$): δ = 7.68 (1H, s), 7.63 (2H, s), 6.30 (1H, s), 1.95 (3H, s), 1.87 (3H, s).

^{13}C NMR (101 MHz, CDCl$_3$): δ = 140.7, 139.8, 131.4 (q, *J* = 33.2 Hz), 128.8, 123.7 (q, *J* = 275.2 Hz), 122.9, 119.6, 27.0, 19.5.

^{19}F NMR (376 MHz, CDCl$_3$): -62.83 (s).

These data are consistent with that previously reported.[11]

N-allyl-4-methyl-N-(4-methylbenzyl)benzenesulfonamide, 2am

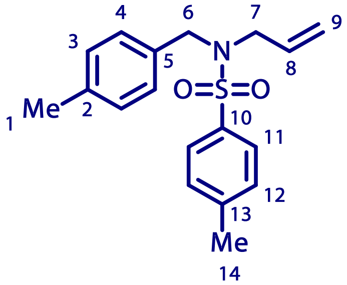

This substrate was synthesised from 4-methylbenzylamine (5 mmol) using **allylation procedure 3** and was purified by silica gel column chromatography (10% EtOAc:Hexane) yield an off-white solid (2.1 g, 65 %).

R$_f$ = 0.3 (10% EtOAc:Hexane)

^{1}H NMR (500 MHz, CDCl$_3$): δ = 7.74 (2H, d, J = 8.3 Hz, H^{11}), 7.31 (2H, d, J = 8.4 Hz, H^{12}), 7.15 – 7.08 (4H, m, $H^{3,4}$), 5.53 – 5.41 (1H, m, H^{8}), 5.08 – 4.97 (2H, m, H^{9}), 4.30 (2H, s, H^{6}), 3.74 (2H, d, 6.3 Hz, H^{6}), 2.44 (3H, s, C$\underline{H_3}$), 2.33 (3H, s, C$\underline{H_3}$).

^{13}C NMR (151 MHz, CDCl$_3$): δ = 143.3 (1C, C^{13}), 137.8 (1C, C^{5}), 137.6 (1C, C^{13}), 133.0 (1C, C^{10}), 132.4 (1C, C^{8}), 129.8 (2C, C^{11}), 129.3 (2C, $\underline{C_{Ar}}H$), 128.6 (2C, $\underline{C_{Ar}}H$), 127.4 (2C, C^{12}), 119.4 (1C, C^{9}), 50.0 (1C, C^{6}), 49.4 (1C, C^{7}), 21.7 (1C, C^{1}), 21.3 (1C, C^{14}).

IR (film) ν_{max}/ cm^{-1}: 1597, 1451, 1340, 1160, 1090, 1017, 814

HRMS (ESI$^+$) calc: [M+Na]$^+$ ($C_{18}H_{21}NO_2SNa$) 338.1191; measured: 338.1183 = 2.14 ppm difference.

N-(4-chlorobenzyl)-4-methyl-N-(2-methylallyl)benzenesulfonamide, 2an

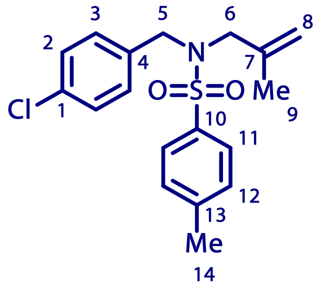

This substrate was synthesised from N-(4-chlorobenzyl)-4-methylbenzenesulfonamide using the **Mitsunobu procedure** and purified using silica gel chromatography (20% EtOAc:Hexane) to yield a viscous, colourless oil (541 mg, 46 %).

R_f = 0.6 (20% EtOAc:Hexane)

^{1}H NMR (400 MHz, CDCl$_3$): δ = 7.70 (2H, d, J = 8.3 Hz, H^{11}), 7.30 (2H, d, J = 7.9 Hz, H^{12}), 7.23 (2H, d, J = 8.5 Hz, H^{2}), 7.13 (2H, d, J = 8.6 Hz, H^{3}), 4.75 (2H, d, J = 48.6 Hz, H^{8}), 4.27 (2H, s, H^{5}), 3.67 (2H, s, H^{6}), 2.44 (3H, s, H^{14}), 1.51 (3H, s, H^{9}).

^{13}C NMR (101 MHz, CDCl$_3$): δ = 143.5 (1C, C^{13}), 140.1 (1C, C^{8}), 137.4 (1C, C^{1}), 134.9 (1C, C^{4}), 133.6 (1C, C^{10}), 130.2 (2C, C^{3}), 129.8 (2C, C^{11}), 128.6 (2C, C^{2}), 127.4 (2C, C^{12}), 115.2 (1C, C^{8}), 53.9 (1C, C^{5}), 50.3 (1C, C^{6}) , 21.7 (1C, C^{14}), 20.0 (1C,C^{9}).

HRMS (EI+) calc: [M+•] ($C_{18}H_{20}ClNO_2S$) 349.0903; measured: 349.0917 = 3.17 ppm difference.

IR (neat) ν_{max}/ cm^{-1}: 1598, 1491, 1443, 1339, 1159, 1093, 1014, 911, 814, 773, 694

N-(but-2-en-1-yl)-N-(4-chlorobenzyl)-4-methylbenzenesulfonamide, 2ao

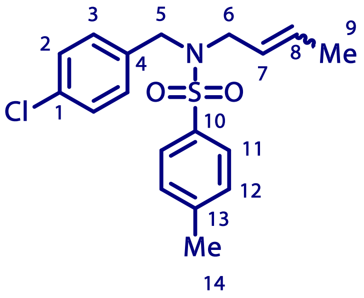

This substrate. **2ai**, was synthesised from crotyl alcohol (10 mmol) using **the Mitsunobu procedure** and was purified by silica gel column chromatography (30% EtOAc:Hexane) to yield a colourless oil (1.25 g, 65 %). Cis:trans = 1.6:1

R_f = 0.4 (30% EtOAc:Hexane)

^{1}H NMR (500 MHz, CDCl$_3$): δ = 7.72 (2H, d, *J* = 8.4 Hz, H^{12}), 7.33 – 7.25 (4H, m, $H^{2,3}$), 7.20 (2H, d, H^{11}), 5.44 – 5.36 (1H, m, H^{8}), 5.11 – 5.04 (1H, m, H^{7}), 4.27 (2H, s, H^{5}), 3.67 (2H, d, *J* = 6.7 Hz, H^{6}), 2.45 (3H, s, H^{14}), 1.56 – 1.54 (3H, m, H^{9}).

^{13}C NMR (125 MHz, CDCl$_3$): δ = 143.4 (1C, C^{13}), 137.6 (1C, C^{1}), 135.1 (1C, C^{4}), 133.5 (1C, C^{10}), 131.3 (1C, C^{8}), 129.9 (2C, C^{3}), 129.8 (2C, C^{11}), 128.8 (2C, C^{2}), 127.4 (2C, C^{12}), 124.8 (1C, C^{7}), 49.6 (1C, C^{5}), 49.3 (1C, C^{6}), 21.7 (1C, C^{14}), 17.7 (1C, C^{9}).

HRMS (ESI) calc: [M+H+] ($C_{18}H_{20}ClNO_2S$) 350.0976; measured: 350.0969 = 1.99 ppm difference.

IR (neat) ν_{max}/ cm^{-1}: 1597, 1491, 1337, 1158, 1090, 1050, 915, 814

N-(4-chlorobenzyl)-4-methyl-N-(3-methylbut-2-en-1-yl)benzenesulfonamide, 2ap

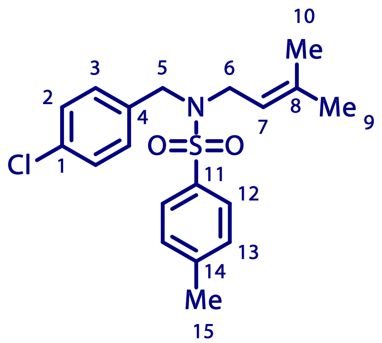

This substrate. **2aj**, was synthesised from prenyl alcohol (10 mmol) using **the Mitsunobu procedure** and was purified by silica gel column chromatography (20% EtOAc:Hexane) to yield a colourless oil (1.16 g, 60 %).

R_f = 0.4 (20% EtOAc:Hexane)

^{1}H NMR (500 MHz, $CDCl_3$): δ = 7.72 (2H, d, *J* = 8.4 Hz, H^{13}), 7.33 – 7.25 (4H, m, $H^{2,3}$), 7.20 (2H, d, H^{12}), 4.83 – 4.78 (1H, m, H^7), 4.26 (2H, s, H^5), 3.73 (2H, d, *J* = 7.5 Hz, H^6), 2.44 (3H, s, H^{15}), 1.55 (3H, s, CH_3), 1.38 (3H, s, CH_3).

^{13}C NMR (125 MHz, $CDCl_3$): 143.4 (1C, C^{14}), 137.6 (1C, C^1), 137.4 (1C, C^8), 135.3 (1C, C^4), 133.5 (1C, C^{11}), 129.8 (2C, C^{12}), 129.7 (2C, C^3), 128.7 (2C, C^2), 127.3 (2C, C^{13}), 118.3 (1C, C^7), 49.8 (1C, C^5), 44.9 (1C, C^6), 25.8 (1C, CH_3), 21.2 (1C, C^{15}), 17.8 (1C, CH_3),

HRMS (EI^+) calc: [$M+Na^+$] ($C_{19}H_{22}ClNO_2SNa$) 386.0957; measured: 386.0941 = 3.71 ppm difference

IR (neat) v_{max}/ cm^{-1}: 1597, 1491, 1339, 1158, 1091, 1014, 911, 814, 750

(Z)-N,N-dibenzylhex-3-en-1-amine, 2aq

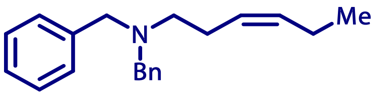

This substrate was synthesised from trans hex-3-en-1-ol (20 mmol) using **mesylation procedure** and was purified by silica gel column chromatography (5% Et_2O:Hexane) to yield a colourless oil (750 mg, 68 %).

R*f* = 0.4 (5% Et_2O:Hexane)

^{1}H NMR (500 MHz, $CDCl_3$): δ = 7.40 – 7.20 (10H, m), 5.41 – 5.35 (1H, m), 5.32 – 5.25 (1H, m), 3.59 (4H, s), 2.47 – 2.25 (2H, t, *J* = 7.4 Hz), 2.49 – 2.45 (2H, m), 2.03 – 1.96 (2H, m), 0.91 (3H, t, *J* = 8.7 Hz).

^{13}C NMR (126 MHz, $CDCl_3$): δ = 140.0, 132.6, 128.9, 128.3, 127.0, 126.9, 58.3, 53.4, 25.0, 20.7, 14.5.

These data are consistent with that previously reported. [12]

(E)-N,N-dibenzylhex-3-en-1-amine, 2ar

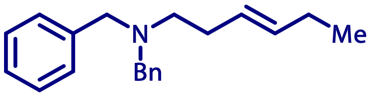

This substrate was synthesised from trans hex-3-en-1-ol (20 mmol) using **mesylation procedure** and was purified by silica gel column chromatography (5% Et_2O:Hexane) to yield a colourless oil (650 mg, 50 %).

R*f* = 0.4 (5% Et_2O:Hexane)

^{1}H NMR (500 MHz, $CDCl_3$): δ = 7.41 – 7.19 (10H, m), 5.51 – 5.41 (1H, m), 5.39 – 5.29 (1H, m), 3.58 (4H, s), 2.47 – 2.25 (2H, t, *J* = 7.4 Hz), 2.25 – 2.18 (2H, m), 2.04 – 1.94 (2H, m), 0.96 (3H, t, *J* = 8.7 Hz).

^{13}C NMR (126 MHz, $CDCl_3$): δ = 140.1, 133.1, 128.9, 128.2, 127.3, 126.9, 58.3, 53.6, 30.5, 25.8, 14.0.

These data are consistent with that previously reported. [12]

1-((Allyloxy)methyl)-2-methylbenzene, 2as

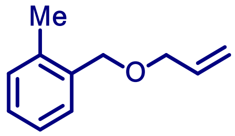

This substrate was synthesised from o-tolylmethanol (20 mmol) using **allylation procedure 1** and was purified by silica gel column chromatography (5% Et_2O:Hexane) to yield a colourless oil (810 mg, 25 %).

R*f* = 0.50 (10% Et_2O:Hexane)

^{1}H NMR (400 MHz, $CDCl_3$): δ = 7.34 (1H, dd, *J* = 6.8, 2.4 Hz), 7.25 – 7.14 (3H, m), 5.98 (1H, ddt, *J* = 17.3, 10.4, 5.6 Hz), 5.33 (1H, dq, *J* = 17.3, 1.7 Hz), 5.25 – 5.19 (1H, m), 4.53 (2H, s), 4.06 (2H, dt, *J* = 5.7, 1.5 Hz), 2.35 (3H, s).

^{13}C NMR (101 MHz, $CDCl_3$): δ = 136.8, 136.3, 135.0, 130.3, 128.6, 127.8, 125.9, 117.2, 71.4, 70.6, 18.9

These data are consistent with that previously reported.[13]

((2-Methylallyloxy)methyl)benzene, 2at

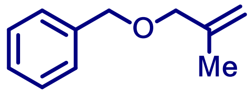

This substrate was synthesised from 2-methylprop-2-en-1-ol (10 mmol) and benzyl bromide (15 mmol) using **allylation procedure 1** to yield a colourless oil (426 mg, 26 %).

^{1}H NMR (400 MHz, $CDCl_3$): δ = 7.38 – 7.31 (2H, m), 7.30 – 7.23 (3H, m), 4.96 (2H, dq, *J* = 33.5, 1.3 Hz), 4.49 (2H, s), 3.97 – 3.91 (2H, m), 1.94 – 1.70 (3H, m)

^{13}C NMR (101 MHz, $CDCl_3$): δ = 142.3, 138.5, 128.4, 127.8, 127.6, 112.4, 74.2, 71.9, 19.6

These data are consistent with that previously reported.[14]

1-(((2-Methylallyl)oxy)methyl)-4-(trifluoromethyl)benzene, 2au

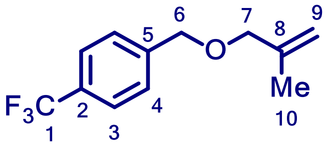

This substrate was synthesised from 2-methylprop-2-en-1-ol (10 mmol) and 1-(Bromomethyl)-4-(trifluoromethyl)benzene (15 mmol) **allylation procedure 2** to yield a colourless oil (1.63 mg, 71 %).

R*f* = 0.3 (20% EtOAc:Hexane)

^{1}H NMR (400 MHz, CDCl$_3$): δ = 7.61 (2H, d, *J* = 8.4 Hz, H^3), 7.47 (2H, d, *J* = 7.7, H^4), 5.03 – 4.93 (2H, m, H^{10}), 4.55 (2H, s, H^6), 3.96 (2H, s, H^7), 1.78 (3H, s, H^9)

^{13}C (101 MHz, CDCl$_3$): δ = 142.8 (1C, C^5), 142.0 (1C, C^8), 129.8 (1C, q, *J* = 32.3 Hz, C^2), 127.6 (2C, C^4), 125.4 (2C, C^3), 124.3 (1C, q, *J* = 274.2 Hz, C^1), 112.7 (1C, s, C^9), 74.6 (1C, C^6), 71.1 (1C, C^7), 19.6 (1C, C^{10}).

^{19}F NMR (376 MHz, CDCl$_3$): δ = -62.36 (3F, s, F^1)

IR (neat) v_{max}/ cm^{-1}: 2856, 1621, 1419, 1322, 1162, 1119, 1064, 1018, 901, 820, 722, 642.

HRMS (APCI) calc: [M-H$^+$] ($C_{12}H_{13}F_3O$) 231.0991; measured: 231.0983 = 3.46 ppm

Synthesis of iodoarene catalysts for benchmarking experiments

Methyl 3,5-dihydroxy-4-iodobenzoate

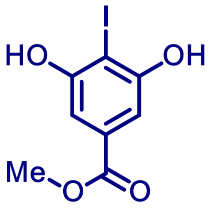

This substrate was synthesised according to a literature procedure[14] using methyl 3,5-dihydroxybenzoate (11.9 mmol) 1-(4-Bromophenyl)-*N,N*-dimethylmethanamine (10 mmol) to yield colourless crystals (3.5 g, 100 %).

^{1}H NMR (400 MHz, $(CD_3)_2SO$): δ = 10.52 (2H, s), 6.93 (2H, s), 3.80 (3H, s).

^{13}C NMR (101 MHz, $(CD_3)_2SO$): δ = 166.0, 158.3, 130.6, 105.8, 81.8, 52.2.

These data are consistent with that previously reported.[15]

Dimethyl2,2'-((2-iodo-5-(methoxycarbonyl)-1,3-phenylene)bis(oxy)) diacetate

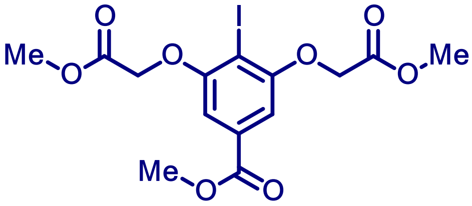

This catalyst was synthesised according to a literature procedure [11] to yield colourless crystals (782 g, 18 %).

^{1}H NMR (400 MHz, $CDCl_3$): δ = 7.07 (2H, s), 4.78 (4H, s), 3.91 (3H. s), 3.82 (6H, s)

^{13}C NMR (101 MHz, $CDCl_3$): δ = 168.5, 166.1, 158.4, 132.0, 106.6, 85.9, 66.4, 52.7, 52.6.

These data are consistent with that previously reported.[12]

Electrochemical setup

Undivided cell (general ‘in-cell’ procedures 1 and 2)

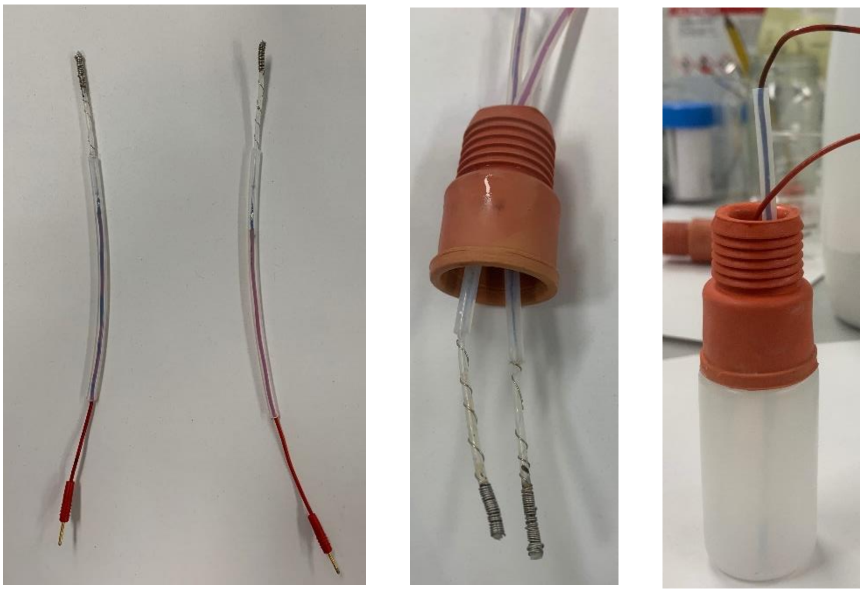

Figure S7: Undivided cell setup. 1. Platinum electrodes used for electrolysis.2. Platinum electrodes through a rubber suba-seal. 3. Platinum electrodes inserted onto a 20 mL HDPE vial.

Platinum electrodes for bulk electrolysis

Platinum electrodes were made by wrapping platinum wire around PTFE tubing to creat a surface area approximately ~1 cm^2. The platinum wire was then fed through PTFE tubing by creating a small hole on the side of the tubing. The wire was then spot-welded to a copper wire.

Reaction vessels

Commercially available scintillation vials and B-19 rubber suba-seals.

Cleaning of electrodes

After each reaction, the electrodes are left to soak in a saturated aqueous Ca_2CO_3 solution for 10 minutes. After this they were rinsed with water, washed with acetone then left to dry in a 70 ºC for several hours before use. Occasionally, there was an amorphous film that is resistant to cleaning, thus at this point the platinum wire is unwound and cleaned with a paper towel soaked in acetone. After re-winding the platinum wire around the PTFE tube, the electrodes are used to apply a potential difference of 10 V to a solution of CH_3CN and $^nBu_4NBF_4$ (0.2M) for 30 minutes. After

this the electrodes are re-cleaned with acetone and left to dry in a 70 ºC for several hours before use.

Divided cell (General 'ex-cell' procedure 3)

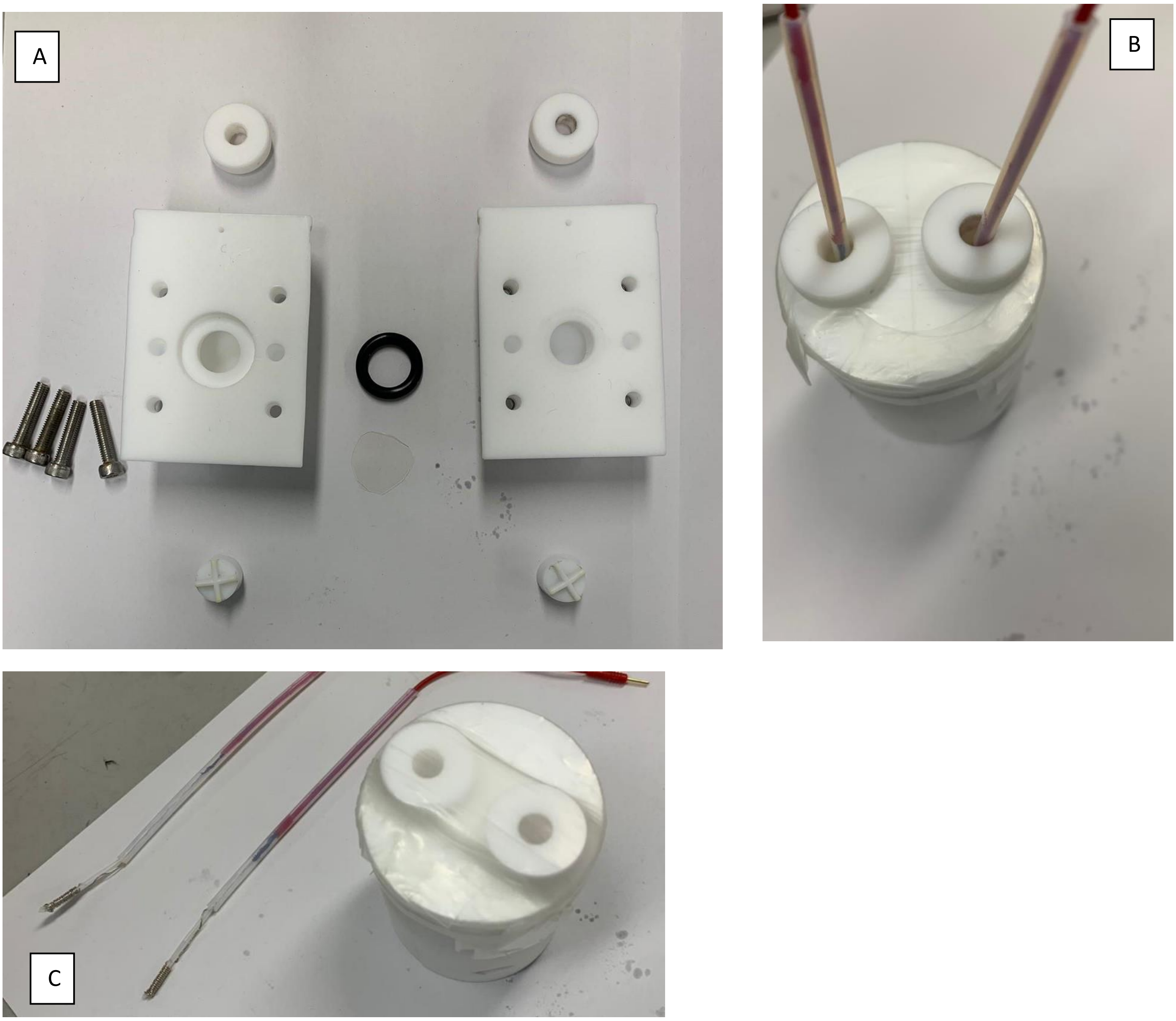

Figure S8: Photos of divided-cell setup: A. The cell taken apart. B. After assembly, the top panel is covered in parafilm and electrodes are placed through the holes on each of the compartments. C. After the electrolysis, the electrodes are removed and the vessels are sealed with parafilm. A more detailed schematic of this cell design that includes details of how to manufacture these cells has already been reported.[16]

Gram-scale reactor

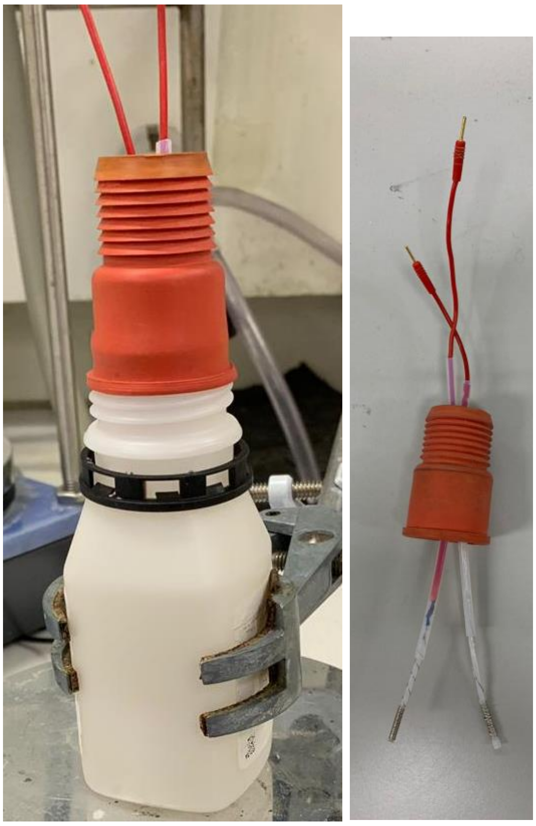

Figure S9: Gram-scale reactor. The platinum electrodes are inserted through a B24 suba-seal and pushed into an old Py.9HF 100 mL plastic bottle purchased from Sigma. No change in the electrode configuration was necessary.

Deca-gram reactor

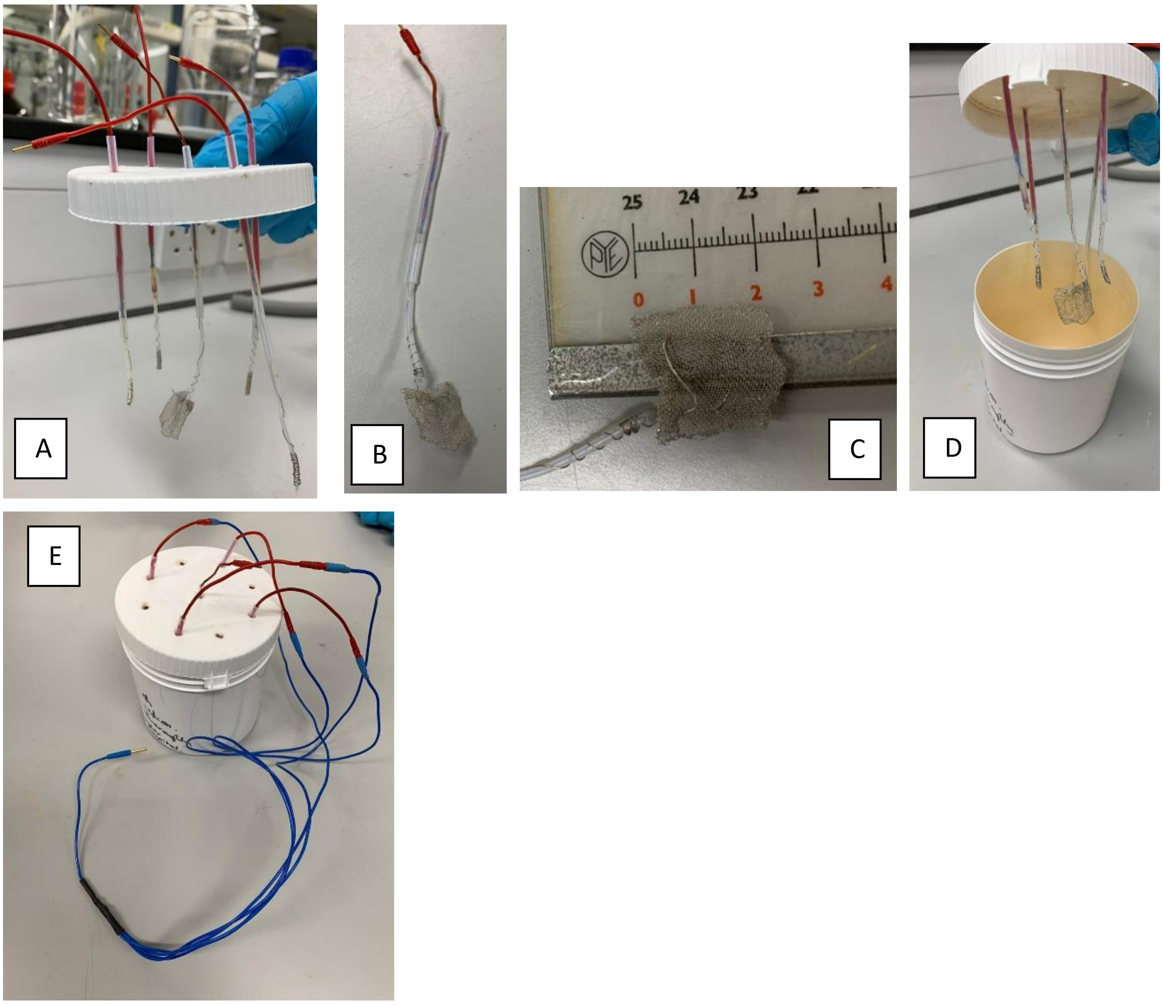

Figure S10: Large scale batch reactor. A) The platinum electrodes pushed through holes in the lid of the plastic pot. B) A platinum mesh electrode was assembled for the counter electrode. C) The size of the platinum mesh (2 cm x 2 cm). D) Assembly of reactor. E) A splitter was used for the 4 anodes.

General procedures for electrochemical 1,2 difluorination of alkenes

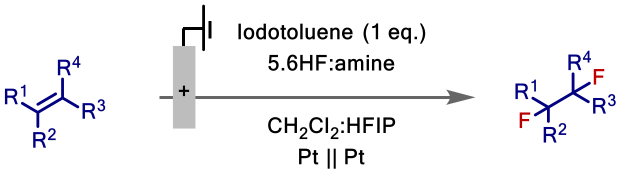

'In-cell' procedure 1

To a 20 mL HDPE vial, equipped with a magnetic stirrer bar, was added iodotoluene (1 eq., 1.2 mmol, 262 mg), CH_2Cl_2 (0.7 mL), HFIP (0.3 mL) and 5.6HF:amine stock solution (3 mL). Alkene substrate (1 eq., 1.2 mmol) was then in one portion either via pipette or spatula and the vial was capped with the suba-seal equipped with platinum anode and cathode. The reaction was then subject electrolysis (3.5 F/mol, 13 mA, 6 hrs). The electrodes were then removed, and the vial was capped with an HDPE cap and the reaction was stirred overnight. The reaction solution was then quenched with 50 mL of cold (0 ºC) saturated aqueous $CaCO_3$ solution. This stirred for 1 hour until the aqueous layer measured pH 7. The mixture was extracted into CH_2Cl_2, dried with Na_2SO_4 and evaporated *en vacuo*. The product was then isolated via column chromatography.

'In-cell' procedure 2

To a 20 mL HDPE vial, equipped with a magnetic stirrer bar, was added iodotoluene (1 eq., 0.8 mmol, 175 mg), CH_2Cl_2 (0.7 mL), HFIP (0.3 mL) and 5.6HF:amine stock solution (3 mL). Alkene substrate (1 eq., 0.8 mmol) was then in one portion either via pipette or spatula and the vial was capped with the suba-seal equipped with platinum anode and cathode. The reaction was then subject electrolysis (3.5 F/mol, 9 mA, 6 hrs). The electrodes were then removed, and the vial was capped with an HDPE cap and the reaction was stirred overnight. The reaction solution was then quenched with 50 mL of cold (0 ºC) saturated aqueous $CaCO_3$ solution. This stirred for 1 hour until the aqueous layer measured pH 7. The mixture was extracted into CH_2Cl_2, dried with Na_2SO_4 and evaporated *en vacuo*. The product was then isolated via column chromatography.

'Ex-cell' procedure 3

To each compartment of PTFE divided cell equipped with a nafion membrane and stirring bars, CH_2Cl_2 (1.05 mL), HFIP (0.45 mL) and 5.6 HF stock solution (4.5 mL) were added. To the anodic compartment, iodotoluene (1 eq., 1.2 mmol, 262 mg) was then added. Each compartment was then capped and wrapped in parafilm. A platinum electrode was inserted into each compartment, and the reaction was subjected to electrolysis (13.4 mA, 3 F/mol, 7.2 hrs). The electrodes were then removed, substrate (1 eq., 1.2 mmol) was then added to the anodic compartment either via pipette or spatula and then caps were wrapped in parafilm to seal the reaction. After stirring overnight, the contents of each compartment were mixed and quenched with 100 mL of cold (0 ºC) saturated aqueous $CaCO_3$ solution This stirred for 1 hour until the aqueous layer measured pH 7. The mixture was extracted into CH_2Cl_2, dried with Na_2SO_4 and evaporated *en vacuo*. The product was then isolated via column chromatography.

Gram scale procedure

To a 20 mL HDPE vial, equipped with a magnetic stirrer bar, was added iodotoluene (1 eq., 7.2 mmol, 1.57 g), CH_2Cl_2 (5.6 mL), HFIP (1.8 mL) and 5.6HF:amine stock solution (18 mL). Alkene substrate (1 eq., 7.2 mmol) was then in one portion either via pipette or spatula and the vial was capped with the suba-seal equipped with platinum anode and cathode. The reaction was then subjected to electrolysis (3 F/mol, 30 mA, 20 hrs). The electrodes were then removed, and the vial was capped with an HDPE cap and the reaction was stirred overnight. The reaction was cooled to 0 ºC and quenched with 300 mL of cold (0 ºC) saturated aqueous $CaCO_3$ solution. This stirred for 1 hour until the aqueous layer measured pH 7. The mixture was extracted into CH_2Cl_2, dried with Na_2SO_4 and evaporated *en vacuo*. The product was then isolated via column chromatography.

Decagram scale-up procedure

To a 500 mL HDPE beaker, equipped with a magnetic stirrer bar, was added iodotoluene (1 eq., 26.3 mmol, 5.73 g), CH_2Cl_2 (15.4 mL), HFIP (6.6 mL) and 5.6HF:amine stock solution (66 mL). Alkene substrate (1 eq., 26.3 mmol) was then added in one portion and the beaked was capped with the lid with electrodes pre-placed. The reaction was then subjected electrolysis (3 F/mol, 30 mA, 70.5hrs). The

lid with electrodes were then removed, and the beaker was capped with another hole-less lid and allowed to stir for a further 12 hours. The reaction solution was then transferred to a 3L conical flask and cooled to 0 ºC. The reaction mixture was then slowly quenched with 1.2 L of cold saturated aqueous $CaCO_3$ solution. This stirred for 1 hour until the aqueous layer measured pH 7. The mixture was extracted into CH_2Cl_2 (3 x 350 mL), dried with Na_2SO_4 and evaporated *en vacuo*. The product was then isolated via column chromatography.

Typical bulk electrolysis traces

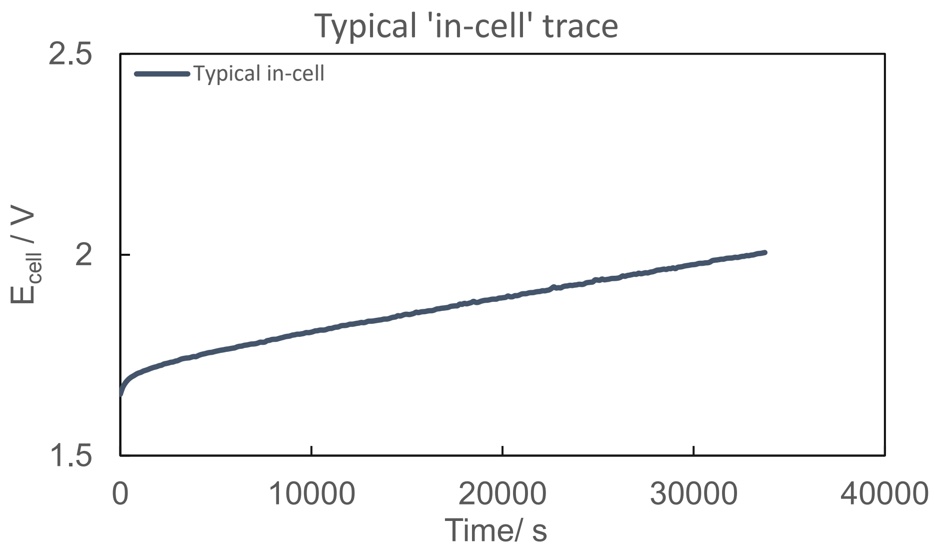

Figure S11: Typical 'in-cell' method trace.

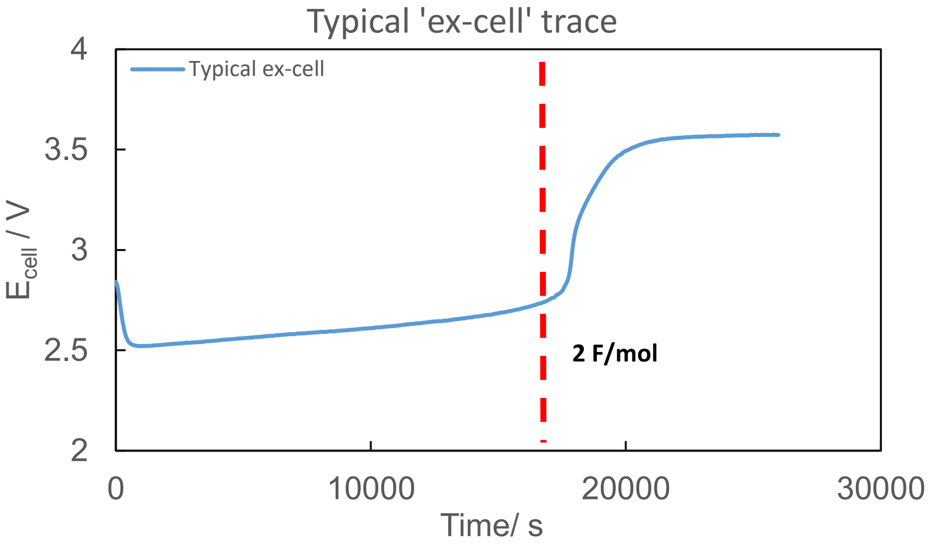

Figure S12: Typical 'ex-cell' method current trace. NOTE: the current spikes when 2 F has been passed, but it was found that 3 F/mol gave higher yields of difluorinated product.

Characterisation of products 3

(2,3-difluoropropyl)benzene, 3a

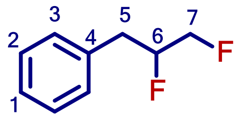

This product, **3a** was synthesised from allylbenzene using **'in-cell' procedure 1** and purified using silica gel chromatography (5% Et_2O:Hexane) to yield volatile colourless oil (122 mg, 65 %).

R_f = 0.3 (5% Et_2O:Hexane)

^{1}H NMR (500 MHz, $CDCl_3$): δ = 7.35 – 7.24 (5H, m, H^{Ar}), 4.95 – 4.77 (1H, m, H^6), 4.54 (1H, dddd, J = 44.2, 23.1, 10.5, 2.1 Hz, H^{7a}), 4.44 (1H, dddd, J = 43.4, 24.7, 11.0, 4.9 Hz, H^{7b}), 3.11 – 2.95 (2H, m, H^5).

^{13}C NMR (151 MHz, $CDCl_3$): δ = 135.7 (1C, C^1), 129.3 (2C, C^{Ar}) , 128.7 (2C, C^{Ar}), 127.0 (1C, C^4), 91.9 (1C, dd, J = 176.8, 21.2 Hz, C^6), 83.1 (1C, dd, J = 175.6, 22.1 Hz, C^7), 36.6 (1C, dd, J = 21.8, 6.6 Hz, C^5).

^{19}F NMR (376 MHz, $CDCl_3$): δ = -186.19 - -196.60 (1F, m, F^6), -231.84 - -232.19 (1F, m, F^7).

These data are consistent with that previously reported. [2]

(2,3-difluoropropyl)-4-(trifluoromethyl)benzene, 3b

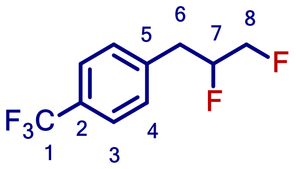

This product, **3b**, was synthesised from 4-trifluormethyl allylbenzene using **'in-cell' procedure 1** and purified using silica gel chromatography (5% EtOAc:Hexane → 10% EtOAc:Hexane) to yield a volatile colourless oil (196 mg, 73 %). Data is in line with previously reported literature. [11]

R_f = 0.3 (10% EtOAc:Hexane)

^{1}H NMR (500 MHz, $CDCl_3$): δ = 7.62 (2H, d, J = 8.3 Hz, H^4), 7.40 (2H, d, J = 8.3 Hz, H^3), 4.98 – 4.80 (1H, m, H^7), 4.59 (1H, dddd, J = 47.2, 21.0, 10.9, 3.1 Hz, H^{8a}), 4.48 (1H, dddd, J = 46.2, 24.4, 11.1, 4.4 Hz, H^{8b}), 3.19 – 3.03 (1H, m, H^6)

^{13}C NMR (151 MHz, $CDCl_3$): δ = 140.1 (1C, d, J = 4.9 Hz, C^5), 129.9 (2C, C^4), 129.6 (1C, q, J = 32.6 Hz, C^2), 125.8 (2C, q, J = 3.6 Hz, C^3), 124.2 (1C, q, J = 274.5 Hz, C^1), 91.5 (1C, dd, J = 177.1, 20.0 Hz, C^7), 83.1 (1C, dd, J = 174.7, 23.5 Hz, C^8), 36.6 (1C, dd, J = 21.9, 6.5 Hz, C^6).

^{19}F NMR (400 MHz, $CDCl_3$): δ = -187.77 - -188.18 (1F, m, F^7), - 231.81 - -232.16 (1F, m, F^8)

These data are consistent with that previously reported. [9]

1-bromo-2-(2,3-difluoropropyl)benzene, 3c

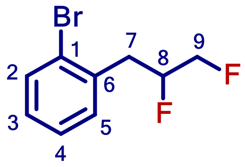

This product, **3c**, was synthesised from 2-bromo allylbenzene using **'in-cell' procedure 1** and purified using silica gel chromatography (100% Hexane) to yield a volatile colourless oil (205 mg, 73 %).

R_f = 0.4 (Hexane)

^{1}H NMR (500 MHz, $CDCl_3$): δ = 7.60 (1H, m, H^2), 7.34 (2H, m, $H^{3,4}$) 7.17 – 7.14 (1H, m, H^5), 5.07 – 4.89 (1H, m, H^8), 4.62 (1H, dddd, J = 47.3, 22.8, 11.0, 2.1 Hz, H^{9a}), 4.49 (1H, dddd, J = 47.6, 26.7, 11.0, 4.6 Hz, H^{9b}), 3.22 – 3.13 (2H, m, H^7)

^{13}C NMR (151 MHz, $CDCl_3$): δ = 135.5 (1C, C^6), 133.1 (1C, C^2), 132.0 (1C, $C^{3,4}$), 128.9 (1C, C^5), 127.81 (1C, $C^{3,4}$), 124.7 (1C, C^1), 90.59 (1C, dd, J = 176.8, 19.2 Hz, C^8), 83.37 (1C, dd, J = 174.7, 22.0 Hz, C^9), 36.79 (1C, dd, J = 22.6, 7.1 Hz, C^7).

^{19}F NMR (376 MHz, $CDCl_3$): δ = -187.41 - -187.81 (1F, m, F^8), -231.76 (1F, tdd, J = 47.8, 22.2, 12.5 Hz, F^9).

These data are consistent with that previously reported.[9]

1-(2,3-difluoropropyl)-4-fluorobenzene, 3d

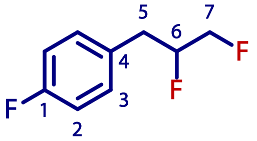

This product, **3d**, was synthesised from 4-fluoro allylbenzene using **'in-cell' procedure 1** and purified using silica gel chromatography (100% Hexane) to yield a volatile colourless oil (152 mg, 73 %).

R_f = 0.3 (Hexane)

^{1}H NMR (500 MHz, $CDCl_3$): δ = 7.24 – 7.17 (2H, m, H^2), 7.07 – 6.97 (2H, m, H^3), 4.94 – 4.70 (1H, m, H^6), 4.67 – 4.31 (2H, m, H^7), 3.09 – 2.89 (2H, m, H^5)

^{13}C NMR (151 MHz, $CDCl_3$): δ = 162.1 (1C, d, *J* = 242.7 Hz, C^1), 131.5 – 131.5 (1C, m, C^4), 131.0 (2C, d, *J* = 8.3 Hz, C^3), 115.7 (2C, d, *J* = 20.5 Hz, C^2), 91.9 (1C, dd, *J* = 177.4, 18.3 Hz, C^6), 83.1 (1C, d, *J* = 174.9, 22.3 Hz, C^7), 35.9 (1C, dd, *J* = 21.9, 6.7 Hz, C^5)

^{19}F NMR (376 MHz, $CDCl_3$): δ = -115.71 - -115.78 (1F, m, F^1), -186.94 - -187.34 (1F, m, F^6), -232.22 (1F, tdd, *J* = 47.4, 20.7, 14.2 Hz, F^7)

HRMS (APCI) calc: [M+H$^+$] ($C_8H_7F_3$) 174.0651; measured: 174.0646, = 2.87 ppm difference

IR (neat) ν_{max}/ cm^{-1}: 1514, 1456, 1158, 1013, 957, 878, 785, 756

1-(2,3-difluoropropyl)-2,3,4,5,6-pentafluorobenzene, 2e

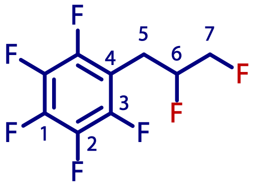

This product, **3e**, was synthesised from allylpentafluorobenzene **2e** using **'in-cell' procedure 1**. Purified using silica gel chromatography (5% EtOAc:Hexane → 10% EtOAc:Hexane) to yield a pale yellow oil (198 mg, 67 %).

R_f = 0.4 (10% EtOAc:Hexane)

^{1}H NMR (500 MHz, $CDCl_3$): δ = 4.95 – 4.77 (1H, m, *H^6*), 4.70 – 4.43 (1H, m, *H^7*), 3.25 – 3.18 (1H, m, *H^{5a}*), 3.09 – 2.99 (1H, m, *H^{5b}*).

^{13}C NMR (151 MHz, $CDCl_3$): δ = 145.6 (1C, dm, *C^2*), 140.6 (2C, dm, *C^1*), 137.6 (1C, dm, *C^3*), 109.4 (1C, tt, *J* = 18.7 Hz, 4.4, *C^4*), 89.4 (1C, dd, *J* = 178.9, 19.8 Hz, *C^6*), 83.1 (1C, dd, *J* = 176.5, 23.2 Hz, *C^7*), 23.9 (1C, dd, *J* = 23.4, 7.5 Hz, *C^5*).

^{19}F NMR (376 MHz, $CDCl_3$): δ = -142.30 – 142.44 (2F, m, *F^3*), -155.03 (1F, t, *J* = 20.1 Hz, *F^1*), -161.68 – 161.85 (2F, m, *F^2*), -189.15 - -189.60 (1F, m, *F^6*), -232.26 (1F, tdd, J= 46.3, 20.9, 13.7 Hz, *F^7*).

HRMS (EI^+) calc: [$M^{+\bullet}$] ($C_9H_5F_7$) 246.0274; measured: 246.0272 = 0.81 ppm difference

IR (neat) ν_{max}/ cm^{-1}: 1578, 1491, 1412, 1315, 1154, 1001, 850, 757

10,11-difluoroundecan-1-ol, 3f

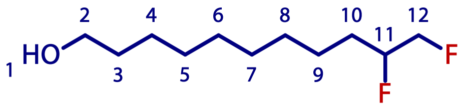

This product, **3f**, was synthesised from 10-undecen-1-ol using **'in-cell' procedure 1** and purified using silica gel chromatography (20% EtOAc:Hexane) to yield a viscous colourless oil (125 mg, 50 %) which solidified over the course of several weeks.

R_f = 0.2 (20% EtOAc:Hexane)

^{1}H NMR (500 MHz, $CDCl_3$): δ = 4.74 – 4.56 (1H, m, H^{11}), 4.55 – 4.28 (2H, m, H^{12}), 3.60 (2H, t, J = 6.6 Hz, H^2), 1.70 – 1.61 (1H, m, H^{10}), 1.60 – 1.20 (17H, m, H^{1-10}).

^{13}C NMR (151 MHz, $CDCl_3$): δ = 92.0 (1C, dd, J = 172.6, 18.9 Hz, C^{11}), 84.3 (1C, dd, J = 173.5, 23.2 Hz, C^{12}), 63.3 (1C, C^2), 32.9 (1C, C^3), 30.2 (1C, dd, J = 20.7, 6.4 Hz, C^3), 29.5 (1C, C^{4-9}), 29.4 (1C, C^{4-9}), 28.9 (1C, C^{4-9}), 28.9 (1C, C^{4-9}), 26.1 (1C, C^{4-9}), 24.9 (1C, C^{4-9}).

^{19}F NMR (376 MHz, $CDCl_3$): δ = -188.45 - -188.89 (1F, m, F^{11}), -229.81 (1F, tdd, J = 49.0, 19.1, 13.3 Hz, F^{12}).

These data are consistent with that previously reported. [2]

10,11-difluoroundecyl 4-methylbenzenesulfonate, 3g

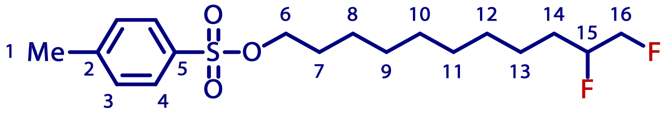

This product, **3g**, was synthesised from **2g** using **'in-cell' procedure 1** and purified using silica gel chromatography (20% EtOAc:Hexane) to yield a viscous colourless oil (304 mg, 70%) which solidified over the course of several weeks.

R_f = 0.3 (20% EtOAc:Hexane)

^{1}H NMR (500 MHz, CDCl$_3$): δ = 7.79 (2H, d, *J* = 8.3 Hz, H^4), 7.34 (2H, d, *J* = 8.3 Hz, H^3), 4.76 – 4.57 (1H, m, H^{15}), 4.58 – 4.36 (2H, m, H^{16}), 4.02 (2H, t, *J* = 6.5 Hz, H^6), 2.45 (3H, s, H^1), 1.78 – 1.24 (16H, m, $H^{7\text{-}14}$).

^{13}C NMR (151 MHz, CDCl$_3$): δ = 144.8 (1C, C^5), 133.4 (1C, C^2), 129.9 (1C, C^3), 128.0 (1C, C^4), 92.0 (1C, dd, *J* = 172.4, 19.1 Hz, C^{15}), 84.3 (1C, dd, *J* = 173.6, 23.0 Hz, C^{16}), 70.8 (1C, C^1), 30.2 (1C, dd, *J* = 20.7, 6.4 Hz, C^{14}), 29.4 (1C, $C^{7\text{-}13}$), 29.4 (1C, $C^{7\text{-}13}$), 29.0 (1C, $C^{7\text{-}13}$), 29.0 (1C, $C^{7\text{-}13}$), 25.5 (1C, $C^{7\text{-}13}$), 24.9 (1C, $C^{7\text{-}13}$), 24.8 (1C, $C^{7\text{-}13}$), 21.8 (1C, $C^{7\text{-}13}$).

^{19}F NMR (376 MHz, CDCl$_3$): δ = -188.58 - -188.99 (1F, m, F^{15}), -229.78 (1F, tdd, *J* = 46.6, 20.9, 12.8 Hz, F^{16}).

These data are consistent with that previously reported. [2]

10,11-difluoroundecyl 4-methoxybenzoate, 3h

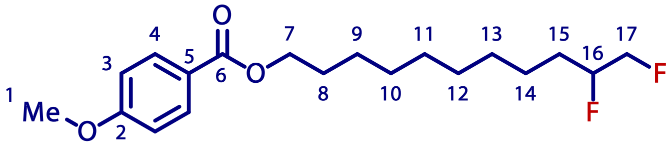

This product, **3h**, was synthesised from **2h** using **'in-cell' procedure 1** and purified using silica gel chromatography (20% EtOAc:Hexane) to yield a viscous colourless oil (295 mg, 72 %) which solidified over the course of several weeks.

R_f = 0.3 (20% EtOAc:Hexane)

^{1}H NMR (500 MHz, CDCl$_3$): δ = 8.00 (2H, d, J = 8.8 Hz, H^4), 6.92 (2H, d, J = 8.8 Hz, H^3), 4.76 – 4.58 (1H, m, H^{16}), 4.58 – 4.36 (2H, m, H^{17}), 4.28 (2H, t, J = 6.8 Hz, H^7), 3.86 (3H, s, H^1), 1.78 – 1.59 (4H, m, $H^{8\text{-}15}$**)**, 1.47 – 1.24 (12H, m, $H^{8\text{-}15}$)

^{13}C NMR (151 MHz, CDCl$_3$): δ = 166.6 (1C, C^6), 163.4 (1C, C^2), 131.7 (1C, C^4), 123.1(1C, C^5), 113.7 (1C, C^3), 92.0 (1C, dd, J = 172.4, 19.1 Hz, C^{16}), 84.3 (1C, dd, J = 173.6, 23.0 Hz, C^{17}), 65.0 (1C, C^1), 55.6 (1C, C^7), 30.2 (1C, dd, J = 20.7, 6.4 Hz, C^{15}), 29.5 (1C, $C^{8\text{-}14}$), 29.5 (1C, $C^{8\text{-}14}$), 29.4 (1C, $C^{8\text{-}14}$), 29.4 (1C, $C^{8\text{-}14}$), 28.9 (1C, $C^{8\text{-}14}$), 26.2 (1C, $C^{8\text{-}14}$), 24.9 (1C, $C^{8\text{-}14}$), 24.9 (1C, $C^{8\text{-}14}$).

^{19}F NMR (376 MHz, CDCl$_3$): δ = -188.51 - -188.93 (1F, m, F^{16}), -229.72 (1F, tdd, J = 48.8, 19.5, 13.4 Hz, F^{17})

These data are consistent with that previously reported. [2]

((2,3-difluoropropoxy)methyl)benzene, 3i

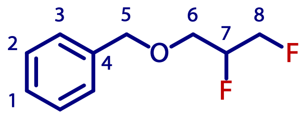

This product, **3i**, was synthesised from allyl benzyl ether using **'in-cell' procedure 1** and purified using silica gel chromatography (10% EtOAc:Hexane) to yield a viscous colourless oil (137 mg, 61 %).

R_f = 0.3 (10% EtOAc:Hexane)

^{1}H NMR (500 MHz, CDCl3): δ = 7.39 – 7.29 (5H, m, $H^{1,2,3}$), 4.95 – 4.72 (1H, m, H^7), 4.63 (2H, ddd, J = 46.5, 24.1, 4.2 Hz, $H^{8a,b}$), 4.59 (2H, s, H^5), 3.72 (2H, ddd, J = 20.0, 4.9, 1.5 Hz, H^6).

^{13}C NMR (151 MHz, $CDCl_3$): δ = 137.6 (1C, C^4), 128.7 (2C, C^2), 128.1 (1C, C^1), 127.9 (2C, C^3), 90.6 (1C, dd, J = 175.0, 20.7 Hz, C^7), 82.3 (1C, dd, J = 171.2, 23.8 Hz, C^8), 73.8 (1C, C^5), 67.9 (1C, dd, J = 25.2, 9.1 Hz, C^6).

^{19}F NMR (376 MHz, $CDCl_3$): δ = -195.83 - -196.22 (1F, m, F^7), -233.57 (1F, tdd, J = 47.4, 21.1, 12.9 Hz, F^8).

These data are consistent with that previously reported.[2]

1-((2,3-difluoropropoxy)methyl)-4-fluorobenzene, 3j

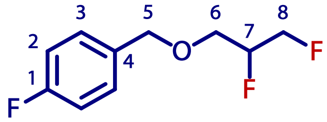

This product, **3j**, was synthesised from **2j** using **'in-cell' procedure 2** and purified using silica gel chromatography (10% Et_2O:Hexane) to yield a volatile, colourless oil (120 mg, 62 %).

R_f = 0.3 (10% Et_2O:Hexane)

^{1}H NMR (500 MHz, $CDCl_3$): δ = 7.33 – 7.28 (2H, m, H^2), 7.08 – 7.00 (2H, m, H^3), 4.92 – 4.74 (1H, m, H^7), 4.71 – 4.55 (2H, m, H^8), 4.54 (2H, s, H^5). 3.71 (2H, dd, *J* = 20.0, 4.8 Hz, H^6).

^{13}C NMR (126 MHz, $CDCl_3$): δ = 162.6 (1C, d, *J* = 246.1 Hz, C^1), 133.4 (1C, d, *J* = 3.2 Hz, C^4), 129.6 (2C, d, *J* = 8.2 Hz, C^3), 115.5 (2C, d, *J* = 21.5 Hz, C^2), 90.5 (1C, dd, *J* = 175.7, 19.9 Hz, C^7), 82.2 (1C, dd, *J* = 172.4, 23.4 Hz, C^8), 73.1 (1C, s, C^5), 68.0 (1C, dd, *J* = 24.2, 7.9 Hz, C^6).

^{19}F NMR (376 MHz, $CDCl_3$): δ = -114.17 - -114.31 (1F, m, F^1), -195.83 – -196.25 (1F, m, F^7), -233.71 (1F, tdd, *J* = 47.3, 21.2, 13.2 Hz, F^8).

HRMS (EI^+) calc: [$M^{+\bullet}$] ($C_{10}H_{11}F_3O$] 204.0757; measured: 204.0753 = 1.96 ppm difference

IR (neat) ν_{max}/ cm^{-1}: 1874, 1604, 1221, 1099, 1031, 926, 826

1-((2,3-difluoropropoxy)methyl)-4-nitrobenzene, 3k

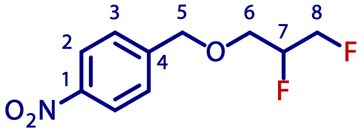

This product, **3k**, was synthesised from **2k** using **'in-cell' procedure 2** and purified using silica gel chromatography (30% EtOAc:Hexane) to yield a viscous yellow oil (133 mg, 72 %).

R_f = 0.35 (30% EtOAc:Hexane)

^{1}H NMR (500 MHz, $CDCl_3$): δ = 8.23 (2H, d, *J* = 9.1 Hz, H^2), 7.50 (2H, d, *J* = 9.1 Hz, H^3), 5.00 – 4.77 (1H, m, H^7), 4.76 – 4.51 (4H, m, $H^{5,8}$), 3.80 (2H, dd, *J* = 20.9, 4.9 Hz, H^6).

^{13}C NMR (126 MHz, $CDCl_3$): δ = 147.7 (1C, C^1), 145.2 (1C, C^4), 127.8 (2C, C^2), 123.9 (2C, C^3), 90.3 (1C, dd, *J* = 176.1, 20.1 Hz, C^7), 81.9 (1C, dd, *J* = 172.8, 23.8 Hz, C^8), 72.6 (1C, C^5), 68.8 (1C, dd, *J* = 24.0, 7.7 Hz, C^6).

^{19}F NMR (376 MHz, $CDCl_3$): δ = -195.89 – -196.29 (1F, m, F^7), -233.94 (1F, tdd, *J* = 47.1, 21.1, 13.6 Hz, F^8).

HRMS (EI$^+$) calc: [M-H$^+$] ($C_{10}H_{10}F_2NO_3$) 230.0623; measured: 230.0621 = 0.71 ppm difference

IR (neat) ν_{max}/ cm^{-1}: 1605, 1519, 1345, 1105, 1030, 850, 739

1-bromo-2-((2,3-difluoropropoxy)methyl)benzene, 3l

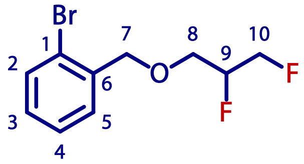

This product, **3l**, was synthesised from **2l** using **'in-cell' procedure 2** and purified using silica gel chromatography (2.5% EtOAc:Hexane) to yield a viscous colourless oil (115 mg, 55%).

R_f = 0.15 (2.5% EtOAc:Hexane)

^{1}H NMR (500 MHz, $CDCl_3$): δ = 7.56 (1H, dd, *J* = 8.0, 1.0 Hz, H^2), 7.45 (1H, dd, *J* = 8.0, 1.1 Hz, H^5), 7.33 (1H, td, *J* = 7.6, 1.1 Hz, H^3), 7.17 (1H, td, *J* = 7.8, 1.7 Hz, H^4), 4.97 – 4.78 (1H, m, H^9), 4.75 – 4.58 (4H, m, $H^{7,10}$), 3.81 (2H, ddd, *J* = 19.8, 4.9, 1.2 Hz, 4H, H^8).

^{13}C NMR (126 MHz, $CDCl_3$): δ = 136.9 (1C, C^1), 132.8 (1C, C^2), 129.4 (1C, C^4), 129.3 (1C, C^3), 127.6 (1C, C^5), 123.0 (1C, C^6), 90.4 (1C, dd, *J* = 175.8, 19.9 Hz, C^9), 82.3 (1C, dd, *J* = 172.4, 23.3 Hz, C^{10}), 73.1 (1C, C^7), 68.6 (1C, dd, *J* = 24.4, 8.0 Hz, C^8).

^{19}F NMR (376 MHz, $CDCl_3$): δ = -195.88 - -196.27 (1F, m, F^9), -233.56 (1F, tddt, *J* = 46.7, 21.1, 13.7, 1.27 Hz, F^{10}).

HRMS (EI$^+$) calc: [$M^{+\bullet}$] ($C_{10}H_{11}OBrF_2$) 263.9956; measured: 263.9954 = 0.76 ppm difference

IR (neat) ν_{max}/ cm^{-1}: 1722, 1568, 1489, 1440, 1363, 1104, 1028, 854, 755

2-((2,3-difluoropropoxy)methyl)pyridine, 3m

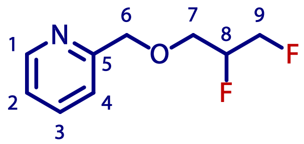

This product, **3m**, was synthesised from **2m** using **'in-cell' procedure 2** and purified using silica gel chromatography (40% EtOAc:Hexane) to yield a viscous, orange oil (76 mg, 50 %).

R_f = 0.2 (40% EtOAc:Hexane)

^{1}H NMR (500 MHz, $CDCl_3$): δ = 8.56 (1H, d, *J* = 4.2 Hz, H^1), 7.71 (1H, t, *J* = 7.3 Hz, H^2), 7.42 (1H, d, *J* = 7.7 Hz, H^4), 7.23 – 7.18 (1H, m, H^3), 4.97 – 4.78 (1H, m, H^8), 4.74 – 4.56 (4H, m, $H^{6,9}$), 3.82 (2H, ddd, *J* = 20.1, 5.1, 1.5 Hz, H^7)

^{13}C NMR (126 MHz, $CDCl_3$): δ = 157.8 (1C, C^5), 149.3 (1C, C^1), 136.9 (1C, C^2), 122.8 (1C, C^3), 121.5 (1C, C^4), 90.3 (1C, dd, *J* = 175.8, 19.9 Hz, C^8), 82.2 (1C, dd, *J* = 172.5, 23.4 Hz, C^9), 74.6 (1C, s, C^6), 68.8 (1C, dd, *J* = 24.1, 7.9 Hz, C^7).

^{19}F NMR (376 MHz, $CDCl_3$): δ = -195.94 - -196.34 (1F, m, F^8), -233.58 (1F, tdd, *J* = 46.7, 21.4, 12.3 Hz, F^9).

HRMS (ESI$^+$) Calc: [M+H$^+$] ($C_9H_{13}F_2NO$) 188.0881; measured: 188.0874 = 1.8 ppm difference.

IR (neat) ν_{max}/ cm^{-1}: 1675, 1591, 1436, 1112, 1030, 855, 761

4-((2,3-difluoropropoxy)methyl)pyridine, 3n

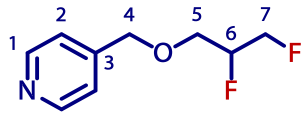

This product, **3n**, was synthesised from **2n** using **'in-cell' procedure 2** and purified using silica gel chromatography (50% EtOAc:Hexane) to yield a viscous yellow oil (53 mg, 35 %).

R_f = 0.2 (50% EtOAc:Hexane)

^{1}H NMR (500 MHz, $CDCl_3$): δ = 8.60 – 8.58 (2H, m, H^1), 7.26 – 7.23 (2H, m, H^2), 4.96 – 4.77 (1H, m, H^6), 4.73 – 4.57 (1H, m, $H^{4,7}$), 3.78 (2H, ddd, J = 20.7, 4.9, 1.1 Hz, H^5).

^{13}C NMR (126 MHz, $CDCl_3$): δ = 150.1 (2C, C^1), 146.7 (1C, C^3), 121.8 (2C, C^2), 90.3 (1C, dd, J = 176.1, 20.1 Hz, C^6), 82.0 (1C, dd, J = 172.7, 23.7 Hz, C^7), 72.1 (1C, s, C^4), 68.8 (1C, dd, J = 24.0, 7.8 Hz, C^5).

^{19}F NMR (376 MHz, $CDCl_3$): δ = -195.94 - -196.33 (1F, m, F^6), -233.88 (1F, tdd, J = 45.8, 22.7, 13.8 Hz, F^7)

HRMS (ESI$^+$) Calc: [M+H$^+$] ($C_9H_{13}F_2NO$) 188.0881; measured: 188.0879 = 1.06 ppm difference.

IR (neat) ν_{max}/ cm^{-1}: 1670, 1575, 1465, 1100, 1067, 834

1-((2,3-difluoropropoxy)methyl)-3,5-dinitrobenzene, 3o

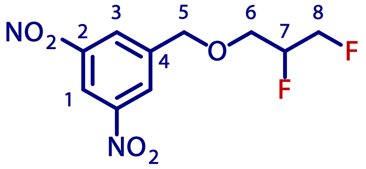

This product, **3o**, was synthesised from **2o** using **'in-cell' procedure 2** and purified using silica gel chromatography (30% EtOAc:Hexane) to yield a viscous orange oil (159 mg, 72 %).

R_f = 0.3 (30% EtOAc:Hexane)

^{1}H NMR (500 MHz, $CDCl_3$): δ = 8.97 (1H, t, *J* = 2.1 Hz, *H^1*), 8.53 (2H, dd, *J* = 2.0, 1.0 Hz, *H^3*), 5.03 – 4.82 (1H, m, *H^7*), 4.81 (2H, s, *H^5*), 4.78 – 4.54 (2H, m, *H^8*), 3.90 (2H, dd, *J* = 21.5, 4.9 Hz, *H^6*)

^{13}C NMR (126 MHz, $CDCl_3$): δ = 148.7 (1C, *C^4*), 142.6 (2C, *C^2*), 127.1 (2C, *C^3*), 118.2 (1C, *C^1*), 90.2 (1C, dd, *J* = 176.4, 20.2 Hz, *C^7*), 81.8 (1C, dd, *J* = 173.0, 23.9 Hz, *C^7*), 71.6 (1C, *C^5*), 69.3 (1C, dd, *J* = 23.7, 7.7 Hz, *C^6*).

^{19}F NMR (400 MHz, $CDCl_3$): δ = -195.69 – -196.08 (1F, m, *F^7*), -234.20 (1F, tdd, *J* = 47.1, 21.5, 13.3 Hz, *F^8*).

HRMS (EI^+) Calc: [M-H^+] ($C_{10}H_{10}F_2N_2O_5$) 276.0558; measured: 276.0561 = 1.5 ppm difference.

IR (neat) $ν_{max}$/ cm^{-1}: 1596, 1542, 1452, 1346, 1121, 1038, 912, 731

N-benzyl-N-(2,3-difluoropropyl)-4-methylbenzenesulfonamide, 3p

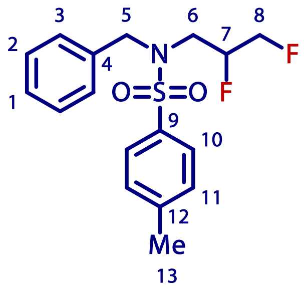

This product, **3p**, was synthesised from **2p** using **'in-cell' procedure 1** and purified using silica gel chromatography (10% Et_2O:Hexane → 20% Et_2O:Hexane) to yield a viscous, colourless oil (276 mg, 67 %).

R_f = 0.2 (10% Et_2O:Hexane)

^1H NMR (500 MHz, $CDCl_3$): δ = 7.73 (2H, d, *J* = 8.3 Hz, H^{10}), 7.38 – 7.29 (5H, m, $H^{11,1,3}$), 7.25 – 7.18 (2H, m, H^2), 4.77 – 4.16 (5H, m, $H^{7,8,5}$), 3.46 (1H, ddd, *J* = 23.0, 15.5, 5.1 Hz, H^{6a}), 3.33 (1H, td, *J* = 14.3, 7.0 Hz, H^{6b}), 2.45 (3H, s, H^{13}).

^{13}C NMR (126 MHz, $CDCl_3$): δ = 144.0 (1C, C^{12}) , 136.4 (1C, C^4), 135.5 (1C, C^{92}), 130.0 (1C, C^{10}), 128.9 (1C, $\underline{C_{Ar}}H$), 128.8 (2C, $\underline{C_{Ar}}H$)), 128.3 (2C, $\underline{C_{Ar}}H$)), 127.4 (1C, C^{11}), 90.5 (1C, dd, *J* = 176.9, 19.1 Hz, C^7), 82.3 (1C, dd, *J* = 173.9, 21.7 Hz, C^8), 53.4 (1C, C^5), 47.1 (1C, dd, *J* = 26.4, 8.4 Hz, C^6), 21.7 (1C, C^{13}).

^{19}F NMR (376 MHz, $CDCl_3$): δ = -191.14 - -191.53 (1F, m, F^7), -234.37 (1F, tdd, *J* = 47.1, 23.6, 11.9 Hz, F^8).

HRMS (ESI$^+$) Calc: [M+H$^+$] ($C_{17}H_{20}F_2NO_2S$) 340.1169; measured: 340.1177 = 2.3 ppm difference.

IR (neat) ν_{max}/cm^{-1}: 1494, 1446, 1323, 1152, 1089, 1017, 948, 917, 896, 813, 778, 728, 679

N-(4-chlorobenzyl)-N-(2,3-difluoropropyl)-4-methylbenzenesulfonamide, 3q

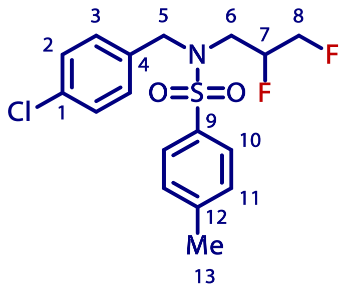

This product, **3q**, was synthesised from **2q** using **'in-cell' procedure 2** and purified using silica gel chromatography (15% EtOAc:Hexane) to yield a viscous, colourless oil (210 mg, 70 %) which solidified over the course of several weeks.

R_f = 0.3 (20% EtOAc:Hexane)

^{1}H NMR (500 MHz, CDCl$_3$): δ = 7.72 (1H, d, *J* = 8.3 Hz, H^{10}), 7.43 (1H, d, *J* = 8.4 Hz, H^{11}), 7.34 (1H, d, *J* = 8.4 Hz, H^3), 7.11 (1H, d, *J* = 8.4 Hz, H^2), 4.81 – 4.59 (1H, m, H^7), 4.51 – 4.25 (4H, m, $H^{5,8}$), 3.49 (1H, ddd, *J* = 25.1, 15.6, 4.4 Hz, H^{6a}), 3.30 (1H, td, *J* = 14.5, 7.3 Hz, H^{6b}), 2.45 (3H, s, H^{13}).

^{13}C NMR (126 MHz, CDCl$_3$): δ = 144.2 (1C, C^{12}), 136.3 (1C, C^{10}), 134.7 (1C, C^4), 132.0 (1C, C^1), 130.4 (2C, C^2), 130.1 (2C, C^{10}), 127.3 (2C, C^{11}), 122.3 (2C, C^3), 90.6 (1C, dd, *J* = 177.2, 19.2 Hz, C^7), 82.2 (1C, dd, *J* = 174.2, 22.0 Hz, C^8), 52.8 (1C, d, *J* = 1.7 Hz, C^5), 47.4 (1C, dd, *J* = 25.5, 8.3 Hz, C^6), 21.7 (1C, C^{13}).

^{19}F NMR (376 MHz, CDCl$_3$): δ = -190.99 - -191.39 (1F, m, F^7), -234.54 (1F, tdd, *J* = 46.7, 23.4, 12.5 Hz, F^8)

HRMS (ESI$^+$) Calc: [M+H$^+$] ($C_{17}H_{20}ClF_2NO_2S$) 374.0228; measured: 374.0288 = <0.1 ppm difference.

IR (neat) ν_{max}/ cm^{-1}: 1596, 1488, 1450, 1407, 1338, 1120, 1089, 1070, 1008, 911

N-(2,3-difluoropropyl)-4-methyl-N-(4-(trifluoromethyl)benzyl) benzenesulfonamide, 3r

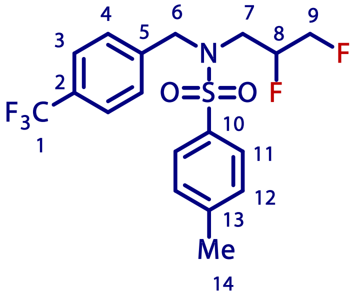

This product, **3r**, was synthesised from **2r** using **'in-cell' procedure 2** and purified using silica gel chromatography (15% EtOAc:Hexane) to yield a viscous, colourless oil (245 mg, 75 %).

R_f = 0.25 (15% EtOAc:Hexane)

^{1}H NMR (500 MHz, $CDCl_3$): δ = 7.74 (2H, d, J = 8.3 Hz, H^{11}), 7.58 (2H, d, J = 8.1 Hz, H^3), 7.41 – 7.33 (4H, m, $H^{4,12}$), 4.86 – 4.67 (1H, m, H^8), 4.63 – 4.32 (3H, m, H^9), 3.56 (1H, ddd, J = 26.4, 15.6, 4.0 Hz, H^{7a}), 3.37 (1H, td, J = 15.3, 7.6 Hz, H^{7b}), 2.45 (3H, s, H^{14}).

^{13}C NMR (126 MHz, $CDCl_3$): δ = 144.3 (1C, s, C^{13}), 140.0 (1C, s, C^{13}), 136.3 (1C, s, C^{10}), 130.4 (1C, q, J = 32.3 Hz, C^2) 130.1 (2C, s, C^{11}), 128.8 (2C, s, C^4), 127.3 (2C, s, C^{12}), 125.7 (2C, q, J = 3.7 Hz, C^3), 124.2 (1C, q, J = 273.3 Hz, C^1), 90.6 (1C, dd, J = 177.4, 19.3 Hz, C^8), 82.2 (1C, dd, J = 174.3, 22.2 Hz, C^9), 52.9 (1C, s, C^6), 47.8 (1C, dd, J = 24.8, 8.2 Hz, C^7), 21.7 (1C, s, C^{14})

^{19}F NMR (376 MHz, $CDCl_3$): δ = -62.48 (1F, s F^1), -191.05 – -191.45 (1F, m, F^9), -234.74 (1F, tdd, J = 47.5, 23.5, 12.6 Hz, F^9)

HRMS (MALDI) calc: [M+Na$^+$] ($C_{18}H_{18}F_5NO_2S$) 430.0871; measured: 430.0879 = 1.86 ppm difference.

IR (neat) $ν_{max}$/ cm^{-1}: 1590, 1470, 1350, 1115, 1055, 1025, 1005, 890

N-(4-bromobenzyl)-N-(2,3-difluoropropyl)-4-methylbenzenesulfonamide, 3s

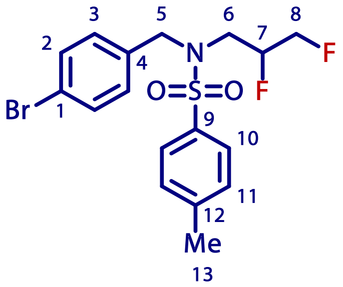

This product, **3s**, was synthesised from **2s** using **'in-cell' procedure 2** and purified using silica gel chromatography (20% EtOAc:Hexane) to yield a viscous, colourless oil (250 mg, 75 %), which solidified over the course of several weeks.

R_f = 0.2 (20% EtOAc:Hexane)

^{1}H NMR (500 MHz, $CDCl_3$): δ = 7.72 (2H, d, *J* = 8.0 Hz, H^{10}), 7.34 (2H, d, *J* = 8.0 Hz, H^{11}), 7.27 (2H, d, *J* = 8.5 Hz, H^3), 7.17 (2H, d, *J* = 8.5 Hz, H^2), 4.79 – 4.61 (1H, m, H^7), 4.59 - 4.28 (4H, m, $H^{5,8}$), 3.48 (1H, ddd, *J* = 25.0, 15.5, 4.5 Hz, H^{6a}), 3.31 (1H, td, *J* =c 14.5, 7.3 Hz, H^{6b}), 2.45 (3H, s, H^{13}).

^{13}C NMR (126 MHz, $CDCl_3$): δ = 144.2 (1C, C^{12}), 136.3 (1C, C^9), 134.2 (1C, C^4), 134.1 (1C, C^1), 130.1 (2C, C^2), 130.0 (2C, C^{10}), 129.0 (2C, C^3), 127.4 (2C, C^{11}), 90.4 (1C, dd, *J* = 177.2, 19.2 Hz, C^7), 82.1 (1C, dd, *J* = 174.2, 22.0 Hz, C^8), 52.7 (1C, d, *J* = 1.7 Hz, C^5), 47.3 (1C, dd, *J* = 25.5, 8.3 Hz, C^6), 21.7 (1C, C^{13}).

^{19}F NMR (376 MHz, $CDCl_3$): δ = -191.00 - -191.40 (1F, m, F^7), -234.54 (1F, tdd, *J* = 46.7, 23.4, 12.5 Hz, F^8).

HRMS (ESI$^+$) Calc: [M+H$^+$] ($C_{15}H_{19}BrF_2NO_2S$) 418.0282; measured: 418.0283 = 0.24 ppm difference.

IR (neat) ν_{max}/ cm^{-1}: 1597, 1491, 1450, 1339, 1159, 1089, 1010, 911

N-cyclopropyl-N-(2,3-difluoropropyl)-4-methylbenzenesulfonamide, 3t

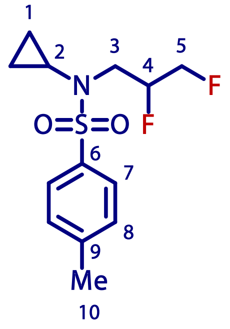

This product, **3t**, was synthesised from **2t** using **'in-cell' procedure 1** and purified using silica gel chromatography (15% EtOAc:Hexane) to yield a viscous, colourless oil (225 mg, 65 %).

Yield: 225 mg, 65%

R_f = 0.3 (15% EtOAc:Hexane)

^{1}H NMR (500 MHz, $CDCl_3$): δ = 7.74 (2H, d, *J* = 8.1 Hz, H^7), 7.34 (2H, d, *J* = 8.1 Hz, H^8), 5.10 – 4.88 (1H, m, H^4), 4.76 - 4.46 (2H, m, H^5), 3.53 (1H, ddd, *J* = 25.0, 15.1, 4.6 Hz, H^{3a}), 3.38 (1H, dddd, *J* = 15.1, 14.1, 7.4, 0.9 Hz, H^{3b}), 2.45 (3H, s, H^{10}), 2.12 – 2.07 (1H, m, H^2), 0.99 – 0.86 (1H, m, H^{1a}), 0.76 – 0.66 (1H, m, H^{1b}).

^{13}C NMR (126 MHz, $CDCl_3$): δ = 144.1 (1C, C^9), 134.5 (1C, C^6), 129.9 (2C, C^7), 128.0 (2C, C^8), 90.6 (1C, dd, *J* = 176.9, 18.7 Hz, C^4), 82.4 (1C, dd, *J* = 173.8, 21.6 Hz, C^5), 51.1 (1C, dd, *J* = 25.9, 8.3 Hz, C^3), 32.5 (1C, C^2), 21.7 (1C, C^{10}), 8.2 (1C, C^{1a}), 7.8 (1C, C^{1b}).

^{19}F NMR (376 MHz, $CDCl_3$): δ = -191.90 - -192.30 (1F, m, F^4), -235.07 (1F, m, F^5)

HRMS (ESI$^+$) Cal: [M+H$^+$] ($C_{13}H_{18}F_2NO_2S$) 290.1021; measured: 290.1019 = 0.09 ppm difference.

IR (neat) ν_{max}/cm^{-1}: 1655, 1487, 1343, 1162, 1089, 1029, 867, 816, 720, 670

1-(2,3-difluoropropyl)-4-methylbenzene, 3u

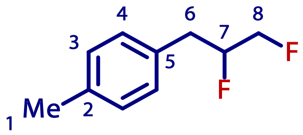

This product, **3u**, was synthesised from 4-allyltoluene using **'ex-cell' procedure 3** and purified using silica gel chromatography (2.5% Et_2O:Hexane) to yield a volatile, colourless oil (123 mg, 60 %).

R_f = 0.3 (2.5% Et_2O:Hexane)

^{1}H NMR (500 MHz, $CDCl_3$): δ = 7.18 – 7.10 (4H, m, $H^{3,4}$), 4.94 – 4.72 (1H, m, H^7) 4.61 – 4.31 (2H, m, H^8), 3.07 – 2.91 (2H, m, H^6), 2.34 (3H, s, H^1).

^{13}C NMR (151 MHz, $CDCl_3$): δ = 136.8 (1C, C^2), 132.7 (1C, C^5), 129.5 (2C, C^{Ar}), 129.3 (2C, C^{Ar}), 92.2 (1C, dd, J = 176.4, 19.6 Hz, C^7), 83.2 (1C, dd, J = 21.9, 6.7 Hz, C^6). 36.3 (1C, dd, J = 21.7, 6.7 Hz, C^6), 21.2 (1C, C^1).

^{19}F NMR (376 MHz, $CDCl_3$): δ = -186.08 – -186.48 (1F, m, F^7), -231.98 (1F, tdd, J = 46.9, 22.31, 14.2 Hz, F^8).

HRMS (EI$^+$) calc: [$M^{+\bullet}$] ($C_{10}H_{12}F_2$) 170.0902; measured: 170.0899 = 1.76 ppm difference

IR (neat) ν_{max}/ cm^{-1}: 1514, 1449, 1073, 1023, 912, 834, 800

1-(4-(2,3-difluoropropyl)phenyl)-N,N-dimethylmethanamine, 3v

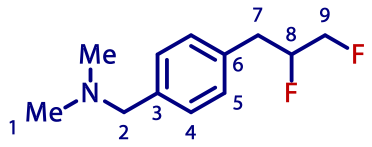

This product, **3v**, was synthesised from **2v** using **'ex-cell' procedure 3** and purified using silica gel chromatography (10% MeOH:EtOAc) to yield a volatile, colourless oil (107 mg, 42 %).

R_f = 0.2 (10% MeOH:EtOAc)

^{1}H NMR (500 MHz, $CDCl_3$): δ = 7.26 (2H, d, J = 8.1 Hz, H^{Ar}), 7.19 (2H, d, J = 8.1 Hz, H^{Ar}), 4.93 – 4.75 (1H, m, H^8), 4.61 – 4.33 (2H, m, H^9), 3.42 (2H, s, H^2), 3.08 – 2.92 (2H, m, H^7), 2.24 (6H, s, H^1).

^{13}C NMR (126 MHz, $CDCl_3$): δ = 137.4 (1C, s, C^3), 134.7 (1C, d, J = 5.6 Hz, C^6), 129.6 (2C, s, C^{Ar}), 129.4 (2C, s, C^{Ar}), 92.1 (1C, dd, J = 175.2, 16.9 Hz, C^8), 83.2 (1C, dd, J = 173.4, 22.6 Hz, C^9), 64.0 (1C, s, C^2), 45.3 (1C, s, C^1), 36.4 (1C, dd, J = 22.6, 6.2 Hz, C^7).

^{19}F NMR (376 MHz, $CDCl_3$): δ = -186.17 – -186.56 (1F, m, F^8), -231.96 (1F, tdd, J = 47.2, 20.9, 14.1 Hz, F^9).

HRMS (ESI): calc [M+H$^+$] ($C_{11}H_{15}F_2N$) 214.1407; measured: 214.1415 = 3.23 ppm difference

IR (neat) ν_{max}/cm^{-1}: 1514, 1455, 1353, 1257, 1075, 1020, 860

4-(2,3-difluoropropyl)-N,N-dimethylaniline, 3w

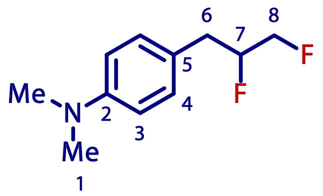

This product, **3w**, was synthesised from **2w** using **'ex-cell' procedure 3** and purified using silica gel chromatography (5% EtOAc:Hexane) to yield a colourless oil (120 mg, 50 %).

R_f = 0.35 (5% EtOAc:Hexane)

^{1}H NMR (500 MHz, $CDCl_3$): δ = 7.12 (2H, d, *J* = 8.7 Hz, H^4), 6.72 (2H, d, *J* = 8.7 Hz, H^3), 4.93 – 4.70 (1H, m, H^7), 4.64 – 4.33 (2H, m, H^8), 3.04 – 2.85 (8H, m, $H^{1,6}$).

^{13}C NMR (126 MHz, $CDCl_3$): δ = 149.8 (1C, C^2), 130.1 (2C, C^4), 123.3 (1C, d, *J* = 7.32 Hz, C^5), 113.0 (2C, C^3), 92.5 (1C, dd, *J* = 175.6, 18.9 Hz, C^7), 83.4 (1C, dd, *J* = 175.4, 22.6 Hz, C^8), 40.8 (2C, C^1), 35.6 (1C, dd, *J* = 21.2, 7.2 Hz, C^6).

^{19}F NMR (376 MHz, $CDCl_3$): δ = -185.48 – -185.87 (1F, m, F^7), -231.95 (1F, tddd, *J* = 47.6, 22.1, 13.9, 1.3 Hz, F^8).

HRMS (ESI): calc [M+H$^+$] ($C_{11}H_{15}F_2N$) 200.1245; measured: 200.1239 = 2.99 ppm difference

IR (neat) v_{max}/cm^{-1}: 1597, 1491, 1339, 1159, 1090, 1014, 917, 814, 762, 659

2-(2,3-difluoropropyl)-1,3,5-trimethylbenzene, 3x

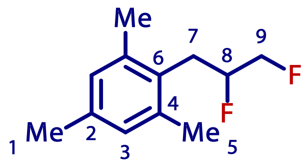

This product, **3x**, was synthesised from **2x** using **'ex-cell' procedure 3** and purified using silica gel chromatography (Hexane) to yield a colourless oil (95 mg, 40 %).

R_f = 0.3 (Hexane)

^{1}H NMR (500 MHz, $CDCl_3$): δ = 6.88 (2H, s, H^3), 4.93 – 4.69 (1H, m, H^8), 4.67 – 4.38 (2H, m, H^9), 3.17 – 2.91 (2H, m, H^7), 2.31 (6H, s, H^5), 2.27 (3H, s, H^1),

^{13}C NMR (126 MHz, $CDCl_3$): δ = 137.1 (2C, C^4), 136.5 (1C, C^2), 129.8 (1C, C^6), 129.4 (2C, C^3), 91.6 (1C, dd, J = 176.2, 19.3 Hz, C^8), 83.7 (1C, dd, J = 174.7, 24.3 Hz, C^9), 30.1 (1C, dd, J = 20.8, 6.4 Hz, C^7), 21.0 (1C, C^1), 20.3 (2C, C^5)

^{19}F NMR (376 MHz, $CDCl_3$): δ = -186.06 – -186.50 (1F, m, F^8), -230.02 (1F, tdd, J = 47.3, 21.6, 13.7 Hz, F^9)

HRMS (EI$^+$) calc: [$M^{+\bullet}$] ($C_{12}H_{16}F_2$) 198.1215; measured: 198.1214 = 0.50 ppm difference

IR (neat) ν_{max}/cm^{-1}: 1613, 1579, 1484, 1454, 1379, 1348, 1252, 1084, 1027, 853, 835

1-(2,3-difluoropropyl)-4-methoxybenzene, 3y

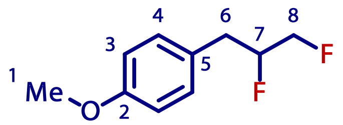

This product, **3y**, was synthesised from 4-allylanisole **2y** using **'ex-cell' procedure 3**. Attempts to purify **3x** using silica gel chromatography (10% EtOAc:Hexane) yielded a colourless oil that was contaminated with an unknown impurity (<10%), and thus an isolated yield was not calculated but a 10% NMR yield was observed.

$\mathbf{R_f}$ = 0.3 (10% EtOAc:Hexane)

^{1}H NMR (500 MHz, $CDCl_3$): 7.15 (2H, d, *J* = 8.6 Hz, H^3), 6.86 (2H, d, *J* = 8.6 Hz, H^4), 4.93 – 4.69 (1H, m, H^7), 4.64 – 4.30 (2H, m, H^8), 3.80 (3H, s, H^1), 3.07 – 2.87 (2H, H^6).

^{13}C NMR (126 MHz, $CDCl_3$): 158.8 (1C, C^2), 130.5 (2C, C^4), 127.7 (1C, C^5), 114.3 (2C, C^3), 92.3 (1C, dd, *J* = 76.2, 19.2 Hz, C^7), 83.2 (1C, dd, *J* = 173.8, 22.8, C^8), 55.4 (1C, C^1), 35.8 (1C, dd, *J* = 21.6, 6.9 Hz, C^6).

^{19}F NMR (376 MHz, $CDCl_3$): -186.20 - -186.62 (1F, m, F^7), -232.12 (1F, tdd, *J* = 47.5, 20.1, 14.2 Hz, F^8).

HRMS (EI$^+$) calc: [$M^{+\bullet}$] ($C_{10}H_{12}F_2O$) 186.0856; measured: 186.0861 = 0.76 ppm difference

IR (neat) ν_{max}/cm^{-1}: 1657, 1512, 1491, 1432, 1383, 1252, 1021, 878, 832

3,4-difluoro-1-morpholinobutan-1-one, 3z

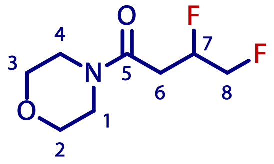

This product, **3z**, was synthesised from **2z** using **'ex-cell' procedure 3** and purified using silica gel chromatography (EtOAc) to yield a colourless oil (86 mg, 37 %).

R_f = 0.35 (EtOAc)

^{1}H NMR (500 MHz, $CDCl_3$): δ = 5.28 – 5.07 (1H, m, H^7), 4.80 – 4.47 (2H, m, H^8), 3.70 – 3.42 (8H, m, $H^{1,2,3,4}$), 2.92 – 2.82 (1H, m, H^{6a}), 2.76 – 2.63 (1H, m, H^{6b}).

^{13}C NMR (126 MHz, $CDCl_3$): δ = 167.3 (1C, d, *J* = 8.6 Hz, C^5), 89.0 (1C, dd, *J* = 170.7, 19.2 Hz, C^7), 83.9 (1C, dd, *J* = 174.3, 20.9 Hz, C^8), 66.9 (1C, s, $C^{1\ or\ 4}$), 66.6 (1C, s, $C^{1\ or\ 4}$), 46.2 (1C, s, $C^{2\ or\ 3}$), 42.1 (1C, s, $C^{2\ or\ 3}$), 33.3 (1C, dd, *J* = 24.5, 6.6 Hz, C^6).

^{19}F NMR (376 MHz, $CDCl_3$): δ = -188.10 – -188.52 (1F, m, F^7), -233.85 (1F, tdd, *J* = 47.6, 24.5, 11.6 Hz, F^8).

HRMS (ESI): Calc [M+H$^+$] ($C_8H_{13}F_2NO_2$) 194.0987; measured: 194.0980 = 4.12 ppm difference

IR (neat) v_{max}/cm^{-1}: 1732, 1528, 1348, 1168, 1088, 1031, 854, 609

N-(2,3-difluoropropyl)-N-(4-fluorophenyl)-4-methylbenzenesulfonamide, 3aa

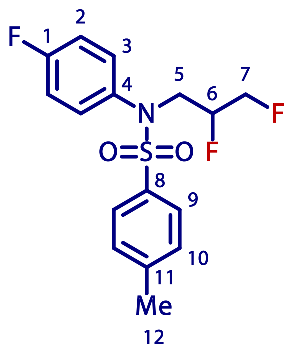

This product, **3aa**, was synthesised from **2aa** using **'ex-cell' procedure 3** and purified using silica gel chromatography (20% EtOAc:Hexane) to yield a viscous orange oil (288 mg, 70 %).

R_f = 0.2 (20% EtOAc:Hexane)

^{1}H NMR (500 MHz, $CDCl_3$): δ = 7.46 (2H, d, J = 8.3 Hz, H^9), 7.27 (2H, d, J = 6.9 Hz, H^{10}), 7.05 – 6.98 (4H, m, $H^{2,3}$), 4.92 – 4.49 (3H, m, $H^{6,7}$), 3.83 (1H, d, J = 5.9 Hz, H^{5a}), 3.81 – 3.79 (1H, m, H^{5b}), 2.43 (3H, s, H^{12}).

^{13}C NMR (126 MHz, $CDCl_3$): δ = 162.3 (1C, d, J = 249.0 Hz, C^1), 144.3 (1C, C^{11}), 135.8 (2C, d, J = 3.3 Hz, C^4), 134.6 (1C, C^8), 130.8 (2C, d, J = 8.8 Hz, C^3), 129.8 (1C, C^9), 127.9 (1C, C^{10}), 116.4 (2C, d, J = 22.8 Hz, C^2), 89.9 (1C, dd, J = 178.4, 19.3 Hz, C^7), 82.2 (1C, dd, J = 174.3, 21.8 Hz, C^7), 51.0 (1C, dd, J = 26.6, 8.1 Hz, C^5), 21.7 (1C, C^{12}).

^{19}F NMR (376 MHz, $CDCl_3$): δ = -112.14 – -112.20 (1F, m, F^1), -192.75 – -193.13 (1F, m, F^6), -234.58 (1F, tdd, J = 46.1, 24.6, 12.9 Hz, F^7).

HRMS (ESI$^+$) Calc: [M+H$^+$] ($C_{16}H_{17}F_3NO_2S$) 344.0927; measured: 344.0923 = 1.20 ppm difference

IR (neat) ν_{max}/cm^{-1}: 1598, 1504, 1349, 1213, 1150, 1090, 1017, 875, 810

***N*-(2,3-difluoropropyl)-*N*-(4-fluorophenyl)-4-nitrobenzenesulfonamide, 3ab**

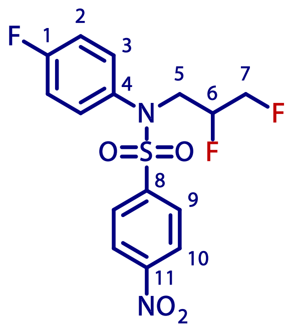

This product, **3ab**, was synthesised from **2ab** using **'ex-cell' procedure 3** and purified using silica gel chromatography (20% EtOAc:Hexane) to yield a viscous yellow oil (314 mg, 70 %) which solidified over the course of several weeks.

R_f = 0.25 (20% EtOAc:Hexane)

^{1}H NMR (500 MHz, $CDCl_3$): δ = 8.33 (2H, d, *J* = 8.9 Hz, H^{9}), 7.79 (2H, d, *J* = 8.9 Hz, H^{10}), 7.06 (4H, d, *J* = 6.4 Hz, $H^{2,3}$), 4.90 – 4.45 (3H, m, $H^{6,7}$), 3.98 (1H, td, *J* = 13.8, 7.6 Hz, H^{5a}), 3.83 (1H, ddd, *J* = 23.4, 13.4, 4.7 Hz, H^{5b}).

^{13}C NMR (126 MHz, $CDCl_3$): δ = 162.6 (1C, d, *J* = 250.7 Hz, C^{1}), 150.5 (1C, C^{11}), 143.7 (1C, C^{8}), 134.6 (2C, d, *J* = 3.3 Hz, C^{4}), 130.9 (2C, d, *J* = 8.9 Hz, C^{3}), 129.1 (2C, C^{10}), 124.4 (2C, C^{9}), 116.9 (2C, d, *J* = 22.9 Hz, C^{2}), 89.5 (1C, dd, *J* = 179.5, 19.6 Hz, C^{6}), 81.8 (1C, dd, *J* = 175.1, 22.2 Hz, C^{7}), 51.6 (1C, dd, *J* = 25.5, 7.9 Hz, C^{5}).

^{19}F NMR (376 MHz, $CDCl_3$): δ = -110.66 – -110.73 (1F, m, F^{1}), -193.01 – -193.39 (1F, m, F^{6}), -234.83 (1F, tdd, *J* = 45.9, 23.9, 13.1 Hz, F^{7}).

HRMS (APCI) calc: [M+H$^+$] ($C_{15}H_{13}N_2O_4F_3S$) 375.0621; measured: 375.0616 = 1.33 ppm difference

IR (neat) ν_{max}/cm^{-1}: 1605, 1530, 1515, 1350, 1312, 1150, 1090, 854

N-(2,3-difluoropropyl)-N-(4-fluoro-2-methylphenyl)-4-nitrobenzenesulfonamide, 3ac

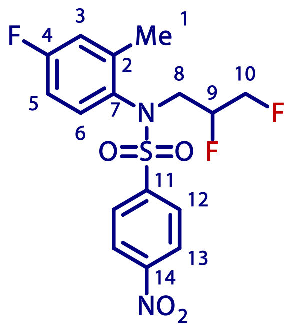

This product, **3ac**, was synthesised from **2ac** using **'ex-cell' procedure 3** and purified using silica gel chromatography (20% EtOAc:Hexane) to yield a viscous, colourless oil (256 mg, 55 %). NMR data suggests two rotamers in solution.

R$_f$ = 0.3 (20% EtOAc:Hexane)

^{1}H NMR (500 MHz, CDCl$_3$): Major rotamer δ = 8.35 (2H, d, *J* = 8.3 Hz, H^{12}), 7.87 (2H, d, *J* = 8.3 Hz, H^{13}), 7.07 – 6.98 (1H, m, H^3), 6.84 – 6.77 (1H, m, H^5), 6.58 – 6.53 (1H, m, H^6), 4.82 – 4.39 (3H, m, $H^{9,10}$), 4.09 – 3.94 (1H, m, H^{8a}), 3.78 – 3.54 (1H, m, H^{8b}), 2.39 (3H, s, H^1). Minor rotamer δ = 8.36 (2H, d, *J* = 8.3 Hz, H^{12}), 7.86 (2H, d, *J* = 8.3 Hz, H^{13}), 7.07 – 6.98 (1H, m, H^3), 6.84 – 6.77 (1H, m, H^5), 6.68 – 6.64 (1H, m, H^6), 4.82 – 4.39 (3H, m, $H^{9,10}$), 4.09 – 3.94 (1H, m, H^{8a}), 3.78 – 3.54 (1H, m, H^{8b}), 2.27 (3H, s, H^1).

^{13}C NMR (126 MHz, CDCl$_3$): Major rotamer δ = 162.6 (1C, d, *J* = 250.1 Hz, C^4), 150.5 (1C, C^{14}), 144.4 (1C, C^{11}), 142.6 (1C, d, *J* = 8.2 Hz, C^2), 133.1 (1C, d, *J* = 3.3 Hz, C^7), 130.4 (1C, d, *J* = 9.3 Hz, C^6), 129.4 (2C, C^{13}), 124.5 (2C, C^{12}), 118.9 (1C, d, *J* = 22.7 Hz, C^3), 114.2 (1C, d, *J* = 21.2 Hz, C^5), 89.7 (1C, dd, *J* = 179.7, 18.7 Hz, C^9), 82.0 (1C, dd, *J* = 174.9, 21.9 Hz, C^{10}), 51.9 (1C, dd, *J* = 23.7, 8.4 Hz, C^8), 18.6 (1C, C^1). Minor rotamer δ = 162.5 (1C, d, *J* = 250.1 Hz, C^4), 150.4 (1C, C^{14}), 144.0 (1C, C^{11}), 142.5 (1C, d, *J* = 8.2 Hz, C^2), 133.2 (1C, d, *J* = 3.3 Hz, C^7), 130.3 (1C, d, *J* = 9.3 Hz, C^6), 129.3 (2C, C^{13}), 124.3 (2C, C^{12}), 118.7 (1C, d, *J* = 22.7 Hz, C^3), 114.0 (1C, d, *J* = 21.2 Hz, C^5), 88.7 (1C, dd, *J* = 179.7, 18.7 Hz, C^9), 82.1 (1C, dd, *J* = 174.9, 21.9 Hz, C^{10}), 52.3 (1C, dd, *J* = 23.7, 8.4 Hz, C^8), 18.5 (1C, C^1).

^{19}F NMR (376 MHz, $CDCl_3$): Major rotamer δ = -110.92 – -110.99 (1F, m, *F^4*), -191.93 – -192.36 (1F, m, *F^9*), -234.25 – -234.65 (1F, m, *F^{10}*). Minor rotamer δ = -111.01 – -111.08 (1F, m, *F^4*), -192.77 – -193.19 (1F, m, *F^9*), -234.25 – -234.65 (1F, m, *F^{10}*).

HRMS (APCI) calc: [$M+H^+$] ($C_{16}H_{15}N_2O_4F_3S$) 389.0777; measured: 389.0774 = 0.77 ppm difference

IR (neat) ν_{max}/cm^{-1}: 1597, 1557, 1356, 1265, 1095

N-(2,3-difluoropropyl)-N-(4-iodophenyl)-4-nitrobenzenesulfonamide, 3ad

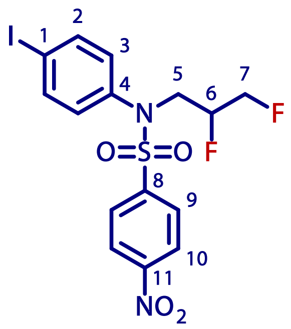

This product, **3ad**, was synthesised from **2ad** using **'ex-cell' procedure 3** and purified using silica gel chromatography (17% EtOAc:Hexane) to yield a viscous, yellow oil (347 mg, 60 %).

R_f = 0.2 (20% EtOAc:Hexane)

^{1}H NMR (500 MHz, $CDCl_3$): 8.33 (2H, d, *J* = 8.9 Hz, H^9), 7.78 (2H, d, *J* = 9.0 Hz, H^{10}), 7.70 (2H, d, *J* = 8.6 Hz, H^2), 6.82 (2H, d, *J* = 8.6 Hz, H^3), 4.89 – 4.43 (3H, m, $H^{6,7}$), 3.96 (1H, td, *J* = 13.7, 7.6 Hz, H^{5a}), 3.82 (1H, ddd, *J* = 22.8, 13.3, 4.7 Hz, H^{5b}).

^{13}C NMR (126 MHz, $CDCl_3$): δ = 150.5 (1C, C^{11}), 143.5 (1C, C^8), 139.1 (2C, C^2), 138.5 (1C, C^1), 130.6 (2C, C^3), 129.0 (1C, C^{10}), 124.5 (1C, C^9), 94.9 (1C, C^4), 89.5 (1C, dd, *J* = 179.6, 19.5 Hz, C^6), 81.7 (1C, dd, *J* = 175.1, 22.1 Hz, C^7), 51.2 (1C, dd, *J* = 25.6, 8.0 Hz, C^5).

^{19}F NMR (126 MHz, $CDCl_3$): δ = -192.86 – -193.25 (1F, m, F^6), -234.86 (1F, tdd, *J* = 47.1, 22.4, 12.5 Hz, F^7)

HRMS (APCI) calc: [M+H$^+$] ($C_{15}H_{13}N_2O_4SF_2IS$) 482.9682; measured: 482.9675 = 1.45 ppm difference

IR (neat) ν_{max}/cm^{-1}: 1517, 1339, 1159, 1090, 1014, 917, 659

N-(2-bromophenyl)-N-(2,3-difluoropropyl)-4-nitrobenzenesulfonamide, 3ae

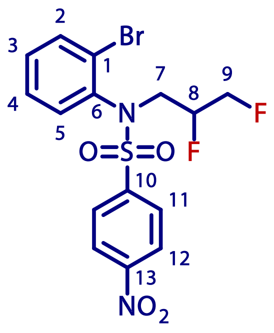

This product, **3ae**, was synthesised from **2ae** using **'ex-cell' procedure 3** and purified using silica gel chromatography (20% EtOAc:Hexane) to yield a viscous, colourless oil (313 mg, 60 %). NMR data suggests two rotamers in solution.

R_f = 0.2 (20% EtOAc:Hexane)

^{1}H NMR (500 MHz, $CDCl_3$): Major rotamer δ = 8.32 (2H, d, *J* = 8.3 Hz, H^{11}), 7.89 (2H, d, *J* = 8.3 Hz, H^{12}), 7.66 – 7.61 (1H, m,), 7.42 – 7.32 (1H, m, H^{Ar}), 7.32 – 7.21 (2H, m, H^{Ar}), 4.77 – 4.47 (1H, m, $H^{8,9}$), 4.30 – 4.21 (1H, m, H^9), 3.89 – 3.78 (1H, m, H^9). Minor rotamer δ = 8.31 (2H, d, *J* = 8.3 Hz, H^{11}), 7.85 (2H, d, *J* = 8.3 Hz, H^{12}), 7.55 – 7.51 (2H, m, H^{Ar}), 7.42 – 7.32 (1H, m, H^{Ar}), 7.32 – 7.21 (1H, m, H^{Ar}), 5.18 – 4.98 (1H, m. H^8), 4.77 – 4.47 (2H, m, H^9), 4.15 – 4.04 (1H, m, H^7), 3.89 – 3.78 (1H, m, H^7).

^{13}C NMR (126 MHz, $CDCl_3$): Major rotamer δ = 150.5 (1C, C^{13}), 145.2 (1C, C^{10}), 136.3 (1C, C^6), 134.7 (1C, C^4), 132.9 (1C, C^3), 131.1 (1C, C^2), 129.3 (1C, C^5), 128.8 (1C, C^{12}), 124.9 (1C, C^1), 124.3 (1C, C^{11}), 88.5 (1C, dd, *J* = 179.7, 19.5 Hz, C^8), 82.0 (1C, dd, *J* = 175.3, 21.9 Hz, C^9), 50.2 (1C, dd, *J* = 24.9, 8.1 Hz, C^7). Minor rotamer δ = 150.4 (1C, C^{13}), 144.7 (1C, C^{10}), 137.4 (1C, C^6), 134.4 (1C, C^4), 134.2 (1C, C^3), 130.9 (1C, C^2), 129.1 (1C, C^5), 128.7 (1C, C^{12}), 125.5 (1C, C^1), 124.4 (1C, C^{11}), 91.4 (1C, dd, *J* = 179.7, 19.5 Hz, C^8), 82.1 (1C, dd, *J* = 175.3, 21.9 Hz, C^9), 52.0 (1C, dd, *J* = 24.9, 8.1 Hz, C^7).

^{19}F NMR (126 MHz, $CDCl_3$): Major rotamer δ = -191.28 - -192.08 (1F, m, F^8), -233.97 - -234.34 (1F, m, F^9). Minor rotamer δ = -193.10 - -193.50 (1F, m, F^8), -234.92 - -235.31 (1F, m, F^9).

HRMS (ESI$^+$) Calc: [M+H$^+$] ($C_{15}H_{14}BrF_2N_2O_4S$) 433.9747; measured: 433.9251 = 1.5 ppm difference

IR (neat) ν_{max}/cm^{-1}: 1606, 1510, 1471, 1357, 1311, 1277, 1095, 1031, 834, 895, 785

N-(2,3-difluoropropyl)-N-mesityl-4-nitrobenzenesulfonamide, 3af

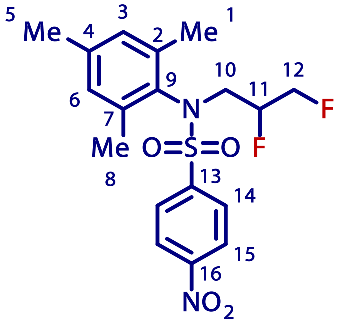

This product, **3af**, was synthesised from **2af** using **'ex-cell' procedure 3** and purified using silica gel chromatography (10% EtOAc:Hexane) to yield a colourless powder (167 mg, 35%).

R_f = 0.2 (10% EtOAc:Hexane)

^{1}H NMR (500 MHz, CDCl$_3$): δ = 8.34 (2H, d, *J* = 8.9 Hz, H^{14}), 7.94 (2H, d, *J* = 9.0 Hz, H^{15}), 6.93 (1H, s, $\underline{C_{Ar}}H$), 6.85 (1H, s, $\underline{C_{Ar}}H$), 4.89 – 4.43 (3H, m, $H^{11,12}$), 3.98 (1H, dt, *J* = 15.2, 8.6 Hz, H^{10}), 3.72 (1H, ddd, *J* = 32.1, 15.2, 2.9 Hz, H^{10}), 2.28 (3H, s, H^5), 2.19 (3H, s, H^1), 1.80 (3H, s, H^8).

^{13}C NMR (126 MHz, CDCl$_3$): δ = 150.2 (1C, C^{16}), 145.9 (1C,C^{13}), 139.2 (1C, C^7), 139.0 (1C, C^2), 137.7 (1C, $C^{9,}$), 133.5 (1C, C^5), 130.7 (1C, C^3), 130.3 (1C, C^6), 129.1 (2C,C^{15}), 124.4 (2C, C^{14}), 89.8 (1C, dd, *J* = 179.6, 17.5 Hz, C^{11}), 82.3 (1C, dd, *J* = 175.5, 22.9 Hz, C^{12}), 52.2 (1C, dd, *J* = 23.9, 8.2 Hz, C^{10}), 21.0 (1C,C^5) , 19.1 (1C, d, *J* = 2.1 Hz, C^1), 18.6 (1C, d, *J* = 1.8 Hz, C^8).

^{19}F NMR (376 MHz, CDCl$_3$): δ = -190.17 – -190.58 (1F, m, F^{11}), -234.66 (1F, tdd, *J* = 47.3, 24.1, 12.7 Hz, F^{12}).

HRMS (APCI) calc: [M+H$^+$] ($C_{18}H_{20}N_2O_4F_2S$) 399.1185; measured: 399.1183 = 0.50 ppm difference

IR (neat) v_{max}/cm^{-1}: 1530, 1350, 1165, 1088, 854, 738

N-(2,3-difluoro-2-methylpropyl)-N-(4-fluorophenyl)-4-nitrobenzenesulfonamide, 3ag

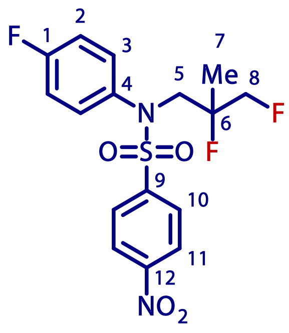

This product, **3ag**, was synthesised from **2ag** using **'ex-cell' procedure 3** and purified using silica gel chromatography (5% EtOAc:Hexane) to yield a colourless powder (280 mg, 60 %).

R_f = 0.15 (5% EtOAc:Hexane)

^{1}H NMR (500 MHz, $CDCl_3$): δ = 8.31 (2H, d, *J* = 8.9 Hz, H^{10}), 7.72 (2H, d, *J* = 9.0 Hz, H^{11}), 7.06 – 6.99 (4H, m, H^{Ar}), 4.55 – 4.27 (2H, m, H^6), 3.94 – 3.87 (2H, m, H^5), 1.48 (3H, dd, *J* = 21.7, 2.3 Hz, H^8).

^{13}C NMR (126 MHz, $CDCl_3$): δ = 162.3 (1C, d, *J* = 250.1 Hz, C^1), 150.5 (1C, C^{12}), 143.4 (1C, C^9) , 135.3 (1C, d, *J* = 3.3 Hz, C^4), 130.6 (2C, d, *J* = 8.7 Hz, C^3), 129.0 (2C, C^{11}), 124.4 (2C, C^{10}), 116.6 (2C, d, *J* = 22.9 Hz, C^2), 95.1 (1C, dd, *J* = 177.2, 18.1 Hz, C^6), 84.8 (1C, dd, *J* = 178.1, 26.4 Hz, C^8), 55.1 (1C, dd, *J* = 25.8, 5.2 Hz, C^5), 19.2 (1C, dd, *J* = 23.0, 4.6 Hz, C^7).

^{19}F NMR (376 MHz, $CDCl_3$): δ = -111.38 - -111.45 (1F, m, F^1), -156.69 - -157.05 (1F, m, F^6), -230.76 (1F, dt, *J* = 46.5, 13.1 Hz, F^8).

HRMS (APCI) calc: [M+H$^+$] ($C_{16}H_{15}N_2O_4F_3S$) 389.0777; measured: 389.0777 = 0.00 ppm difference

IR (neat) ν_{max}/ cm^{-1}: 1616, 1530, 1507, 1355, 1087, 812

1-(2,2-difluoroethyl)-4-fluorobenzene, 3ah

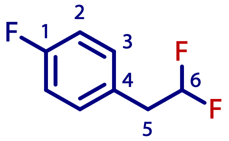

To each compartment of PTFE divided cell equipped with a nafion membrane and stirring bars, $CHCl_3$ (2.1 mL), HFIP (0.9 mL) and 4.5HF:amine stock solution (3 mL) were added. To the anodic compartment, iodotoluene (1 eq., 262 mg, 1.2 mmol) was then added. Each compartment was then capped and wrapped in parafilm. A platinum electrode was inserted into each compartment, and the reaction was subjected to electrolysis (13.4 mA, 3 F/mol, 7.2 hrs). The electrodes were then removed, 4-fluoro styrene (1 eq., 1.2 mmol, 167 µL) was then added to the anodic compartment and then caps were wrapped in parafilm to seal the reaction. After stirring overnight, the anodic compartment was quenched with 75 mL of cold (0 ºC) saturated aqueous $CaCO_3$ solution. This stirred for 1 hour until the aqueous layer measured pH 7. The mixture was extracted into CH_2Cl_2, dried with Na_2SO_4 and evaporated *en vacuo*. The product was then isolated via column chromatography. This product, **3ah**, was purified using silica gel chromatography (Hexane) to yield a colourless oil (87 mg, 45 %).

R_f = 0.5 (Hexane)

^{1}H NMR (500 MHz, $CDCl_3$): δ = 7.22 – 6.95 (4H, m, $H^{2,3}$), 5.81 (1H, tt, J = 56.3, 3.7 Hz, H^6), 3.09 (2H, dt, J = 16.7, 4.7 Hz, H^5).

^{13}C NMR (126 MHz, $CDCl_3$): δ = 162.1 (1C, d, J = 241.3 Hz, C^1), 131.5 – 131.5 (1C, m, C^4), 131.0 (2C, d, J = 7.8 Hz, C^3), 115.7 (2C, d, J = 20.9 Hz, C^2), 113.5 (1C, d, J = 241.3 Hz, C^6), 40.2 (1C, t, J = 22.5 Hz, C^5)

^{19}F NMR (376 MHz, $CDCl_3$): δ = -155.7 – -155.4 (1F, m, F^1), -155.5 (2F, dt, J = 55.7, 18.5 Hz, F^6).

Data are in agreement with that previously reported. [17]

1-(1,2-difluoroethyl)-3-nitrobenzene, 3ai

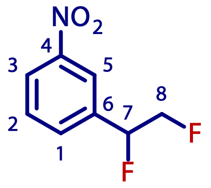

To each compartment of PTFE divided cell equipped with a nafion membrane and stirring bars, $CHCl_3$ (2.1 mL), HFIP (0.9 mL) and 4.5HF:amine stock solution (3 mL) were added. To the anodic compartment, iodotoluene (1 eq., 262 mg, 1.2 mmol) was then added. Each compartment was then capped and wrapped in parafilm. A platinum electrode was inserted into each compartment, and the reaction was subjected to electrolysis (13.4 mA, 3 F/mol, 7.2 hrs). The electrodes were then removed, 3-nitrostyrene (1 eq., 1.2 mmol, 167 μL) was then added to the anodic compartment and then caps were wrapped in parafilm to seal the reaction. After stirring overnight, the anodic compartment was quenched with 75 mL of cold (0 °C) saturated aqueous $CaCO_3$ solution. This stirred for 1 hour until the aqueous layer measured pH 7. The mixture was extracted into CH_2Cl_2, dried with Na_2SO_4 and evaporated *en vacuo*. The product was then isolated via column chromatography. This product, **3ai**, was purified using silica gel chromatography (5% EtOAc:Hexane) to yield a viscous orange oil (100 mg, 45 %).

R_f = 0.25 (5% EtOAc:Hexane)

^{1}H NMR (500 MHz, $CDCl_3$): δ = 8.26 – 8.25 (2H, m, H^{Ar}), 7.74 – 7.72 (1H, m, H^{Ar}), 7.64 – 7.61 (1H, m, H^{Ar}), 5.79 (1H, ddt, *J* = 46.8, 17.9, 4.6 Hz, H^7), 4.76 – 4.61 (2H, m, H^8)

^{13}C NMR (151 MHz, $CDCl_3$): δ = 148.6 (1C, C^4), 137.1 (1C, dd, *J* = 21.1, 6.7 Hz, C^6), 132.0 (1C, d, *J* = 6.9 Hz, C^5), 130.0 (1C, C^3), 124.2 (1C, d, *J* = 1.2 Hz, C^2), 121.2 (1C, d, *J* = 8.0 Hz, C^1), 90.9 (1C, dd, *J* = 179.5, 20.5 Hz, C^7), 84.1 (1C, dd, *J* = 179.9, 25.0 Hz, C^8).

^{19}F NMR (376 MHz, $CDCl_3$): δ = -188.46 - -188.75 (1F, m, F^7), -226.37 (1F, tdd, *J* = 46.4, 17.8, 14.7 Hz, F^8)

Data are in agreement with that previously reported.[17]

1-(1,2-difluoro-2-methylpropyl)-4-nitrobenzene, 3aj

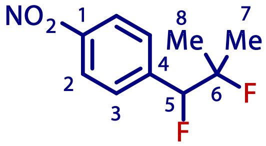

This product, **3aj**, was synthesised from **2aj** using **'ex-cell' procedure 3** and purified using silica gel chromatography (20% EtOAc:Hexane) to yield a colourless oil (206 mg, 80 %).

R_f = 0.3 (20% EtOAc:Hexane

^{1}H NMR (500 MHz, CDCl3): δ = 8.25 (1H, d, *J* = 8.7 Hz, *H^2*), 7.56 (1H, d, *J* = 8.4 Hz, *H^3*), 5.40 (1H, dd, *J* = 45.1, 12.2 Hz, *H^5*), 1.44 (3H, d, *J* = 21.7 Hz, *$H^{7,8}$*), 1.33 (3H, d, *J* = 21.7 Hz, *$H^{7,8}$*).

^{13}C NMR (151 MHz, $CDCl_3$): δ = 148.3 (1C, *C^1*), 142.8 (1C, dd, *J* = 21.2, 1.6 Hz, *C^4*), 128.0 (1C, dd, *J* = 8.3, 2.3 Hz, *C^3*), 123.4 (1C, *C^2*), 95.4 (1C, dd, *J* = 182.4, 27.5 Hz, *C^5*), 94.5 (1C, dd, *J* = 174.4, 24.4 Hz, *C^6*), 23.0 (2C, ddd, *J* = 187.6, 23.7, 2.9 Hz, *$C^{7,8}$*).

^{19}F NMR (376 MHz, $CDCl_3$): δ = - 150.98 - -151.41 (1F, m, *F^5*), -189.23 (1F, dd, *J* = 46.3, 8.6 Hz, *F^6*).

Data are in agreement with that previously reported.[9]

1-(1,2-difluoro-2-methylpropyl)-4-fluorobenzene, 3ak

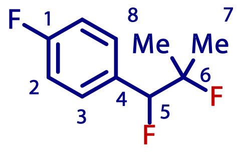

This product, **3ak**, was synthesised from **2ak** using **'ex-cell' procedure 3** and was purified using silica gel chromatography (100% Hexane) to yield a volatile, colourless oil (188 mg, 83 %).

R_f = 0.3 (Hexane)

^{1}H NMR (500 MHz, $CDCl_3$): δ = 7.39 – 7.34 (2H, m, H^2), 7.10 (2H, t, J = 8.3 Hz, H^3), 5.31 (1H, dd, J = 45.6, 14.2 Hz, H^5), 1.40 (3H, dd, J = 15.2, 1.9 Hz, H^7), 1.36 (3H, dd, J = 15.2, 1.9 Hz, H^8).

^{13}C NMR (125 MHz, $CDCl_3$): δ = 163.1 (1C, dd, J = 247.3, 1.8 Hz, C^1), 131.6 (1C, dt, J = 21.5, 3.2 Hz, C^4), 129.0 (2C, td, J = 7.9, 2.1 Hz, C^3), 115.3 (2C, d, J = 21.6 Hz, C^2), 96.2 (1C, dd, J = 180.4, 26.2 Hz, C^5), 94.9 (1C, dd, J = 172.5, 24.9 Hz, C^6), 23.0 (1C, dd, J = 23.5, 3.5 Hz, C^7), 22.8 (1C, dd, J = 23.5, 3.5 Hz, C^8).

^{19}F NMR (376 MHz, $CDCl_3$): δ = -112.94 – -113.06 (1F, m, F^1), -150.92 – -151.42 (1F, m, F^5), -186.22 (1F, dd, J = 44.9, 10.8 Hz, F^6).

HRMS (EI$^+$) calc: [$M^{+\bullet}$] ($C_{10}H_{11}F_3$) 188.0807; measured: 188.0805 = 1.06 ppm difference

IR (neat) ν_{max}/cm^{-1}: 1604, 1512, 1405, 1393, 1228, 1075, 1050, 891

1-(1,2-difluoro-2-methylpropyl)-3,5-bis(trifluoromethyl)benzene, 3al

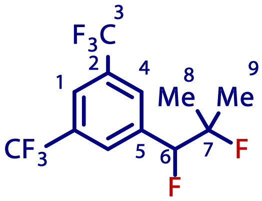

This product, **3al**, was synthesised from **2al** using **'ex-cell' procedure 3**. Purified using silica gel chromatography (100% Hexane) to yield a volatile, colourless oil (293 mg, 80 %).

R_f = 0.3 (Hexane)

^{1}H NMR (500 MHz, $CDCl_3$): δ = 7.93 (1H, s, *H^1*), 7.86 (2H, s, *H^4*), 5.44 (1H, dd, *J* = 44.6, 12.1 Hz, *H^6*), 1.48 (1H, dd, *J* = 21.7, 1.9 Hz, *$H^{8,9}$*), 1.37 (1H, dd, *J* = 21.6, 1.3 Hz, *$H^{8,9}$*).

^{13}C NMR (126 MHz, $CDCl_3$): δ = 138.4 (1C, dd, *J* = 21.9, 1.0 Hz, *C^5*), 131.8 (2C, q, *J* = 33.6 Hz, *C^2*), 127.3 (2C, s, *C^4*), 123.3 (1C, q, *J* = 272.7 Hz, *C^3*) 122.8 (1C, m, *C^1*), 95.1 (1C, dd, *J* = 182.8, 27.8 Hz, *C^6*), 94.2 (1C, dd, *J* = 174.2, 24.6 Hz, *C^7*), 23.9 (1C, dd, *J* = 23.7, 2.8 Hz, *$C^{8,9}$*), 22.0 (1C, dd, *J* = 23.9, 2.9 Hz, *$C^{8,9}$*).

^{19}F NMR (376 MHz, $CDCl_3$): δ = -62.84 (1F, s, *F^3*), -152.02 – -152.44 (1F, m, *F^6*), -189.20 (1F, dd, *J* = 44.7, 8.9 Hz, *F^7*)

HRMS (EI^+) calc: [M-F^+] ($C_{12}H_{10}F_7$) 287.0665; measured: 287.0663 = 0.70 ppm difference

IR (neat) v_{max}/cm^{-1}: 1517, 1462, 1318, 1018, 965, 850

N-(2,3-difluoropropyl)-4-methyl-N-(4-methylbenzyl)benzenesulfonamide, 3am

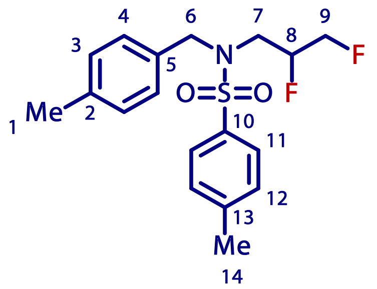

This product, **3am**, was synthesised from **2am** using **'ex-cell' procedure 3** and purified using silica gel chromatography (10% EtOAc:Hexane) to yield a viscous, colourless oil (270 mg, 60 %).

R_f = 0.2 (10% EtOAc:Hexane)

^{1}H NMR (500 MHz, $CDCl_3$): δ = 7.73 (2H, d, *J* = 8.7 Hz, H^{11}), 7.34 (2H, d, *J* = 8.7 Hz, H^{12}), 7.13 – 7.03 (4H, m, H^{Ar}), 4.76 – 4.23 (5H, m, $H^{8,9,6}$), 3.49 – 3.25 (2H, m, H^7), 2.46 (3H, s, $H^{1,14}$), 2.33 (3H, s, $H^{1,14}$)

^{13}C NMR (126 MHz, $CDCl_3$): δ = 144.0 (1C, C^{13}), 138.1 (1C, C^5), 136.4 (1C, C^{10}), 132.4 (1C, C^5), 130.0 (2C, C^{11}), 129.6 (2C, C^3), 128.8 (2C, C^4), 127.5 (2C, C^{12}), 90.4 (1C, dd, *J* = 176.4, 18.1 Hz, C^8), 82.3 (1C, dd, *J* = 174.4, 21.5 Hz, C^8), 53.2 (1C, C^6), 46.9 (1C, dd, *J* = 25.4, 7.6 Hz, C^7), 21.7 (1C, C^{14}), 21.3 (1C, C^1).

^{19}F NMR (376 MHz, $CDCl_3$): δ = -191.15 – -191.54 (1F, m, F^8), -234.22 (1F, tdd, *J* = 47.5, 23.9, 12.4 Hz, F^9)

HRMS (ESI$^+$) calc: $[M+Na]^+$ ($C_{18}H_{21}F_2NO_2SNa$) 376.1153; measured: 376.1141 = 3.19 ppm difference.

IR (neat) v_{max}/cm^{-1}: 1597, 1514, 1450, 1336, 1157, 1089, 1016, 950, 811,

N-(4-chlorobenzyl)-N-(2,3-difluoro-2-methylpropyl)-4-methylbenzenesulfonamide, 3an

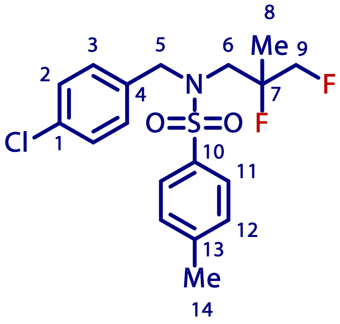

This product, **3an**, was synthesised from **2an** using **'ex-cell' procedure 3** and purified using silica gel chromatography (10% EtOAc:Hexane) to yield a colourless oil (275 mg, 60 %).

R_f = 0.3 (10% EtOAc:Hexane)

^{1}H NMR (500 MHz, $CDCl_3$): δ = 7.68 (d, *J* = 8.3 Hz, 2H), 7.31 (2H, d, *J* = 8.4 Hz, H^{Ar}), 7.20 (d, *J* = 8.5 Hz, 2H), 7.02 (d, *J* = 8.4 Hz, 2H), 4.44 (2H, d, *J* = 3.7 Hz, H^5), 4.35 (dd, *J* = 18.5, 1.8 Hz, 1H), 3.49 – 3.43 (m, 2H), 2.45 (s, 3H), 1.34 (dd, *J* = 21.9, 2.3 Hz, 3H).

^{13}C NMR (126 MHz, $CDCl_3$): δ = 144.0 (1C, C^{13}), 137.2 (1C, C^{10}), 134.1 (1C, C^4), 133.8 (1C, C^1), 130.4 (2C, C^2), 130.0 (2C, C^{11}), 128.7 (2C, C^3), 127.4 (2C, C^{12}), 96.4 (1C, dd, *J* = 175.0, 18.1 Hz, C^7), 85.3 (1C, dd, *J* = 177.0, 27.1 Hz, C^9), 52.4 (1C, C^5), 51.5 (1C, dd, *J* = 22.6, 5.3 Hz, C^6), 21.7 (1C, C^{14}), 19.2 (1C, dd, *J* = 23.4, 4.7 Hz, C^8).

^{19}F NMR (376 MHz, $CDCl_3$): δ = -155.31 – -155.73 (1F, m, F^7), -231.47 (1F, dt, *J* = 47.5, 13.4 Hz, F^9).

HRMS (ESI) calc: $[M+Na]^+$ ($C_{18}H_{20}ClF_2NO_2SNa$) 410.0769; measured 410.0772 = 1.7 ppm difference

IR (neat) v_{max}/cm^{-1}: 1612, 1495, 1356, 1159, 1058, 991, 846

N-(4-chlorobenzyl),N-2,3-difluorobutyl)-4-methylbenzenesulfonamide, 3ao

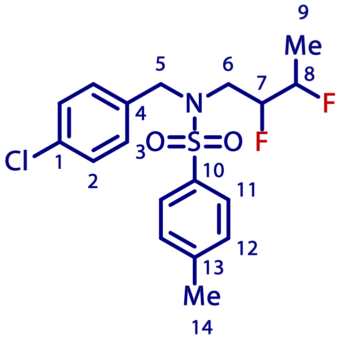

This product, **3ao**, was synthesised from **2ao** using **'ex-cell' procedure 3** and purified using silica gel chromatography (5% EtOAc:Hexane) to yield a colourless oil (255 mg, 55 %).

R_f = 0.15 (5% EtOAc:Hexane)

^{1}H NMR (500 MHz, $CDCl_3$): Major diastereomer δ = 7.72 (2H, d, *J* = 8.4 Hz, H^{11}), 7.39 – 7.26 (4H, m, $H^{2,3}$), 7.19 (2H, d, *J* = 8.4 Hz, H^{12}), 4.77 – 4.28 (4H, m, $H^{7,8,5}$), 3.60 (1H, ddd, *J* = 35.4, 15.9, 2.7 Hz, H^{6a}), 3.20 (1H, td, *J* = 14.8, 8.5 Hz, H^{6b}), 2.44 (3H, s, H^{14}), 1.40 – 1.25 (3H, m, H^{9}). Minor diastereomer δ = 7.71 (2H, d, *J* = 8.4 Hz, H^{11}), 7.39 – 7.26 (4H, m, $H^{2,3}$), 7.16 (2H, d, *J* = 8.4 Hz, H^{12}), 4.77 – 4.28 (4H, m, $H^{7,8,5}$), 3.51 (1H, ddd, *J* = 30.0, 15.6, 3.7 Hz, H^{6a}), 3.31 (1H, td, *J* = 14.3, 7.3 Hz, H^{6b}), 2.44 (3H, s, H^{14}), 1.40 – 1.25 (3H, m, H^{9}).

^{13}C NMR (126 MHz, $CDCl_3$): Major diastereomer δ = 144.0 (1C, C^{13}), 136.8 (1C, C^{1}), 134.3 (1C, C^{4}), 134.0 (1C, C^{10}), 130.0 (2C, C^{11}), 130.0 (2C, C^{3}), 128.9 (2C, C^{2}), 127.3 (2C, C^{12}), 93.3 (1C, dd, *J* = 179.3, 22.3 Hz, C^{7}), 89.7 (1C, dd, *J* = 171.6, 24.3 Hz, C^{8}), 52.2 (1C, d, *J* = 1.7 Hz, C^{5}), 47.2 (1C, dd, *J* = 24.7, 7.5 Hz, C^{6}), 21.7 (1C, C^{14}), 16.0 (1C, dd, *J* = 21.9, 6.0 Hz, C^{9}). Minor diastereomer δ = 144.0 (1C, C^{13}), 136.6 (1C, C^{1}), 134.3 (1C, C^{4}), 134.0 (1C, C^{10}), 130.0 (4C, C^{3}), 128.9 (2C, C^{2}), 127.4 (2C, C^{12}), 93.0 (1C, dd, *J* = 182.3, 29.2 Hz, C^{7}), 88.3 (1C, dd, *J* = 174.6, 20.0 Hz, C^{8}), 52.5 (1C, d, *J* = 1.7 Hz, C^{5}), 48.0 (1C, dd, *J* = 21.9, 6.9 Hz, C^{6}), 21.7 (1C, C^{14}), 16.1 (1C, dd, *J* = 23.4, 5.9 Hz, C^{9}).

^{19}F NMR (376 MHz, $CDCl_3$): Major diastereomer δ = -187.07 – -187.50 (1F, m, F^7), -191.48 – -191.86 (1F, m, F^8). Minor diastereomer δ = -192.53 – -192.97 (1F, m, F^7), -200.95 – -201.31 (1F, m F^8).

HRMS (ESI) calc: [M+H$^+$] ($C_{18}H_{21}ClF_2NO_2S$) 388.0944; measured: 388.0939 = 1.54 ppm difference

IR (neat) ν_{max}/cm^{-1}: 1597, 1491, 1338, 1157, 1090, 1013, 986, 918, 803

N-(4-chlorobenzyl)-N-(2,3-difluoro-3-methylbutyl)-4-methylbenzenesulfonamide, 3ap

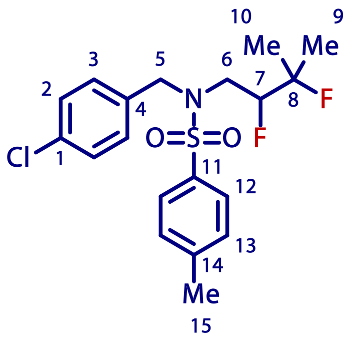

This product, **3ap**, was synthesised from **2ap** using **'ex-cell' procedure 3** and purified using silica gel chromatography (5% EtOAc:Hexane) to yield a colourless oil (246 mg, 50 %).

R_f = 0.15 (5% EtOAc:Hexane)

^{1}H NMR (500 MHz, $CDCl_3$): δ = 7.72 (2H, d, *J* = 8.4 Hz, H^{11}), 7.37 – 7.26 (4H, m, $H^{2,3}$), 7.21 – 7.17 (2H, m, H^{12}), 4.52 – 4.26 (3H, m, $H^{5,7}$), 3.81 – 3.57 (1H, m, H^{6a}), 3.31 – 3.11 (1H, m, H^{6b}), 2.45 (3H, s, H^{15}), 1.36 – 1.23 (6H, m, $H^{9,10}$).

^{13}C NMR (126 MHz, $CDCl_3$): δ = 143.9 (1C, C^{14}), 137.1 (1C, C^1), 134.4 (1C, C^4), 133.9 (1C, C^{11}), 130.0 (2C, C^{13}), 130.0 (2C, C^3), 128.9 (2C, C^2), 127.3 (2C, C^{13}), 95.6 (1C, dd, *J* = 182.4, 24.5 Hz, C^7), 94.2 (1C, dd, *J* = 169.8, 21.4 Hz, C^8), 51.9 (1C, s, C^5), 46.8 (1C, dd, *J* = 21.4, 6.4 Hz, C^6), 23.6 (1C, dd, *J* = 23.7, 3.4 Hz, $C^{9\ or\ 10}$), 22.3 (1C, dd, *J* = 24.3, 5.6 Hz, $C^{9\ or\ 10}$), 21.7 (1C, s, C^{15}).

^{19}F NMR (376 MHz, $CDCl_3$): δ = -152.94 – -153.38 (1F, m, F^7), -191.08 – -191.43 (1F, m, F^8).

HRMS (ESI) calc: [M+H$^+$] ($C_{19}H_{21}ClF_2NO_2S$) 402.1101; measured: 402.1095 = 1.49 ppm difference

IR (neat) v_{max}/cm^{-1}: 1597, 1491, 1338, 1157, 1089, 1011, 918, 805

(±) *N,N-dibenzyl-3,4-difluorohexan-1-amine, 3aq*

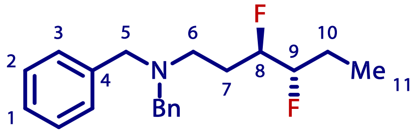

This product, **3aq**, was synthesised from **2aq** using **'ex-cell' procedure 3** and purified using silica gel chromatography (20% EtOAc:Hexane) to yield a viscous orange oil (229 mg, 60 %).

R_f = 0.2 (20% EtOAc:Hexane)

^{1}H NMR (500 MHz, $CDCl_3$): δ = 7.43 – 7.26 (10H, m, H^{Ar}), 4.73 – 4.28 (2H, m, $H^{8,9}$), 3.75 – 3.50 (4H, m, H^5), 2.72 – 2.47 (2H, m, H^7), 1.91 – 1.61 (4H, m, $H^{7,10}$), 1.03 (3H, t, *J = 7.1 Hz*, H^{11}).

^{13}C NMR (126 MHz, $CDCl_3$): δ = 139.6 (2C, C^1), 128.9 (4C, C^{Ar}), 128.4 (4C, C^{Ar}), 127.1 (1C, *C4*), 95.1 (1C, dd, *J* = 173.3, 23.7 Hz, C^9), 91.9 (1C, dd, *J* = 173.3, 23.7 Hz, C^9), 58.6 (2C, C^5), 49.4 (1C, d, *J* = 3.5 Hz, C^6), 28.2 (1C, dd, *J* = 22.3, 4.4 Hz, C^7), 23.5 (1C, dd, *J* = 21.6, 5.7 Hz, C^{10}), 9.5 (1C, d, *J* = 4.5 Hz, C^{11}).

^{19}F NMR (376 MHz, $CDCl_3$): δ = -192.35 – -192.81 (1F, m, F^8), -194.02 – -194.42 (1F, m, F^9). $^3J_{FF}$ = 14.6 Hz.

HRMS (ESI) Calc: [M+H$^+$] ($C_{20}H_{25}F_2N$) 317.1955; measured: 317.1949 = 2.7 ppm difference

IR (neat) v_{max}/cm^{-1}: 1351, 1114, 1096, 853, 711, 651

(±) *N,N-dibenzyl-3,4-difluorohexan-1-amine, 3ar*

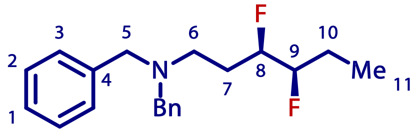

This product, **3ar**, was synthesised from **2ar** using **'ex-cell' procedure 3** and purified using silica gel chromatography (20% EtOAc:Hexane) to yield a viscous orange oil (217 mg, 57%).

R_f = 0.2 (20% EtOAc:Hexane)

^{1}H NMR (500 MHz, $CDCl_3$): δ = 7.39 – 7.23 (10H, m, H^{Ar}), 4.65 – 4.46 (1H, m, H^8), 4.20 – 4.00 (1H, m, H^9), 3.64 – 3.53 (4H, app q, H^5), 2.66 – 2.56 (2H, m, H^7), 2.03 – 1.50 (4H, m, $H^{7,10}$), 0.97 (3H, t, *J = 7.1 Hz*, H^{11}).

^{13}C NMR (126 MHz, $CDCl_3$): δ = 139.6 (2C, C^1), 129.0 (4C, C^{Ar}), 128.4 (4C, C^{Ar}), 127.1 (1C, *C4*), 94.3 (1C, dd, *J* = 174.9, 21.3 Hz, C^9), 91.5 (1C, dd, *J* = 174.9, 20.1 Hz, C^9), 58.7 (2C, C^5), 49.3 (1C, d, *J* = 5.6 Hz, C^6), 28.4 (1C, dd, *J* = 20.9, 5.0 Hz, C^7), 23.7 (1C, dd, *J* = 21.6, 5.7 Hz, C^{10}), 9.5 (1C, d, *J* = 6.1 Hz, C^{11}).

^{19}F NMR (376 MHz, $CDCl_3$): δ = -197.68 – -198.15 (1F, m, F^8), -200.56 – -200.96 (1F, m, F^9). $^3J_{FF}$ = 10.3 Hz.

HRMS (ESI) Calc: [M+H$^+$] ($C_{20}H_{25}F_2N$) 317.1955; measured: 317.1951 = 1.6 ppm difference

IR (neat) ν_{max}/cm^{-1}: 1352, 1114, 1098, 855, 712, 654

1-((2,3-difluoropropoxy)methyl)-2-methylbenzene, 3as

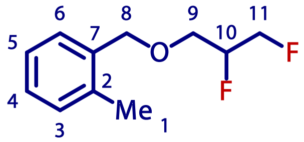

This product, **3as**, was synthesised from **2as** using **'in-cell' procedure 2** and purified using silica gel chromatography (5% Et_2O:Hexane) to yield a volatile, colourless oil (167 mg, 65%).

R_f = 0.3 (5% Et_2O:Hexane)

^{1}H NMR (500 MHz, $CDCl_3$): δ = 7.30 - 7.27 (1H, m, H^{Ar}), 7.25 - 7.18 (3H, m, H^{Ar}), 4.93 – 4.74 (1H, m, H^{10}), 4.71 – 4.54 (4H, m, $H^{8,11}$), 3.73 (2H, ddd, J = 19.9, 5.0, 1.4 Hz, H^9), 2.34 (3H, s, H^1).

^{13}C NMR (126 MHz, $CDCl_3$): δ = 137.0 (1C, C^2), 135.4 (1C, C^7), 130.5 (1C, C^{Ar}), 128.9 (1C, C^{Ar}), 128.3 (1C, C^{Ar}), 126.0 (1C, C^{Ar}), 90.5 (1C, dd, J = 175.5, 19.8 Hz, C^{10}), 82.3 (1C, dd, J = 172.3, 23.3 Hz, C^{11}), 72.4 (1C, C^8), 68.0 (1C, dd, J = 24.4, 8.0 Hz, C^9), 18.9 (1C, C^1).

^{19}F NMR (376 MHz, $CDCl_3$): δ = -195.76 – -196.16 (1F, m, F^{10}), -233.61 (1F, tddt, J = 46.7, 19.9, 12.5, 2.2 Hz, F^{11})

HRMS (EI$^+$) calc: [$M^{+\bullet}$] ($C_{11}H_{14}OF_2$) 199.0929; measured: 199.0926 = 1.51 ppm difference

IR (neat) v_{max}/ cm^{-1}: 1515, 1418, 1095, 1030, 857, 745

((2,3-difluoro-2-methylpropoxy)methyl)benzene, 3at

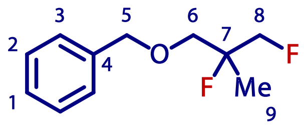

This product, **3at**, was synthesised from, **2at**, using **'ex-cell' procedure 3** and purified using silica gel chromatography (10% EtOAc:Hexane) to yield a volatile colourless oil (168 mg, 70 %).

R_f = 0.3 (10% EtOAc:Hexane)

^{1}H NMR (500 MHz, $CDCl_3$): δ = 7.38 – 7.28 (5H, m, $H^{1,2,3}$), 4.59 (2H, d, *J* = 5.5 Hz, H^5), 4.49 (2H, ddd, *J* = 47.4, 19.0, 5.5 Hz, H^8), 3.64 (1H, ddd, *J* = 14.4, 10.3, 2.0 Hz, H^{6a}), 3.54 (1H, ddd, *J* = 17.1, 10.3, 2.2 Hz, H^{6b}), 1.39 (3H, dd, *J* = 21.9, 2.2 Hz, 2H, H^9).

^{13}C NMR (126 MHz, $CDCl_3$): δ = 137.8 (1C, C^4), 128.6 (2C, $C^{2,3}$), 128.0 (1C, C^1), 127.8 (2C, $C^{2,3}$), 94.7 (1C, dd, *J* = 172.7, 18.3 Hz, C^7), 84.5 (1C, dd, *J* = 175.7, 27.0 Hz, C^8), 73.8 (1C, C^5), 71.5 (1C, dd, *J* = 27.4, 4.9 Hz, C^6), 18.6 (1C, dd, *J* = 23.2, 5.0 Hz, C^9).

^{19}F NMR (376 MHz, $CDCl_3$): δ = -162.17 – -162.56 (1F, m, F^7), -232.45 (1F, tdq, *J* = 47.6, 12.8, 2.2 Hz, F^8).

HRMS (EI^+) calc: [$M^{+\bullet}$] ($C_{11}H_{14}OF_2$) 200.1013; measured: 200.1026 = 3.89 ppm difference

IR (neat) v_{max}/ cm^{-1}: 1612, 1225, 1005, 1056, 978, 856, 845, 785

1-((2,3-difluoro-2-methylpropoxy)methyl)-4-(trifluoromethyl)benzene, 3au

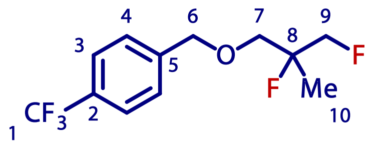

This product, **3au**, was synthesised from **2au** using **'ex-cell' procedure 3** and purified using silica gel chromatography (10% Et_2O:Hexane) to yield a volatile, colourless oil (234 mg, 73 %).

R_f = 0.2 (10% Et_2O:Hexane)

^{1}H NMR (500 MHz, $CDCl_3$): δ = 7.62 (2H, d, *J* = 7.9 Hz, H^3), 7.44 (2H, d, *J* = 7.9 Hz, H^4), 4.65 (2H, s, H^6), 4.49 (2H, dd, *J* = 47.3, 18.5 Hz, H^9), 3.72 – 3.55 (2H, m, $H^{7a.b}$), 1.41 (3H, d, *J* = 21.8 Hz, H^{10}).

^{13}C NMR (126 MHz, $CDCl_3$): δ = 142.0 (1C, C^5) , 130.1 (1C, C^4), 127.6 (2C, q, *J* = 3.8 Hz, C^3), 125.5 (1C, q, *J* = 277.1 Hz, C^1), 124.3 (2C, q, *J* = 32.4 Hz, C^2), 94.5 (1C, dd, *J* = 173.0, 18.4 Hz, C^8), 84.4 (1C, dd, *J* = 175.8, 27.4 Hz, C^9), 73.0 (1C, C^6), 71.9 (1C, dd, *J* = 27.2, 4.8 Hz, C^7), 18.6 (1C, dd, *J* = 23.1, 4.8 Hz, C^{10}).

^{19}F NMR (376 MHz, $CDCl_3$): δ = -62.43 (3F, s, F^1), -162.32 – -162.71 (1F, m, F^8), -232.20 (1F, tdq, *J* = 47.3, 11.2, 2.2 Hz, F^9).

HRMS (EI$^+$) calc: [$M^{+\bullet}$] ($C_{12}H_{13}OF_5$) 268.0881; measured: 268.0881 = 0 ppm difference

IR (neat) v_{max}/cm^{-1}: 1621, 1419, 1336, 1162, 1120, 1066, 1018, 896, 824

Faradaic efficiency calculations

Typical 'in-cell' method:

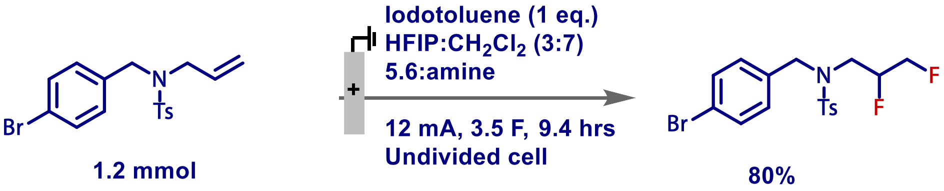

Yield of reaction = 80%

Charge required = $2\ x\ \ 96.5\ C\ mmol^{-1}\ x\ 1.2\ mmol = \mathbf{231.6\ C}$

Charge used in reaction: $3.5\ x\ \ 96.5\ C\ mmol^{-1}\ x\ 1.2\ mmol = \mathbf{405.3\ C}$

Faradaic efficiency $= \left(\frac{231.6\ C}{405.3\ C}\right)\ x\ 0.8 = \mathbf{0.46}$

Typical 'ex-cell' method:

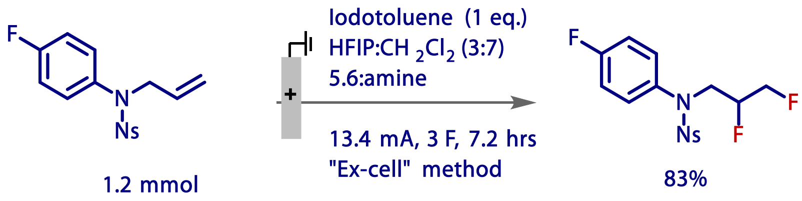

Yield of reaction = 83%

Charge required = $2\ x\ \ 96.5\ C\ mmol^{-1}\ x\ 1.2\ mmol = \mathbf{231.6\ C}$

Charge used in reaction: $3\ x\ \ 96.5\ C\ mmol^{-1}\ x\ 1.2\ mmol = \mathbf{347.4\ C}$

Faradaic efficiency $= \left(\frac{231.6\ C}{347.4\ C}\right)\ x\ 0.83 = \mathbf{0.55}$

Benchmarking experiments:

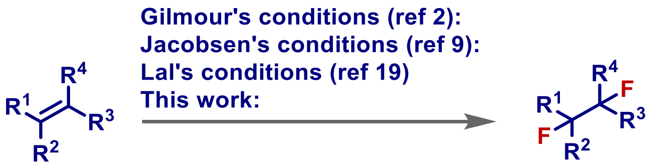

Gilmour's reaction conditions:[2]

The reaction was carried out on a 1.2 mmol scale. A 20mL HDPE Scintillation Vial reaction vessel was charged with the appropriate olefin (1.2 mmol, 1 eq.), 4-iodotoluene (52.2 mg, 0.2 mmol, 0.2 eq.) and DCE (3 mL) under an atmosphere of air. To the stirred clear solution was added the HF source (3 mL, amine : HF / 1 : 4.5) and Selectfluor® (363 mg, 1.8 mmol, 1.50 eq.) in one portion, consecutively. The reaction vessel was then sealed with a polypropylene screwcap and the reaction mixture was vigorously stirred at room temperature. After 14 h, the reaction mixture was thoroughly quenched by the addition of 14 mL of a saturated aqueous solution of $NaHCO_3$. The biphasic mixture was then extracted with DCM (3 x 10 mL) and the combined organic layers were dried over Na_2SO_4 and evaporated *en vacuo*. To the resulting crude was added a quantitative amount of internal standard (hexafluorobenzene) to determine the ^{19}F NMR yield.

Jacobsen's reaction conditions:[9]

The reaction was carried out on a 1.2 mmol scale. A 75 mL vial equipped with a stir bar was charged with the alkene substrate (1.2 mmol, 1 eq.), **catalyst A** (92.0 mg, 0.24 mmol, 0.2 eq.), and dichloromethane (23 mL). The mixture was stirred and pyridinium poly(hydrogen fluoride) (pyr.9HF, 3.14 mL, 120 mmol, 100 eq. HF) was added via plastic syringe, followed by m-chloroperbenzoic acid (mCPBA, 77% by weight, 350 mg, 1.56 mmol, 1.30 eq.) as a solid, all under an air atmosphere. The tube was sealed with a cap. After 20 hours, the reaction mixture was cooled to −78 °C and 4 M aqueous NaOH solution was added until pH = 14, and the mixture was allowed to warm to room temperature. The biphasic mixture was diluted with water (100 mL), the organic layer was separated, and the aqueous layer was extracted with ethyl acetate (3 × 50 mL). The combined organic layers were dried over anhydrous $MgSO_4$ and were evaporated *en vacuo*. To the resulting crude was added a quantitative amount of internal standard (hexafluorobenzene) to determine the ^{19}F NMR yield.

Lal's reaction conditions: [19]

A 20mL HDPE Scintillation vial was charged with the appropriate olefin (1.2 mmol, 1 equivalent), Selectfluor® (534 mg, 1.2 mmol, 1 eq.) and pyridinium poly(hydrogen fluoride (pyr.9HF, 1.2 mL, 46 mmol, 39 eq.) under an N_2 atmosphere. The reaction was allowed to stir for 72 hours and then saturated aqueous $NaHCO_3$ solution (100 mL) was added and the organic layers were extracted with ether (3 x 30 mL). This extract was dried over anhydrous $MgSO_4$, and evaporated *en vacuo*. To the resulting crude was added a quantitative amount of internal standard (hexafluorobenzene) to determine the ^{19}F NMR yield.

Substrate	Ref 2 / %	Ref 9 / %	Ref 19 / %	This work/%
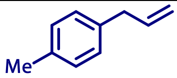 2t	41	54	/	73
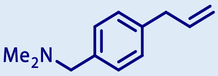 2v	<5	19	/	55
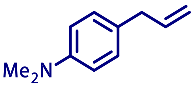 2w	/	/	<5	55
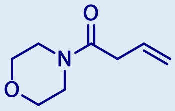 2z	<5	15	<5	45
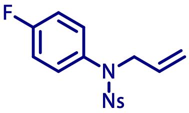 2ab	<5	33	0	85
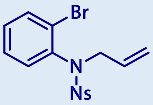 2ae	0	49	/	63
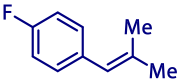 2ak	50 [17]	63	45	90
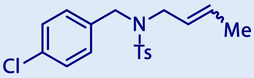 2ao	0	<5	<5	57

E-factor analysis

To ascertain the sustainability of the methods, E-factors were calculated for the generation of several products made by the electrochemical method and compared to the literature methods of Gilmour[2] and Jacobsen,[9] who use Selectfluor and mCPBA, respectively.

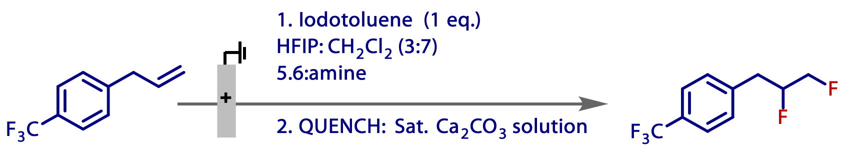

Lennox- procedure 1

Reagent	Eq.	Yield/ %	Mr/ g mol^{-1}	Moles/ mmol	Volume/ mL	Mass/ g	Waste Mass/ g
Iodotoluene	1.00		218.03	1.20		0.26	0.10
DCM			84.93		0.70	0.93	0.93
HFIP	2.38		168.04	2.86	0.30	0.48	0.48
5.6 HF:Amine	68.08				3.00	3.11	3.05
Sat. $CaCO_3$ Solution					50.00	48.88	48.88
DCM					50.00	66.5	66.50
Substrate	1.00		186.18	1.20		0.22	0.06
Product		73	224.17	0.88		0.20	
**Total waste with quench**							**120.06**
**E-Factor**							**600.00**
**Total waste without quench**							**4.62**
**E-Factor**							**23.10**

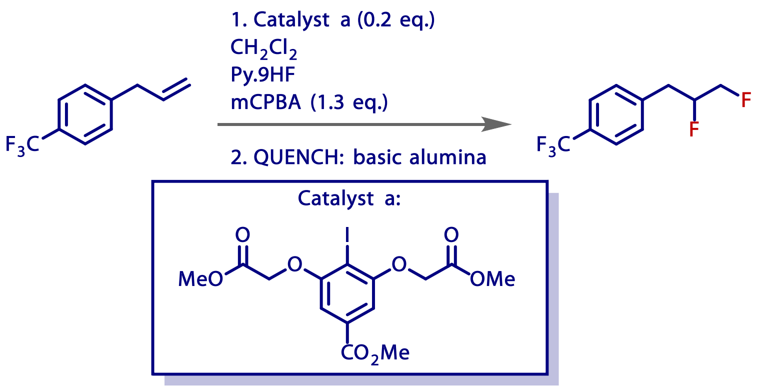

Jacobsen

Reagent	Eq.	Yield/ %	Mr/ g mol^{-1}	Moles/ mmol	Volume/ mL	Mass/ g	Waste Mass/ g
Catalyst 1b	0.20		438.17	0.21		0.09	0.09
mCPBA	1.10		172.57	1.11		0.26	0.26
DCM					20.00	26.60	26.60
Py.9HF	100.00			104	2.72	2.99	2.96
DCM					2.00	2.66	2.66
Basic Alumina						15.00	15.00
DCM					250.00	332.50	332.50
Substrate	1.00		186.18	1.04		0.19	0.076
Product		61	224.17	0.63		0.14	
**Total waste with quench**							**380.15**
**E-Factor**							**2715.33**
**Total waste without quench**							**32.65**
**E-Factor**							**233.19**

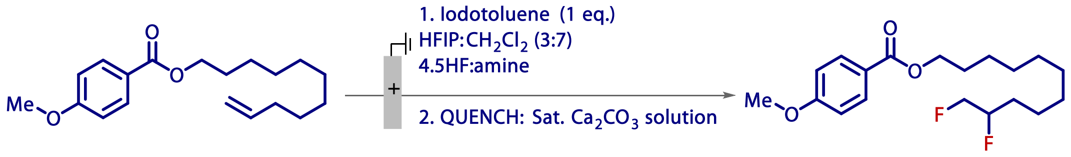

Lennox – Method X

Reagent	Eq.	Yield/ %	Mr/ g mol^{-1}	Moles/ mmol	Volume/ mL	Mass/ g	Waste Mass/ g
Iodotoluene	1.00		218.03	1.20		0.26	0.10
DCM			84.93		0.70	0.93	0.93
HFIP	2.38		168.04	2.86	0.30	0.48	0.48
4.5 HF:Amine	68				3.00	3.30	3.24
Sat. $CaCO_3$ Solution					50.00	48.88	48.88
DCM					50.00	66.50	66.50
Substrate	1.00		304.43	1.20		0.37	0.10
Product		73	342.43	0.88		0.30	
**Total waste with quench**							**120.23**
**E-Factor**							**400.6**
**Total waste without quench**							**4.85**
**E-Factor**							**16.16**

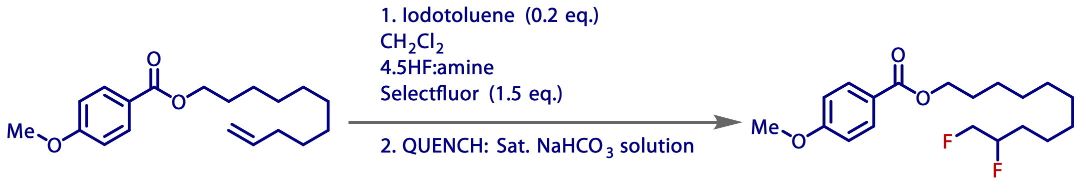

Gilmour – Method A

Reagent	Eq.	Yield/ %	Mr/ g mol^{-1}	Moles mmol	Volume / mL	Mass/ g	Waste Mass/ g
Iodotoluene	0.20		218.03	0.04		0.0087	0.0087
DCE			98.95		0.5	0.63	0.63
4.5 HF:Amine	74				0.5	0.55	0.54
Selectfluor ®	1.5			0.30		0.11	0.11
Sat. $NaHCO_3$					10	10.23	10.23
DCM						39.90	39.90
Substrate	1.00		304.43	0.20		0.061	0.015
Product		76	342.43	0.15		0.050	
**Total waste with quench**							**51.44**
**E-Factor**							**1028.67**
**Total waste without quench**							**1.31**
**E-Factor**							**26.27**

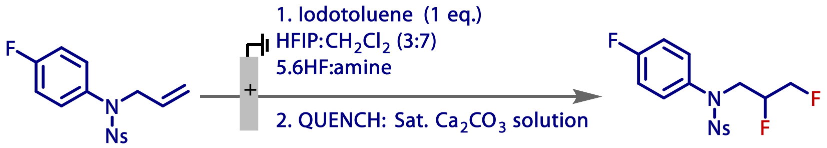

Reagent	Eq.	Yield/ %	Mr/ g mol^{-1}	Moles/ mmol	Volume/ mL	Mass/ g	Waste Mass/ g
Iodotoluene	1.00		218.03	1.20		0.26	0.10
DCM			84.93		2.1	2.79	2.79
HFIP	4.76		168.04	5.92	0.9	1.44	1.44
5.6 HF:Amine	195				9.00	9.34	9.20
Sat. $CaCO_3$ Solution					200	195.52	195.52
DCM					75	99.75	99.75
Substrate	1.00		336.34	1.20		0.40	0.06
Product		83	374.73	1.00		0.37	
**Total waste with quench**							**308.86**
**E-Factor**							**834.76**
**Total waste without quench**							**13.59**
**E-Factor**							**36.73**

For this product that uses the ex-cell method, there is no comparison published, but is calculated to assess the sustainability of this procedure.

Price of oxidant calculations

Electrochemical method: Typical 'in-cell'

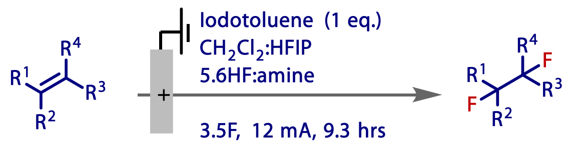

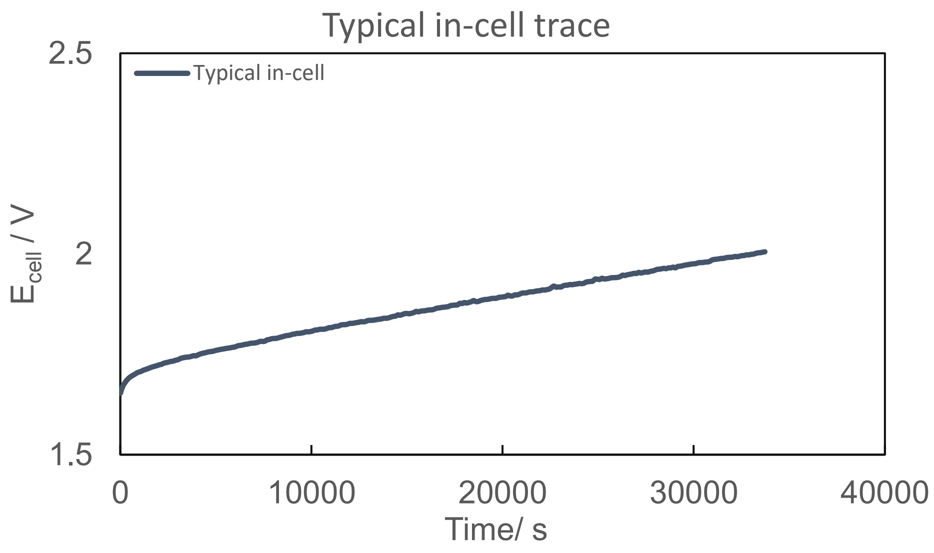

Solving for area underneath the graph gives: 62815.13 Vs

62815.13 Vs / 3600 = 17.45 Vh

17.45 Vh * 0.012 A = 0.21 Wh

0.21 Wh / 1000 = 0.00021 kWh.

Using British gas price of electricity (£0.15 per kWh):[20]:

0.00021 kWh x £0.15 per kWh = £0.000032 per 1.2 mmol

0.000032 pence per 1.2 mmol / 1.2 = £0.000027 per 1 mmol reaction

£0.000027 per 1 mmol reaction x 1000 = **£0.027 / mol**

£0.027 / mol x 1.23 (GBP to USD conversion) [21] = **$0.033 / mol**

Typical 'ex-cell' method:

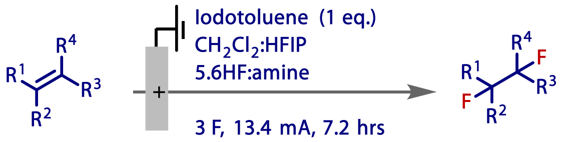

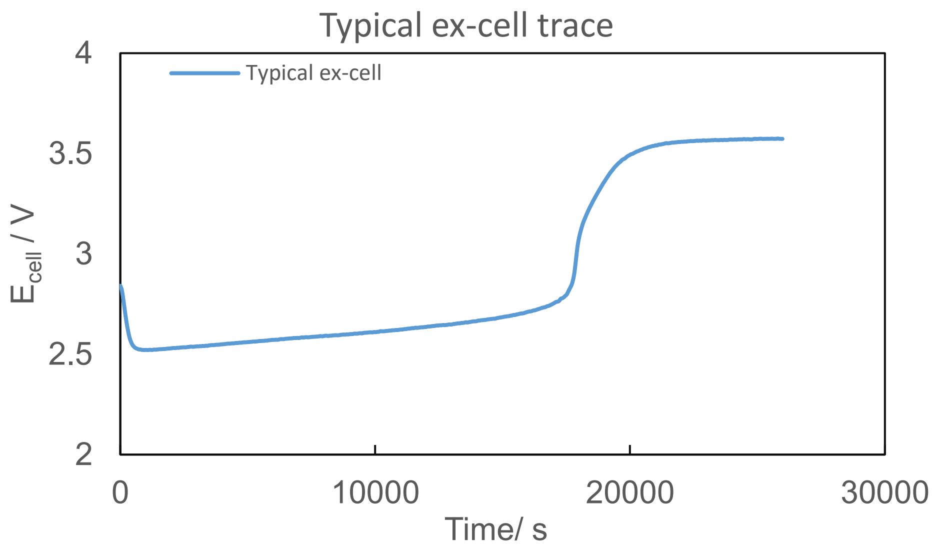

Solving for area underneath the graph gives: 75126.8 Vs

75126.8 Vs / 3600 = 20.87 Vh

20.87 Vh * 0.012 A = 0.28 Wh

0.28 Wh / 1000 = 0.00028 kWh.

Using British gas price of electricity (£0.15 pence per kWh)[20]:

0.00028 kWh x 15.260 = £0.000043 per 1.2 mmol

£0.000043 per 1.2 mmol / 1.2 = £0.000036 per 1 mmol reaction

£0.000036 per 1 mmol reaction x 1000 = **£0.036 /mol**

£0.036 / mol x 1.23 (GBP to USD conversion) [21] = **£0.043 /mol**

Selectfluor (Gilmour procedure)

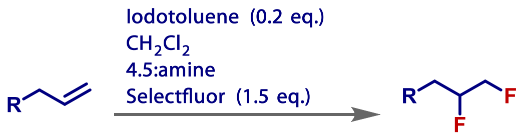

Price of selectfluor (purchased from sigma): £23.90 / 5g

For a 1.0 mol scale reaction: 1.5 eq. x 1 mol = 1.5 mol of selectfluor

1.5 mol of selectfluor = 531 mg

531 g / 5 g = 162

162 x £23.90 = **£2540 /mol**

£2540 per 1 mmol x 1.23 (GBP to USD conversion) [21] = **$3100 /mol**

mCPBA (Jacobsen procedure)

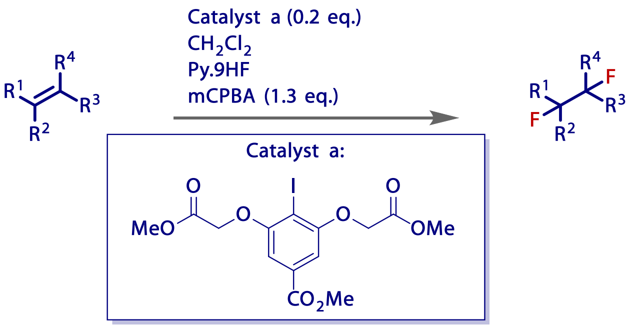

Price of mCPBA (purchased from sigma): £24.70 / 25g

For a 1 mol scale reaction: 1.3 eq. x 1 mol = 1.3 mol of mCPBA

1.3 mol of mCPBA = 292 g

292 g / 25 g = 12.00

12.00 x £24.70 = **£296 /mol**

£296 per 1 mol reaction x 1.23 (GBP to USD conversion) [21] = **$370 /mol**

Unsuccessful substrates

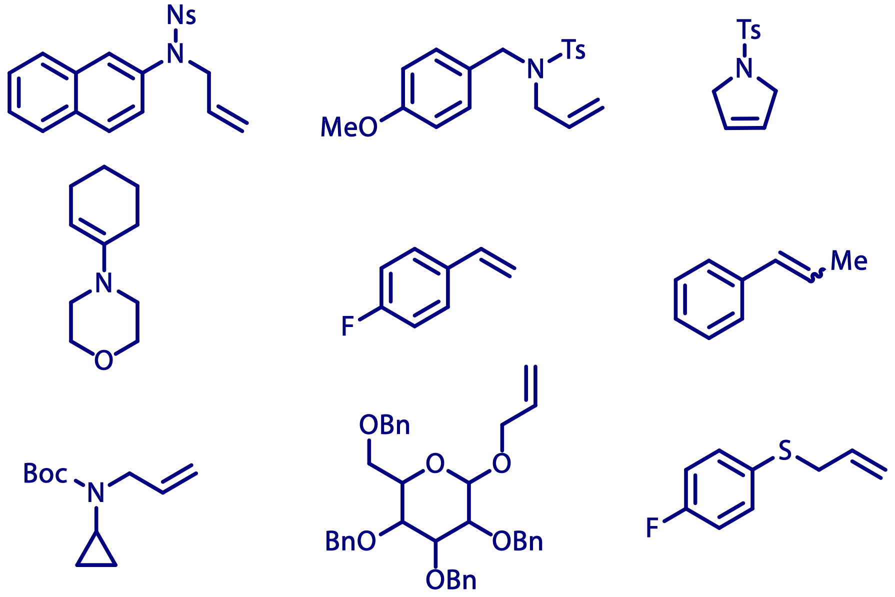

Problematic substrates included those that are known to undergo C-H activation with hypervalent iodine oxidants or can participate in single electron transfer processes. Activated styrenes either underwent gem difluorination or showed no reactivity. Additionally, acid sensitive groups, such as Boc, were not tolerated.

NMR Spectra of substrates

1-((Allyloxy)methyl)-4-nitrobenzene, 2k

^{1}H NMR (400 MHz, $CDCl_3$):

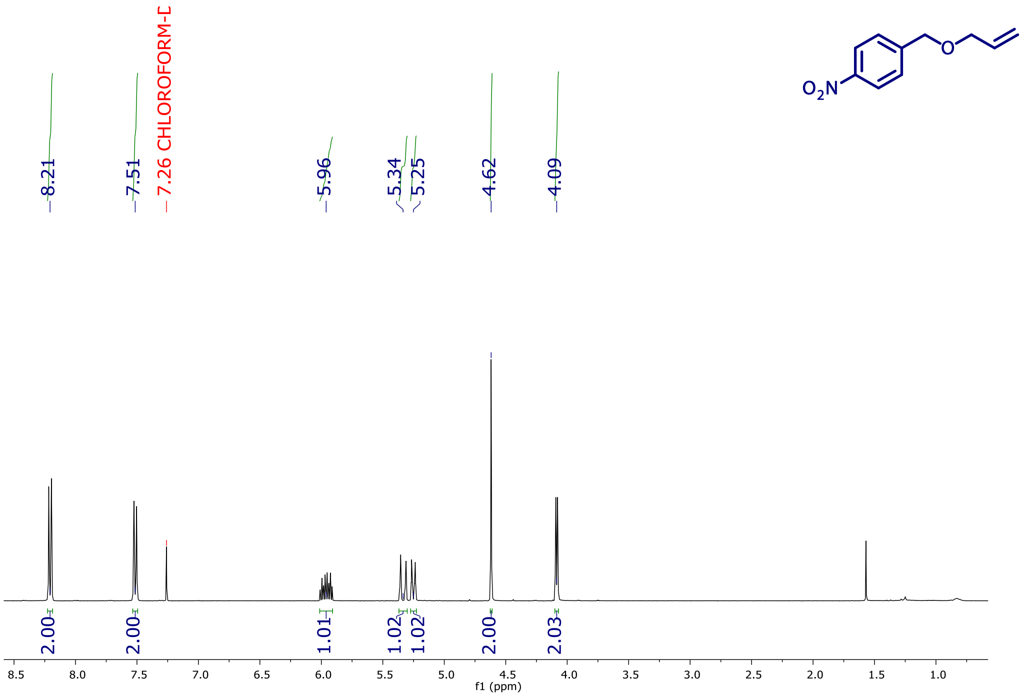

^{13}C NMR (101 MHz, $CDCl_3$):

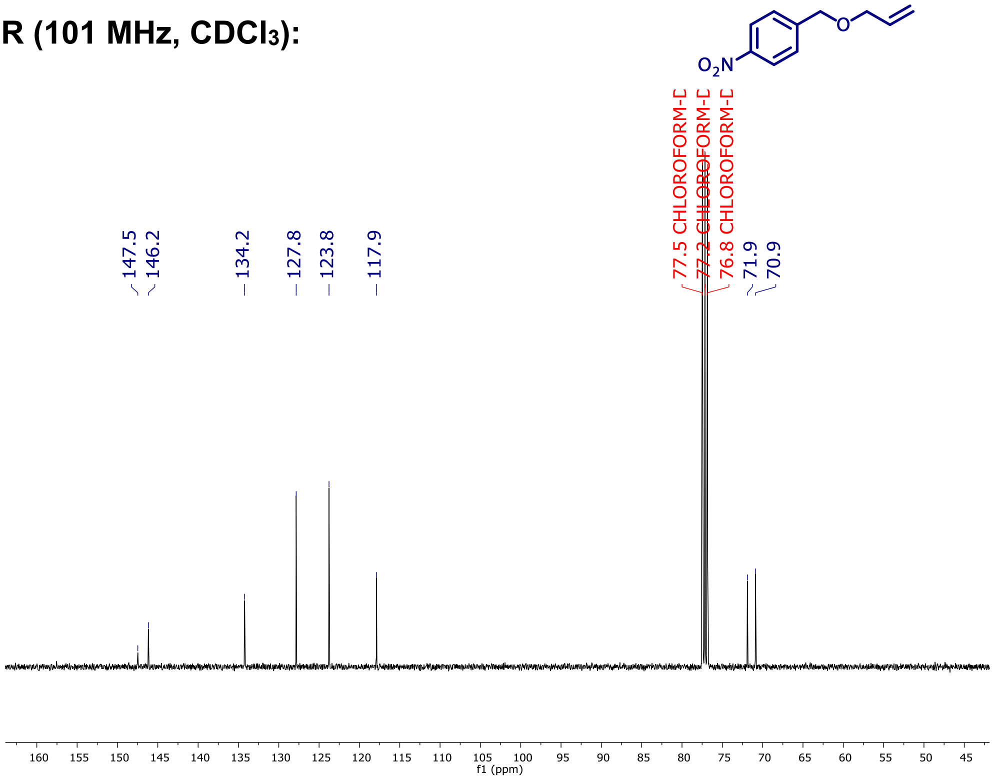

4-((Allyloxy)methyl)pyridine, 2n

^{1}H NMR (400 MHz, $CDCl_3$):

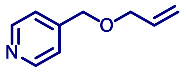

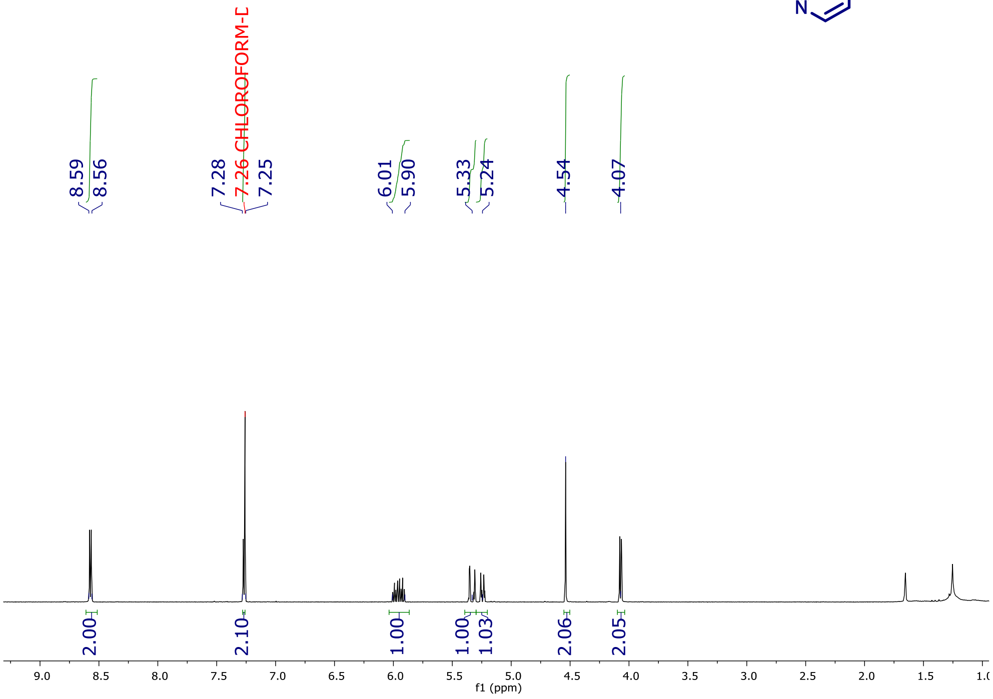

^{13}C NMR (101 MHz, $CDCl_3$):

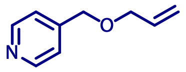

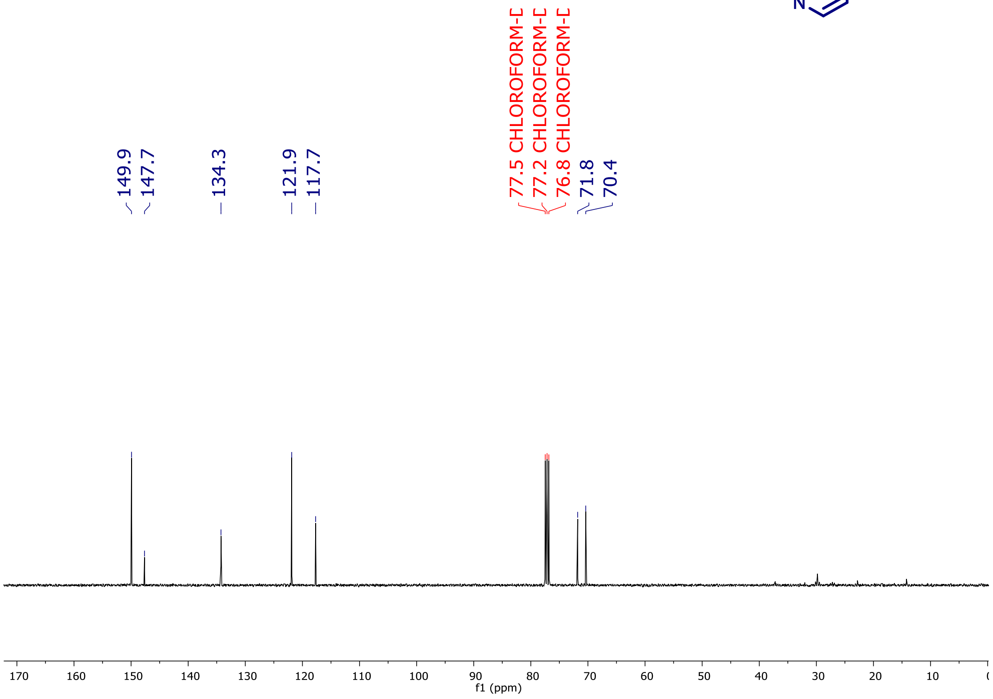

1-((Allyloxy)methyl)-3,5-dinitrobenzene, 2o

^{1}H NMR (400 MHz, $CDCl_3$):

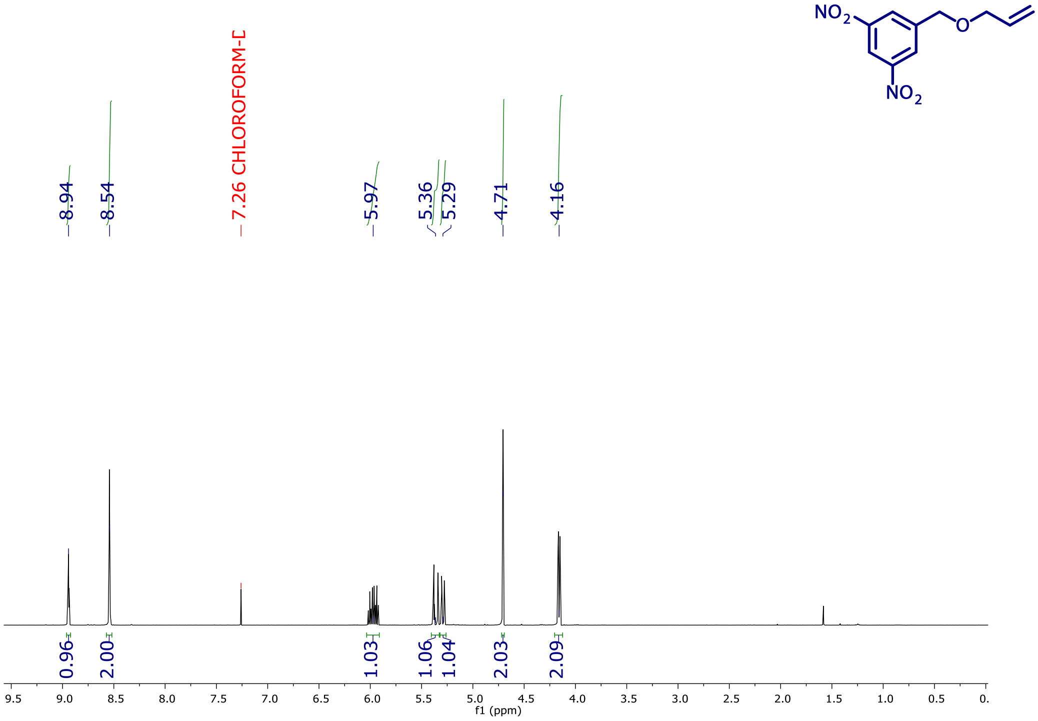

^{13}C NMR (101 MHz, $CDCl_3$):

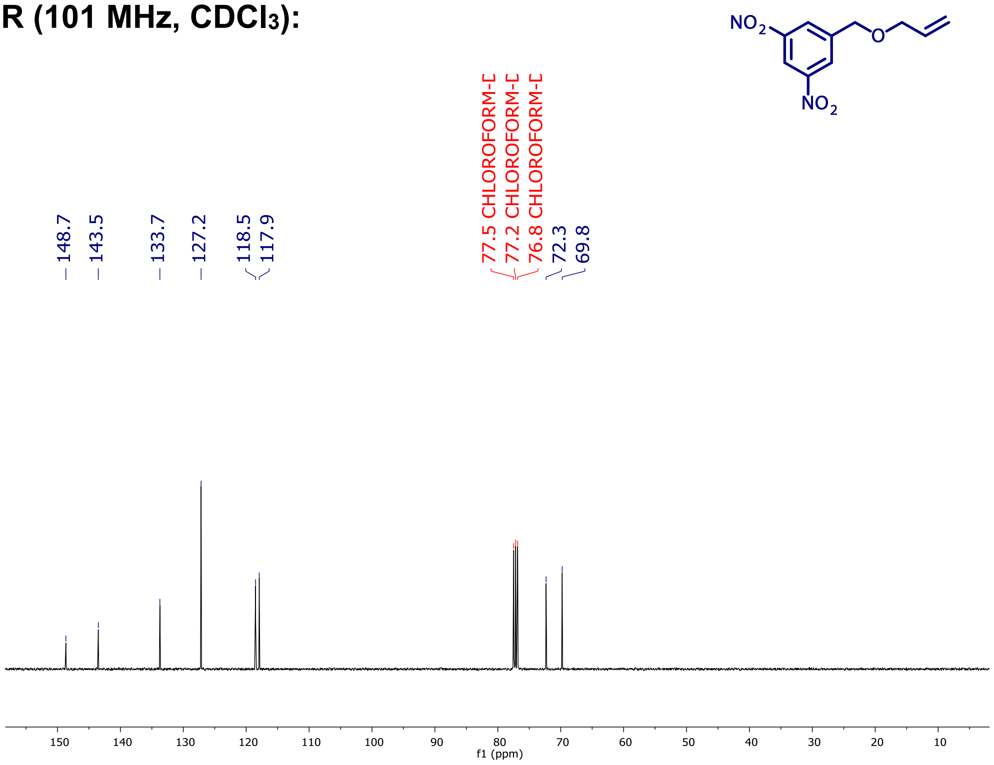

N-Allyl-N-(4-chlorobenzyl)-4-methylbenzenesulfonamide, 2q

^{1}H NMR (400 MHz, $CDCl_3$):

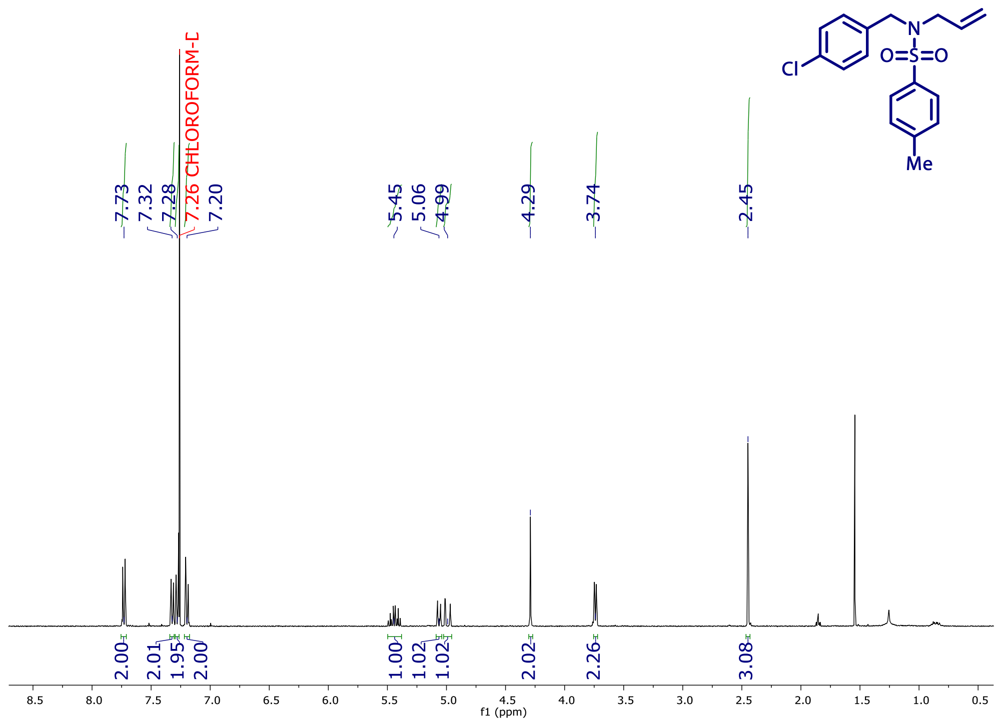

^{13}C NMR (101 MHz, $CDCl_3$):

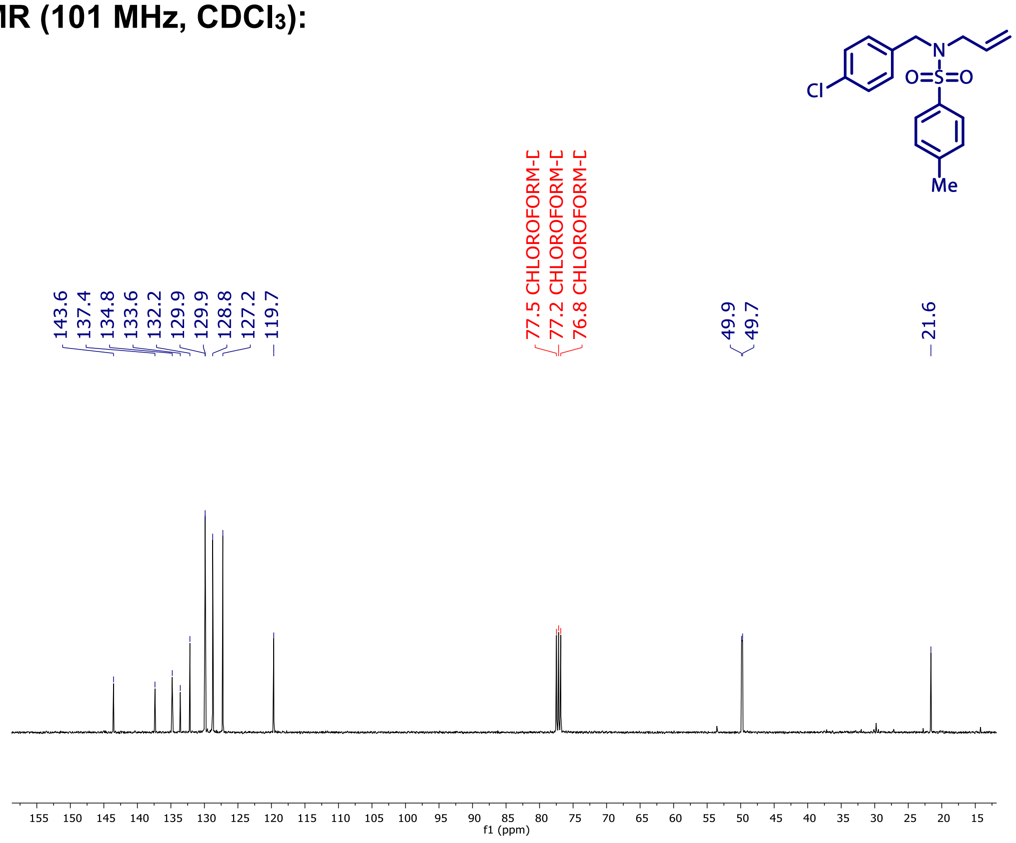

N-allyl-4-methyl-N-(4-(trifluoromethyl)benzyl)benzenesulfonamide, 2r

^{1}H NMR (400 MHz, $CDCl_3$):

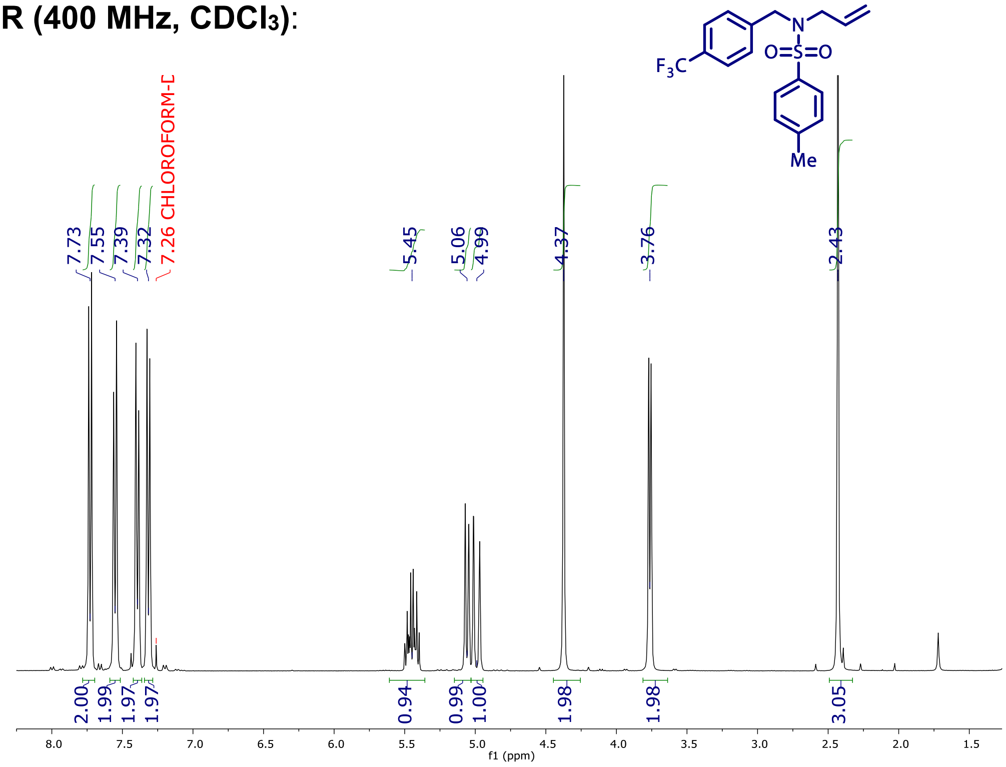

^{13}C NMR (101 MHz, $CDCl_3$):

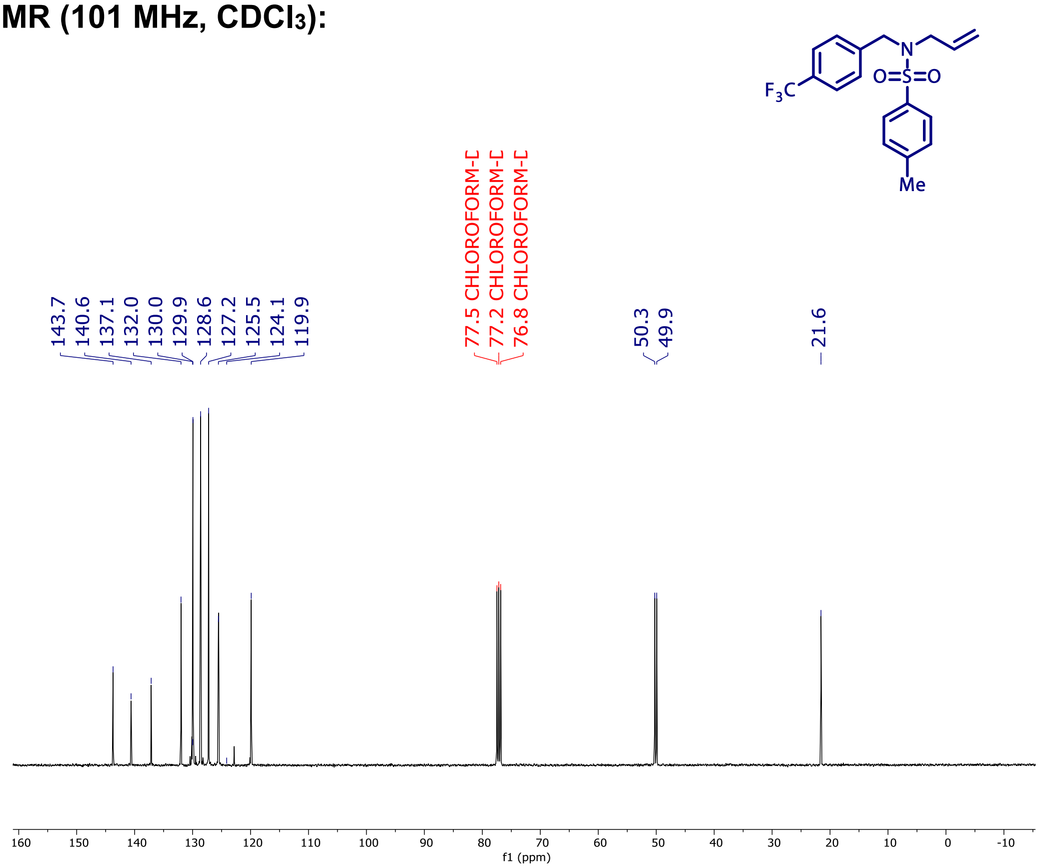

^{19}F NMR (377 MHz, $CDCl_3$):

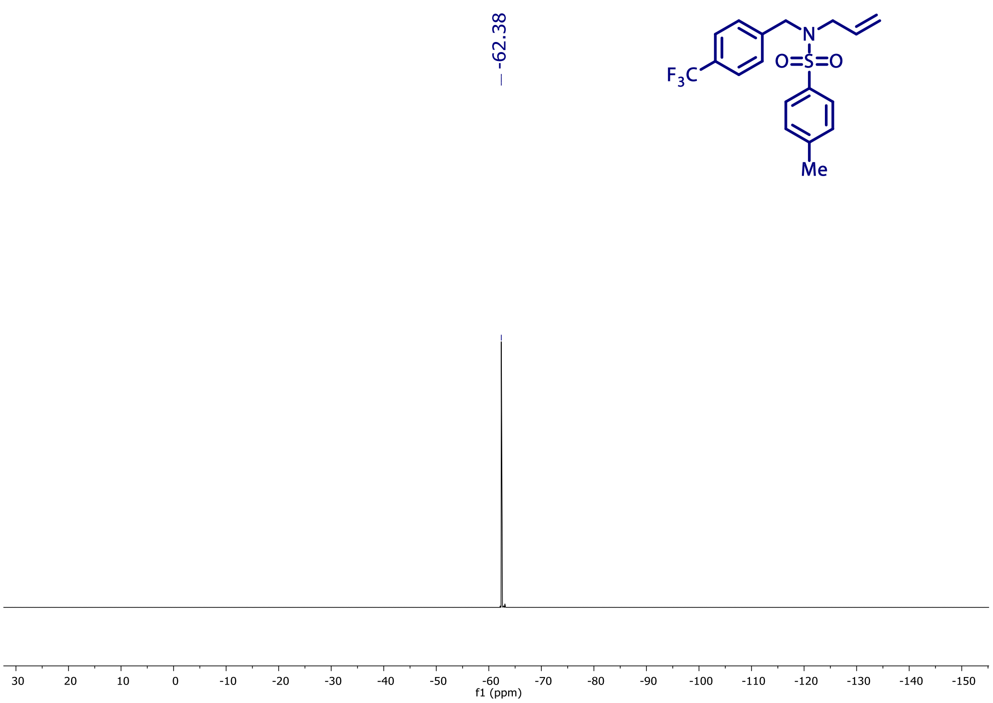

N-Allyl-N-(4-chlorobenzyl)-4-methylbenzenesulfonamide, 2s

^{1}H NMR (400 MHz, $CDCl_3$):

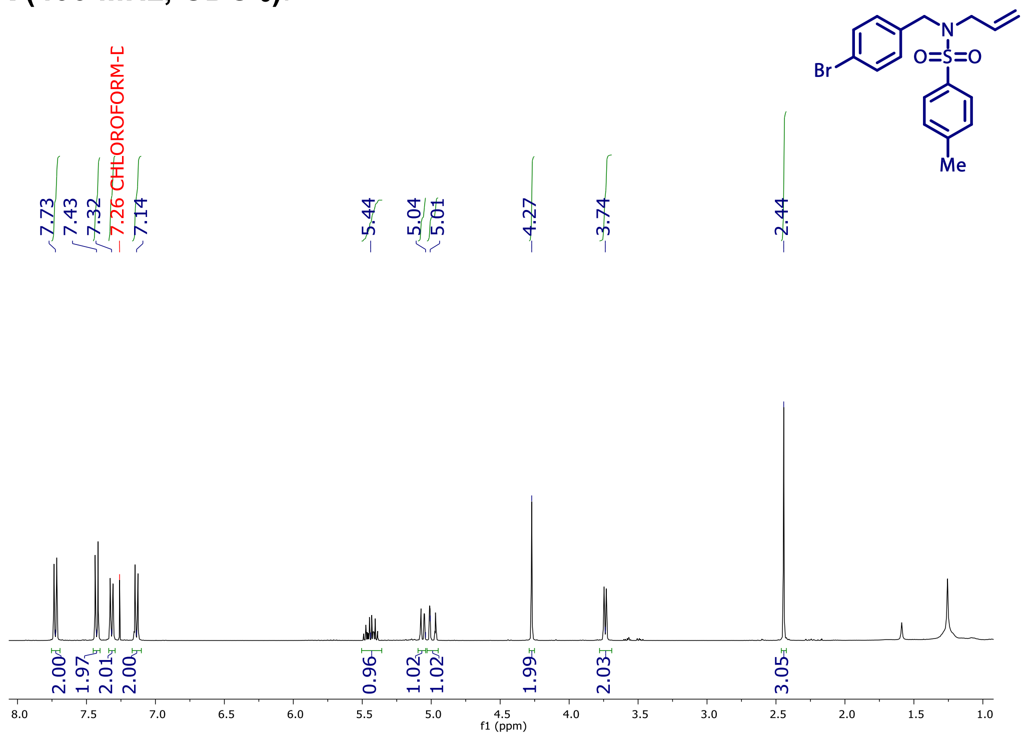

^{13}C NMR (101 MHz, $CDCl_3$):

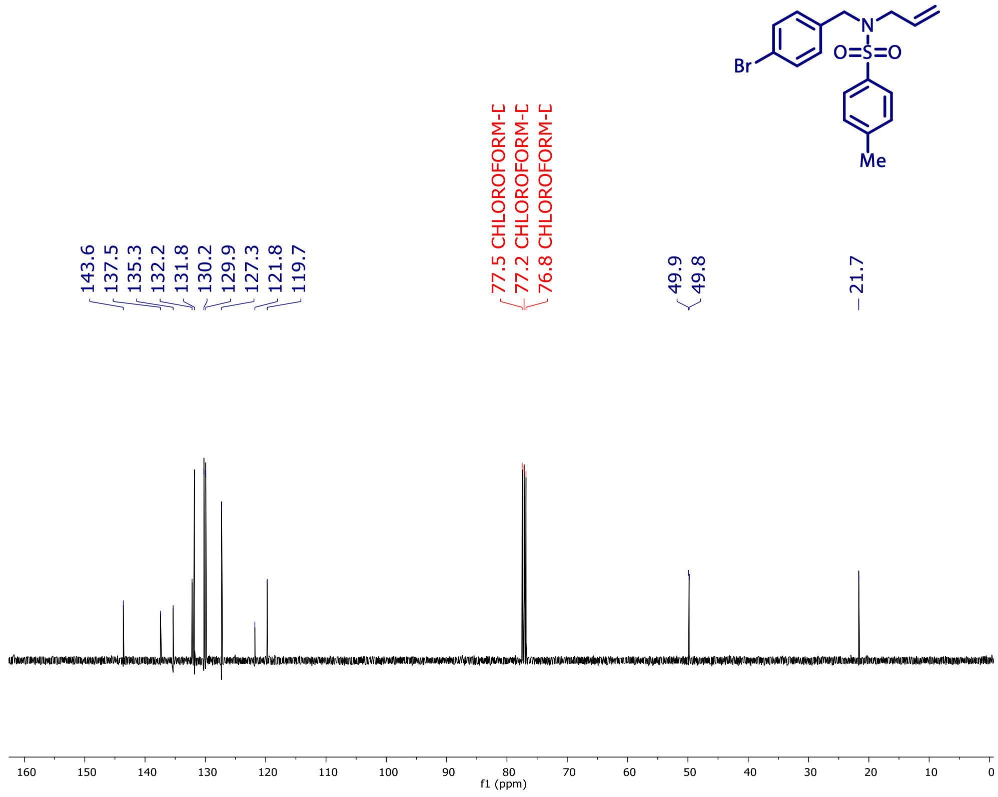

N-allyl-N-cyclopropyl-4-methylbenzenesulfonamide, 2t

^{1}H NMR (400 MHz, $CDCl_3$):

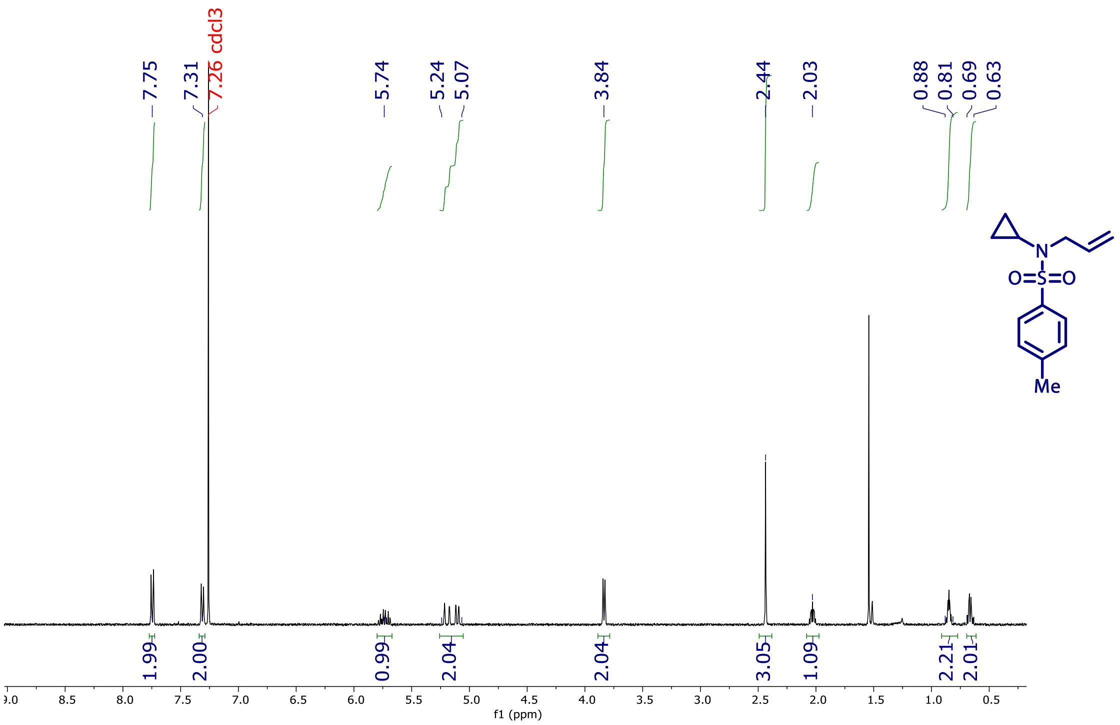

^{13}C NMR (101 MHz, $CDCl_3$):

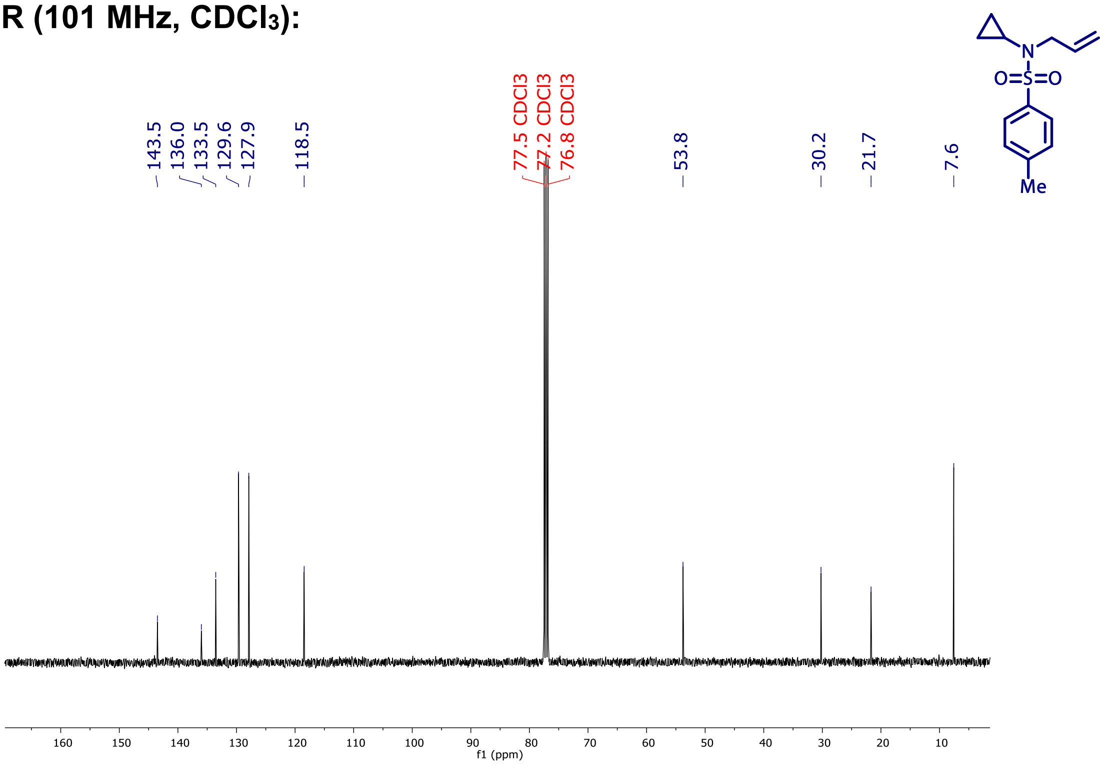

1-(4-Allylphenyl)-N,N-dimethylmethanamine, 2v

^{1}H NMR (400 MHz, $CDCl_3$):

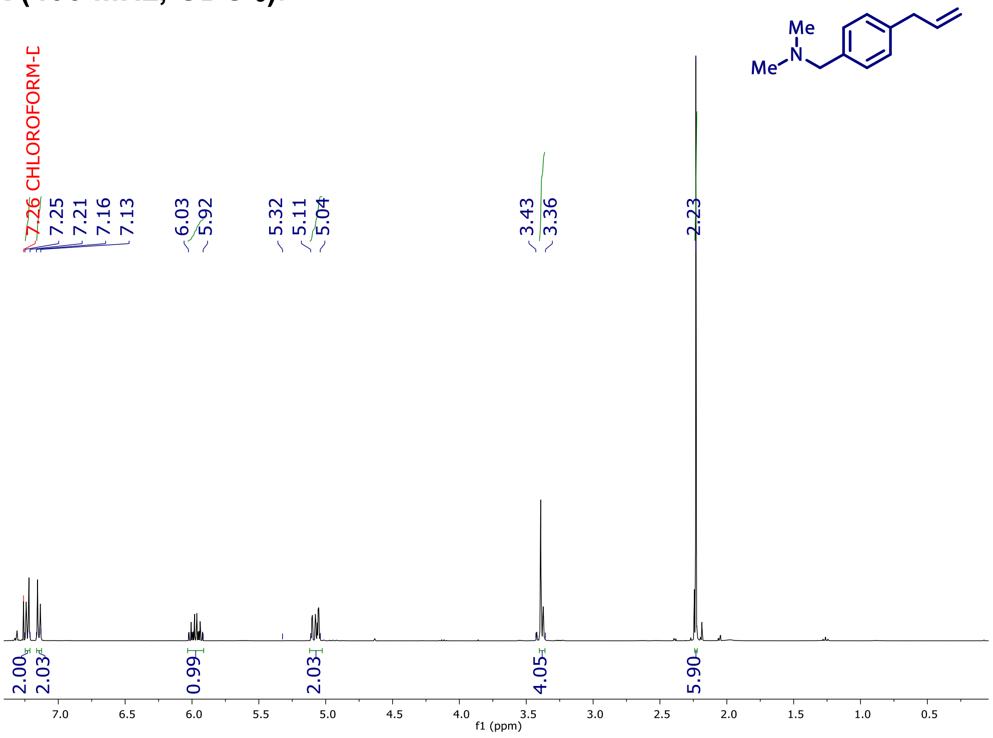

^{13}C NMR (101 MHz, $CDCl_3$):

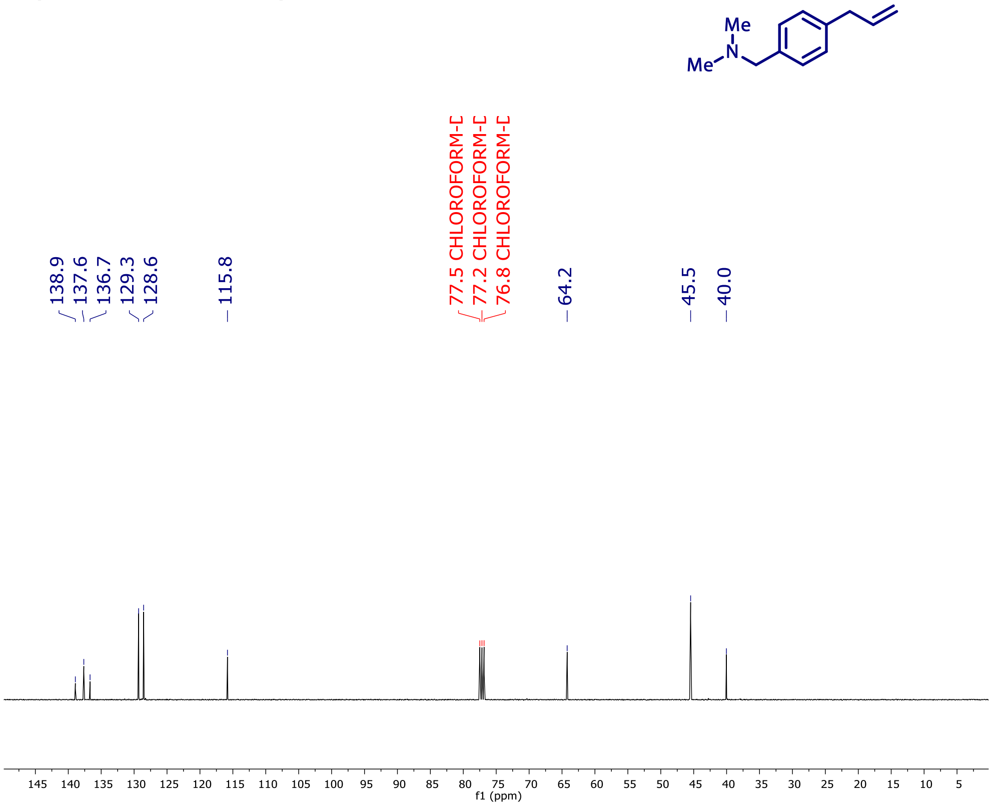

N-allyl-N-(4-fluoro-2-methylphenyl)-4-nitrobenzenesulfonamide, 2ac

^{1}H NMR (500 MHz, $CDCl_3$):

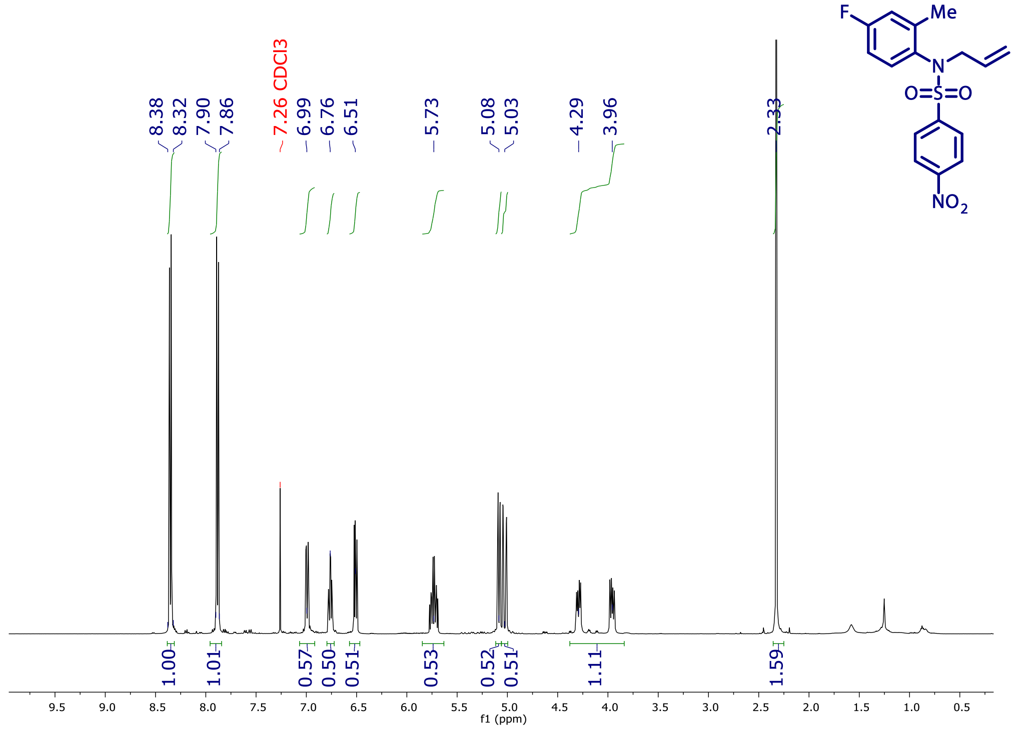

^{13}C NMR (101 MHz, $CDCl_3$):

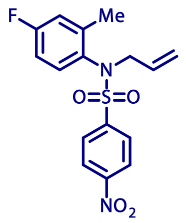

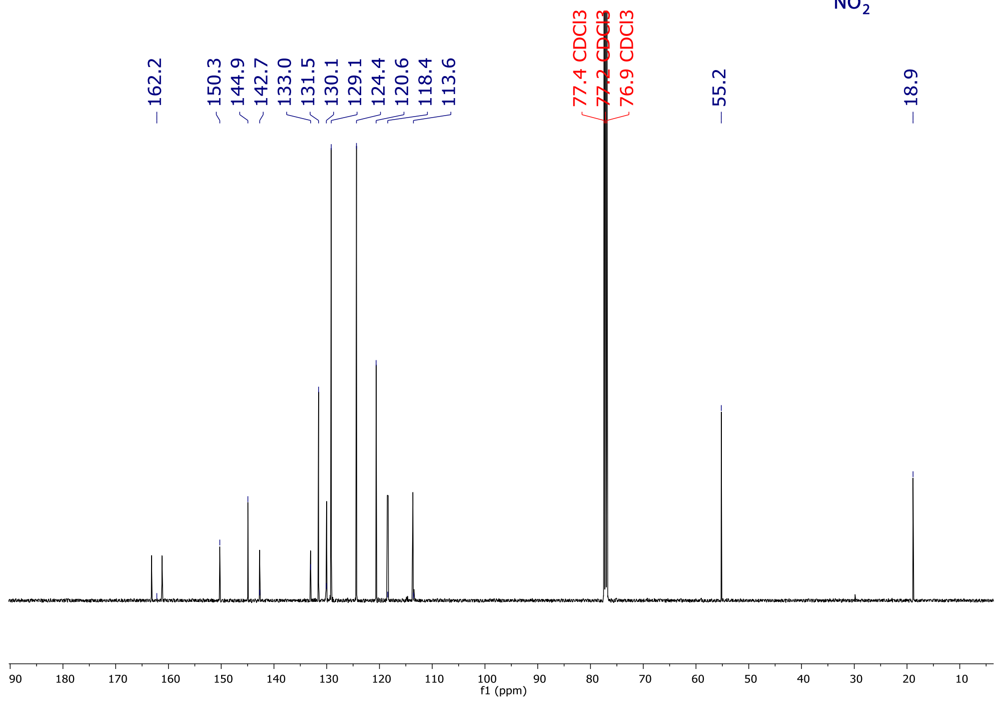

^{19}F NMR (377 MHz, $CDCl_3$):

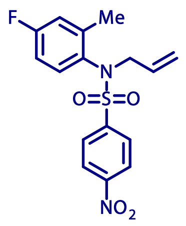

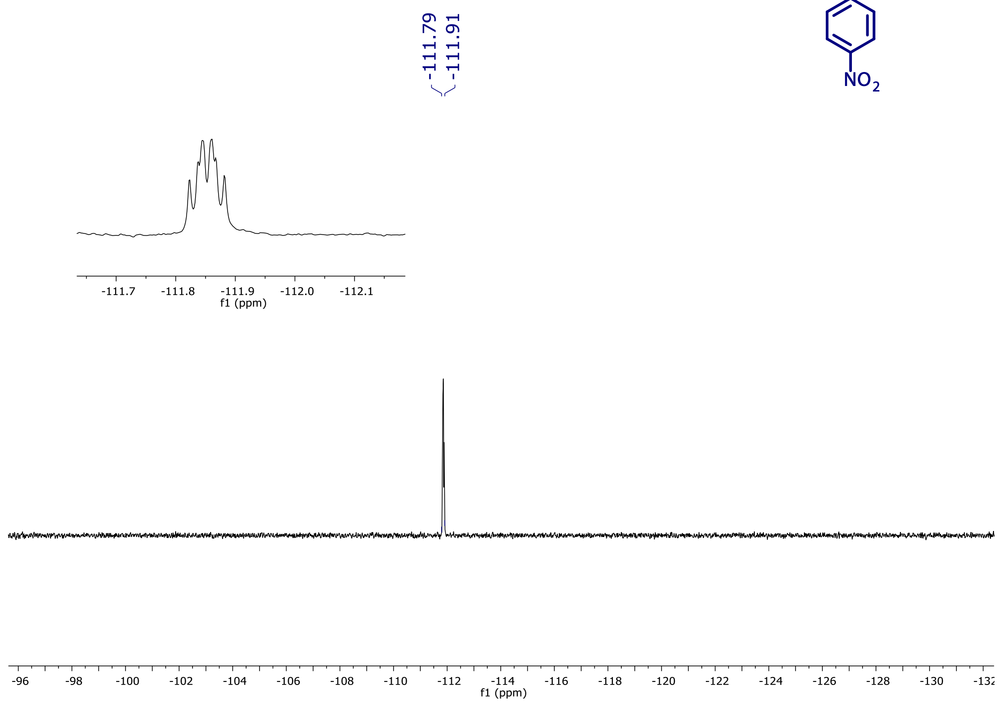

N-allyl-N-(2-bromophenyl)-4-nitrobenzenesulfonamide, 2ae

^{1}H NMR (400 MHz, $CDCl_3$):

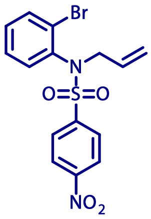

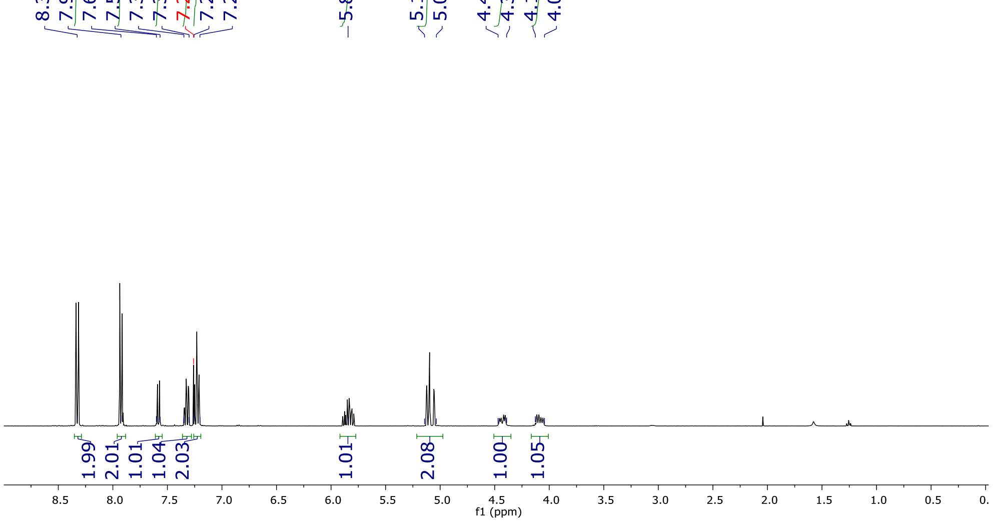

^{13}C NMR (101 MHz, $CDCl_3$):

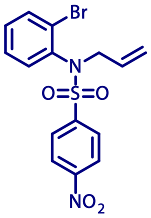

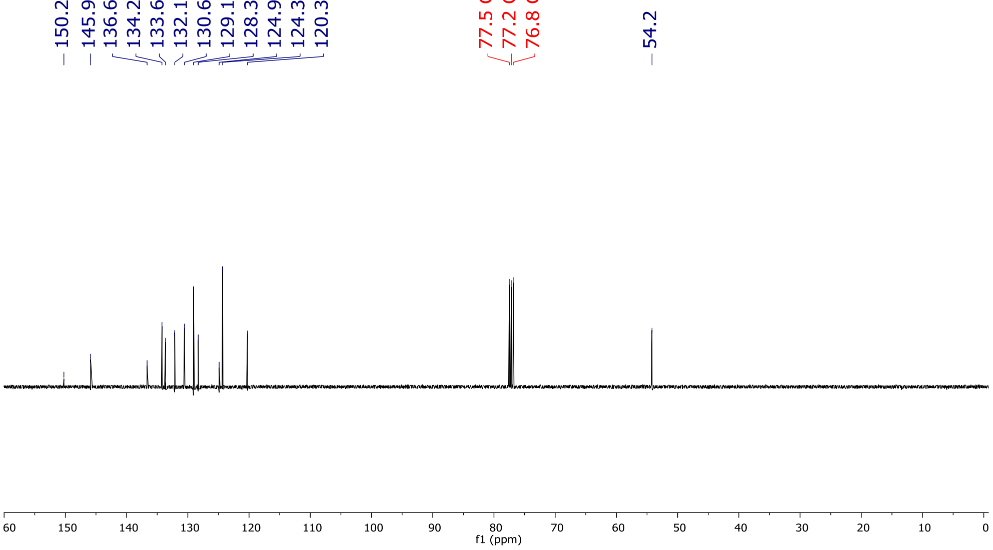

N-(4-fluorophenyl)-N-(2-methylallyl)-4-nitrobenzenesulfonamide, 2ag

^{1}H NMR (400 MHz, $CDCl_3$):

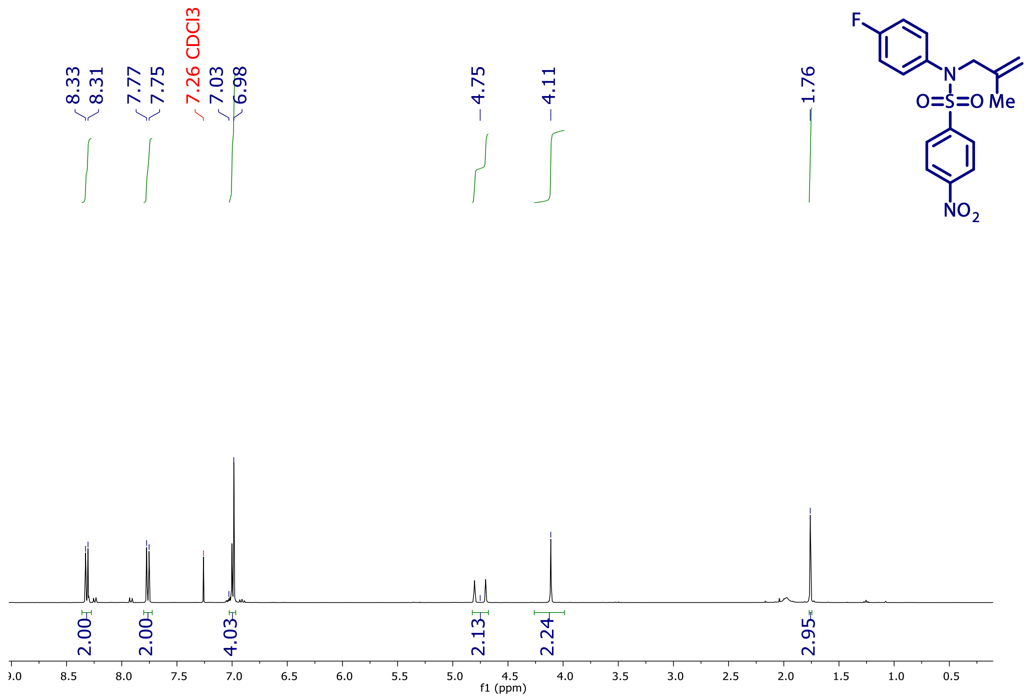

^{13}C NMR (101 MHz, $CDCl_3$):

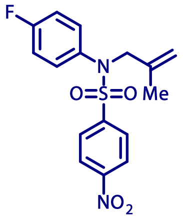

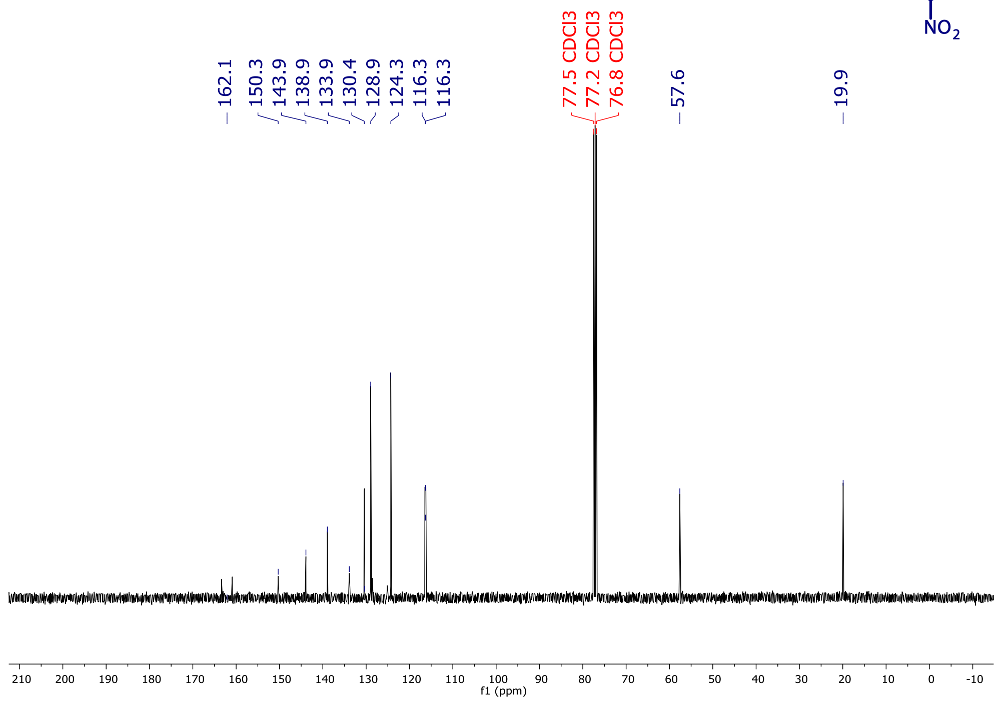

^{19}F NMR (377 MHz, $CDCl_3$):

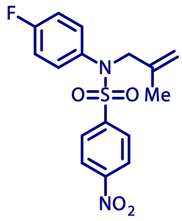

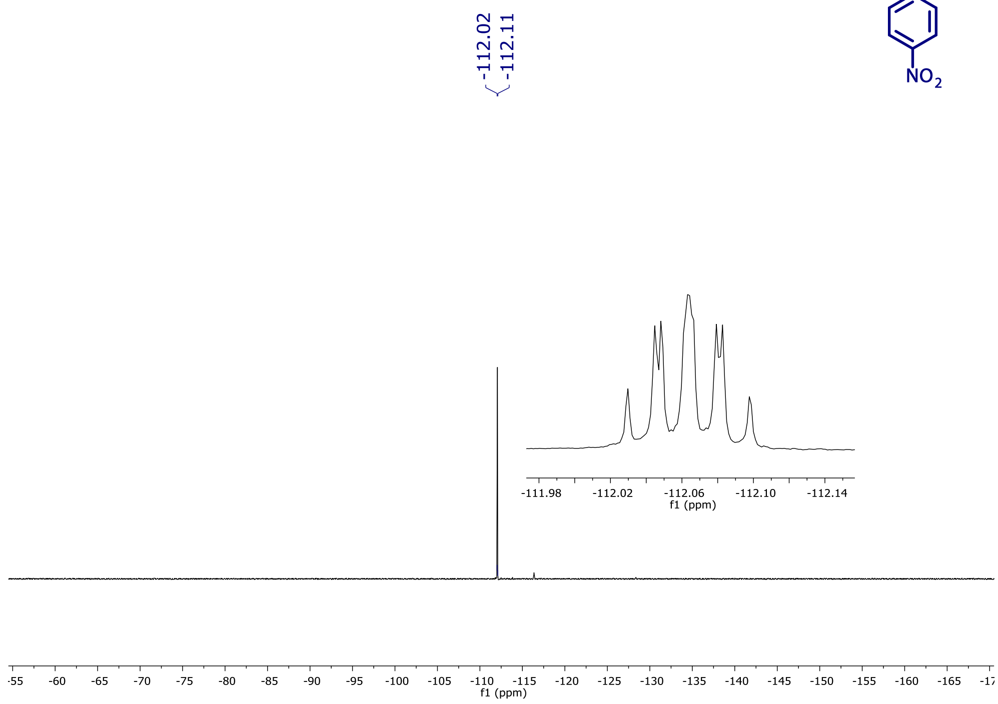

N-allyl-4-methyl-N-(4-methylbenzyl)benzenesulfonamide, 2am

^{1}H NMR (400 MHz, $CDCl_3$):

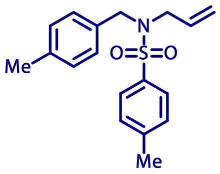

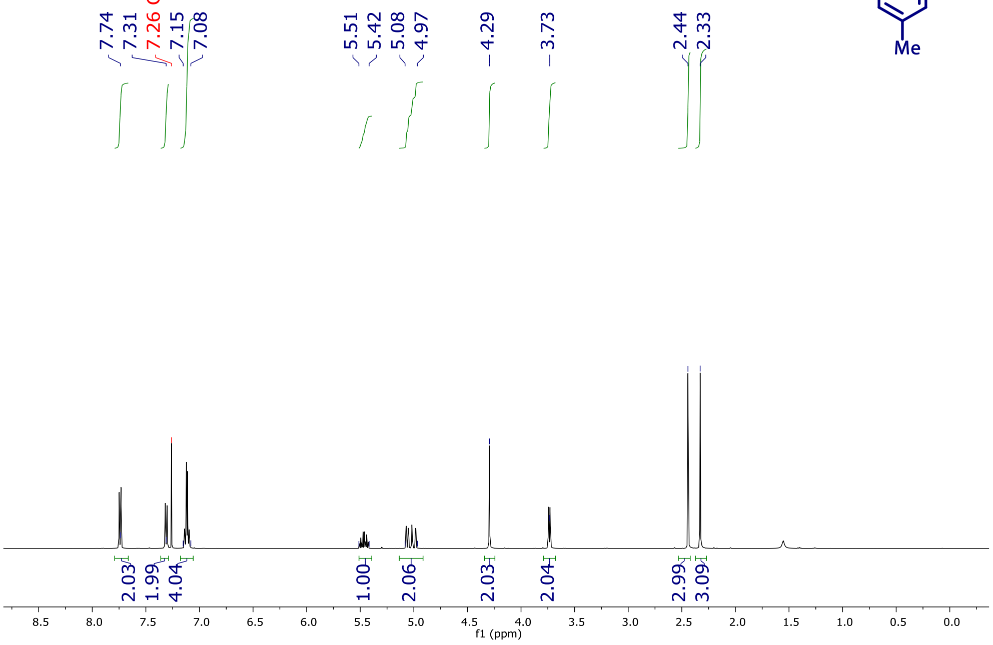

^{13}C NMR (101 MHz, $CDCl_3$):

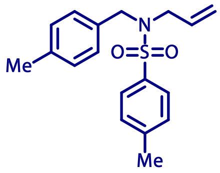

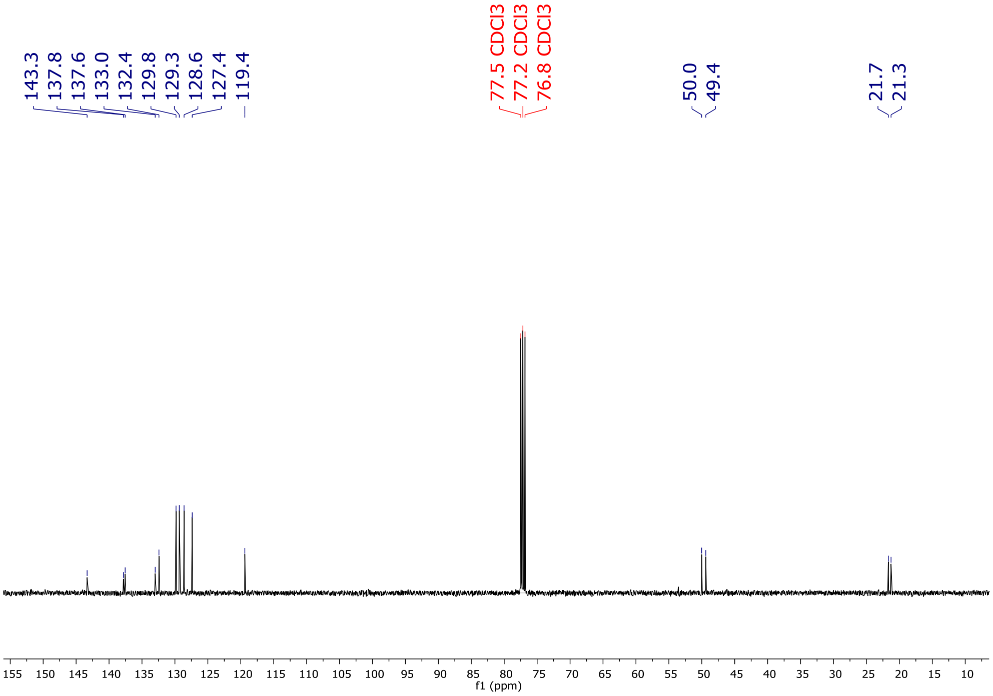

N-(4-chlorobenzyl)-4-methyl-N-(2-methylallyl)benzenesulfonamide, 2an

^{1}H NMR (400 MHz, $CDCl_3$):

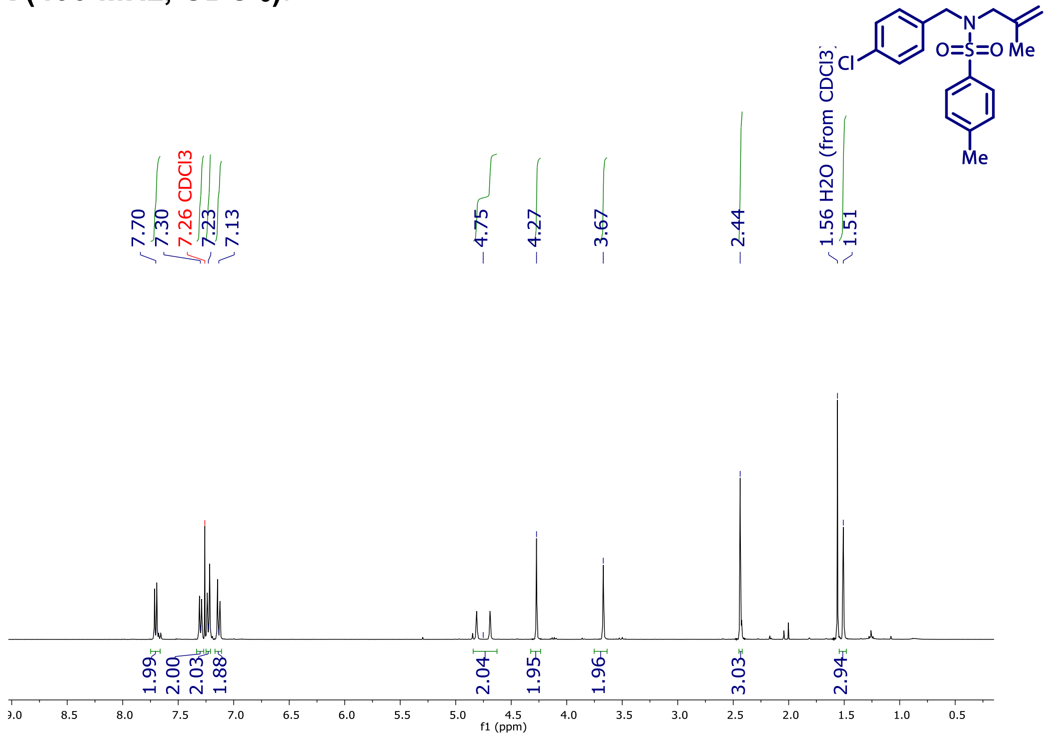

^{13}C NMR (101 MHz, $CDCl_3$):

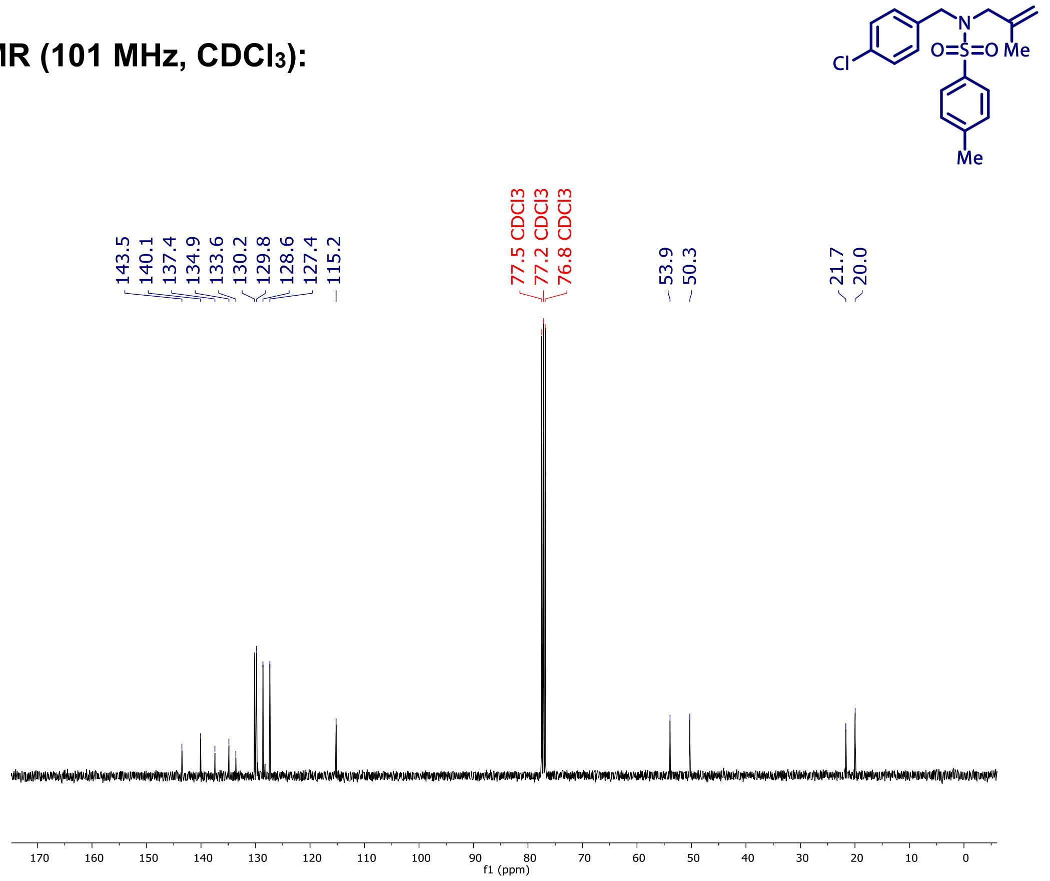

N-(but-2-en-1-yl)-N-(4-chlorobenzyl)-4-methylbenzenesulfonamide, 2ao

^{1}H NMR (500 MHz, CDCl$_3$):

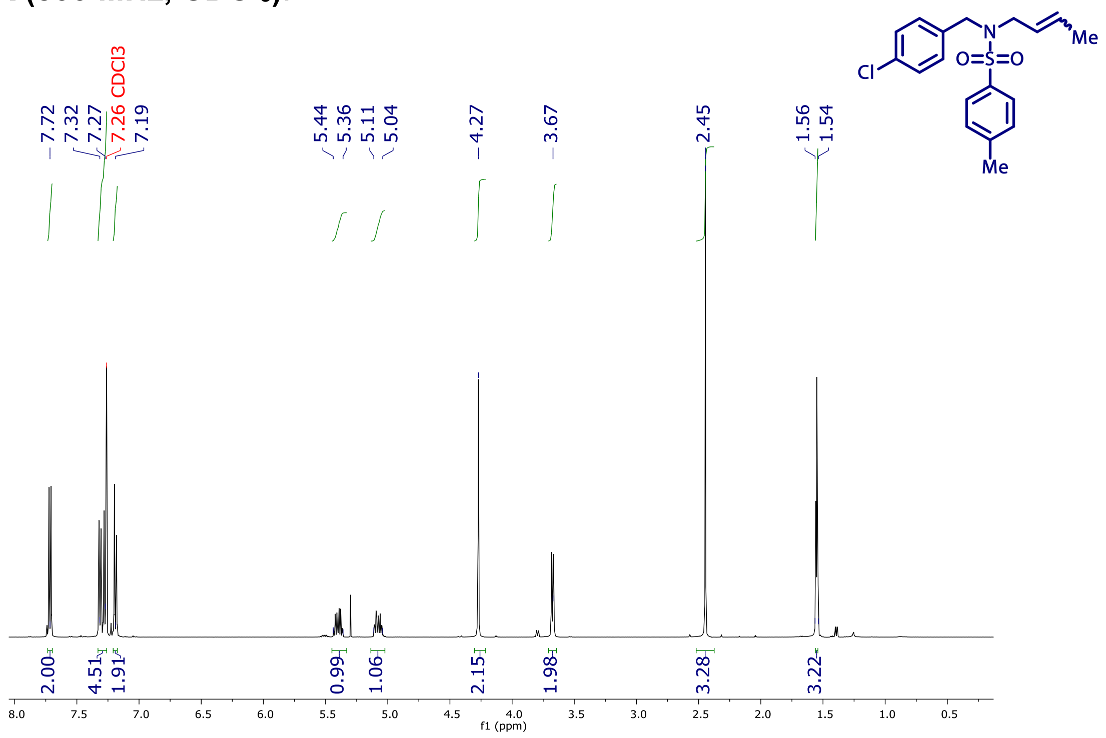

^{13}C NMR (101 MHz, CDCl$_3$):

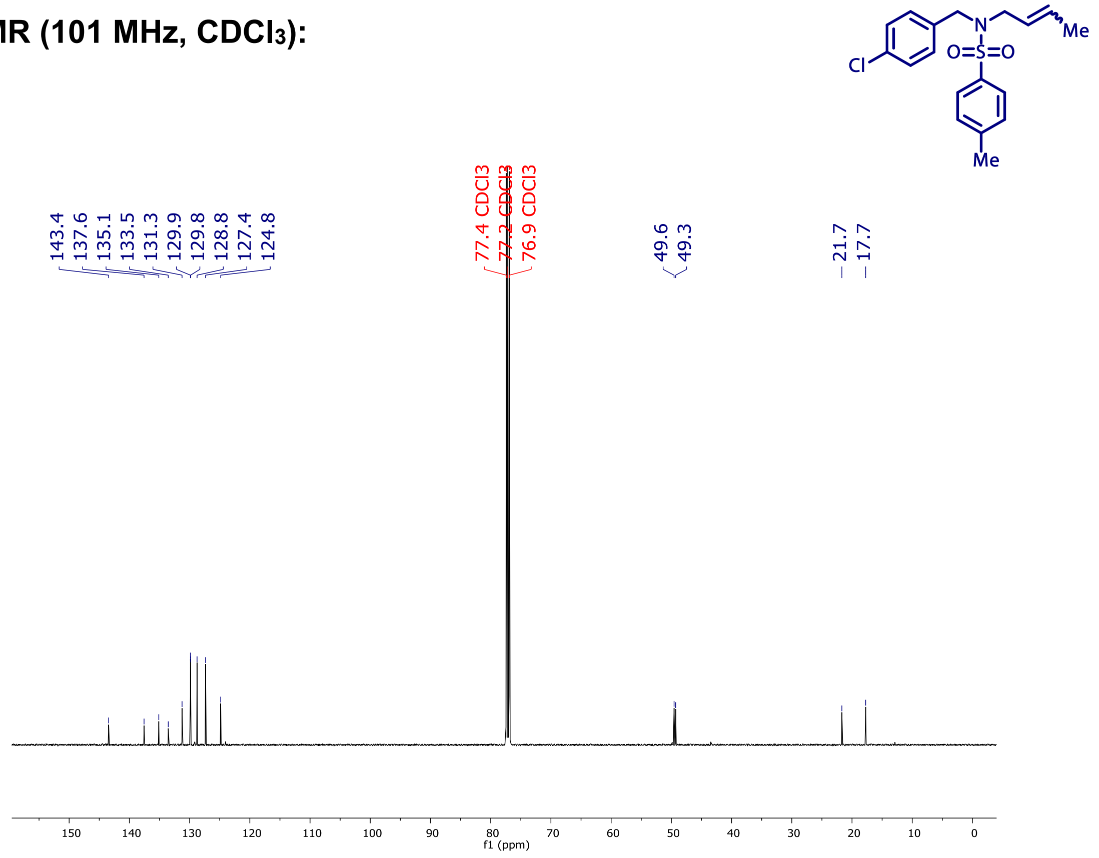

N-(4-chlorobenzyl)-4-methyl-N-(3-methylbut-2-en-1-yl)benzenesulfonamide, 2ap

^{1}H NMR (500 MHz, $CDCl_3$):

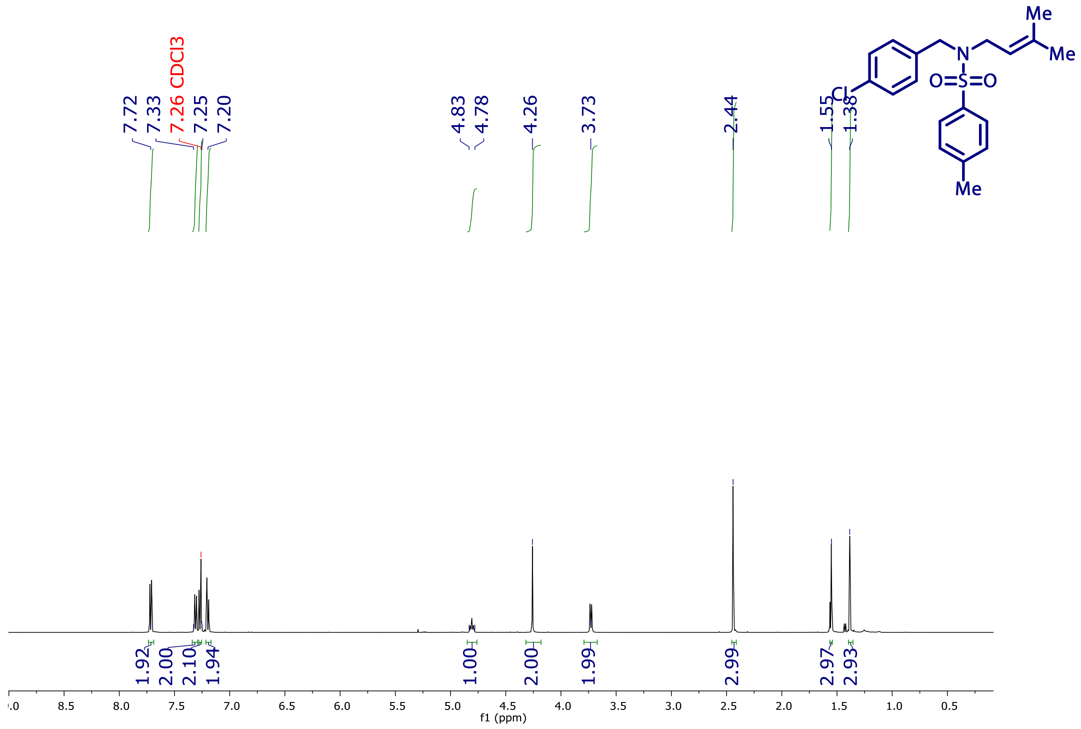

^{13}C NMR (126 MHz, $CDCl_3$):

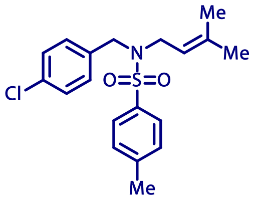

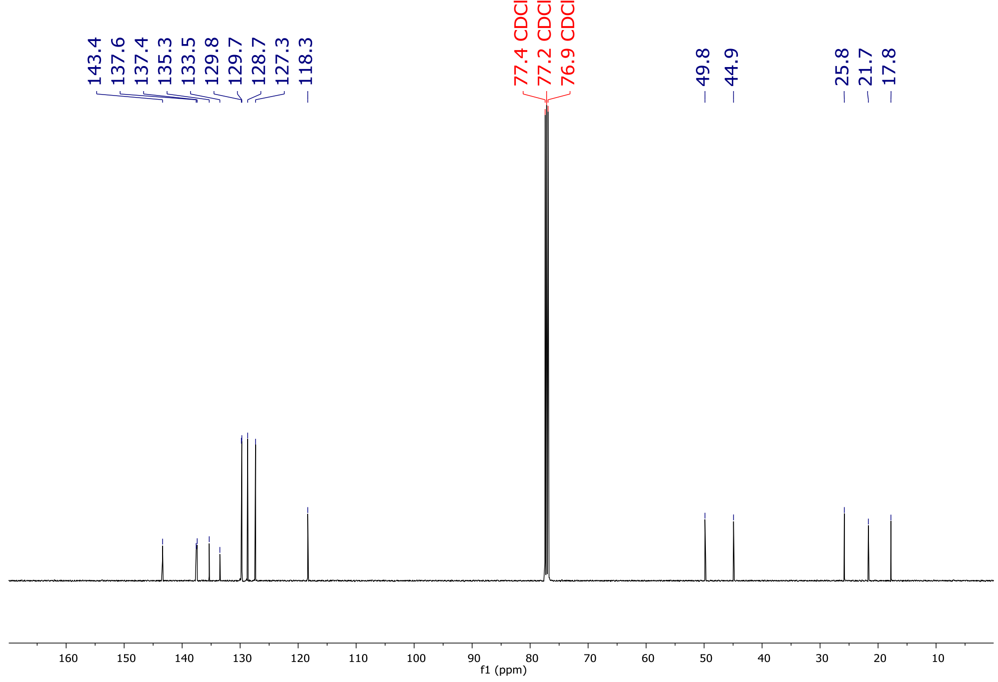

1-(((2-Methylallyl)oxy)methyl)-4-(trifluoromethyl)benzene, 2au

^{1}H NMR (500 MHz, $CDCl_3$):

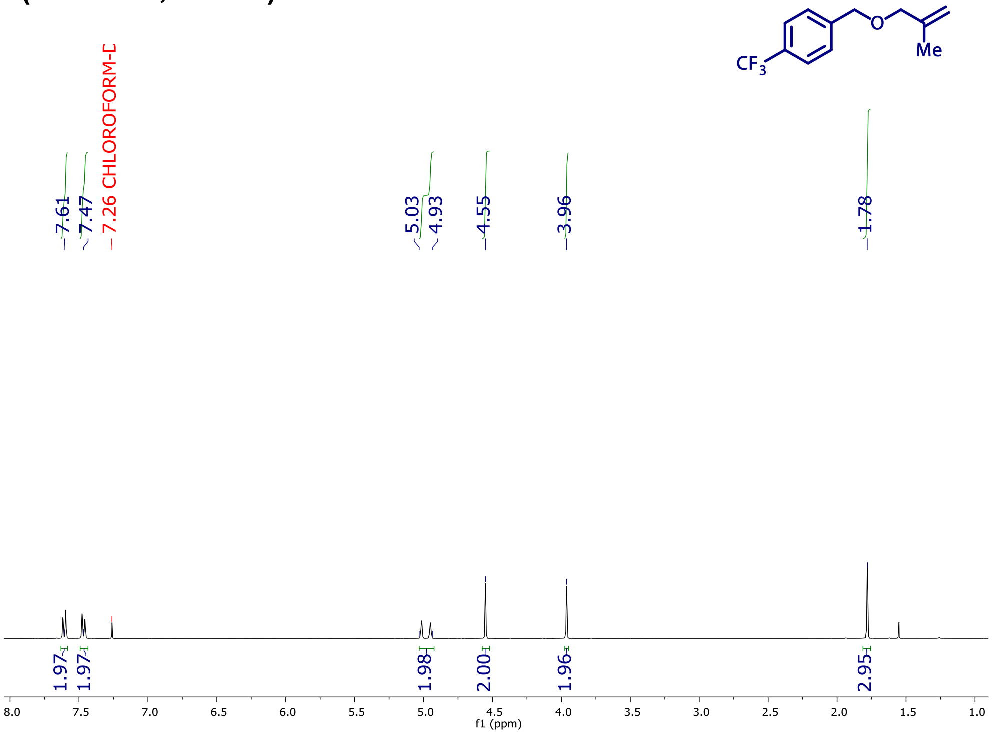

^{13}C NMR (126 MHz, $CDCl_3$):

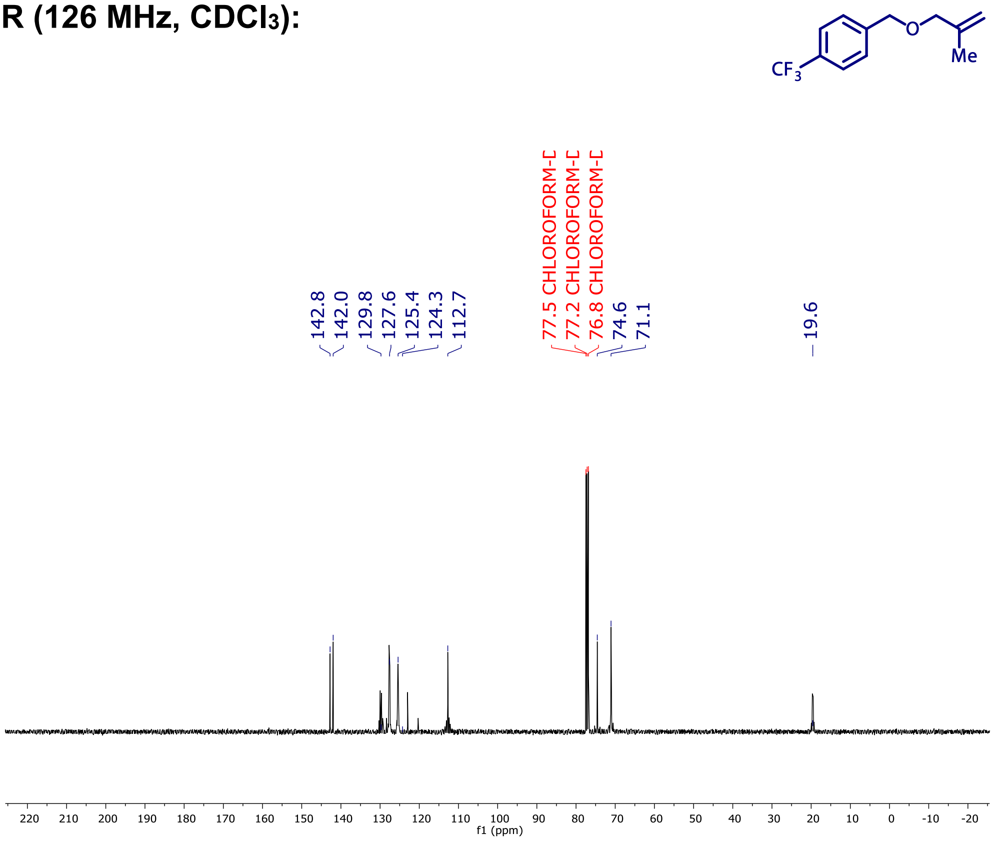

NMR Spectra of products

1-(2,3-difluoropropyl)-4-fluorobenzene, 3d

^{1}H NMR (500 MHz, CDCl$_3$):

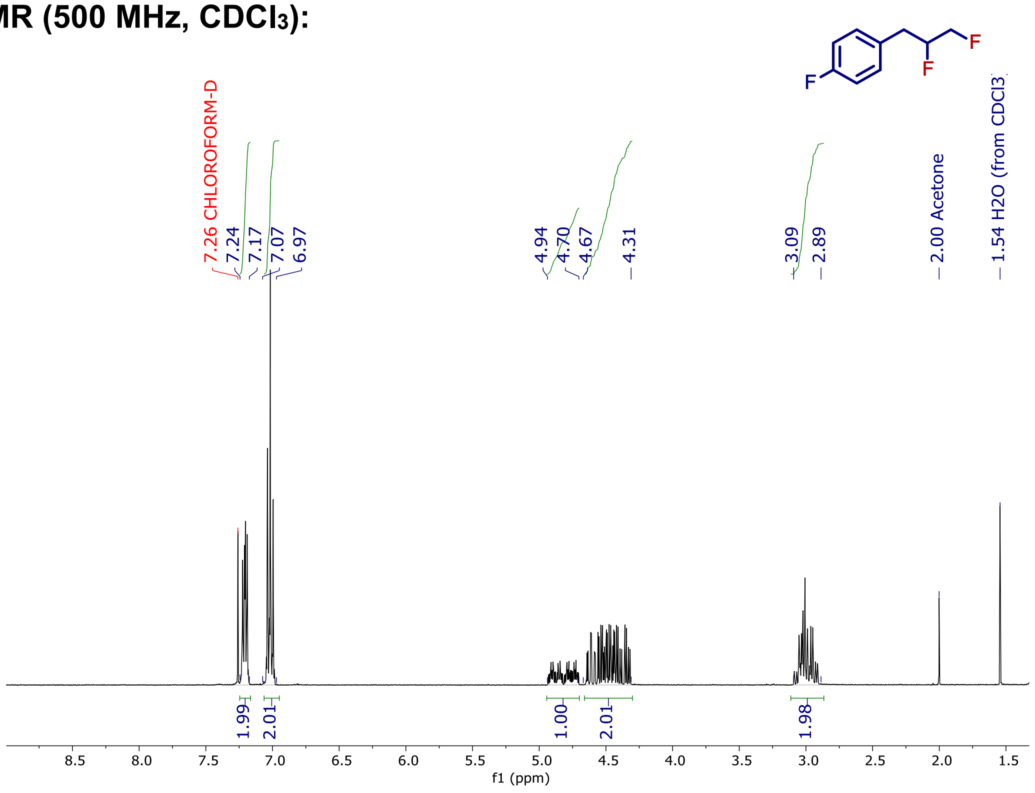

^{13}C NMR (126 MHz, CDCl$_3$):

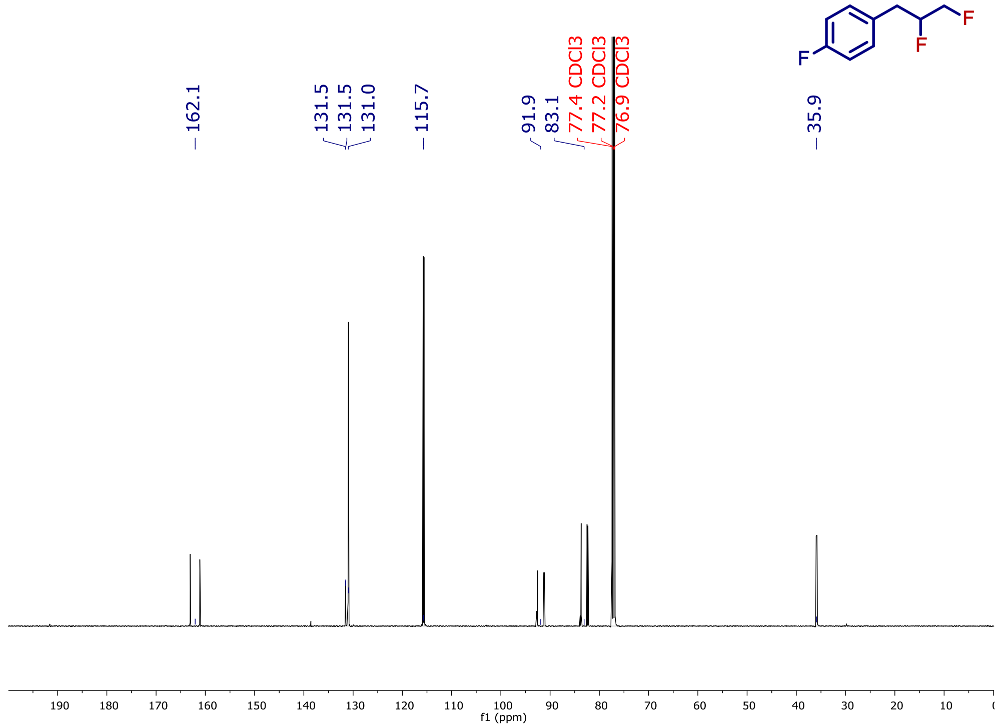

^{19}F NMR (376 MHz, CDCl$_3$):

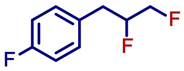

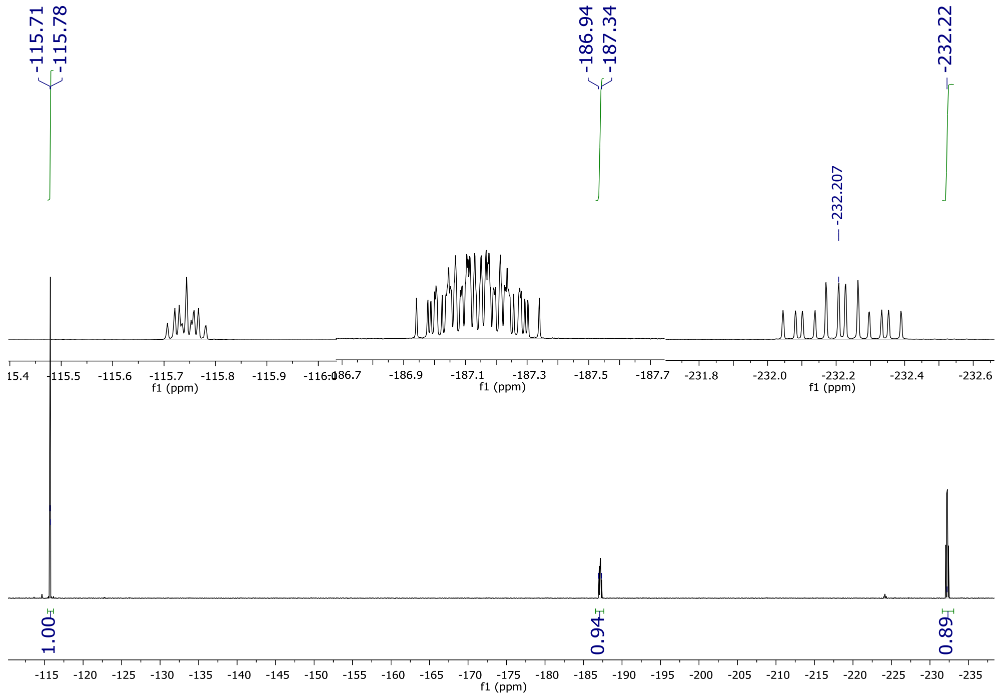

1-(2,3-difluoropropyl)-2,3,4,5,6-pentafluorobenzene, 2e

^{1}H NMR (500 MHz, $CDCl_3$):

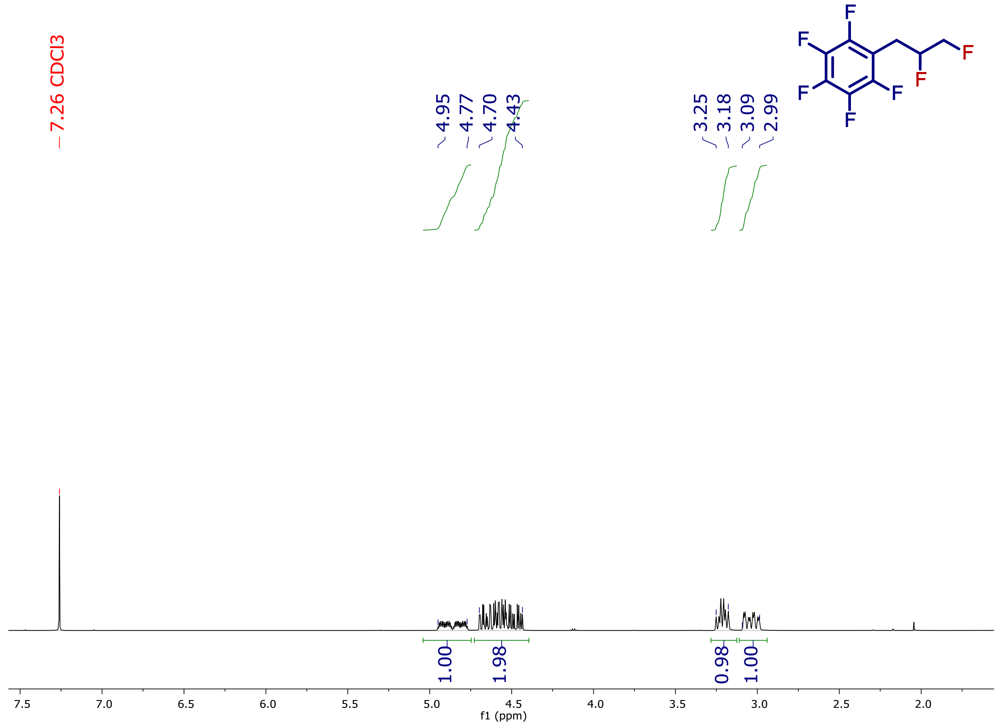

^{13}C NMR (126 MHz, $CDCl_3$):

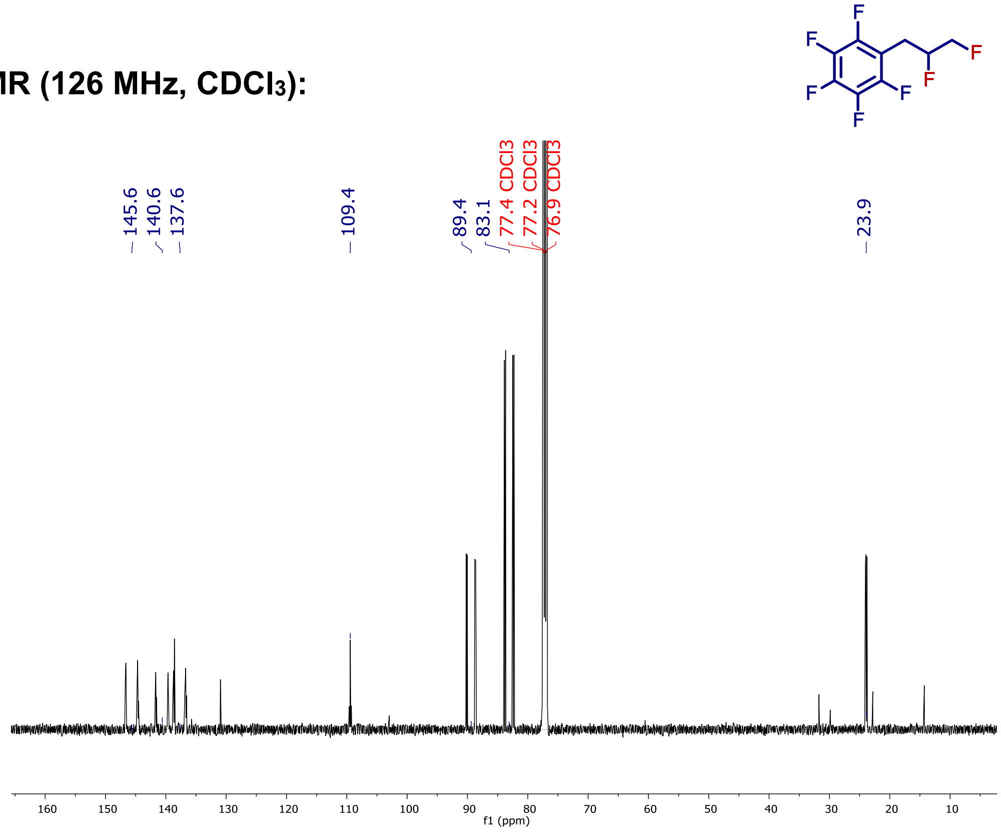

^{19}F NMR (376 MHz, CDCl$_3$):

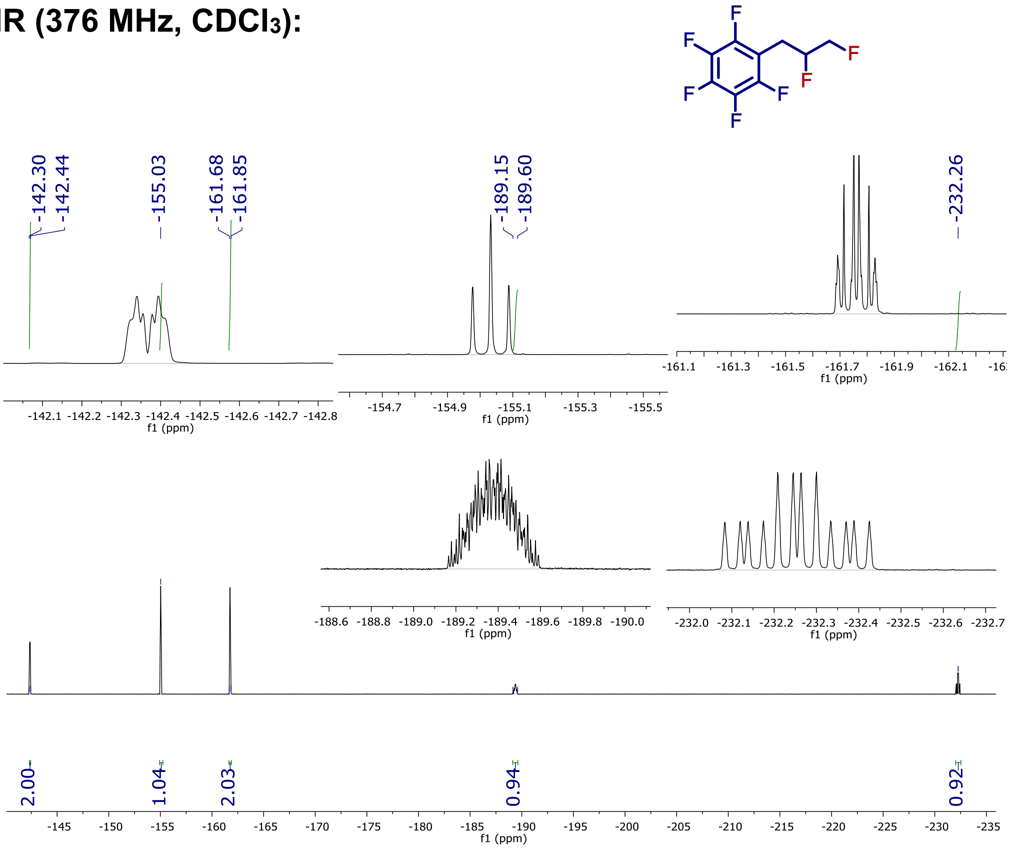

1-((2,3-difluoropropoxy)methyl)-4-fluorobenzene, 3j

^{1}H NMR (500 MHz, $CDCl_3$):

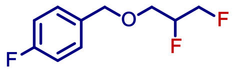

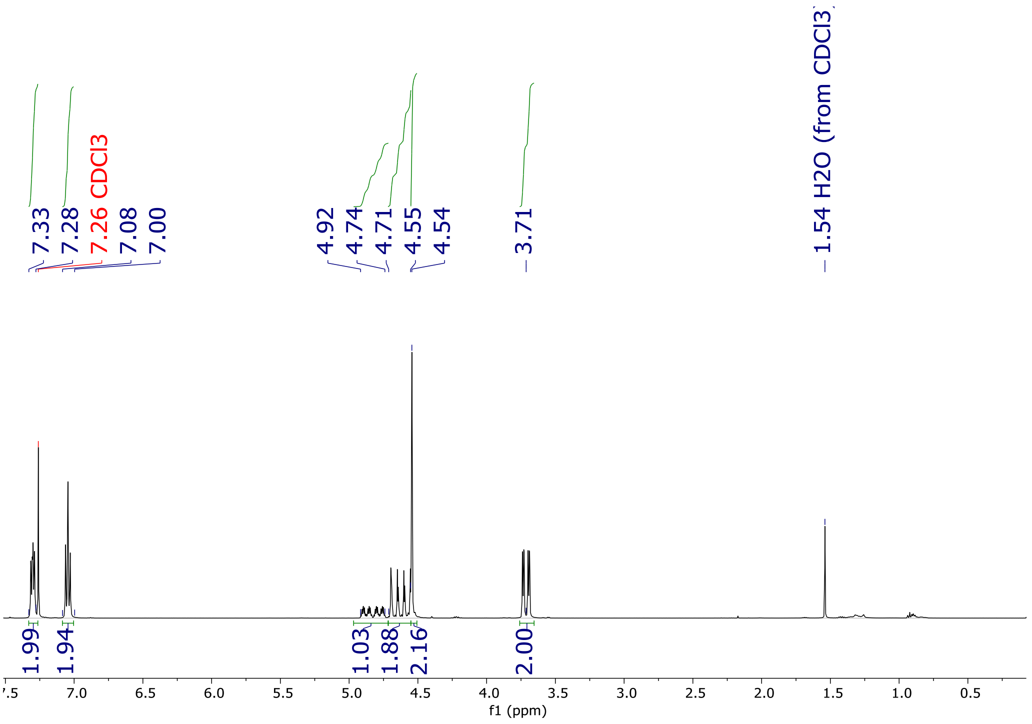

^{13}C NMR (126 MHz, $CDCl_3$):

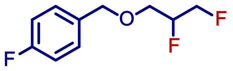

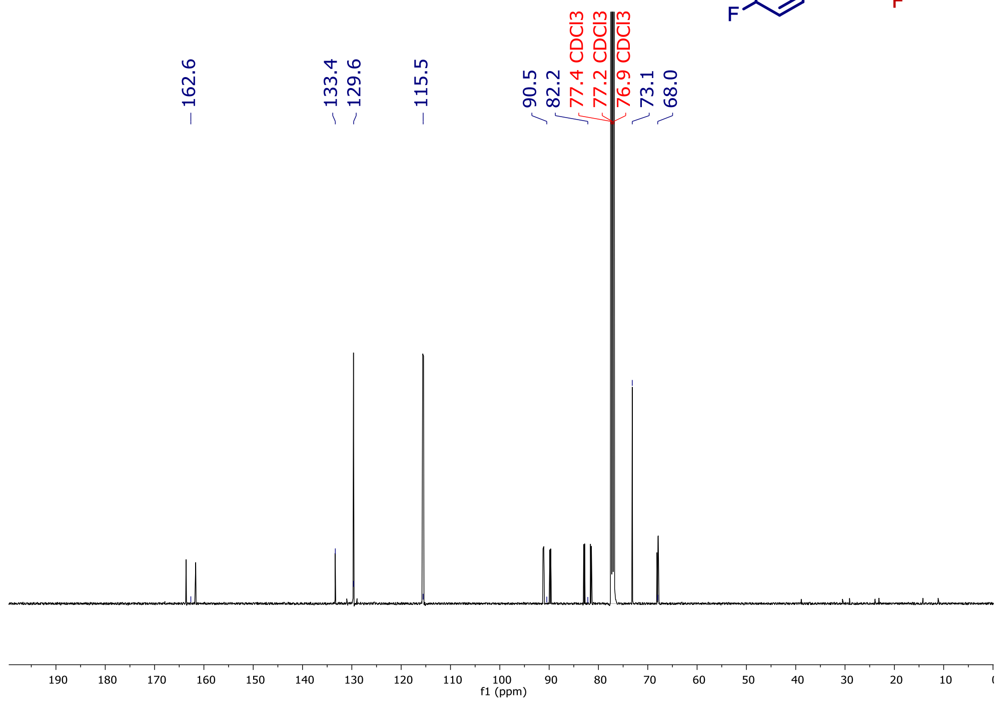

^{19}F NMR (376 MHz, CDCl$_3$):

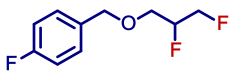

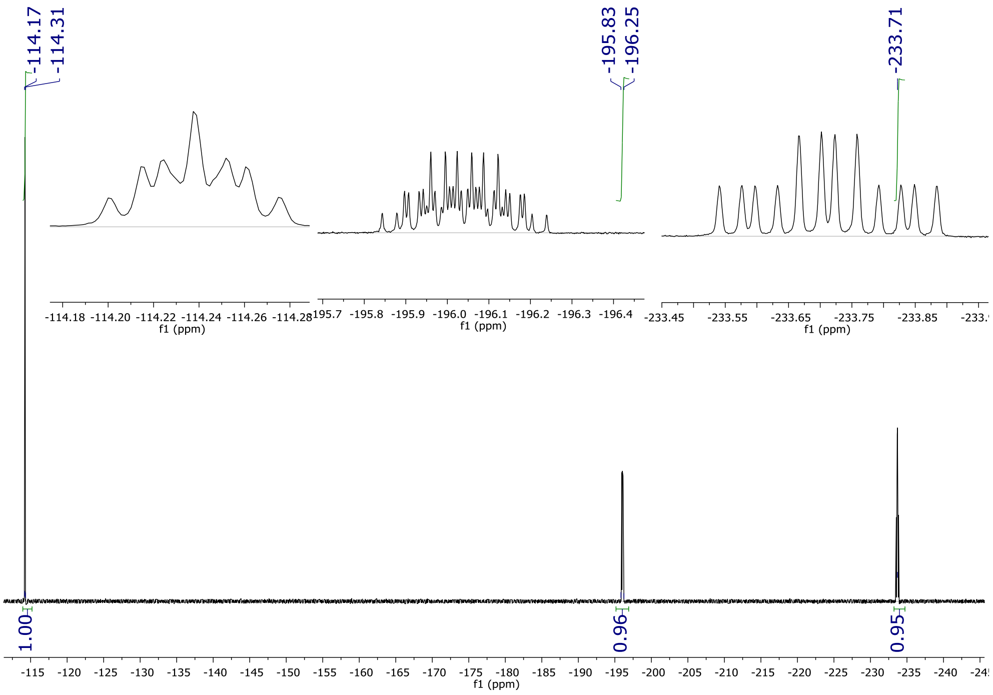

1-((2,3-difluoropropoxy)methyl)-4-nitrobenzene, 3k

^{1}H NMR (500 MHz, $CDCl_3$):

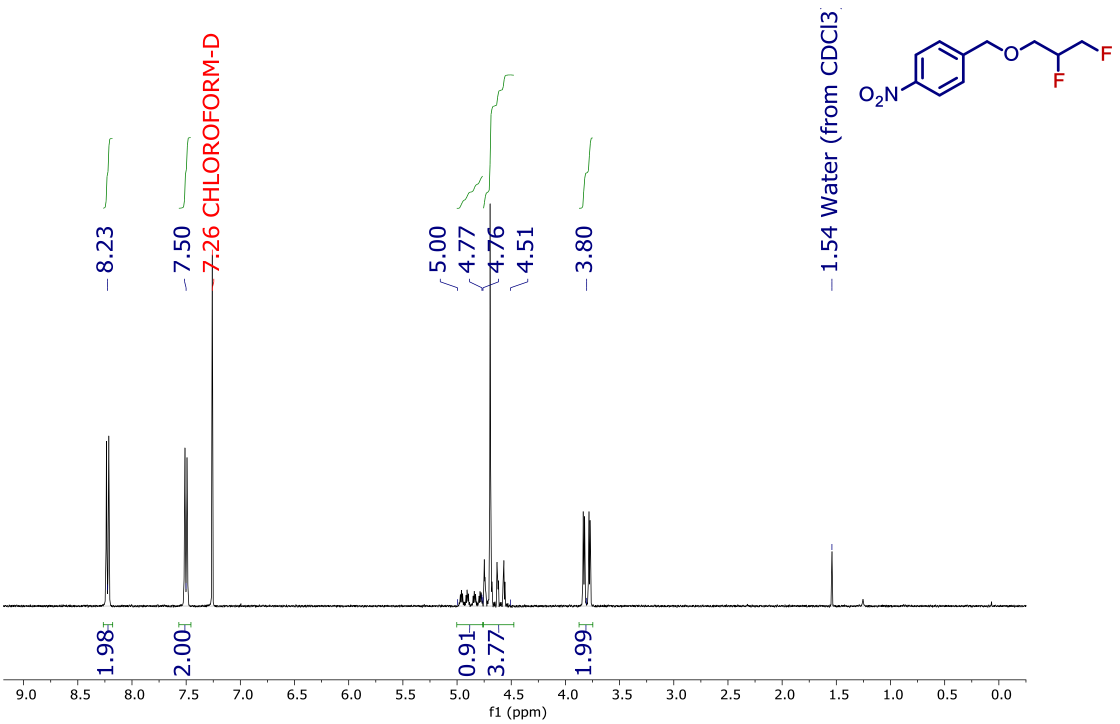

^{13}C NMR (126 MHz, $CDCl_3$):

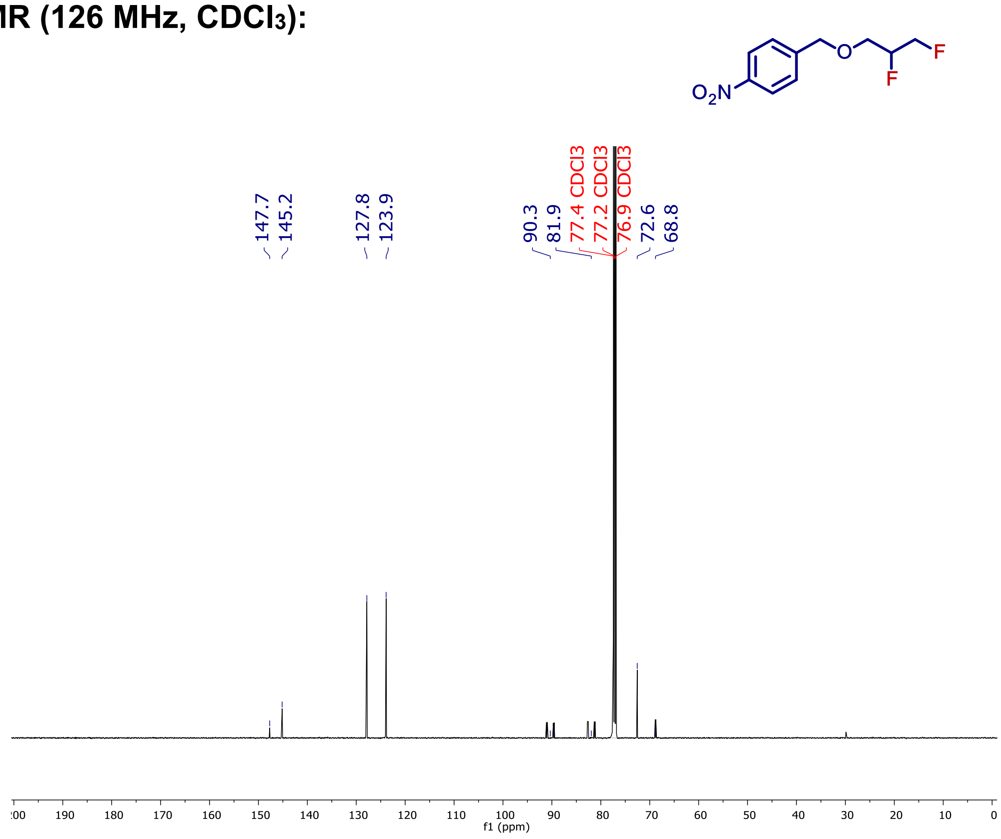

^{19}F NMR (376 MHz, $CDCl_3$):

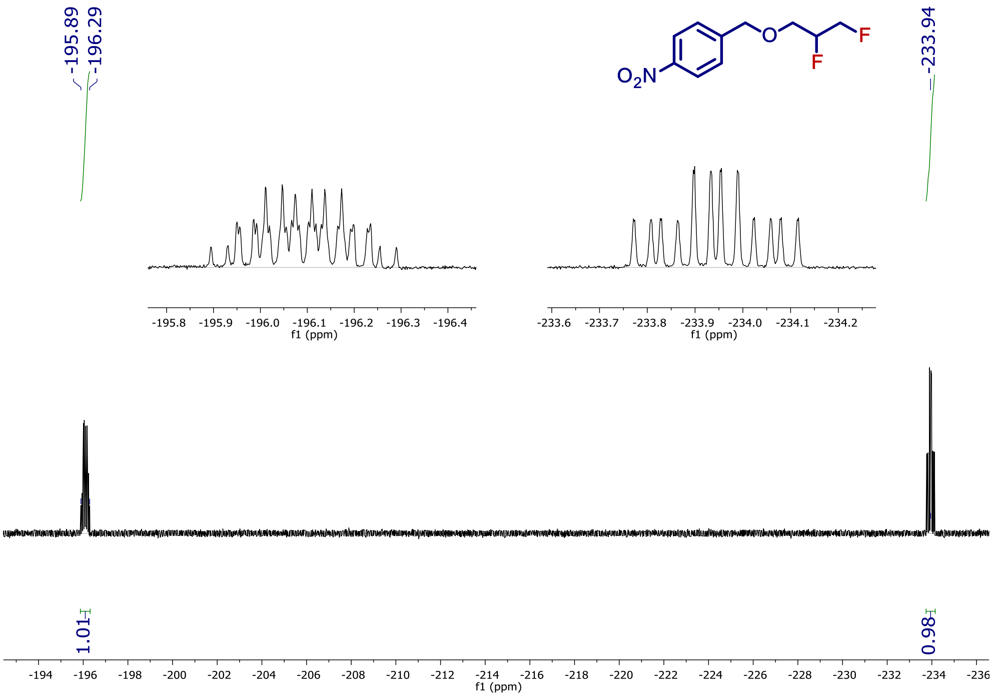

1-bromo-2-((2,3-difluoropropoxy)methyl)benzene, 3l

^{1}H NMR (500 MHz, $CDCl_3$):

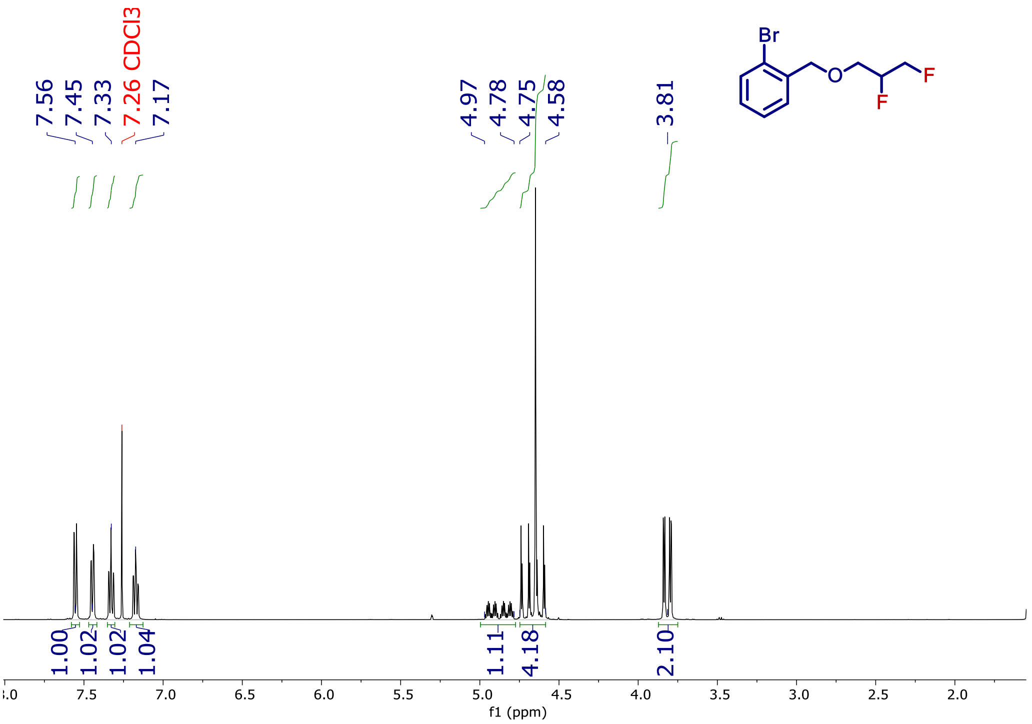

^{13}C NMR (126 MHz, $CDCl_3$):

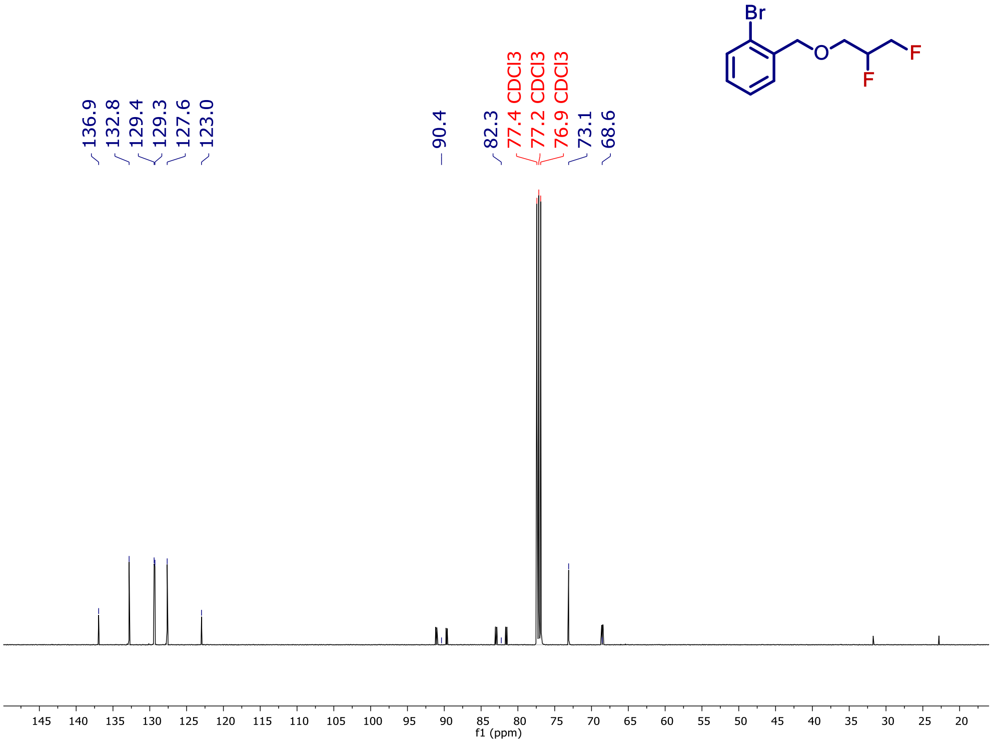

^{19}F NMR (376 MHz, CDCl$_3$):

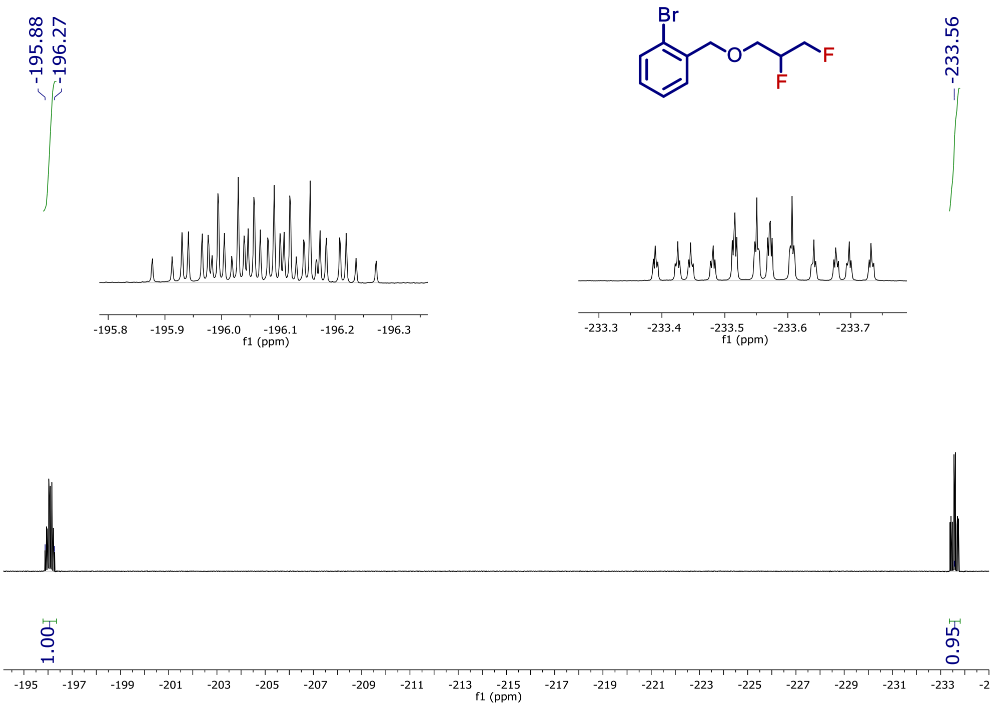

2-((2,3-difluoropropoxy)methyl)pyridine, 3m

^{1}H NMR (500 MHz, $CDCl_3$):

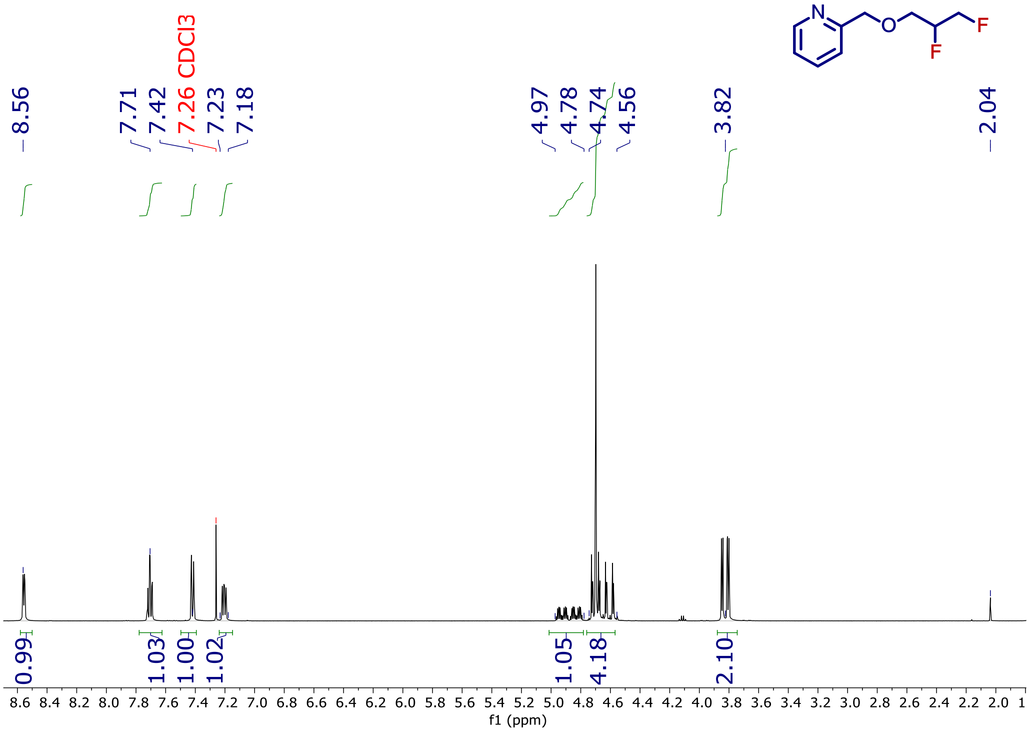

^{13}C NMR (126 MHz, $CDCl_3$):

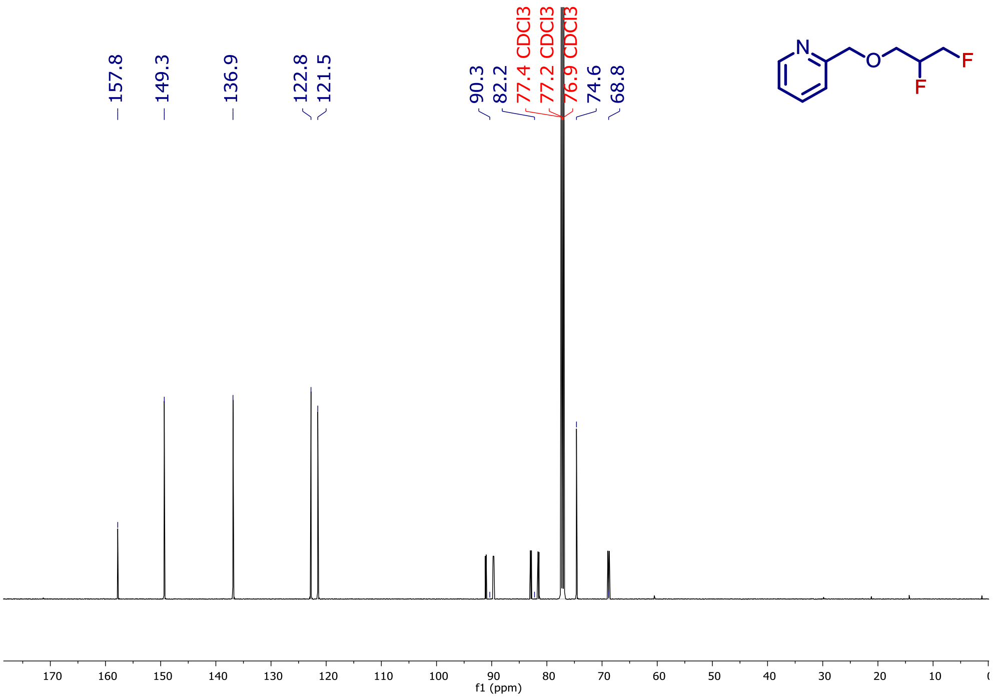

¹⁹F NMR (376 MHz, CDCl₃):

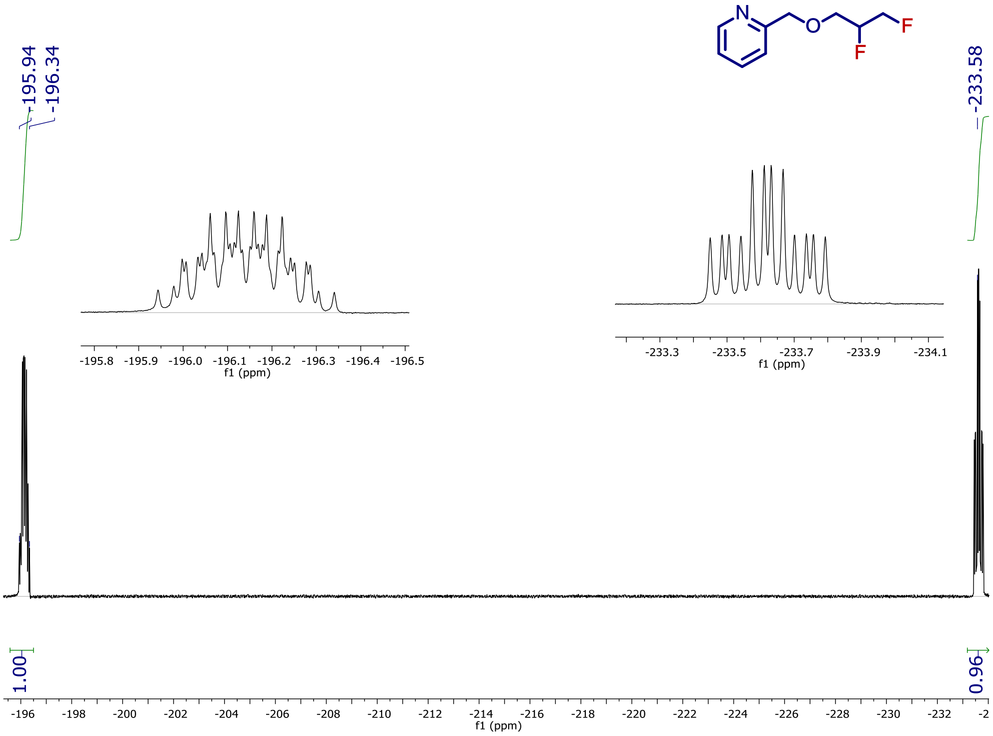

4-((2,3-difluoropropoxy)methyl)pyridine, 3n

^{1}H NMR (500 MHz, $CDCl_3$):

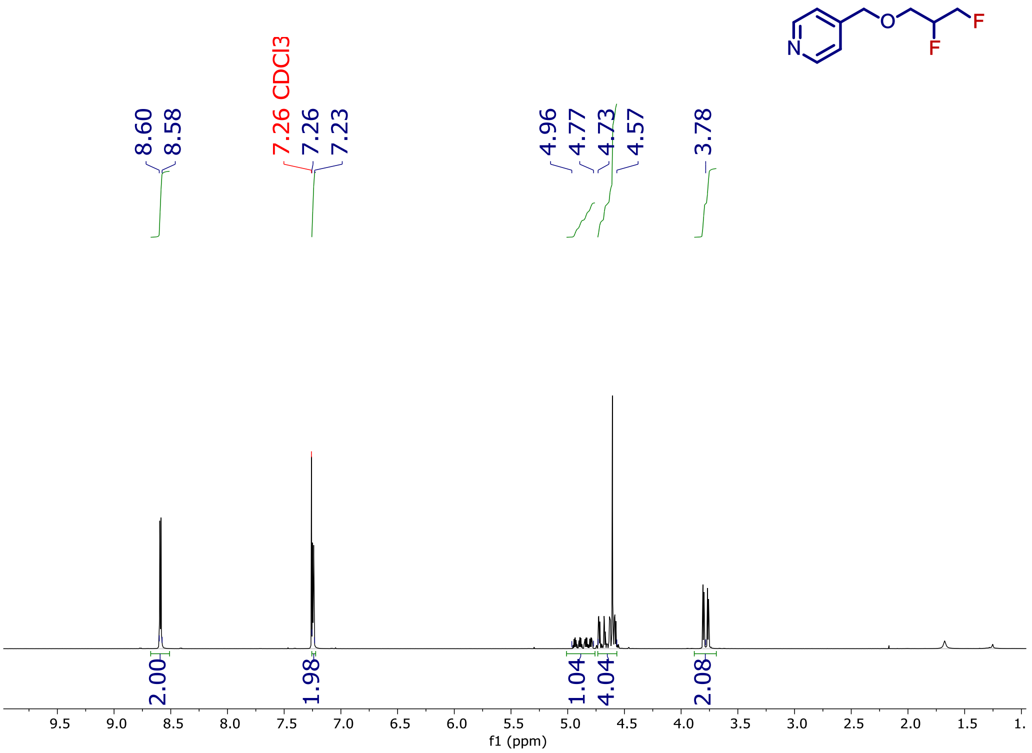

^{13}C NMR (126 MHz, $CDCl_3$):

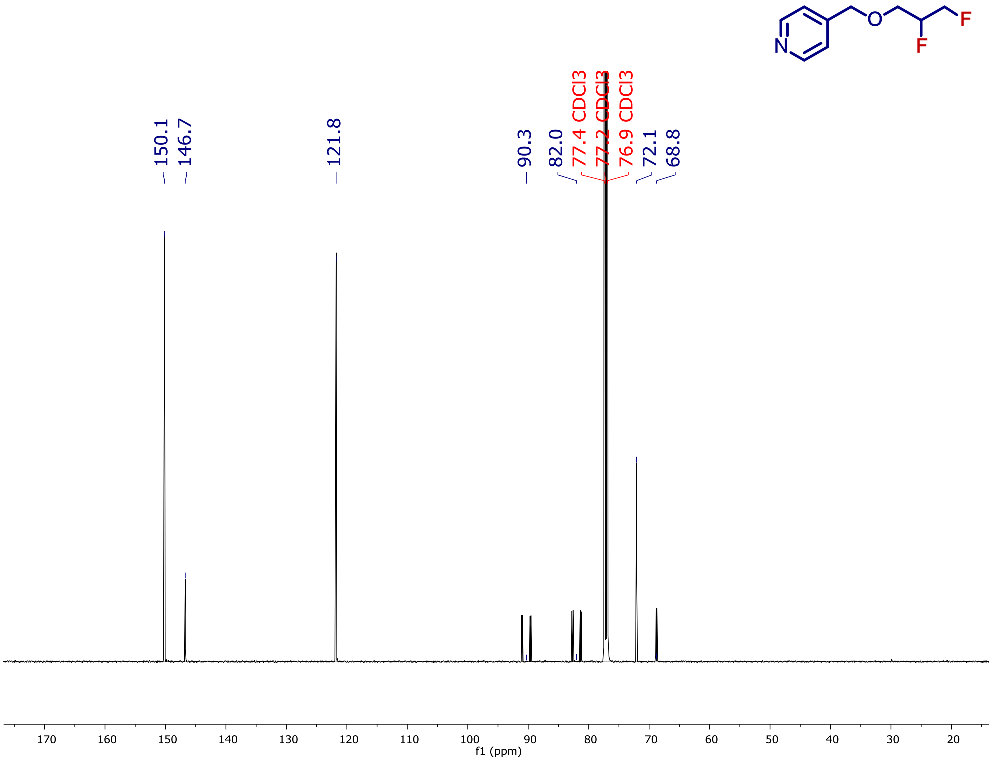

^{19}F NMR (376 MHz, CDCl$_3$):

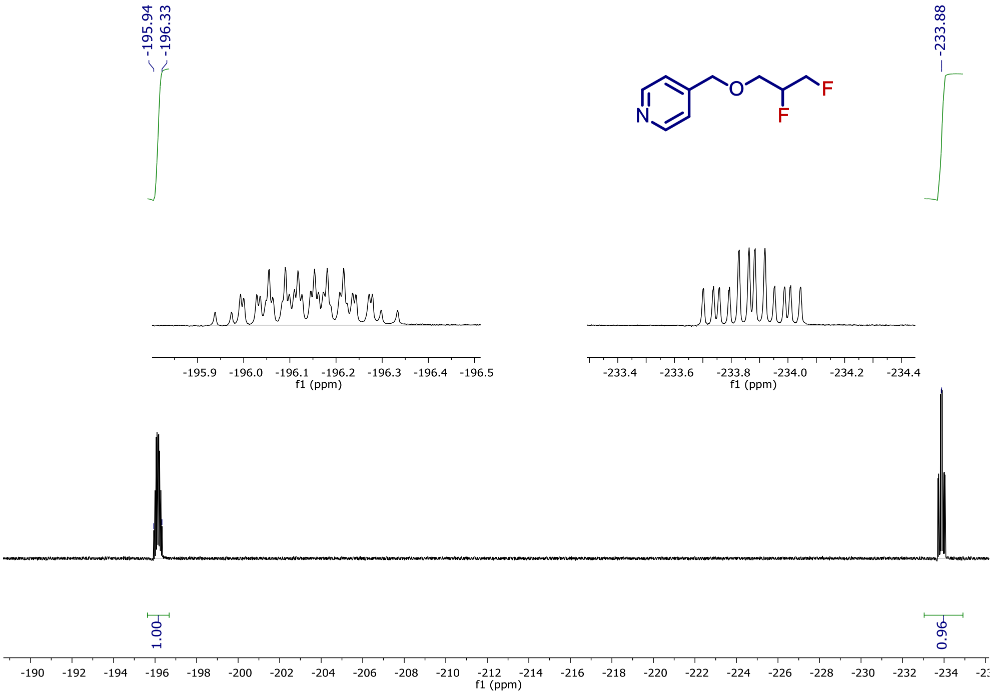

1-((2,3-difluoropropoxy)methyl)-3,5-dinitrobenzene, 3o

^{1}H NMR (500 MHz, CDCl3):

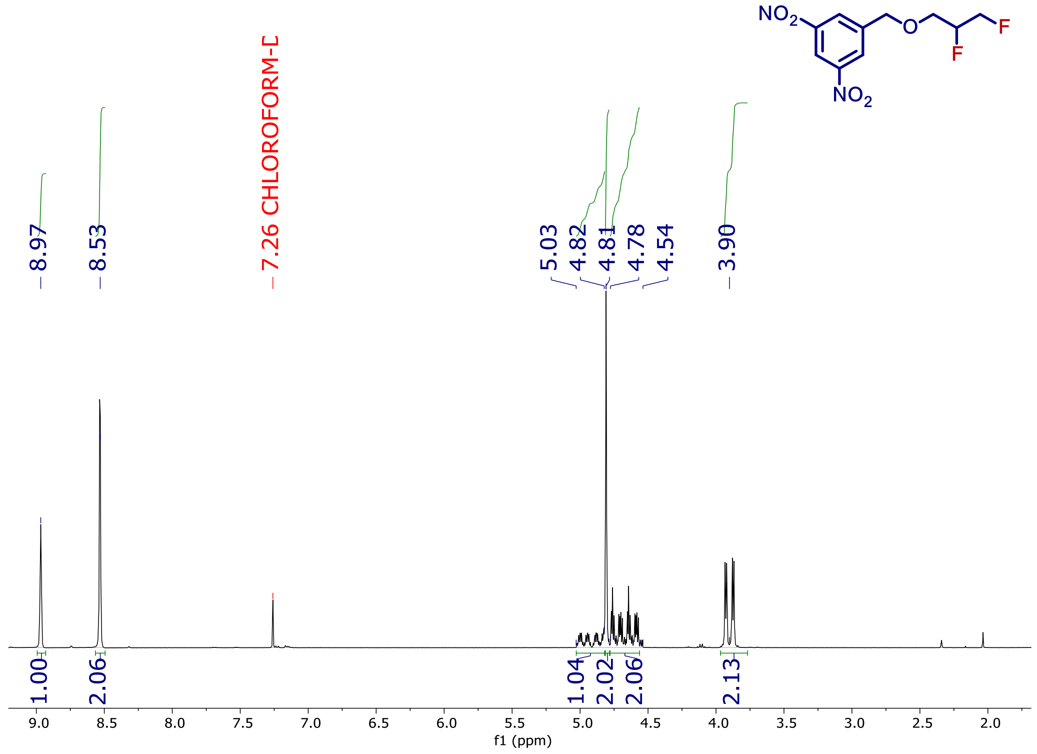

^{13}C NMR (126 MHz, CDCl3):

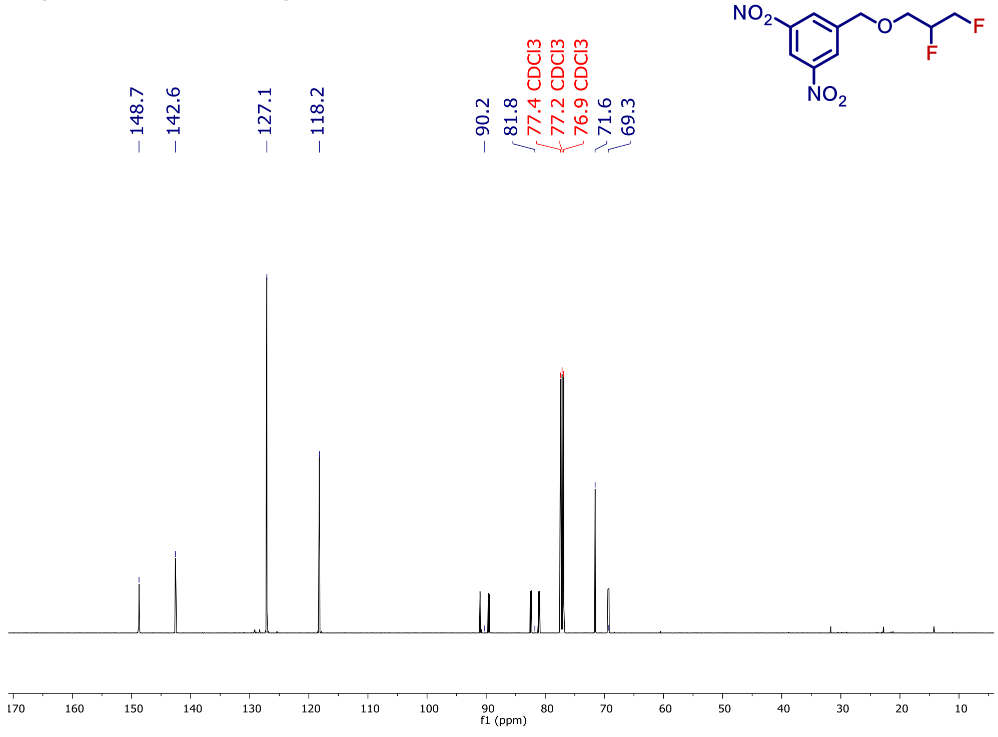

^{19}F NMR (376 MHz, $CDCl_3$):

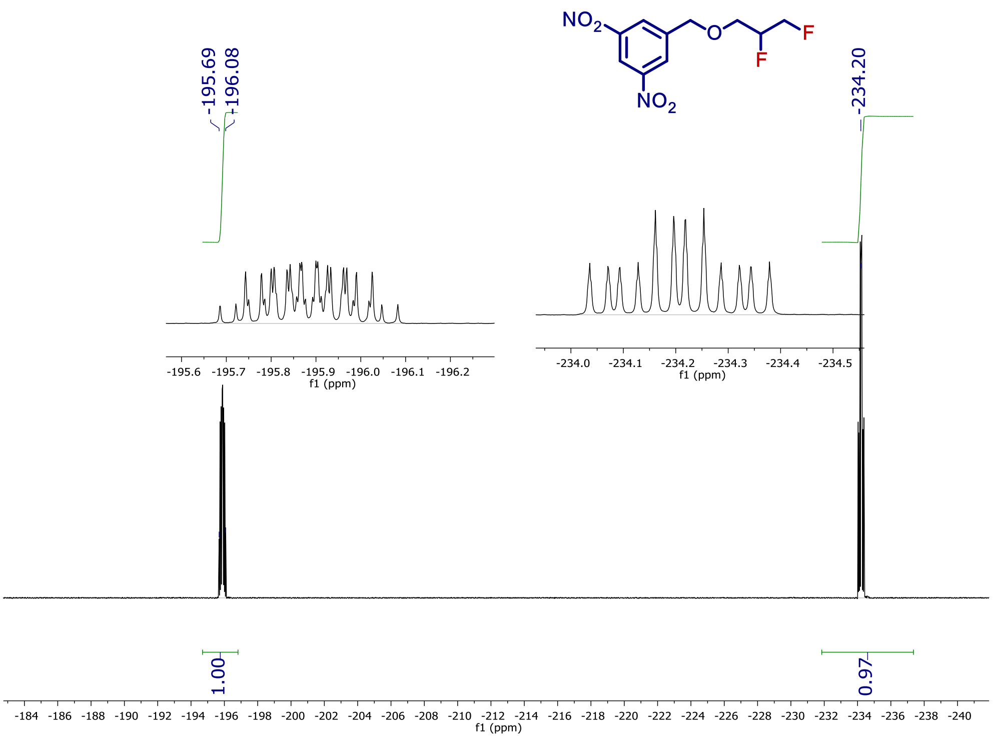

N-benzyl-N-(2,3-difluoropropyl)-4-methylbenzenesulfonamide, 3p

^{1}H NMR (500 MHz, $CDCl_3$):

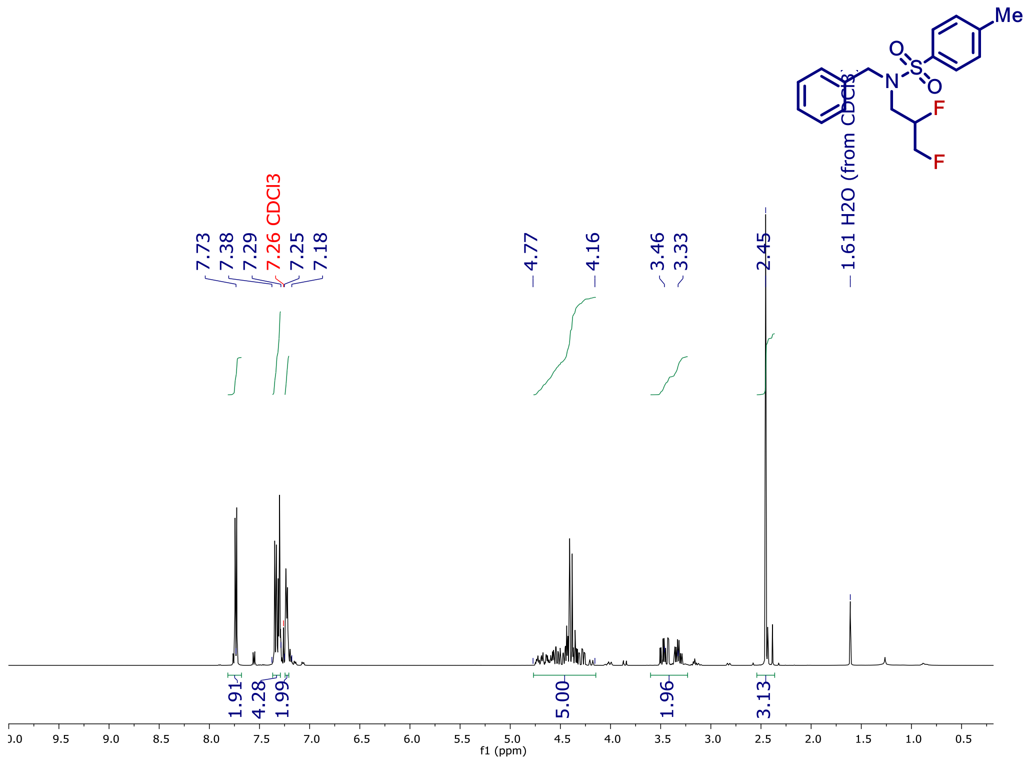

^{13}C NMR (126 MHz, $CDCl_3$):

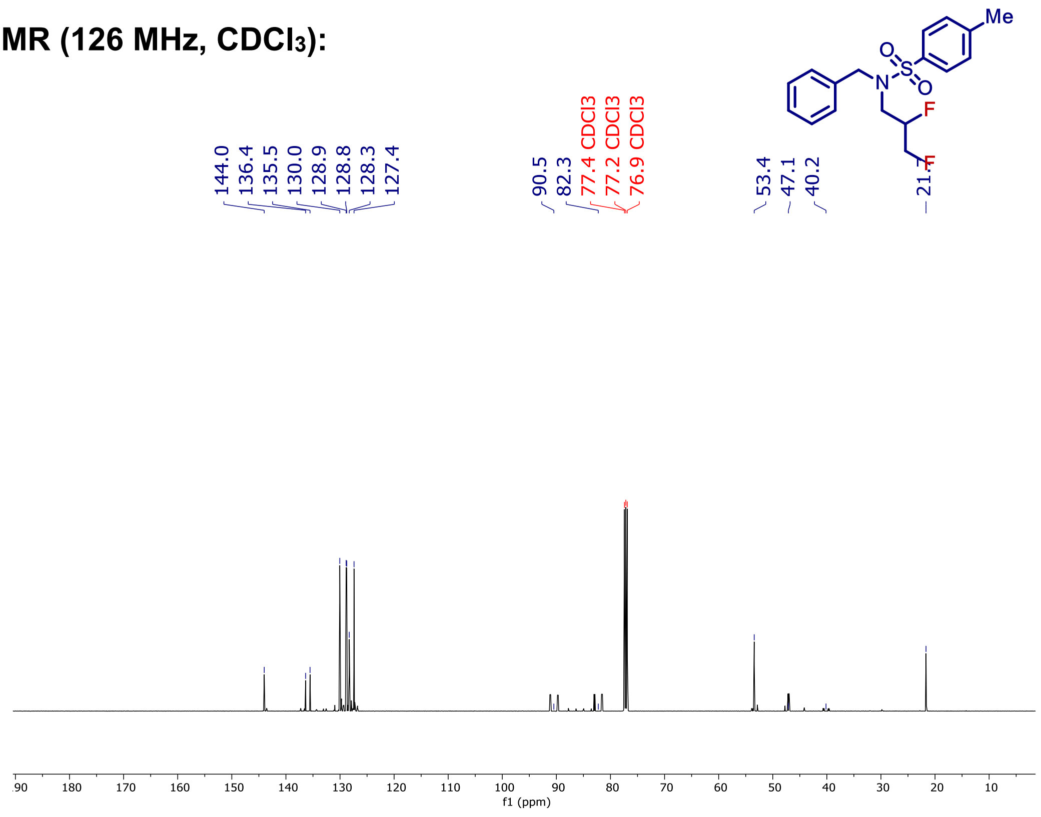

^{19}F NMR (376 MHz, CDCl3):

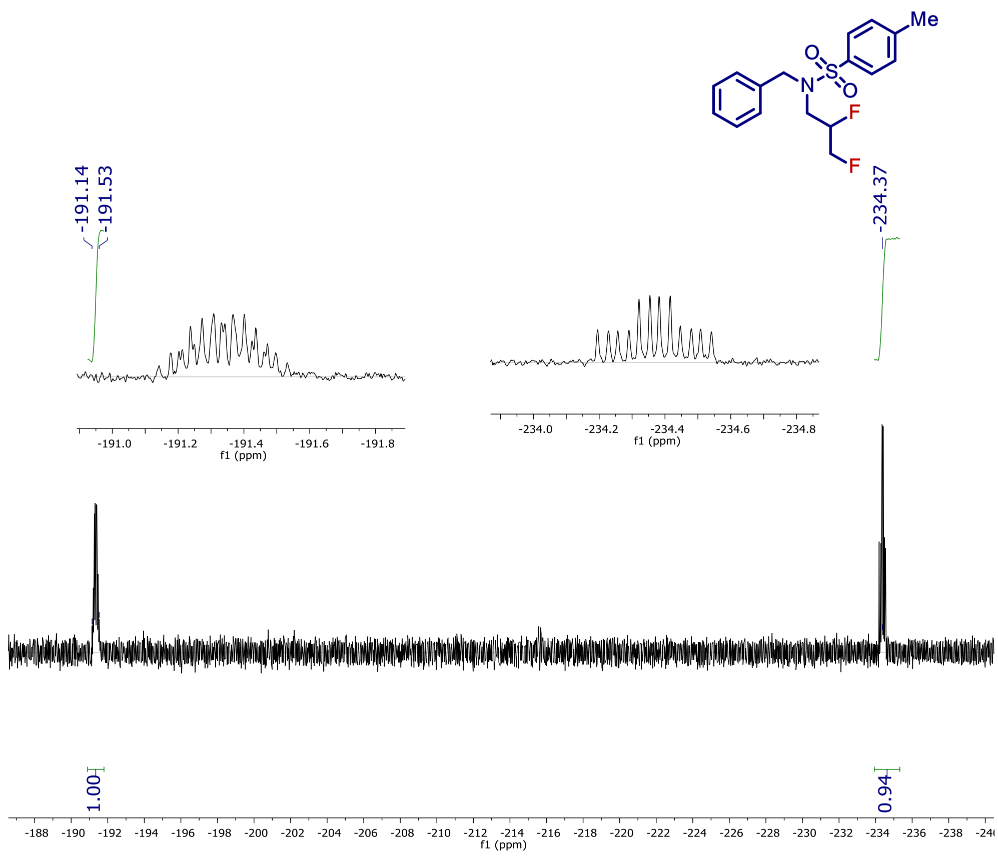

N-(4-chlorobenzyl)-N-(2,3-difluoropropyl)-4-methylbenzenesulfonamide, 3q

^{1}H NMR (500 MHz, CDCl$_3$):

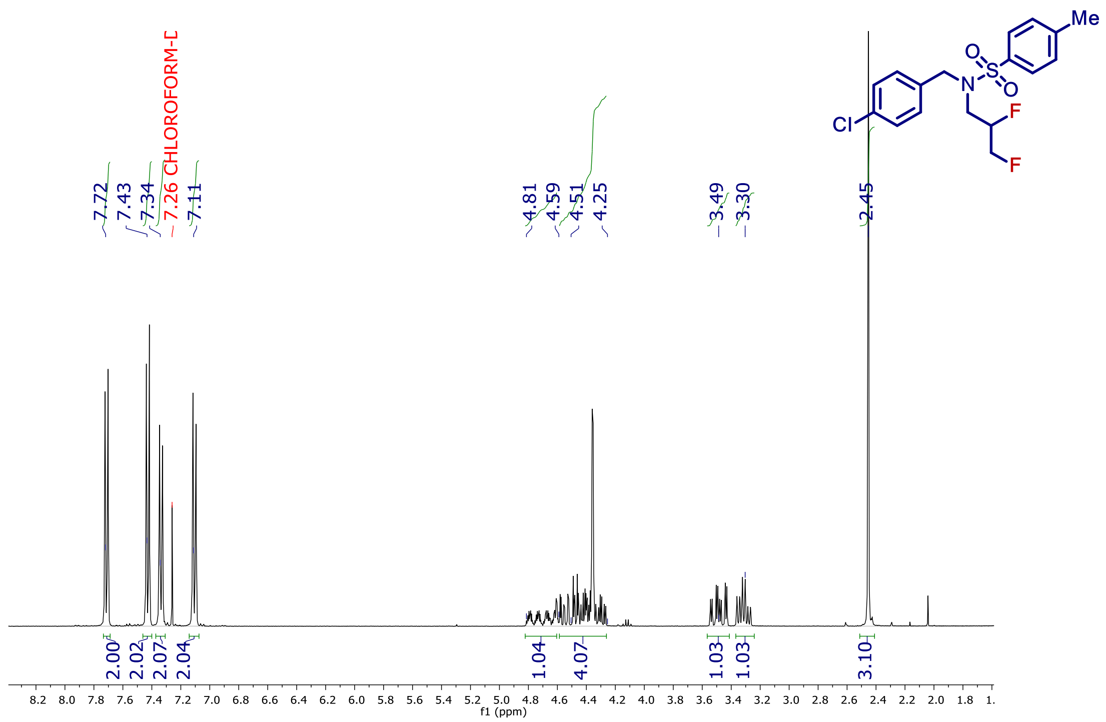

^{13}C NMR (126 MHz, CDCl$_3$):

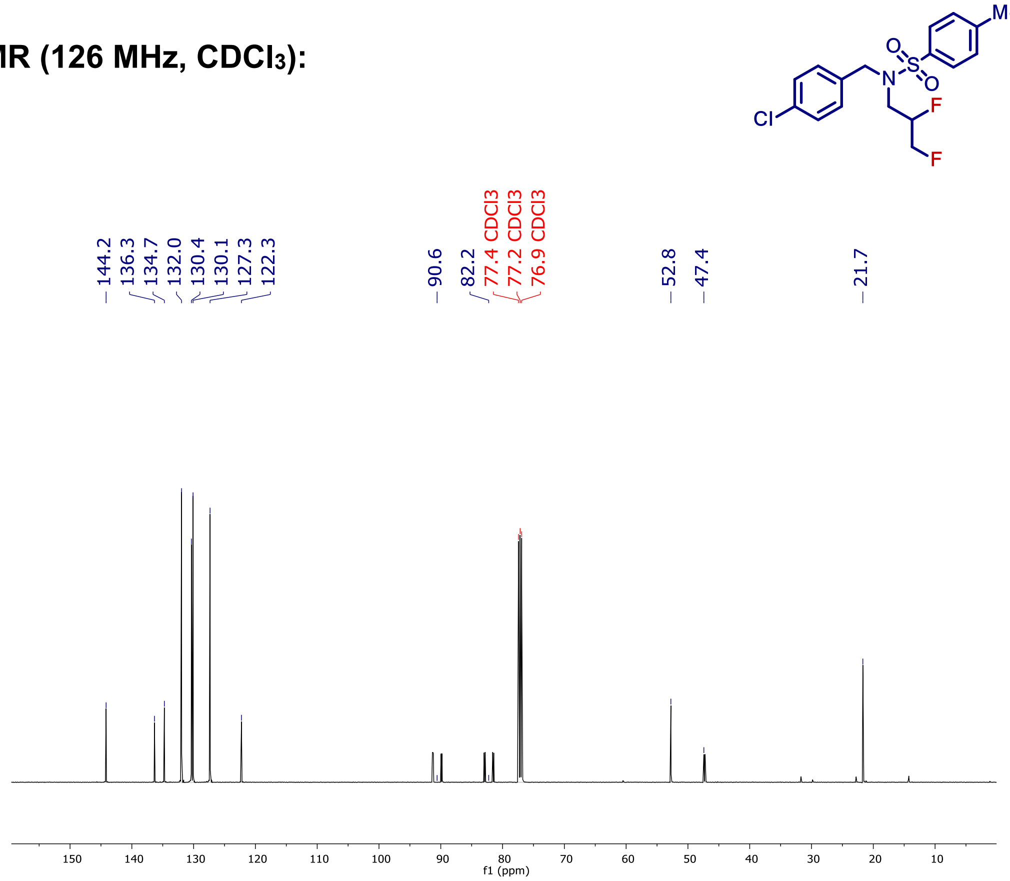

^{19}F NMR (376 MHz, CDCl$_3$):

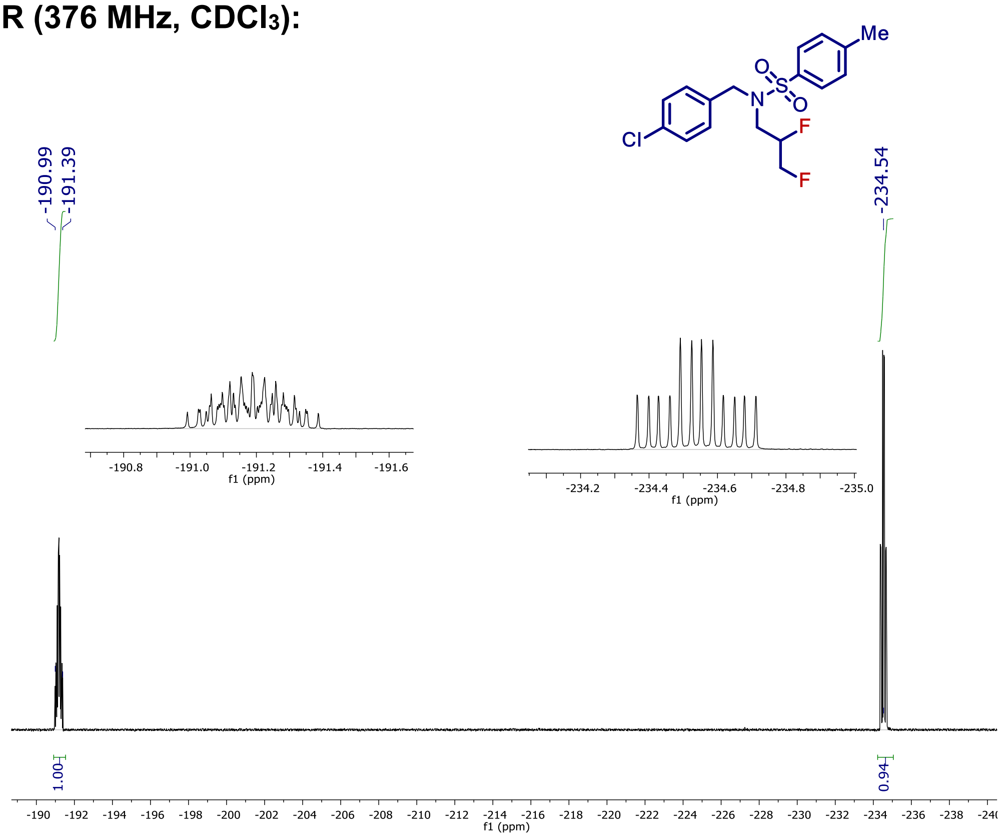

***N*-(2,3-difluoropropyl)-4-methyl-*N*-(4-(trifluoromethyl)benzyl) benzenesulfonamide, 3r**

^{1}H NMR (500 MHz, $CDCl_3$):

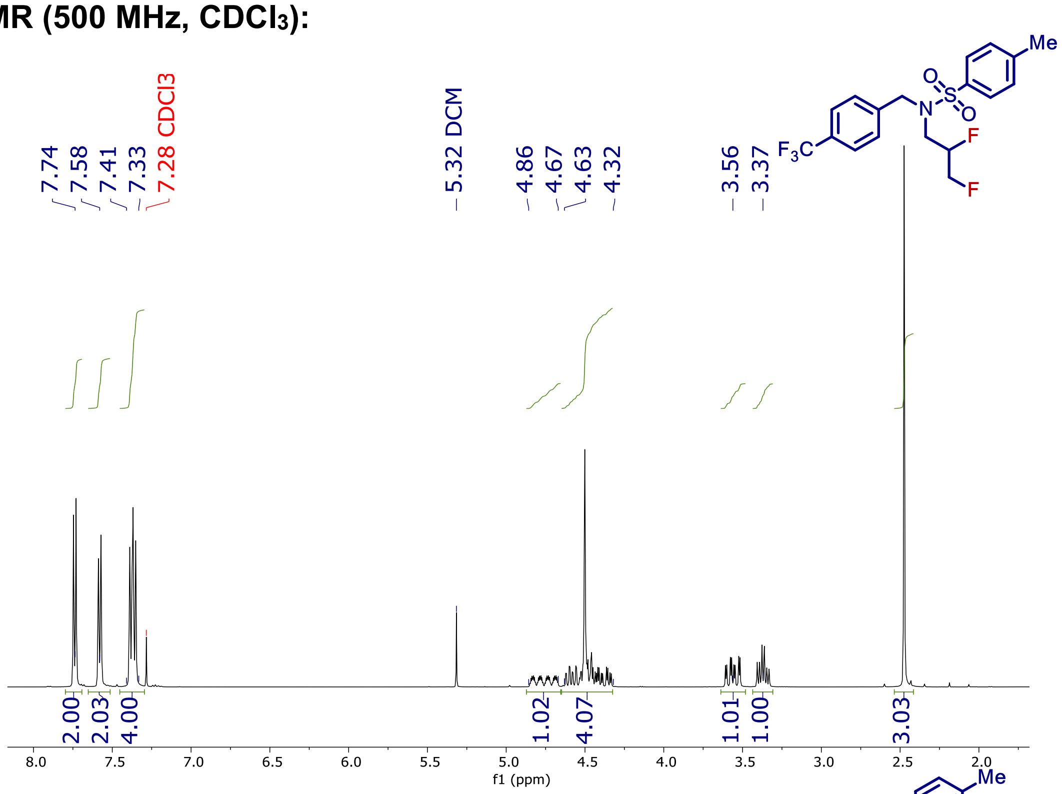

^{13}C NMR (126 MHz, $CDCl_3$):

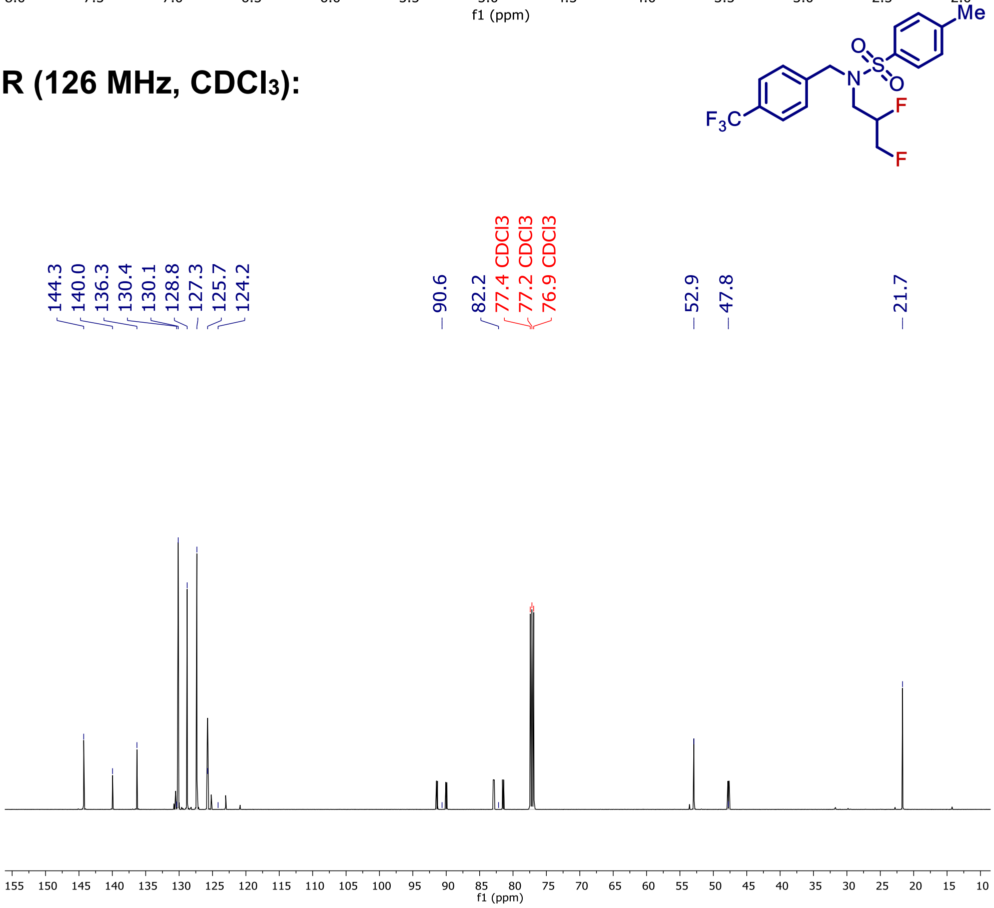

^{19}F NMR (376 MHz, CDCl$_3$):

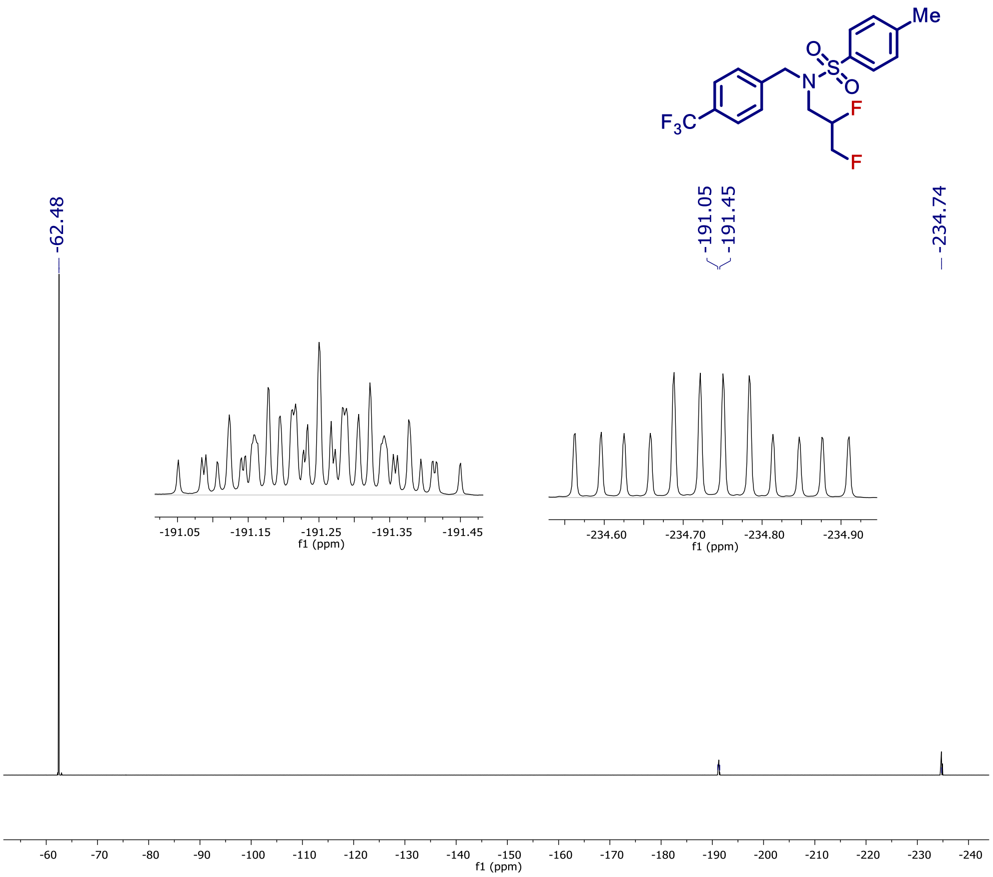

N-(4-bromobenzyl)-N-(2,3-difluoropropyl)-4-methylbenzenesulfonamide, 3s

^{1}H NMR (500 MHz, $CDCl_3$):

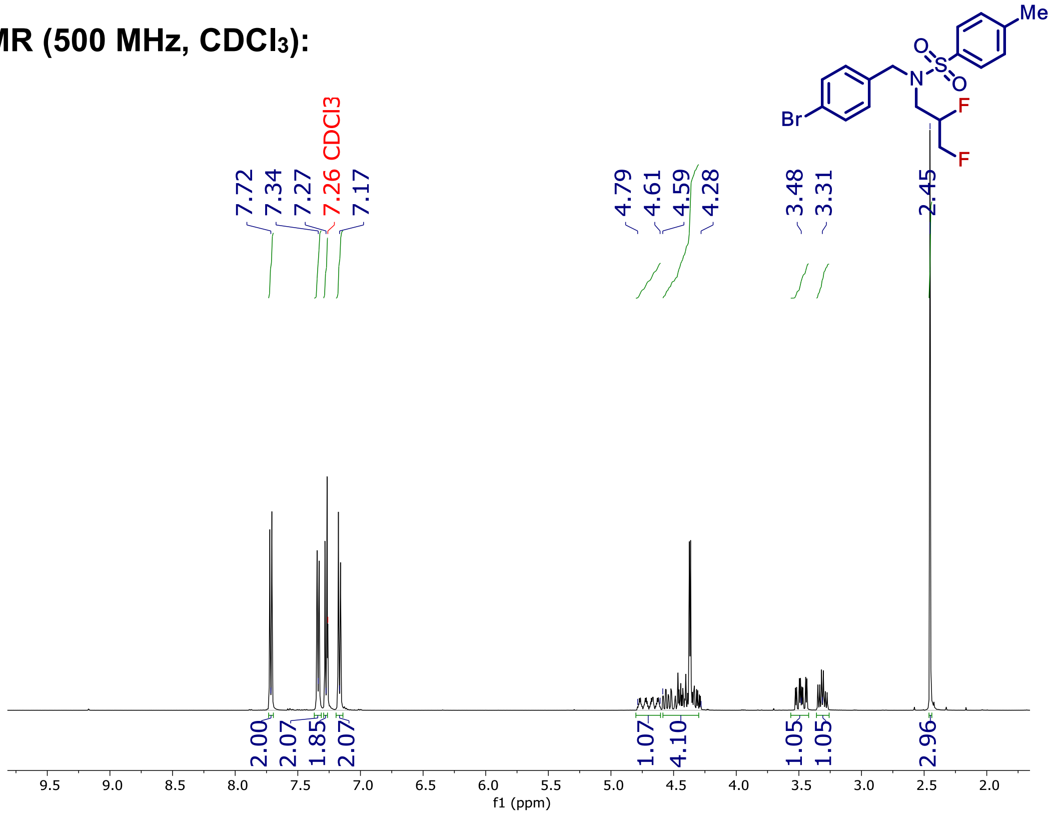

^{13}C NMR (126 MHz, $CDCl_3$):

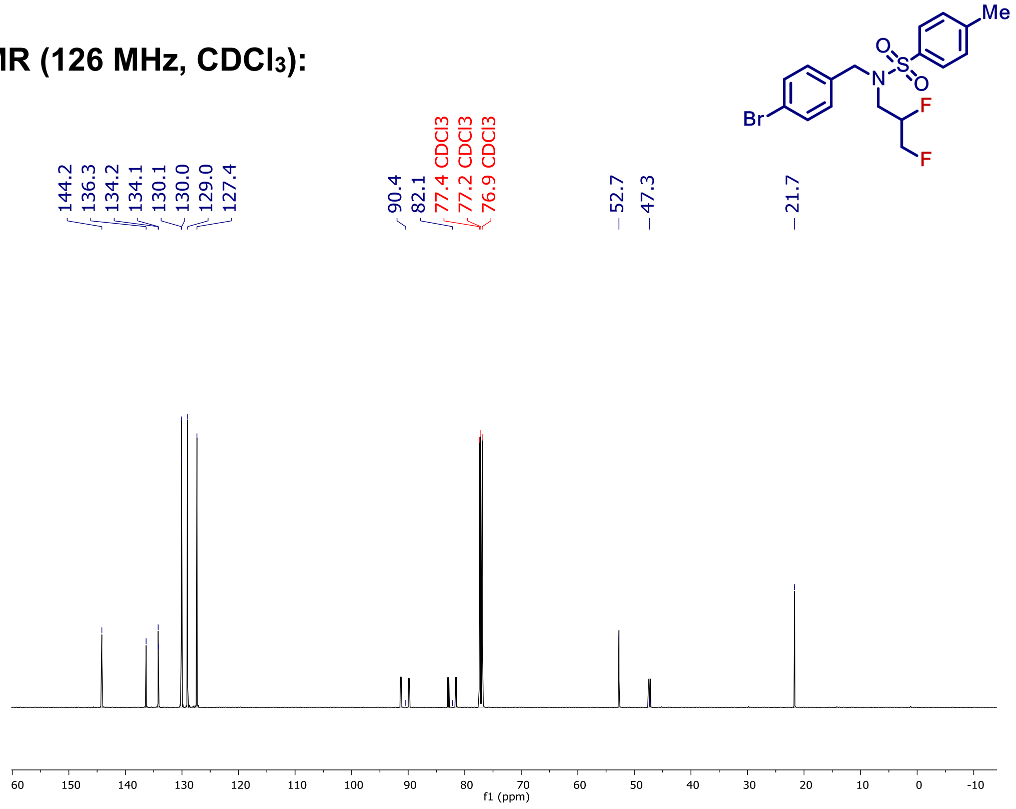

^{19}F NMR (376 MHz, CDCl$_3$):

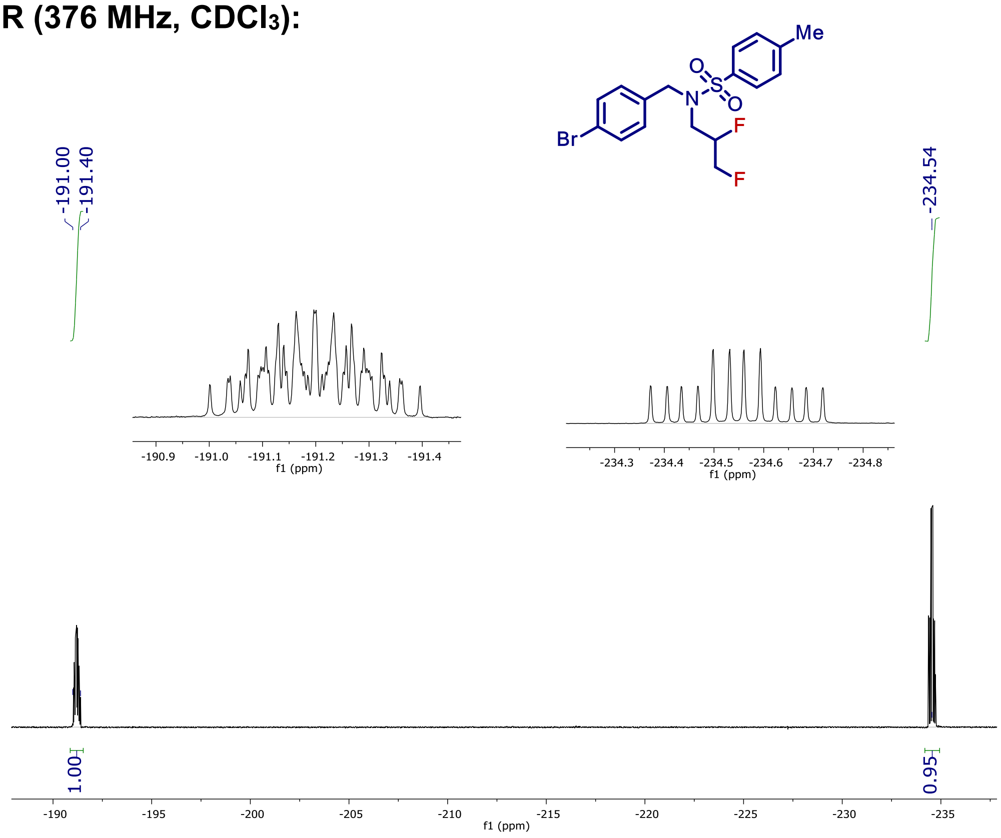

N-cyclopropyl-N-(2,3-difluoropropyl)-4-methylbenzenesulfonamide, 3t

^{1}H NMR (500 MHz, $CDCl_3$):

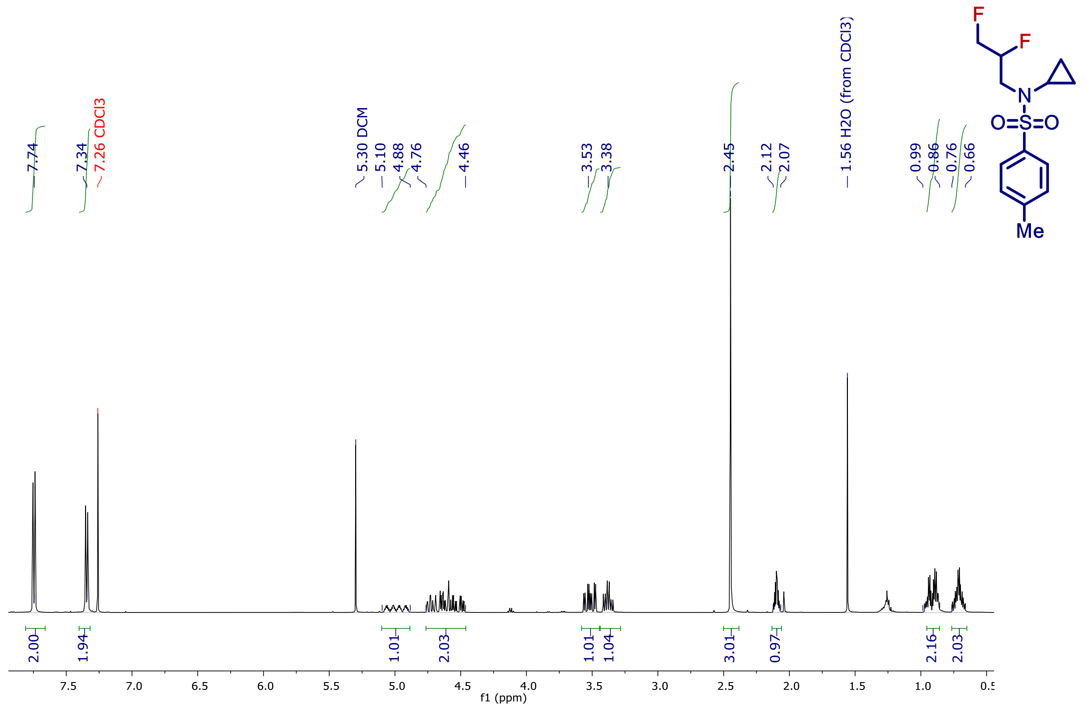

^{13}C NMR (126 MHz, $CDCl_3$):

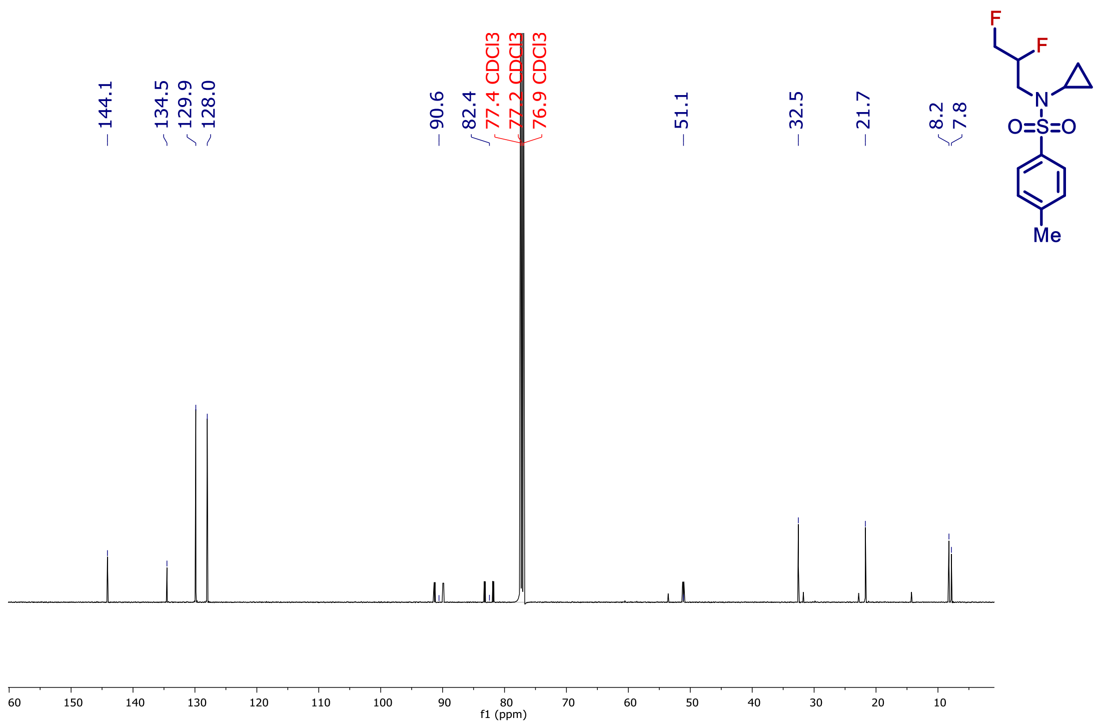

^{19}F NMR (376 MHz, $CDCl_3$):

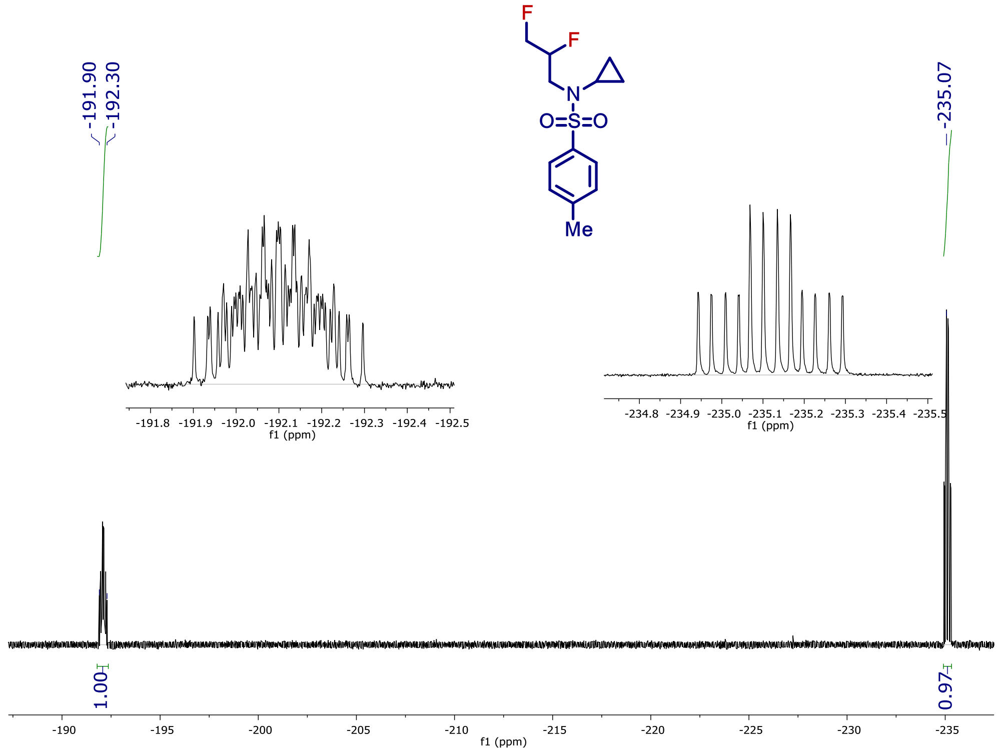

1-(2,3-difluoropropyl)-4-methylbenzene, 3u

^{1}H NMR (500 MHz, $CDCl_3$):

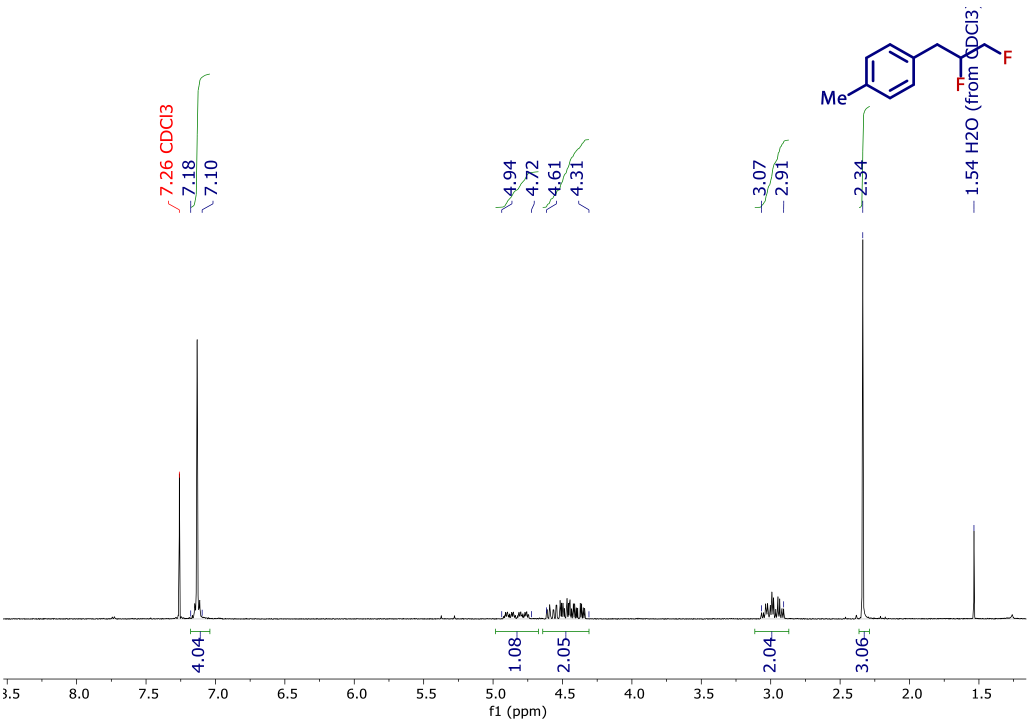

^{13}C NMR (126 MHz, $CDCl_3$):

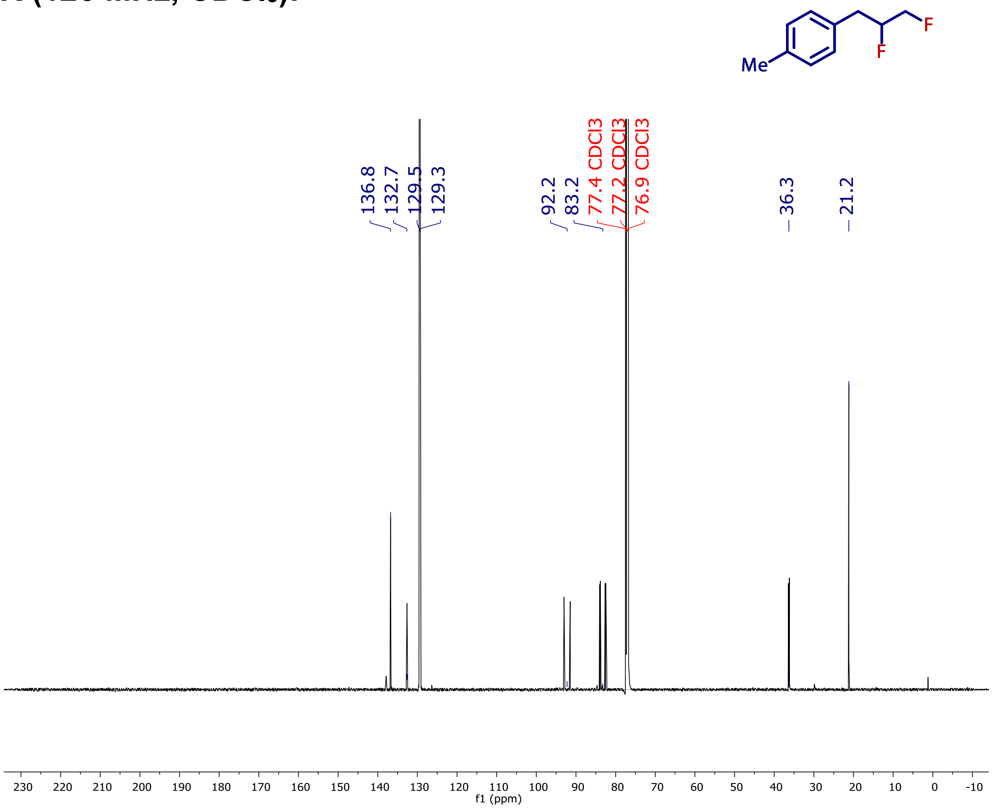

^{19}F NMR (376 MHz, $CDCl_3$):

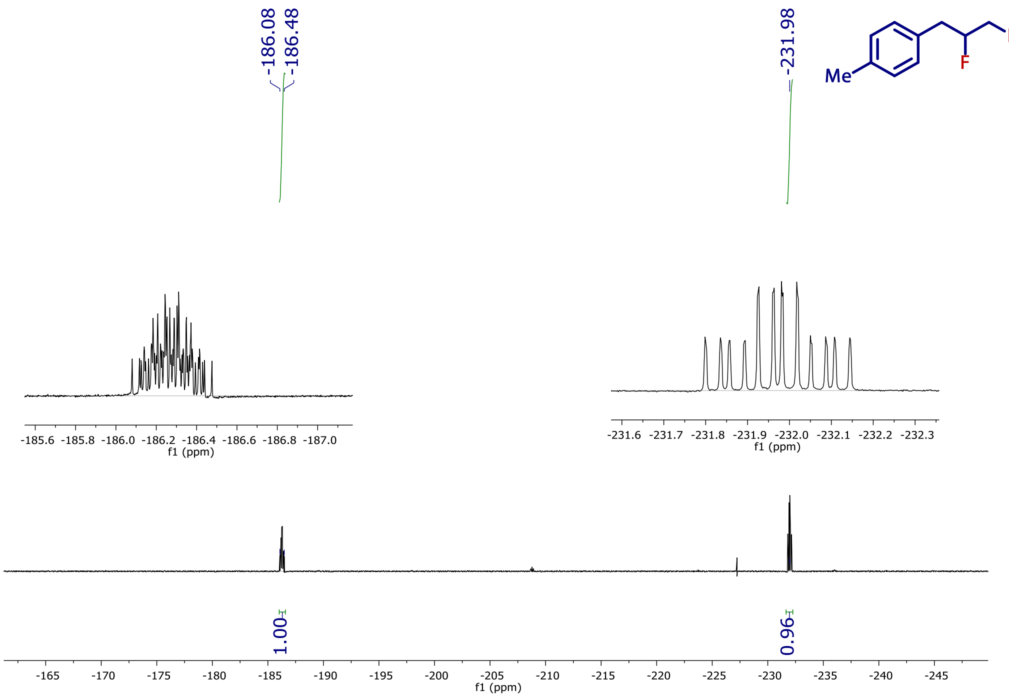

1-(4-(2,3-difluoropropyl)phenyl)-N,N-dimethylmethanamine, 3v

^{1}H NMR (500 MHz, $CDCl_3$):

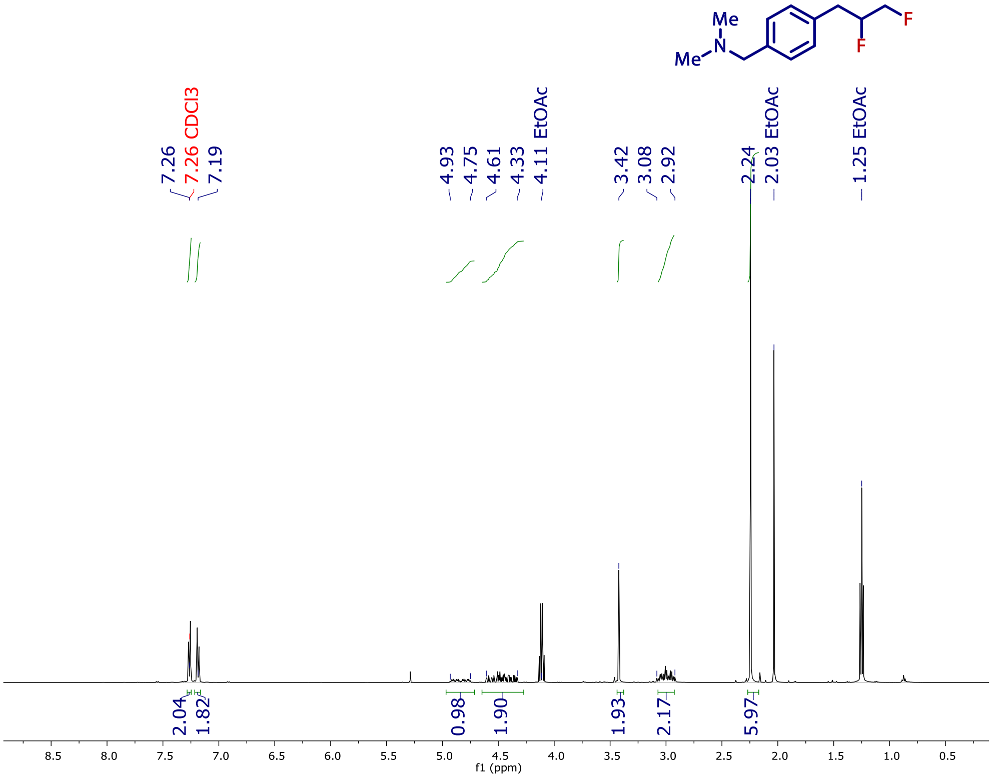

^{13}C NMR (126 MHz, $CDCl_3$):

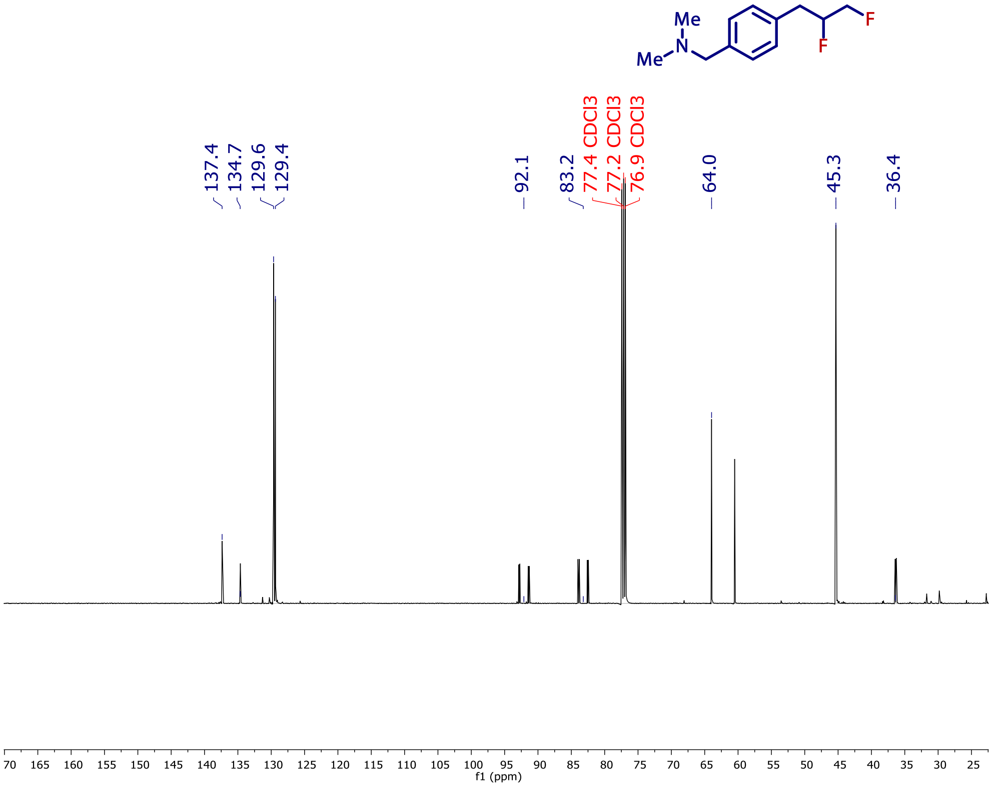

^{19}F NMR (376 MHz, CDCl$_3$):

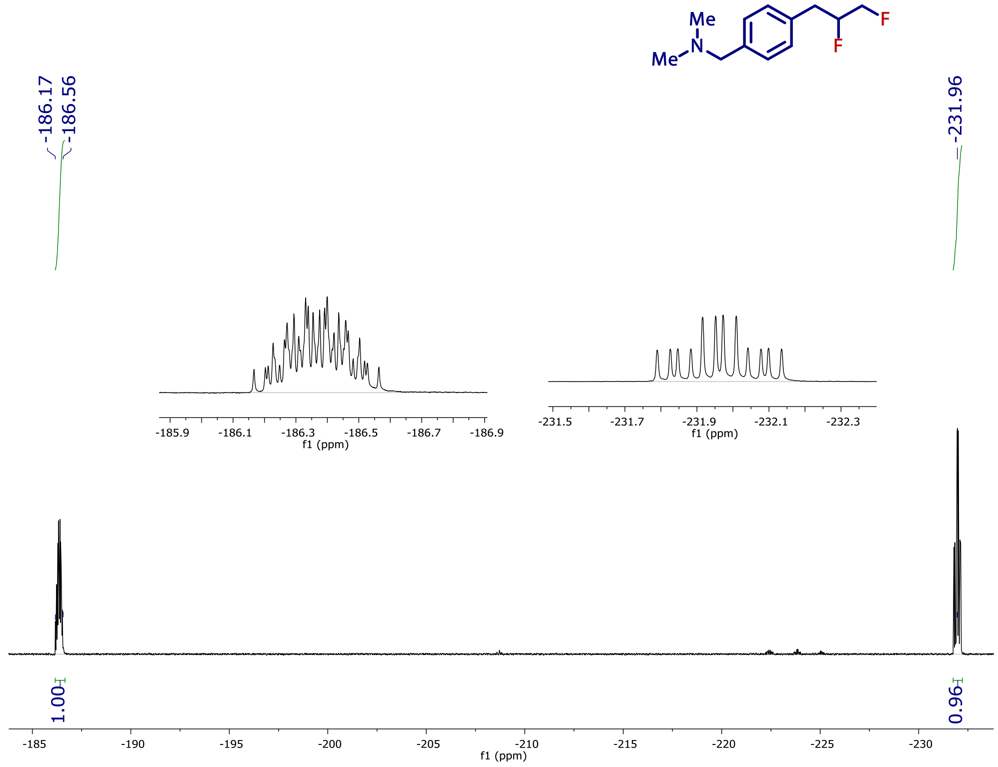

4-(2,3-difluoropropyl)-N,N-dimethylaniline, 3w

^{1}H NMR (500 MHz, CDCl$_3$):

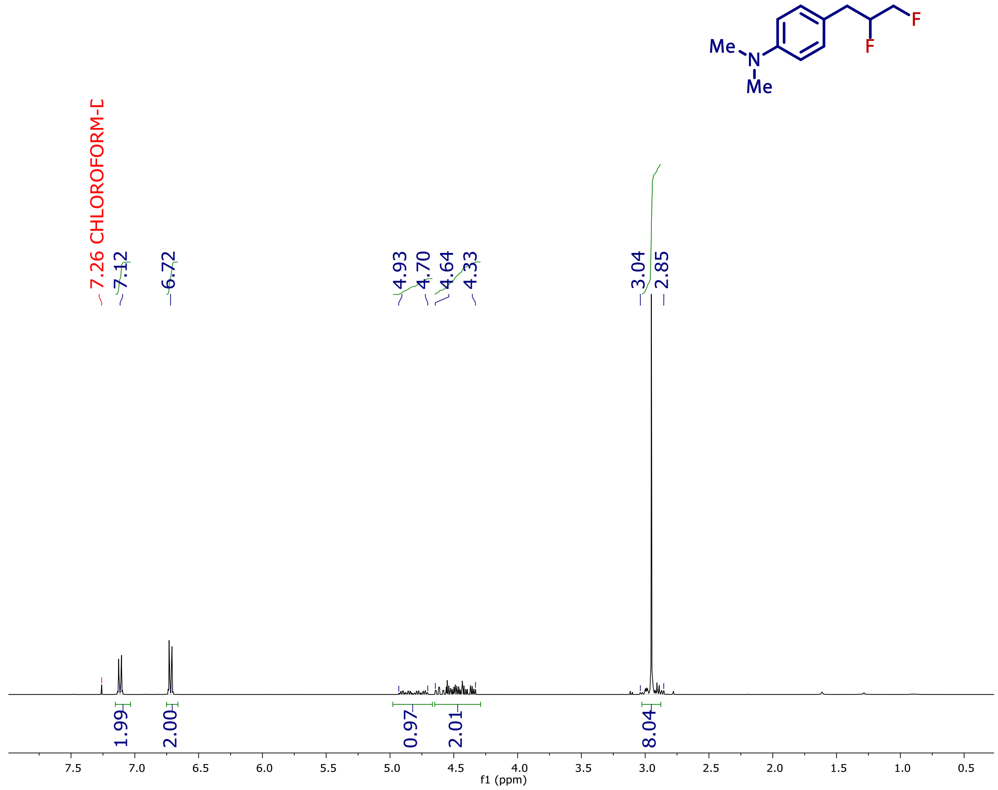

^{13}C NMR (126 MHz, CDCl$_3$):

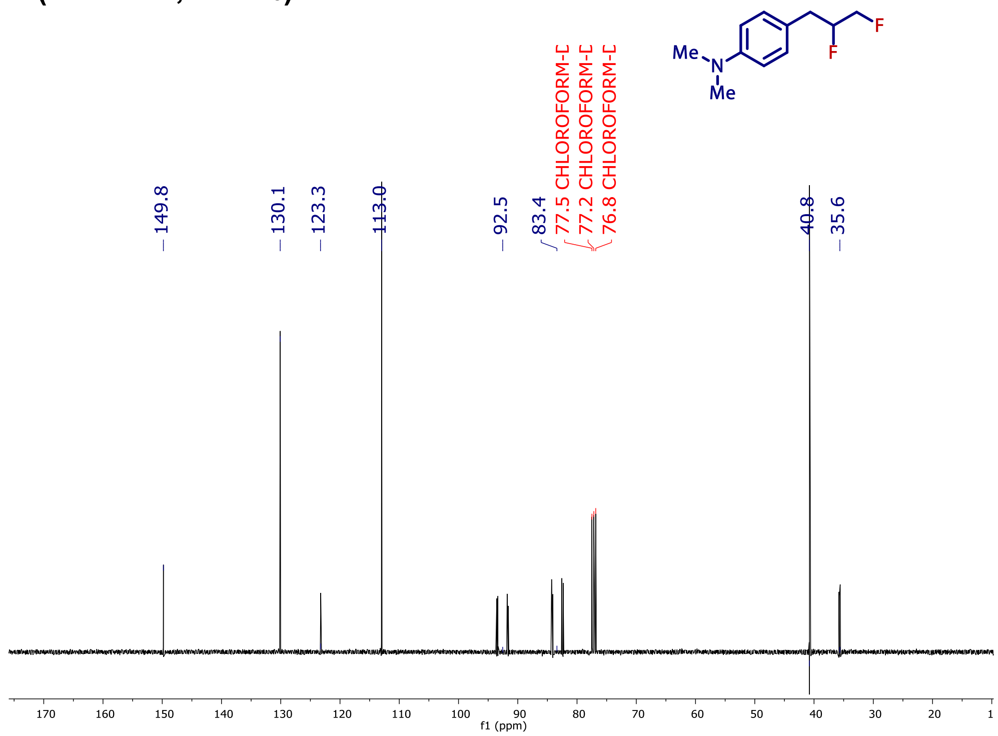

^{19}F NMR (376 MHz, CDCl$_3$):

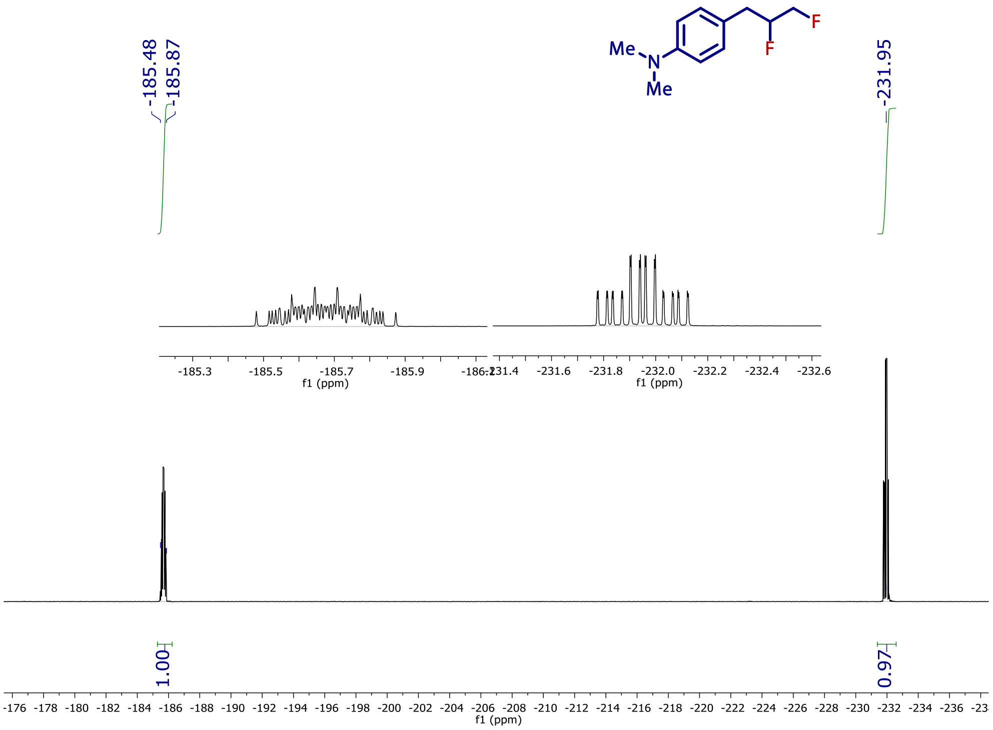

2-(2,3-difluoropropyl)-1,3,5-trimethylbenzene, 3x

^{1}H NMR (500 MHz, $CDCl_3$):

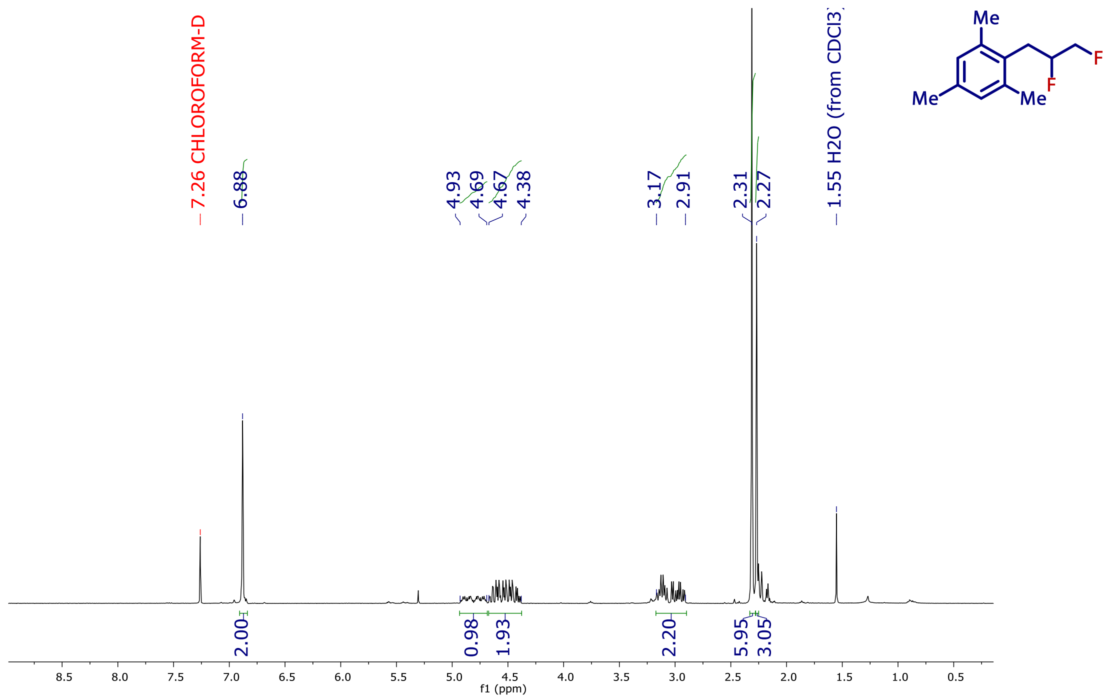

^{13}C NMR (126 MHz, $CDCl_3$):

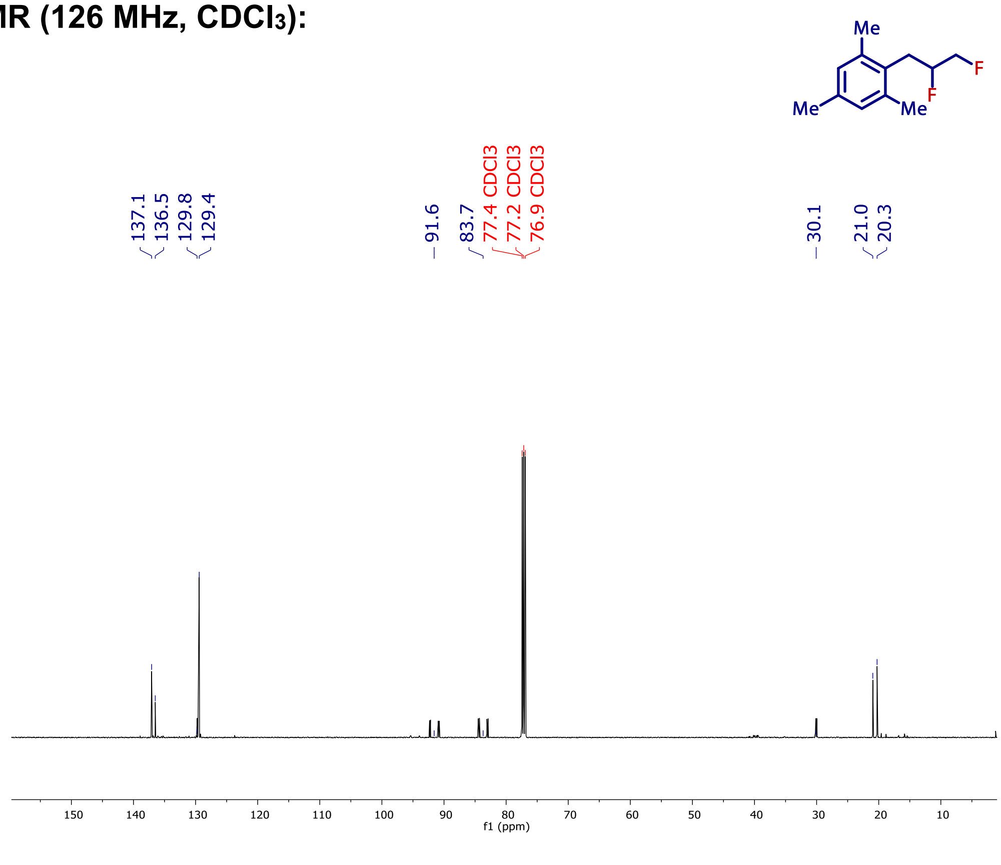

^{19}F NMR (376 MHz, $CDCl_3$):

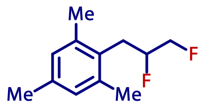

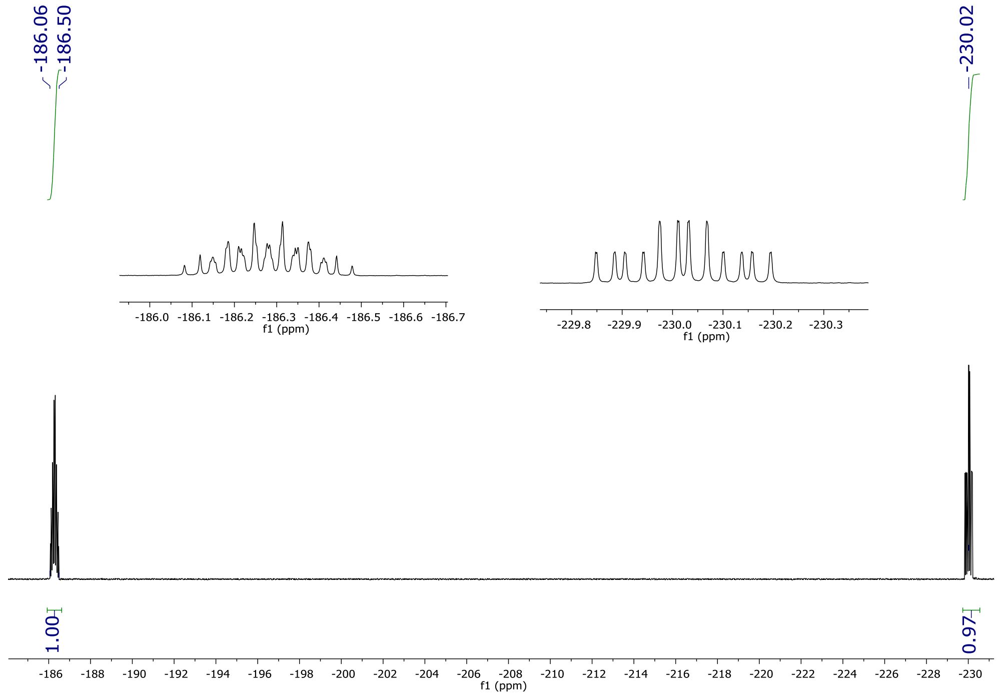

1-(2,3-difluoropropyl)-4-methoxybenzene, 3y

^{1}H NMR (500 MHz, CDCl$_3$):

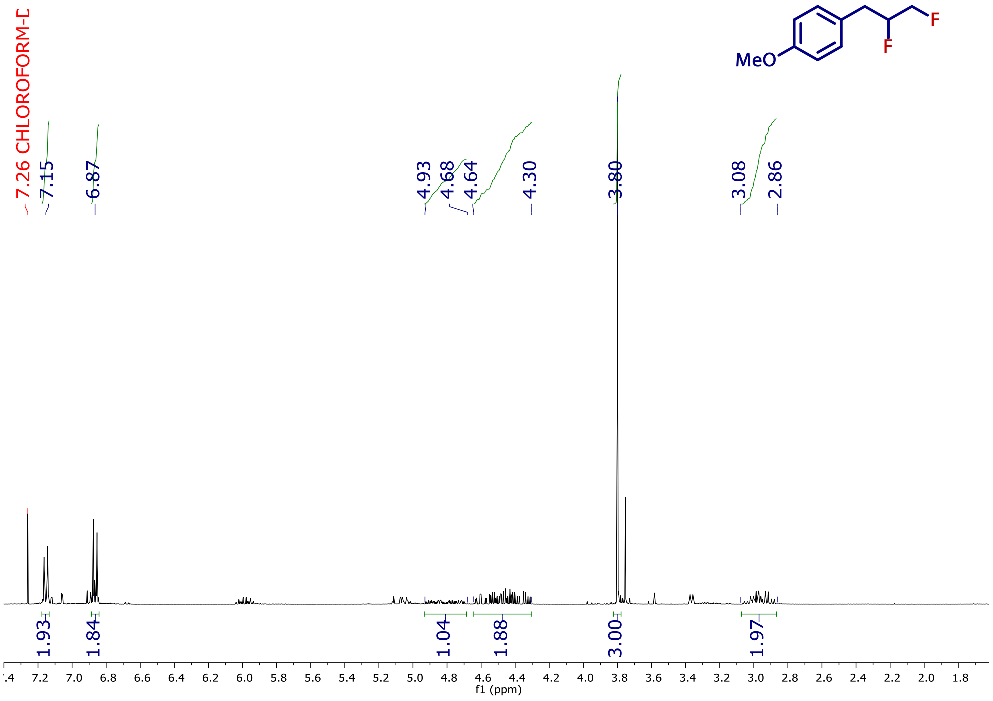

^{13}C NMR (126 MHz, CDCl$_3$):

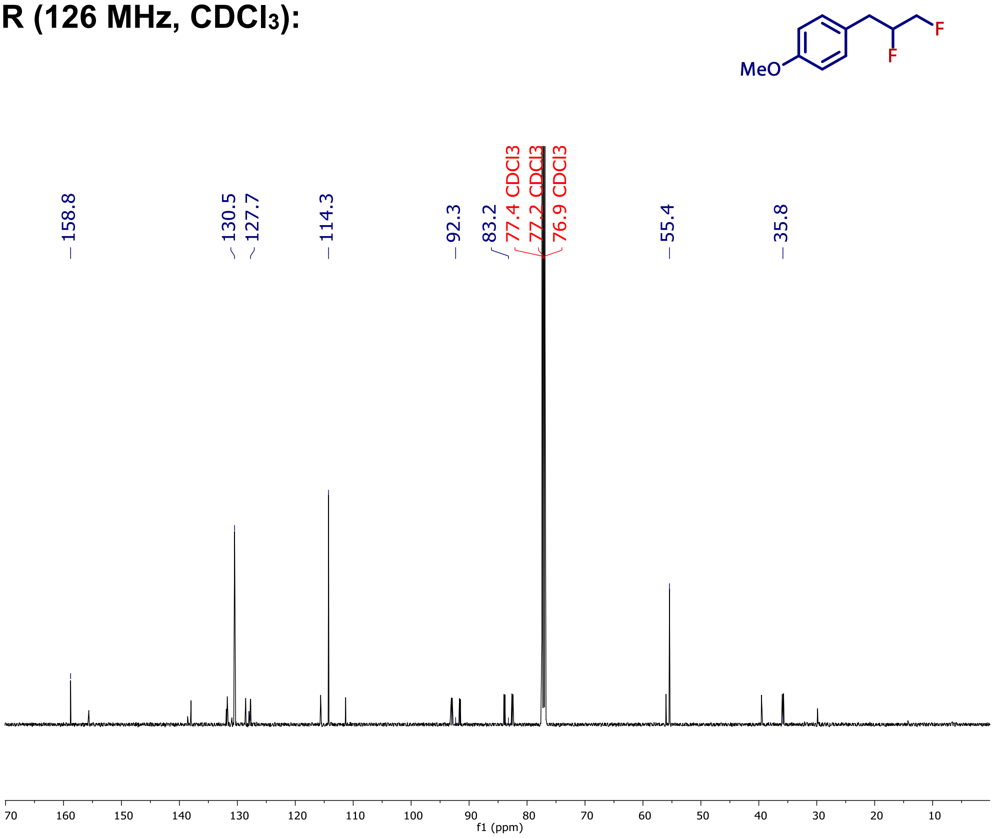

^{19}F NMR (376 MHz, CDCl$_3$):

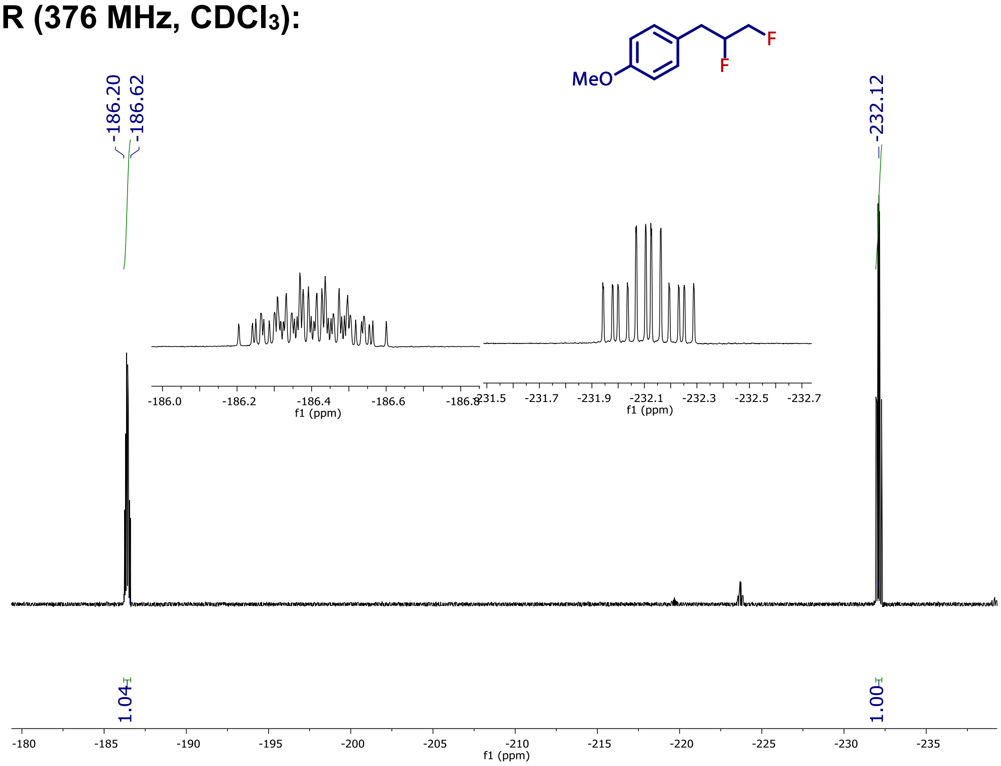

3,4-difluoro-1-morpholinobutan-1-one, 3z

^{1}H NMR (500 MHz, CDCl$_3$):

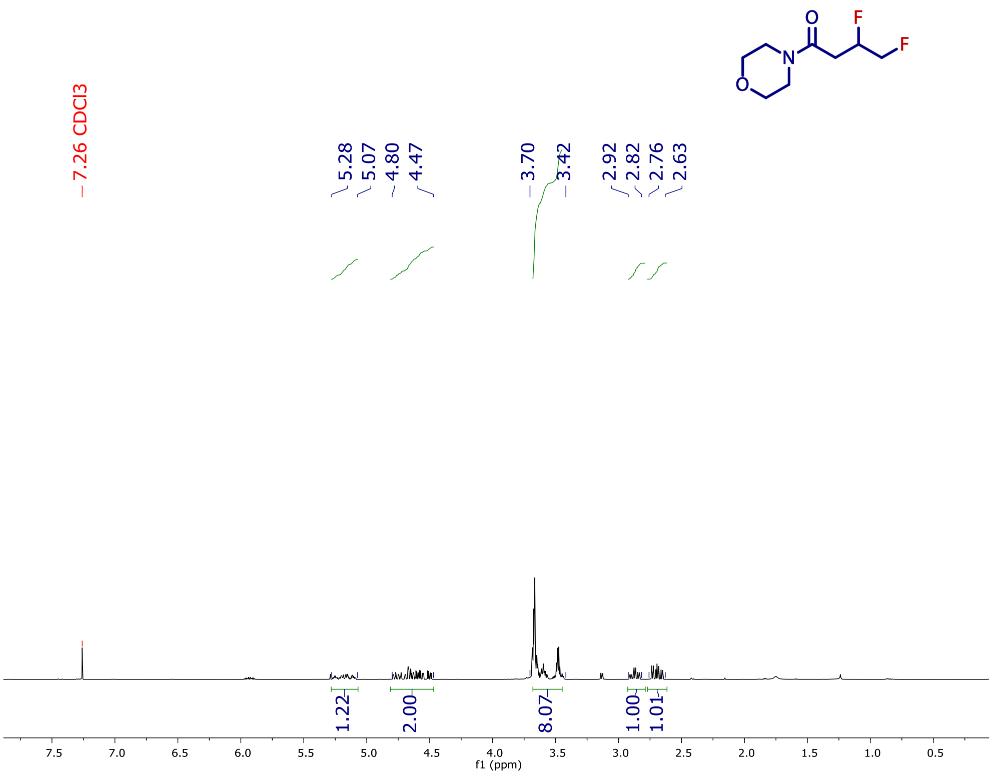

^{13}C NMR (126 MHz, CDCl$_3$):

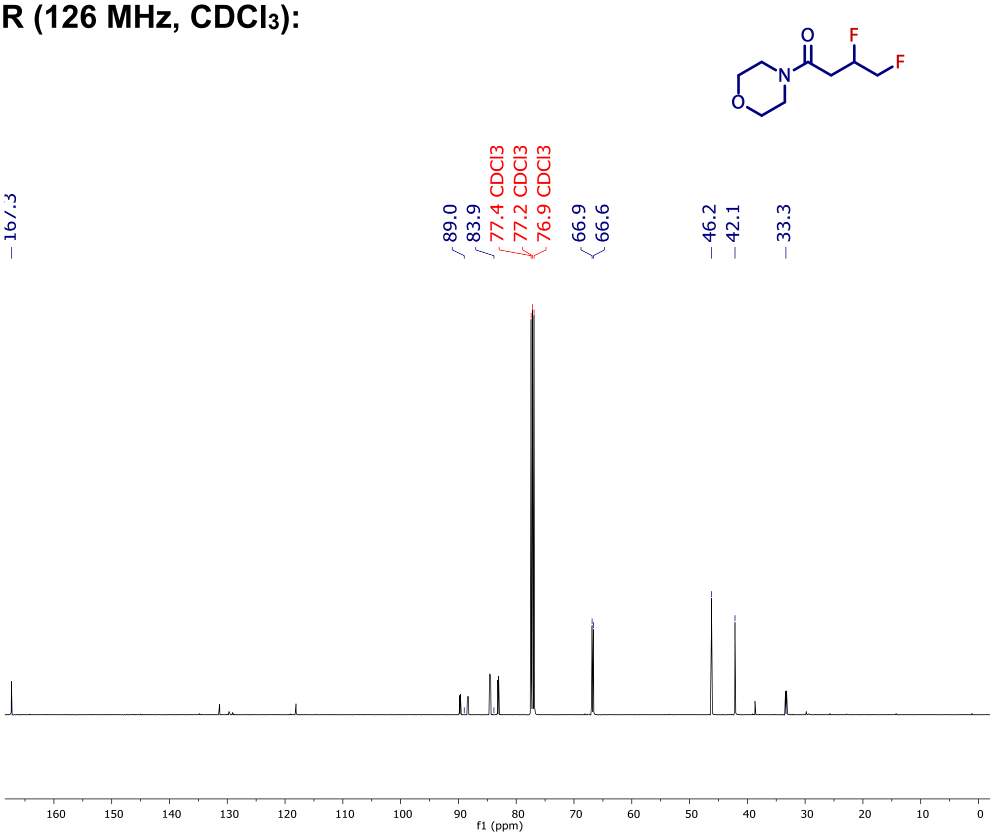

^{19}F NMR (376 MHz, $CDCl_3$):

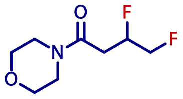

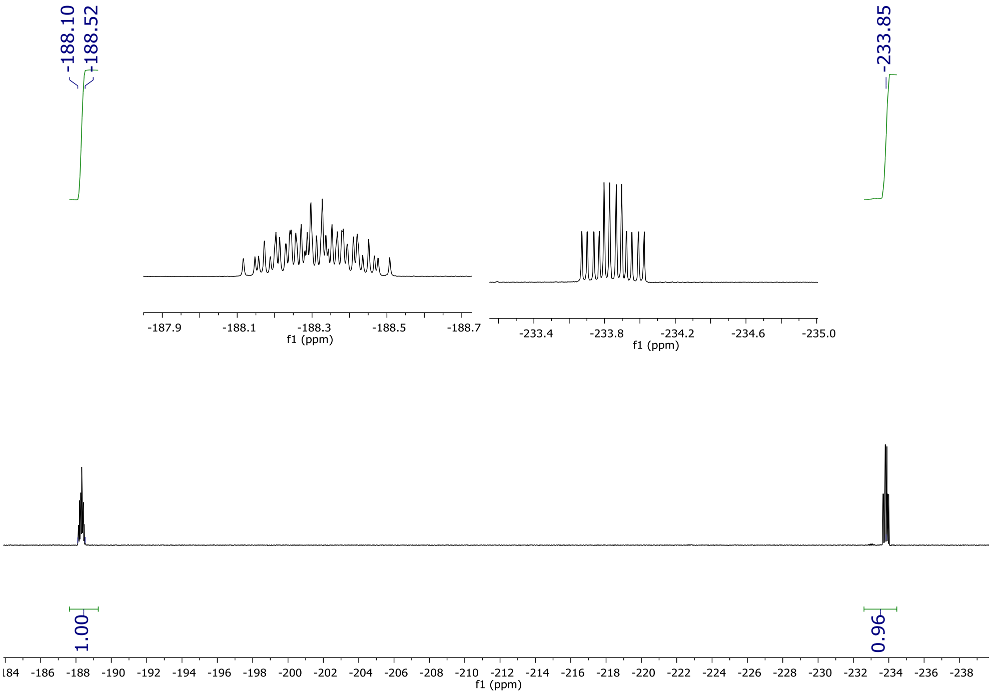

N-(2,3-difluoropropyl)-N-(4-fluorophenyl)-4-methylbenzenesulfonamide, 3aa

^{1}H NMR (500 MHz, CDCl3):

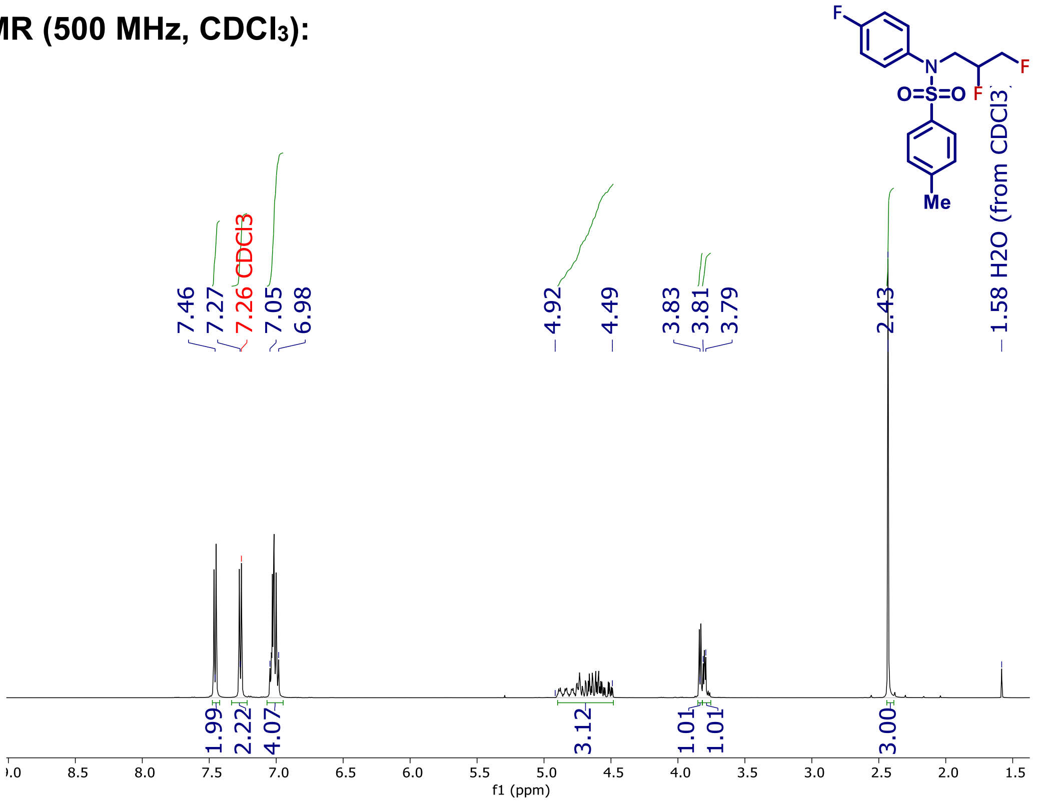

^{13}C NMR (126 MHz, CDCl3):

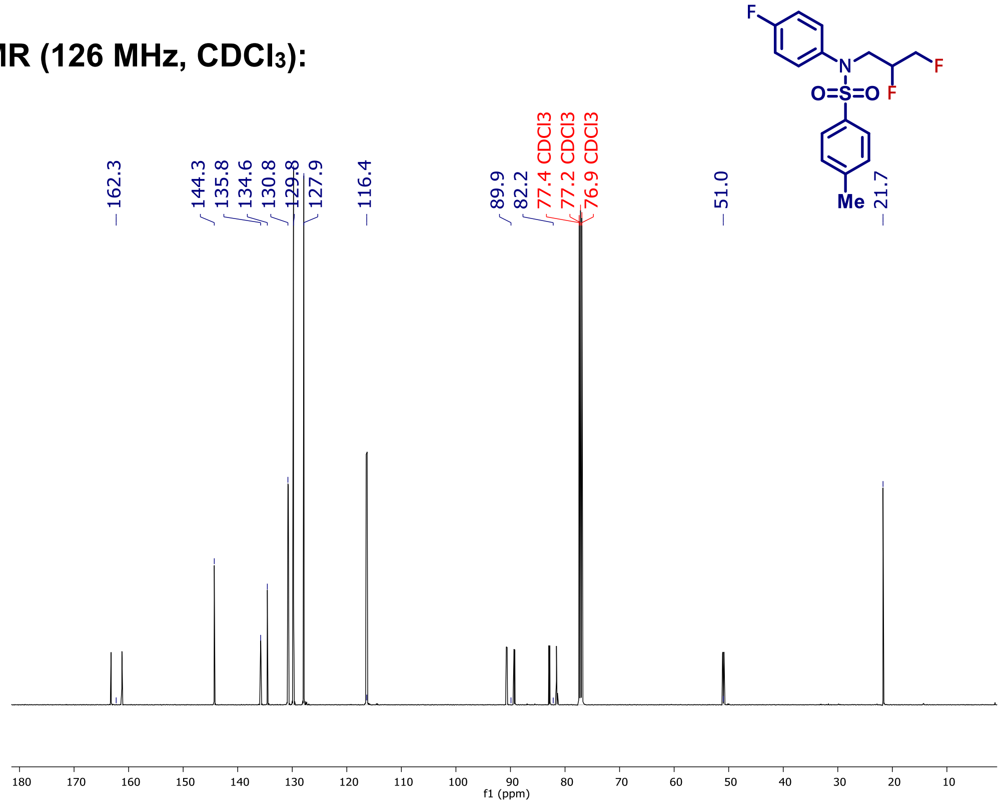

^{19}F NMR (376 MHz, $CDCl_3$):

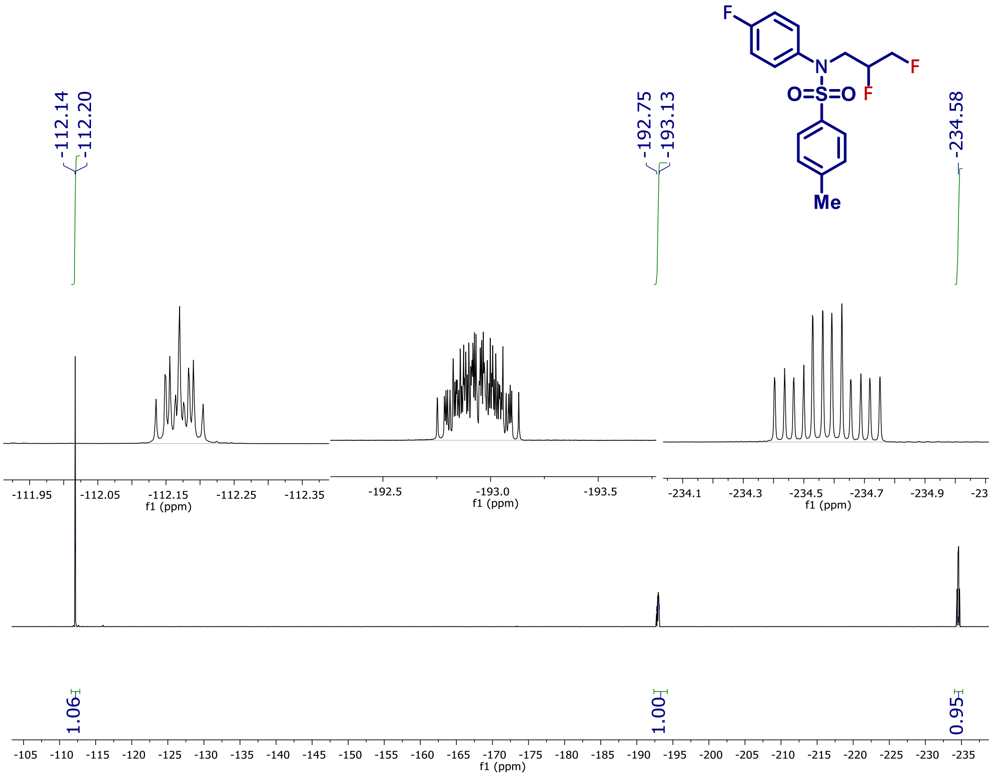

N-(2,3-difluoropropyl)-N-(4-fluorophenyl)-4-nitrobenzenesulfonamide, 3ab

^{1}H NMR (500 MHz, $CDCl_3$):

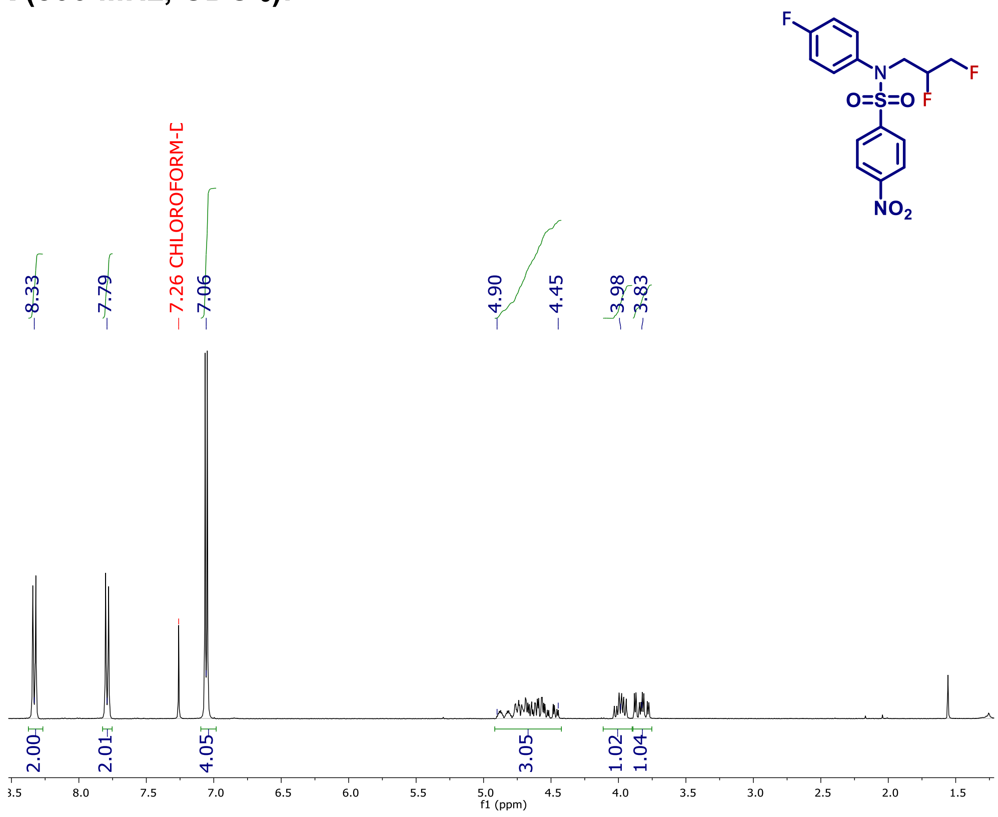

^{13}C NMR (126 MHz, $CDCl_3$):

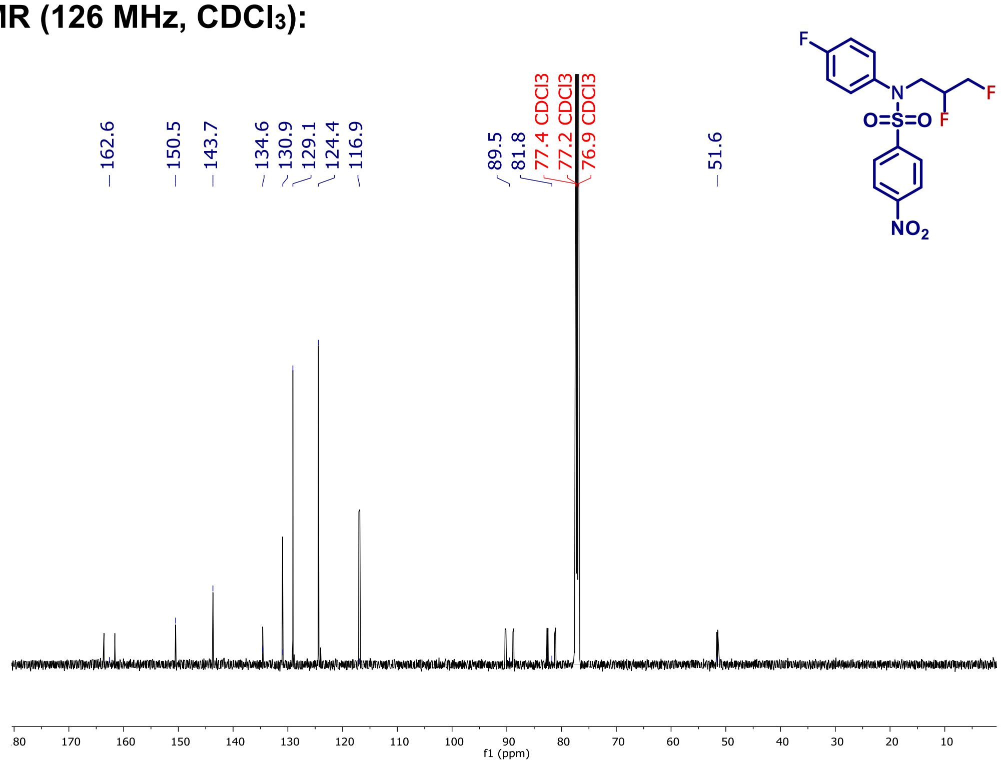

^{19}F NMR (376 MHz, CDCl$_3$):

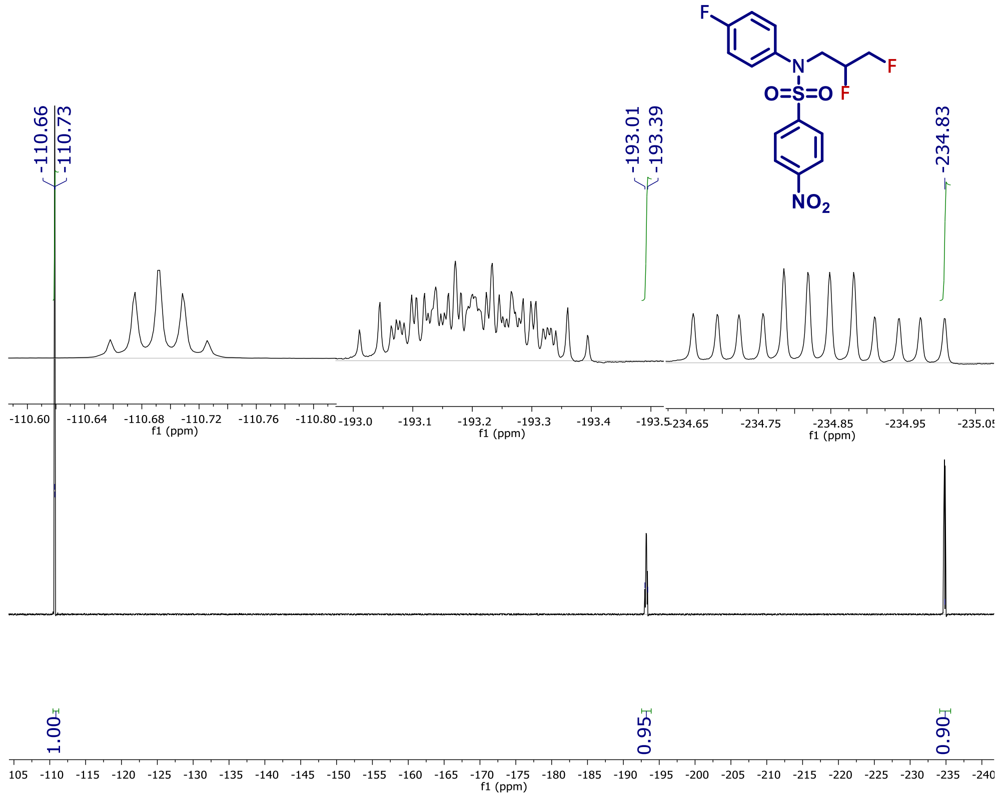

N-(2,3-difluoropropyl)-N-(4-fluoro-2-methylphenyl)-4-nitrobenzenesulfonamide, 3ac

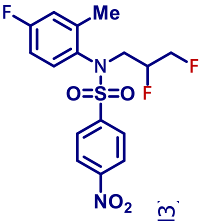

^{1}H NMR (500 MHz, CDCl$_3$):

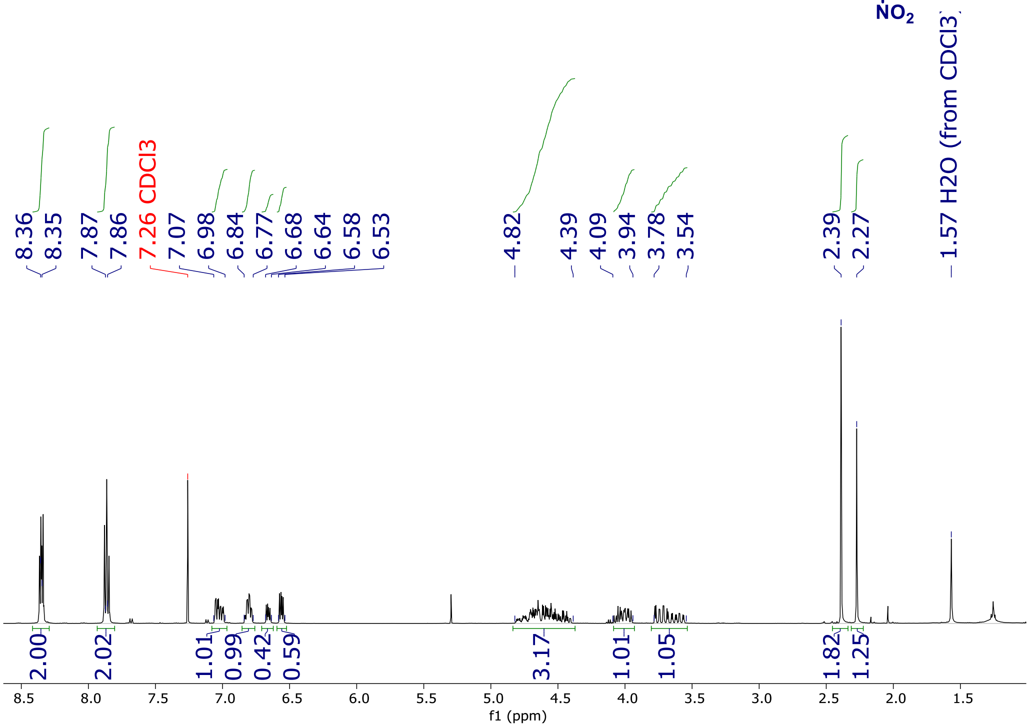

^{13}C NMR (126 MHz, CDCl$_3$):

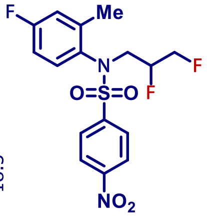

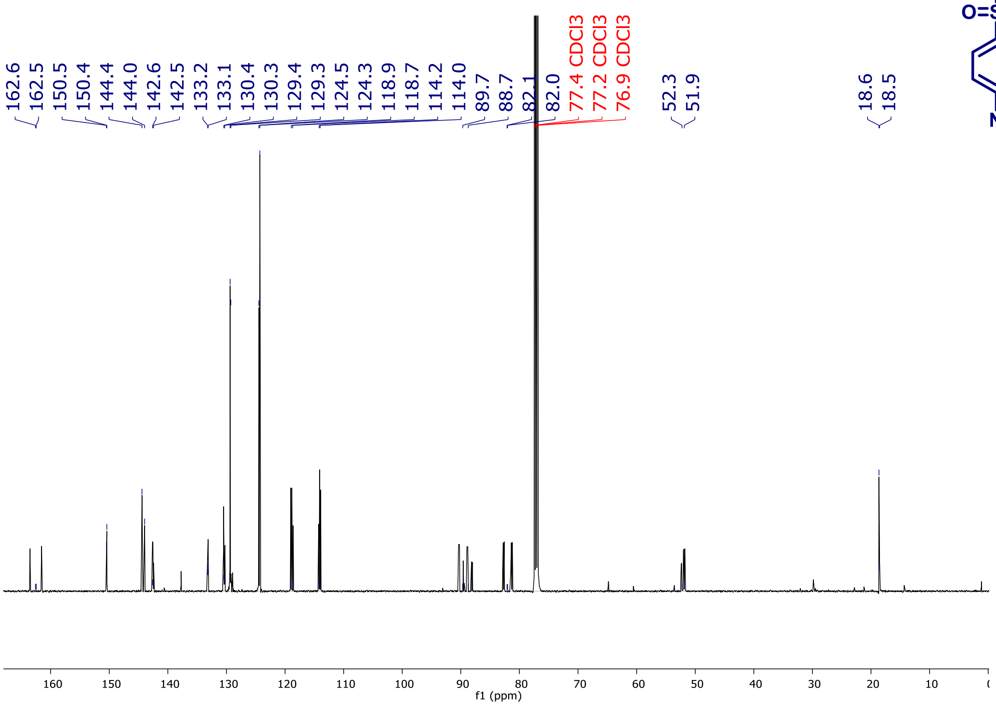

^{19}F NMR (376 MHz, CDCl$_3$):

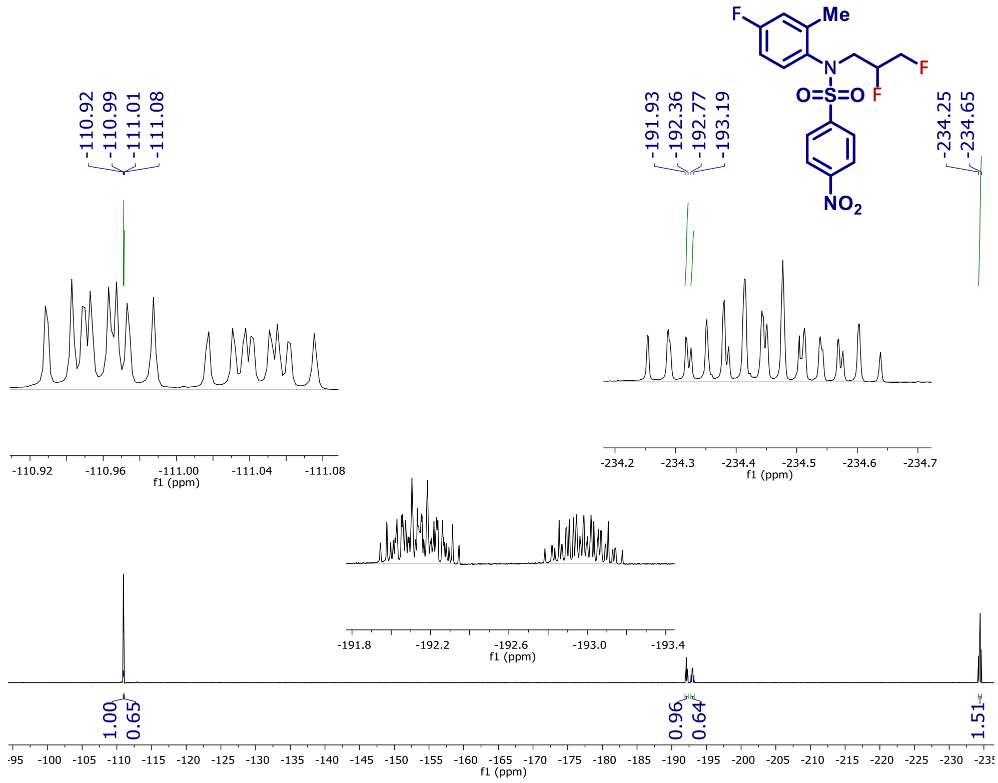

N-(2,3-difluoropropyl)-N-(4-iodophenyl)-4-nitrobenzenesulfonamide, 3ad

^1H NMR (500 MHz, $CDCl_3$):

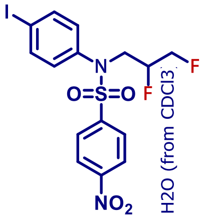

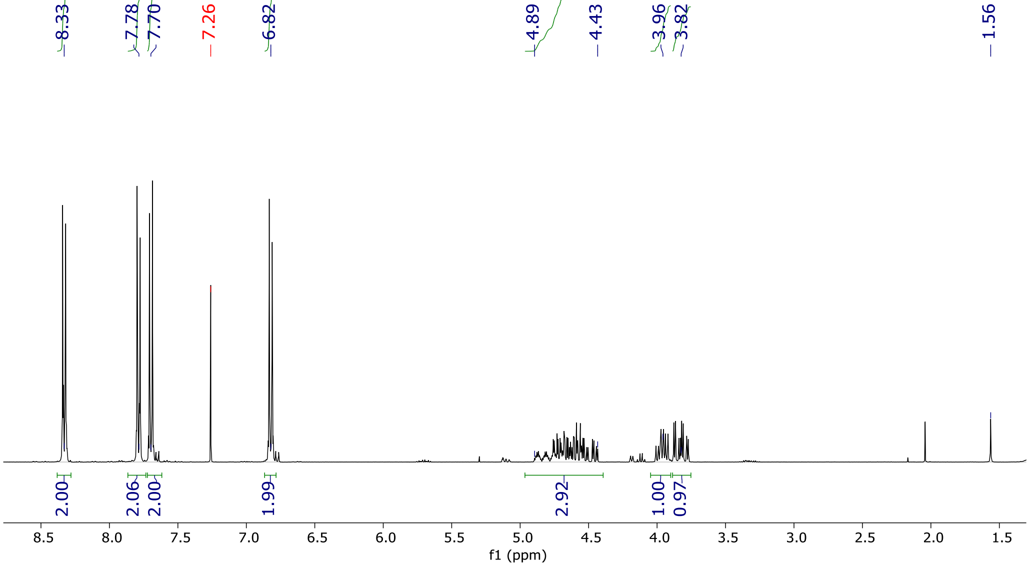

^{13}C NMR (126 MHz, $CDCl_3$):

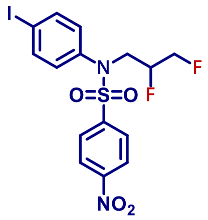

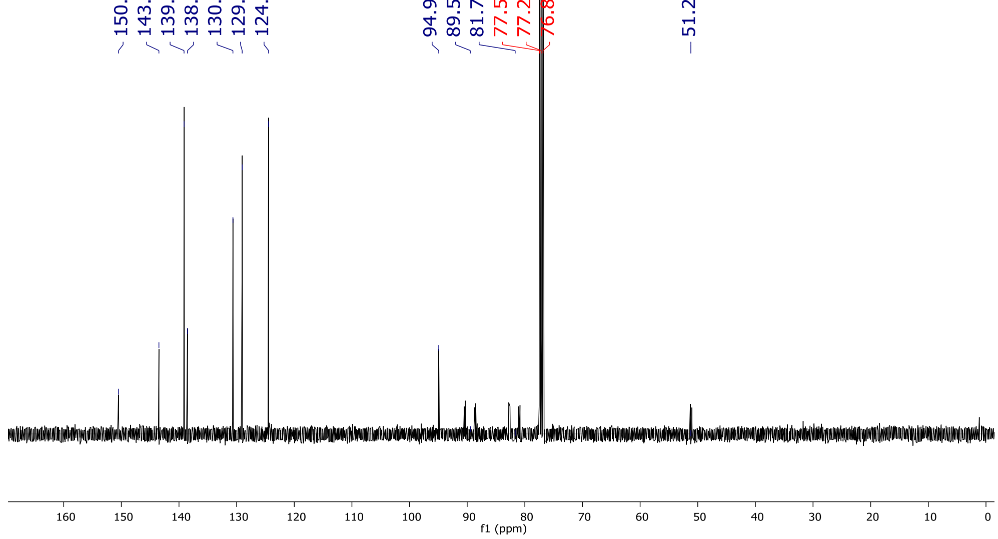

^{19}F NMR (376 MHz, CDCl3):

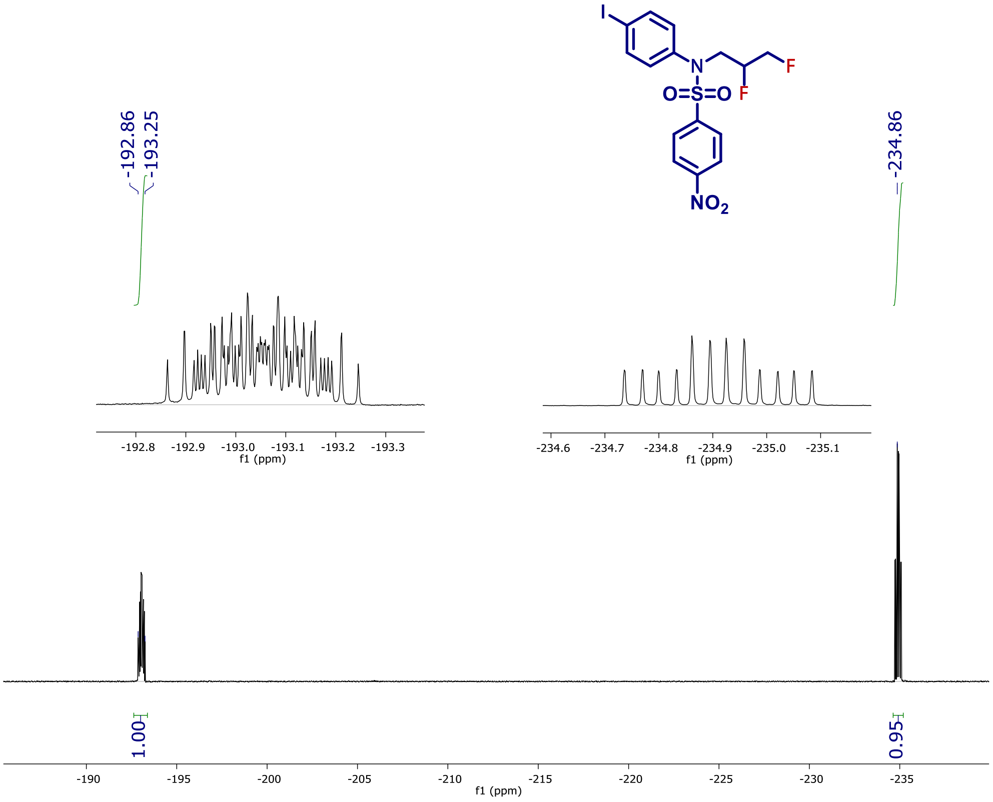

N-(2-bromophenyl)-N-(2,3-difluoropropyl)-4-nitrobenzenesulfonamide, 3ae

^{1}H NMR (500 MHz, $CDCl_3$):

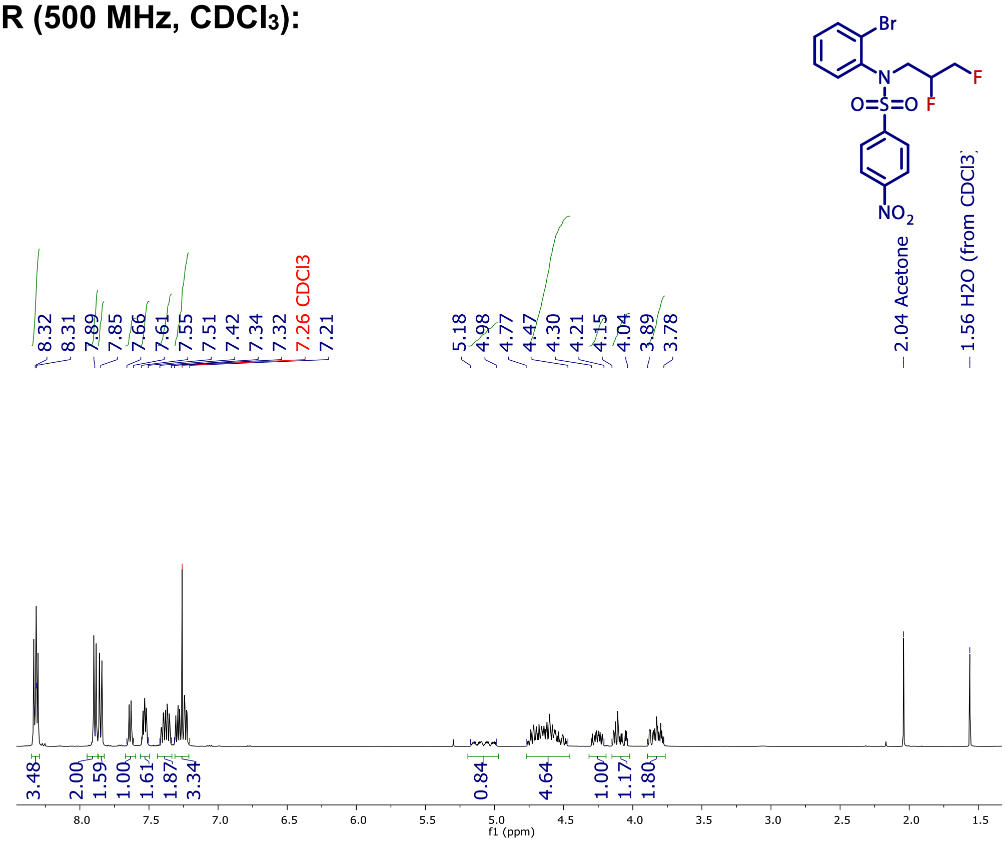

^{13}C NMR (126 MHz, $CDCl_3$):

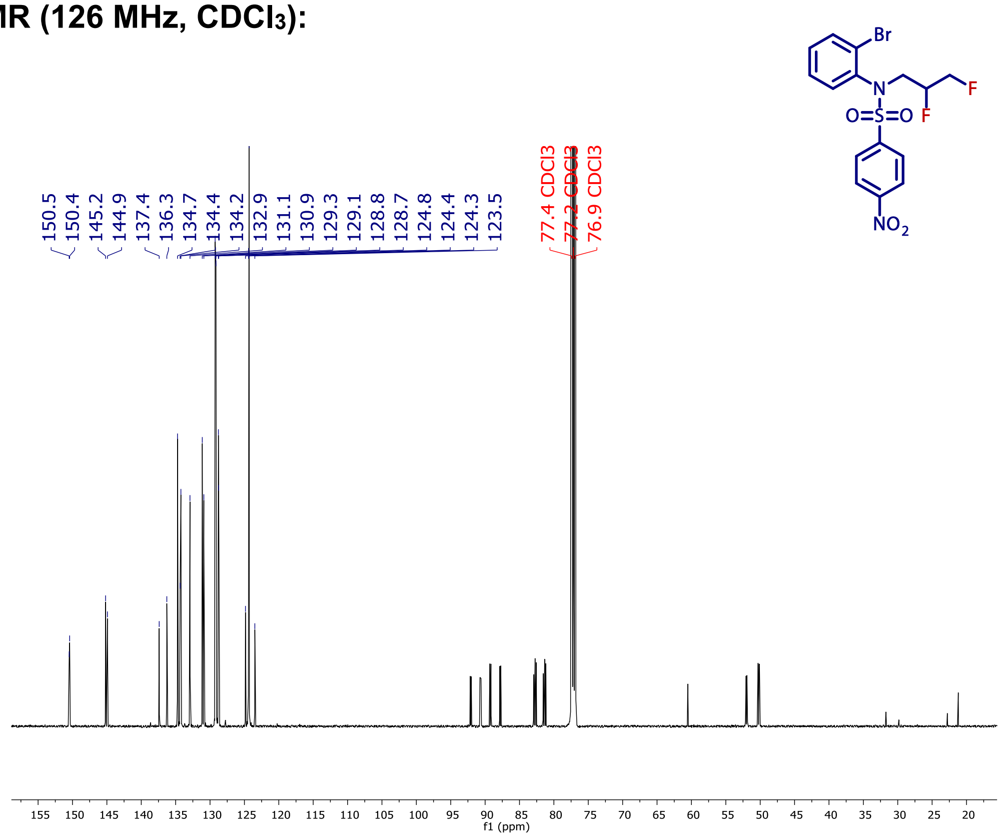

^{19}F NMR (376 MHz, CDCl₃):

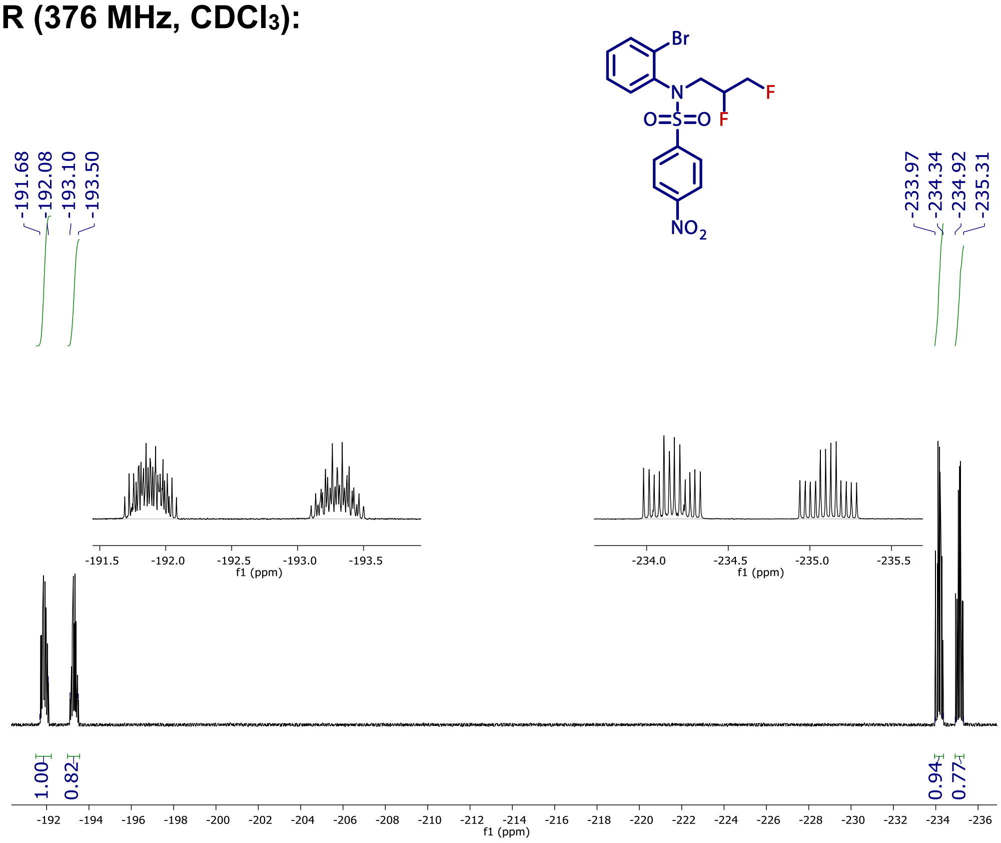

N-(2,3-difluoropropyl)-N-mesityl-4-nitrobenzenesulfonamide, 3af

^{1}H NMR (500 MHz, $CDCl_3$):

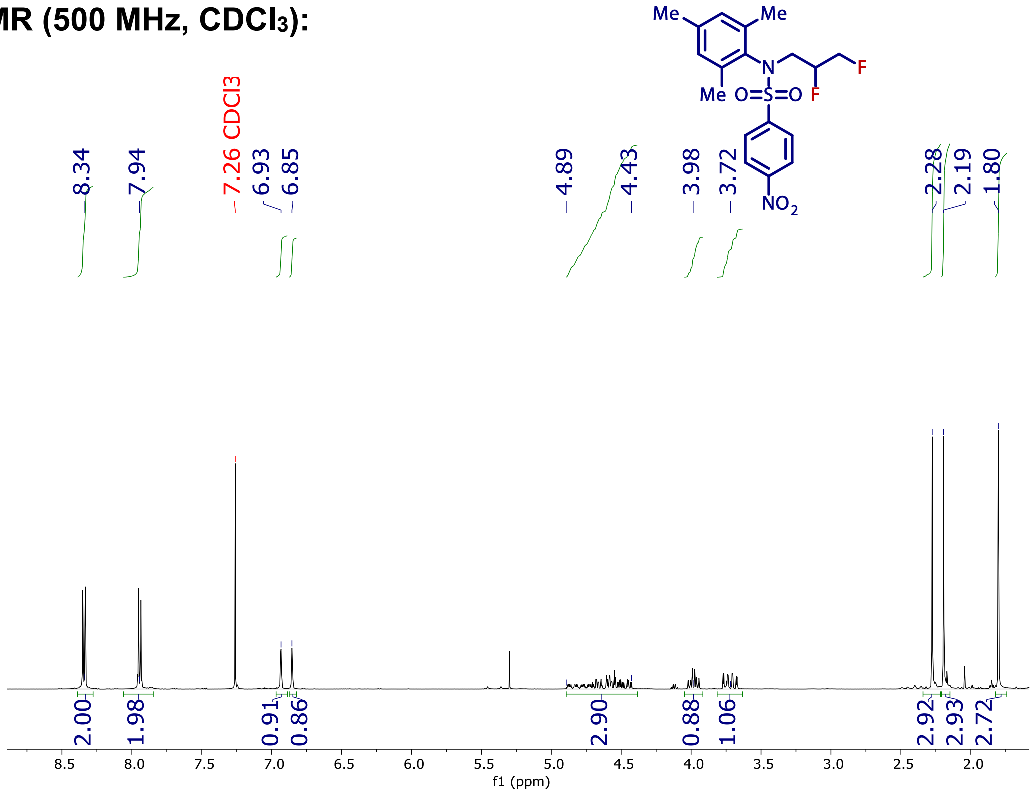

^{13}C NMR (126 MHz, $CDCl_3$):

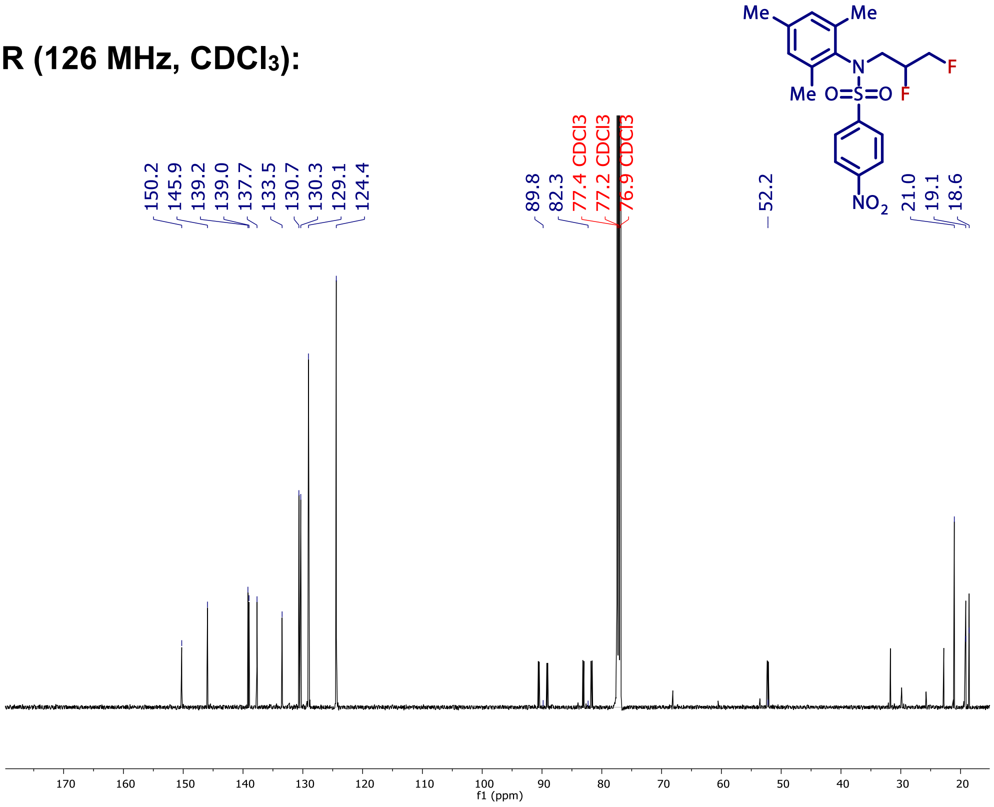

^{19}F NMR (376 MHz, CDCl$_3$):

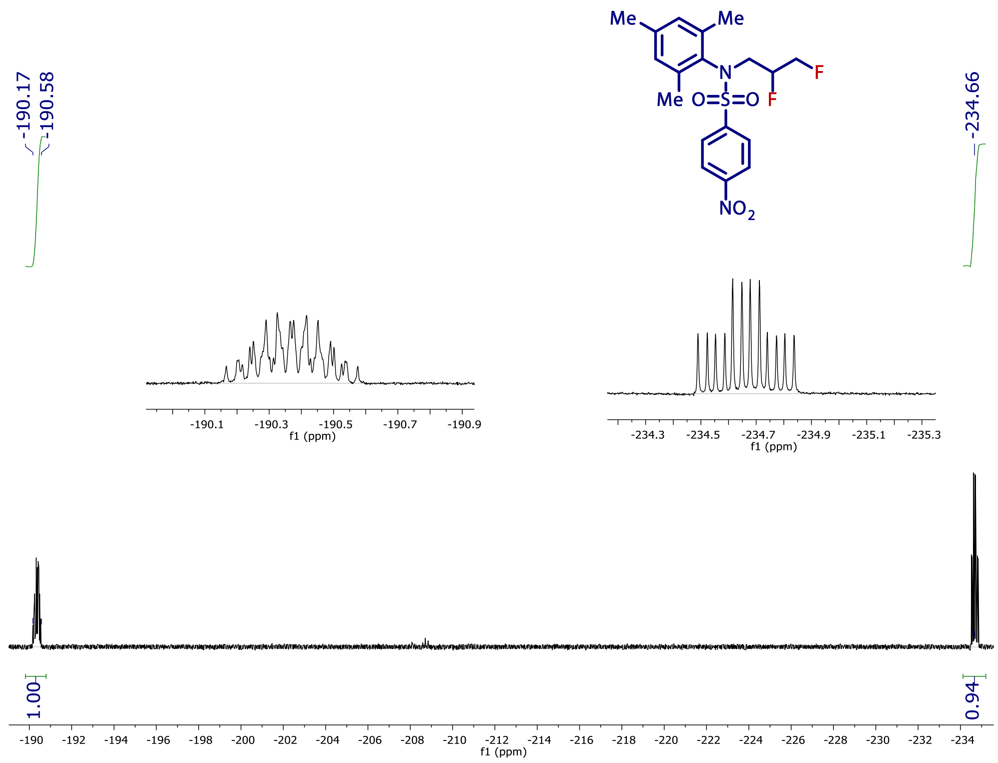

N-(2,3-difluoro-2-methylpropyl)-N-(4-fluorophenyl)-4-nitrobenzenesulfonamide, 3ag

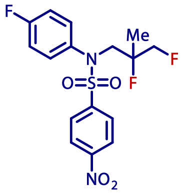

^{1}H NMR (500 MHz, CDCl$_3$):

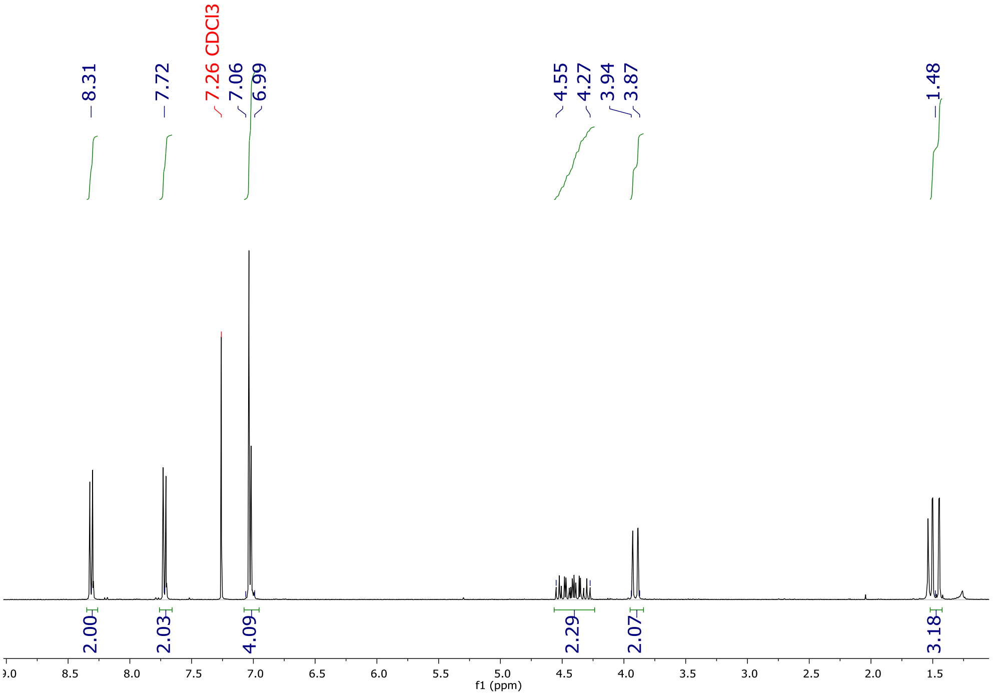

^{13}C NMR (126 MHz, CDCl$_3$):

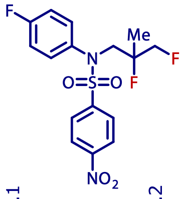

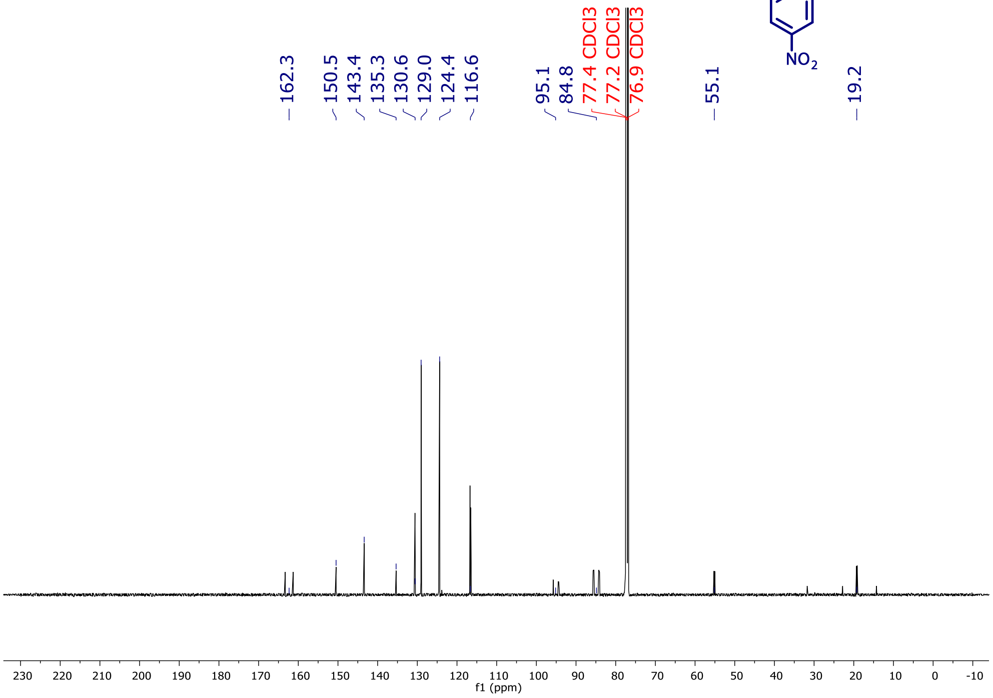

^{19}F NMR (376 MHz, CDCl$_3$):

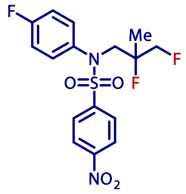

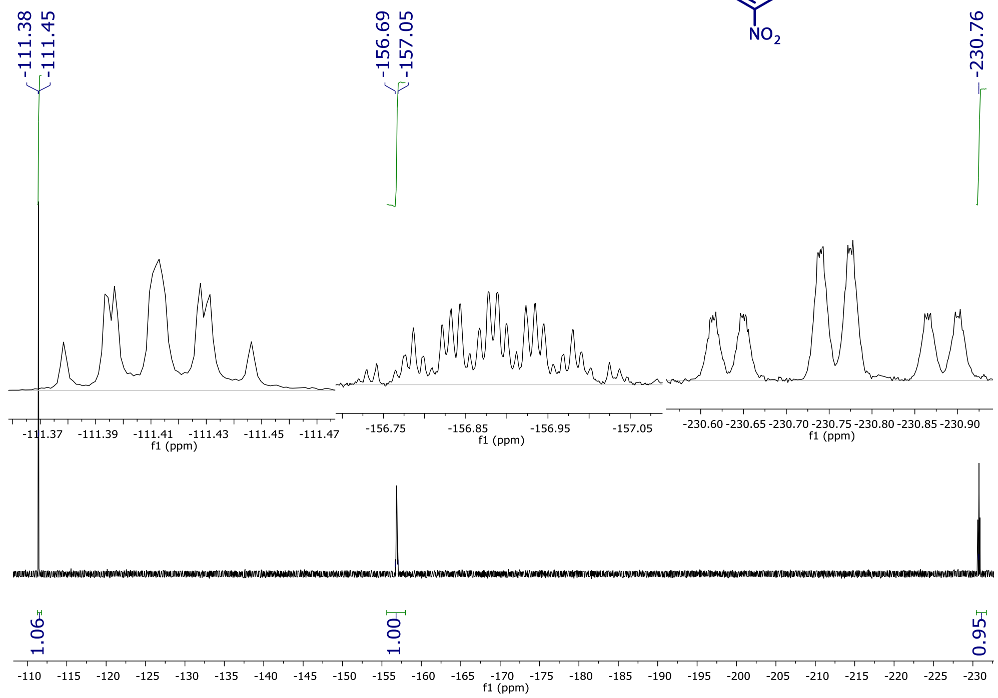

1-(1,2-difluoro-2-methylpropyl)-4-fluorobenzene, 3ak

^{1}H NMR (500 MHz, $CDCl_3$):

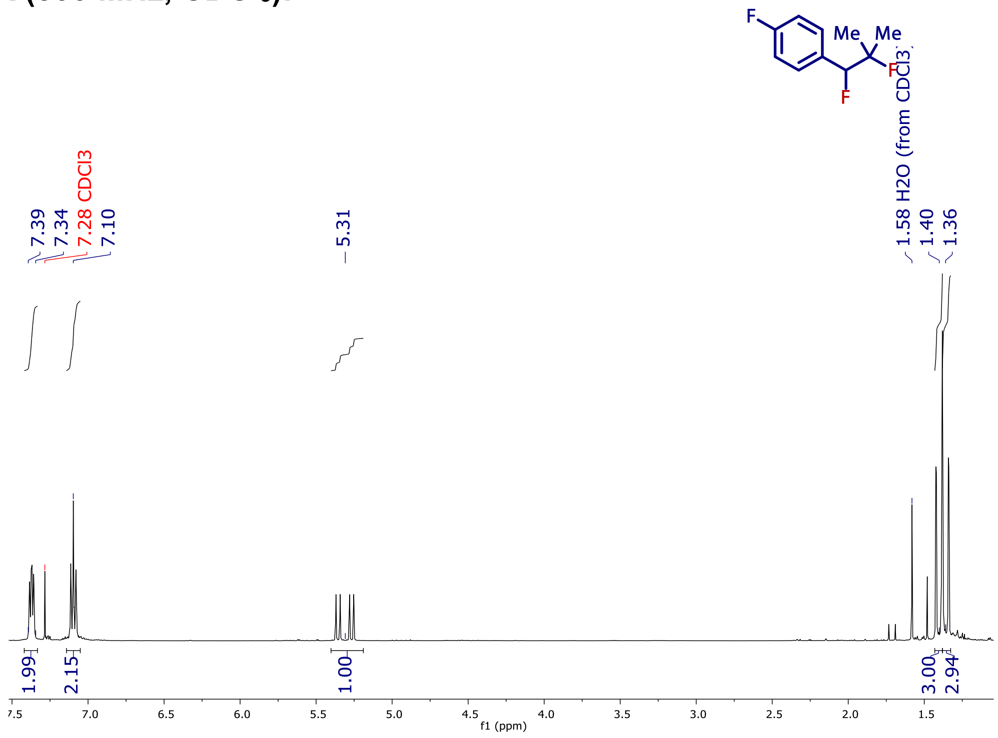

^{13}C NMR (126 MHz, $CDCl_3$):

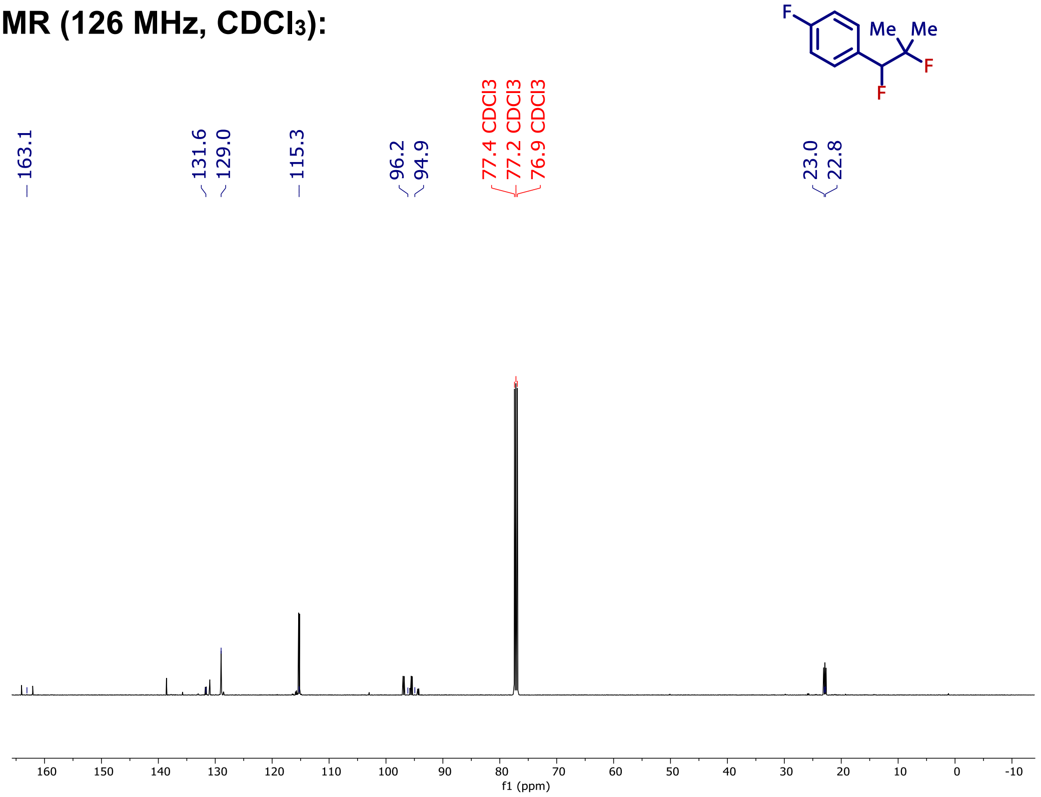

^{19}F NMR (376 MHz, CDCl$_3$):

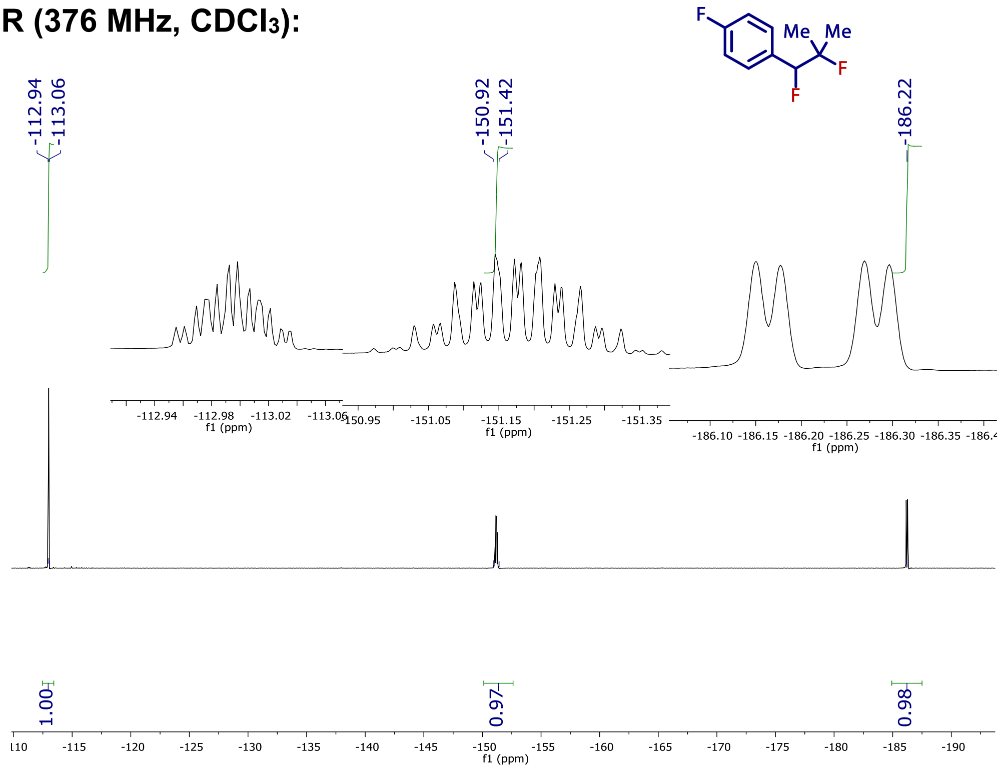

1-(1,2-difluoro-2-methylpropyl)-3,5-bis(trifluoromethyl)benzene, 3al

^{1}H NMR (500 MHz, CDCl3):

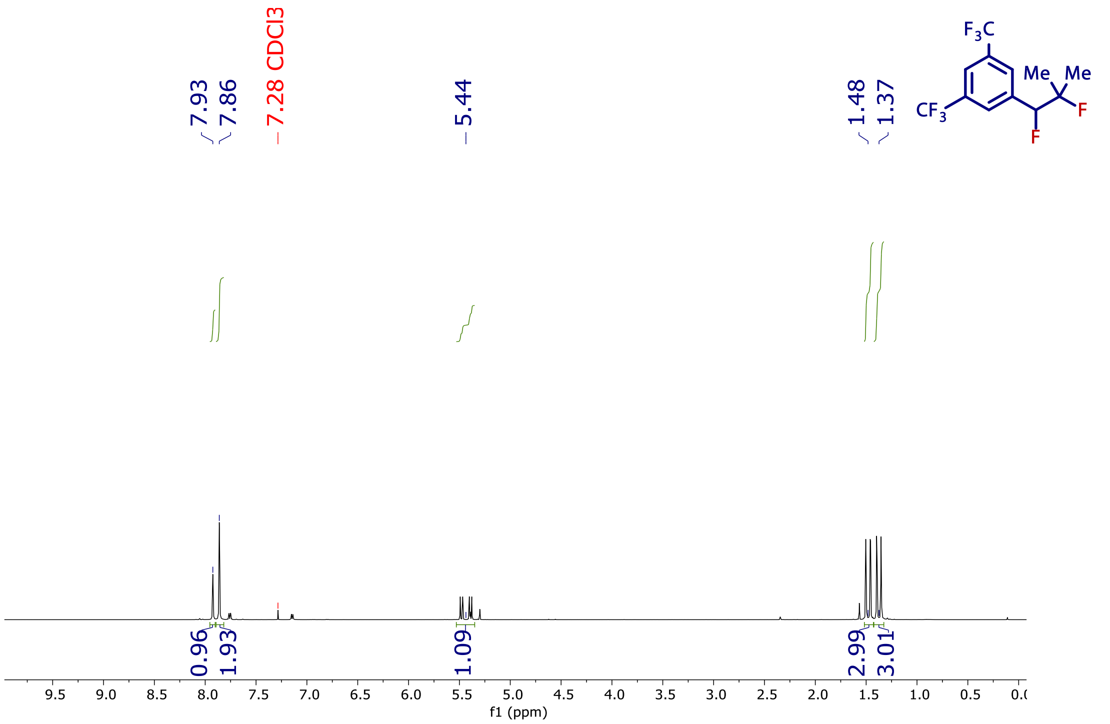

^{13}C NMR (126 MHz, CDCl3):

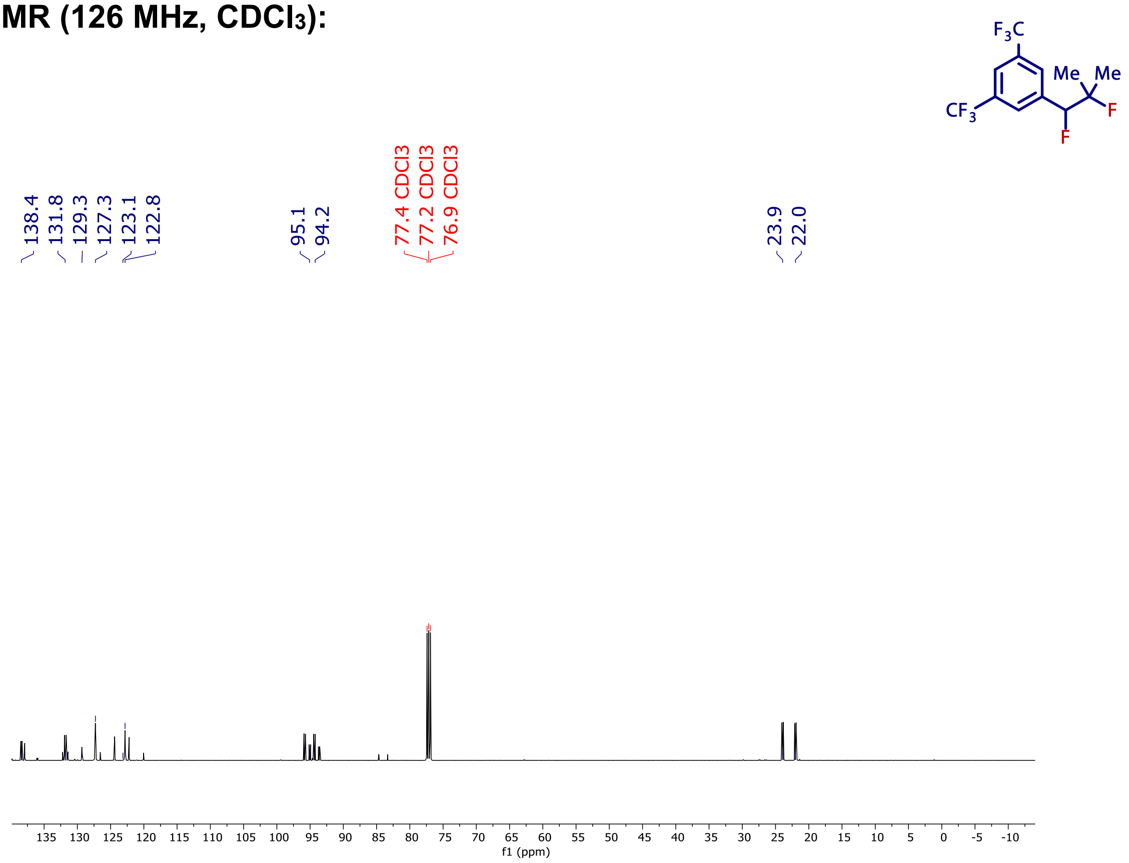

^{19}F NMR (376 MHz, CDCl₃):

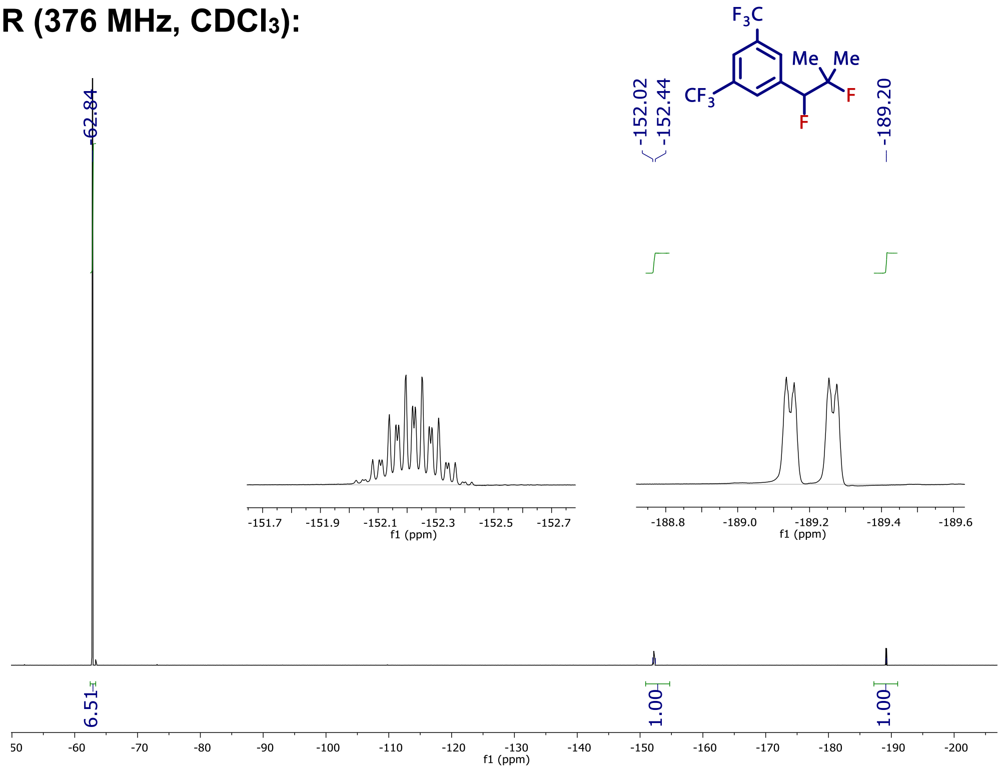

N-(2,3-difluoropropyl)-4-methyl-N-(4-methylbenzyl)benzenesulfonamide, 3am

^{1}H NMR (500 MHz, CDCl$_3$) (Containing 5% impurity)**:**

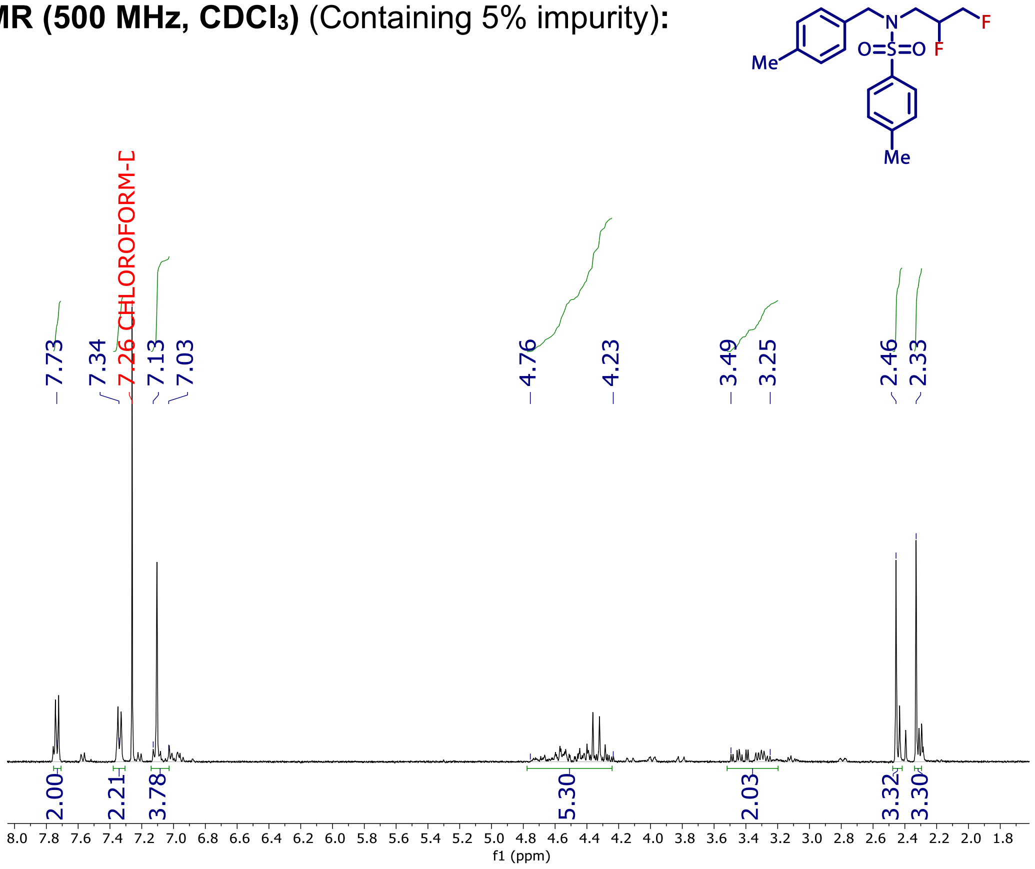

^{13}C NMR (126 MHz, CDCl$_3$):

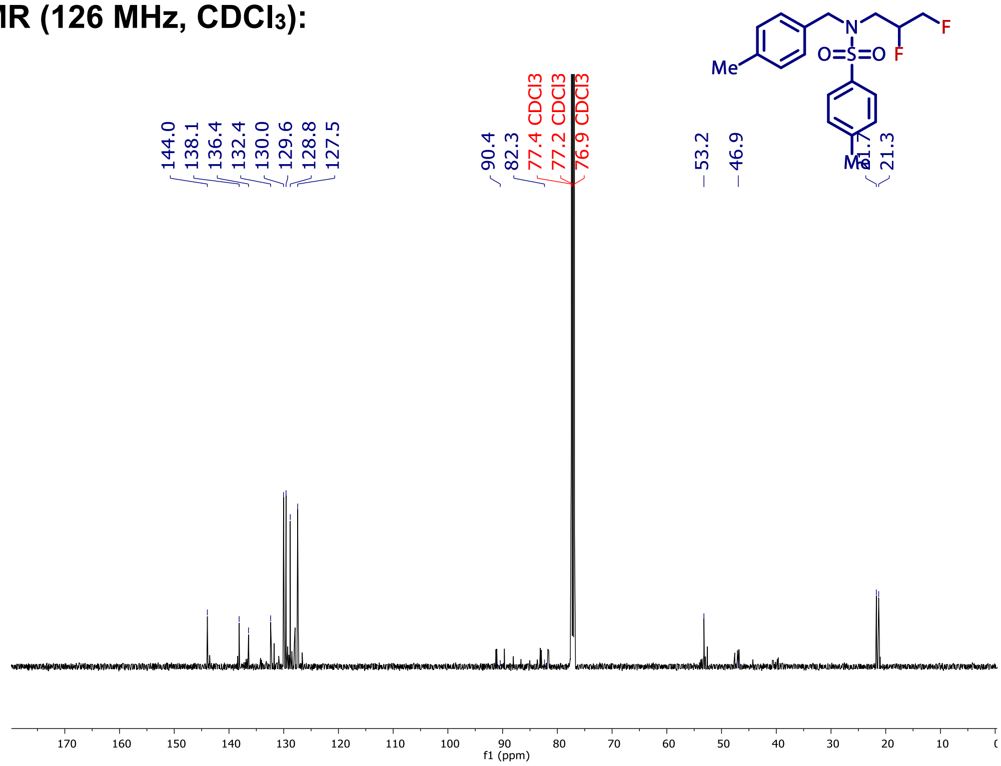

^{19}F NMR (376 MHz, CDCl$_3$):

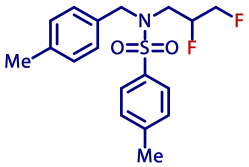

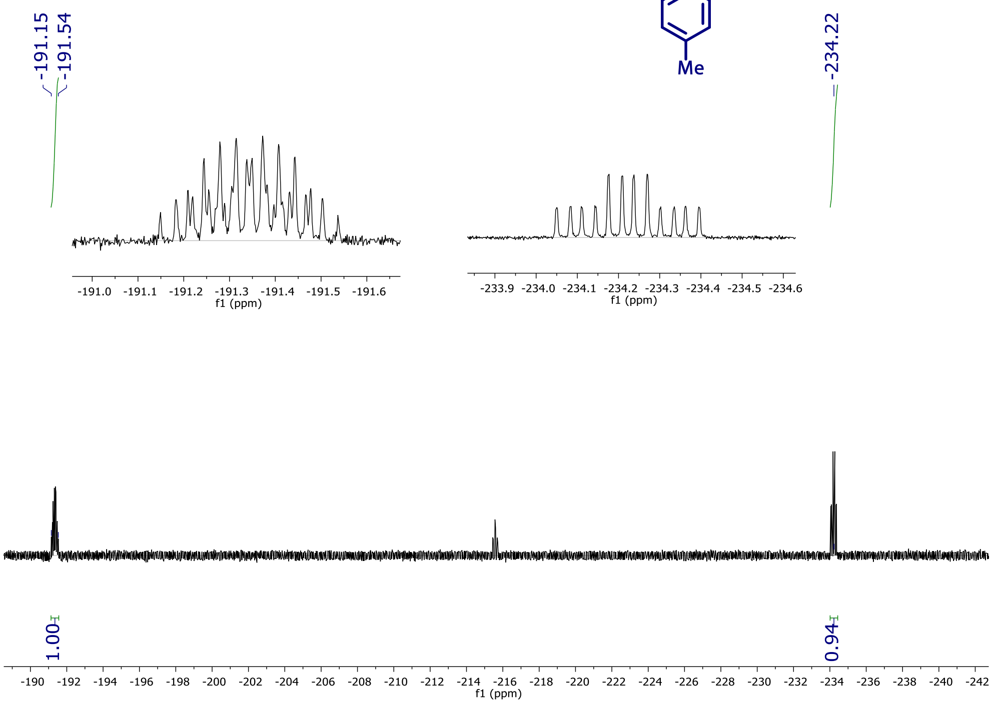

N-(4-chlorobenzyl)-N-(2,3-difluoro-2-methylpropyl)-4-methylbenzenesulfonamide, 3an

^{1}H NMR (500 MHz, $CDCl_3$):

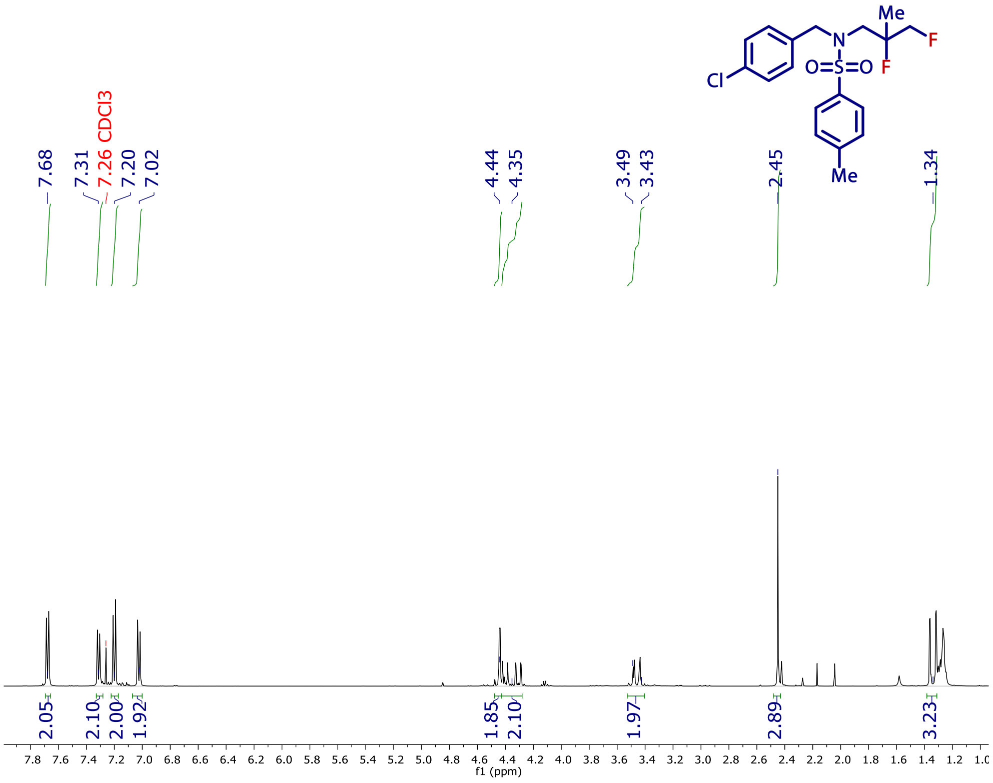

^{13}C NMR (126 MHz, $CDCl_3$):

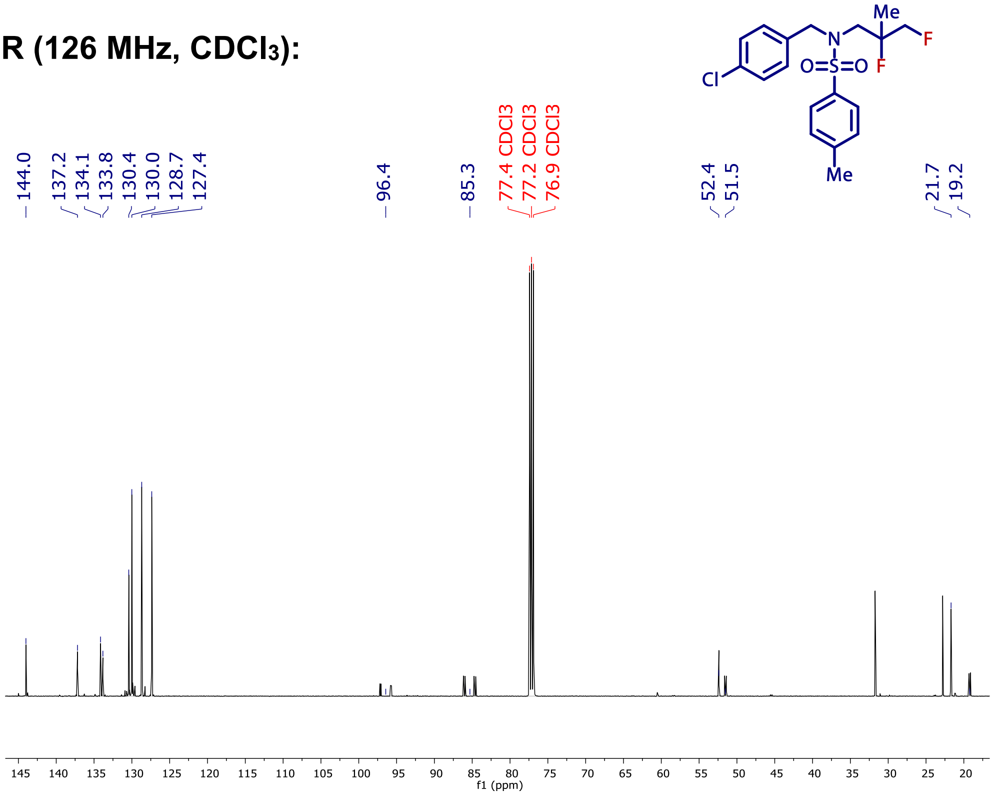

^{19}F NMR (376 MHz, CDCl$_3$):

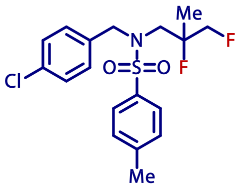

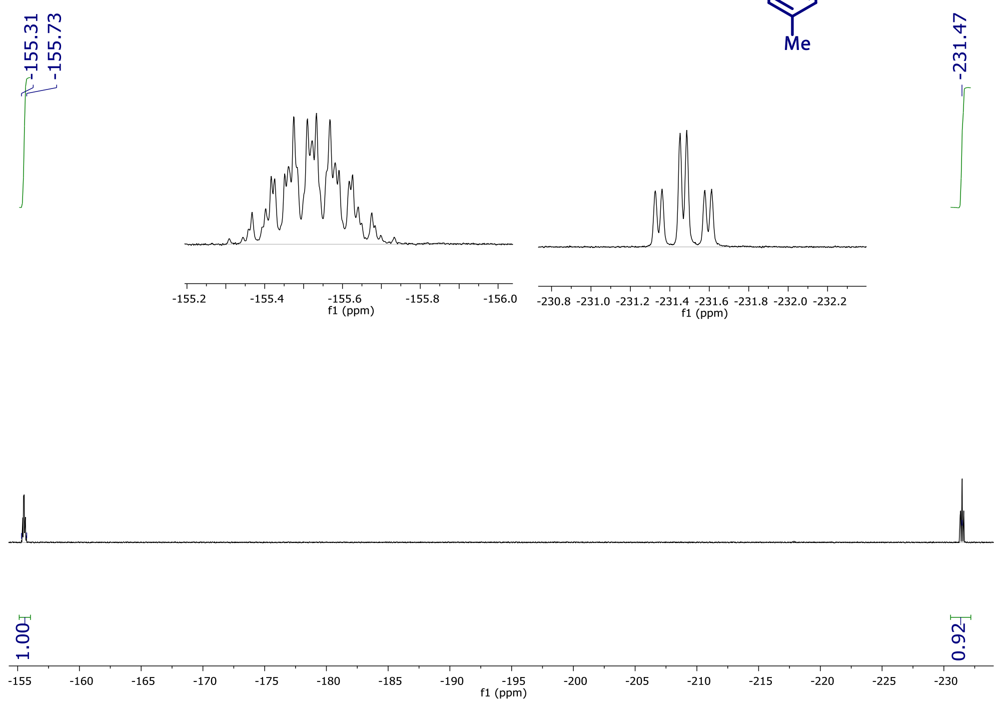

N-(4-chlorobenzyl),N-2,3-difluorobutyl)-4-methylbenzenesulfonamide, 3ao

^{1}H NMR (500 MHz, $CDCl_3$):

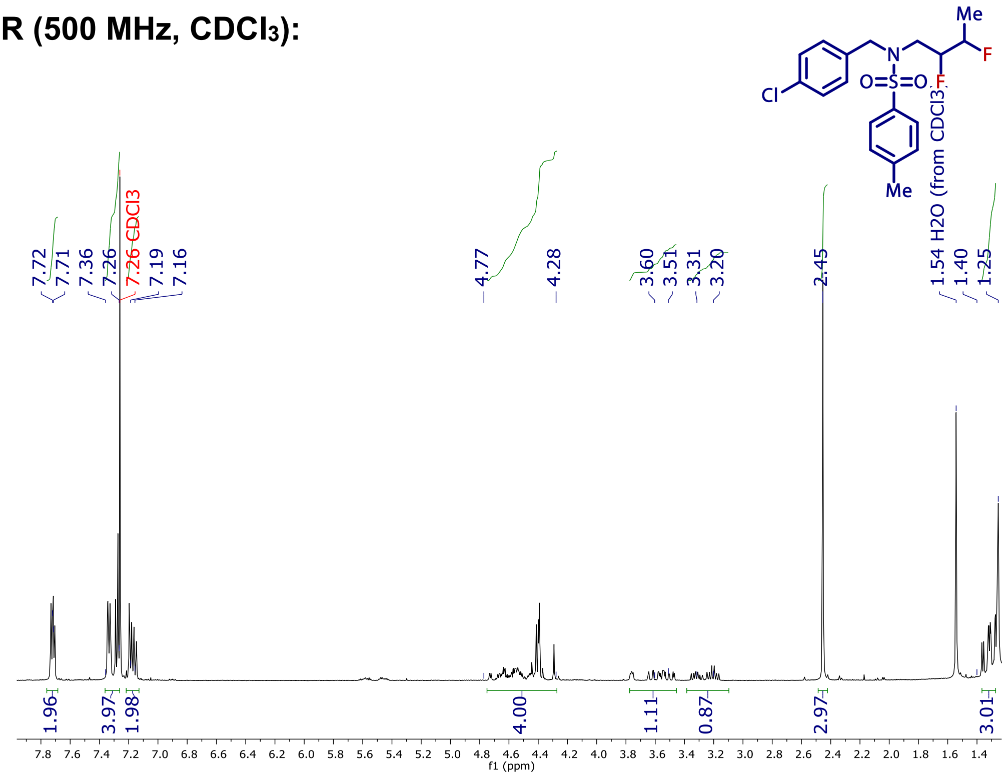

^{13}C NMR (126 MHz, $CDCl_3$):

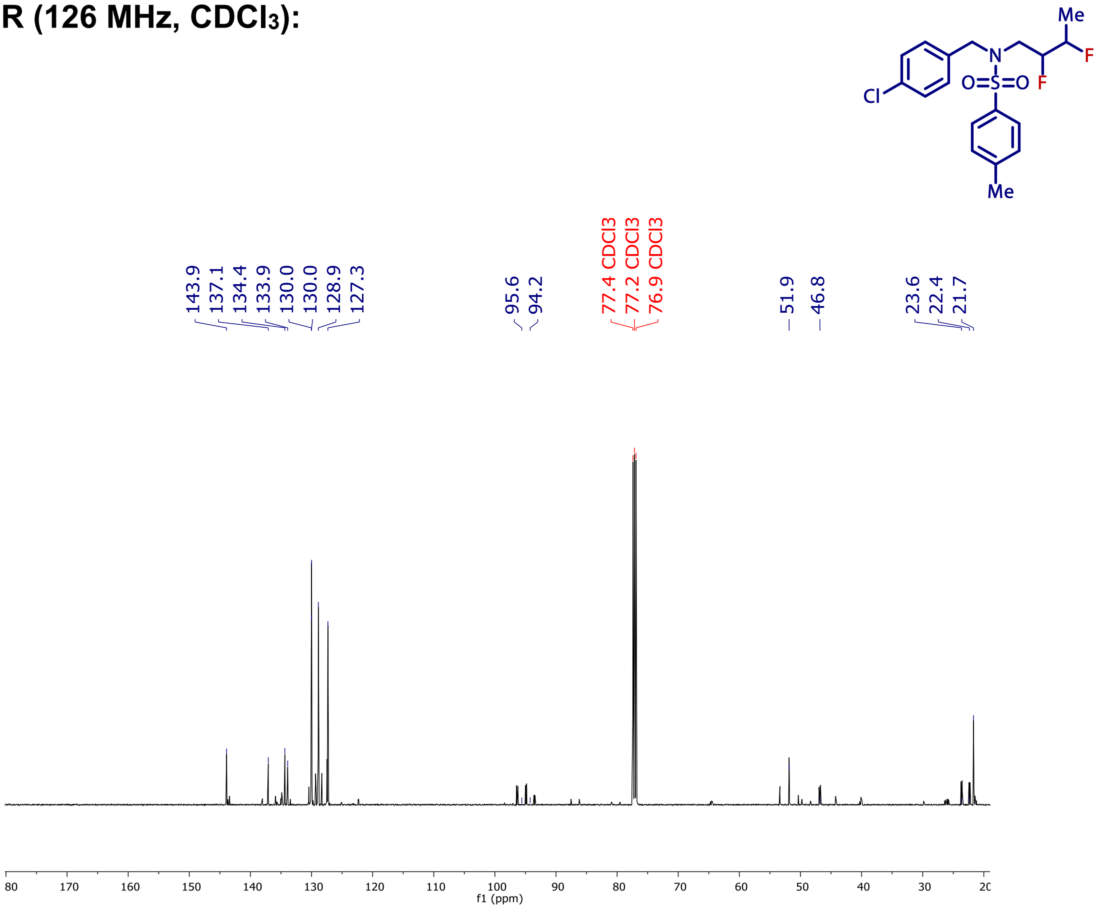

^{19}F NMR (376 MHz, $CDCl_3$):

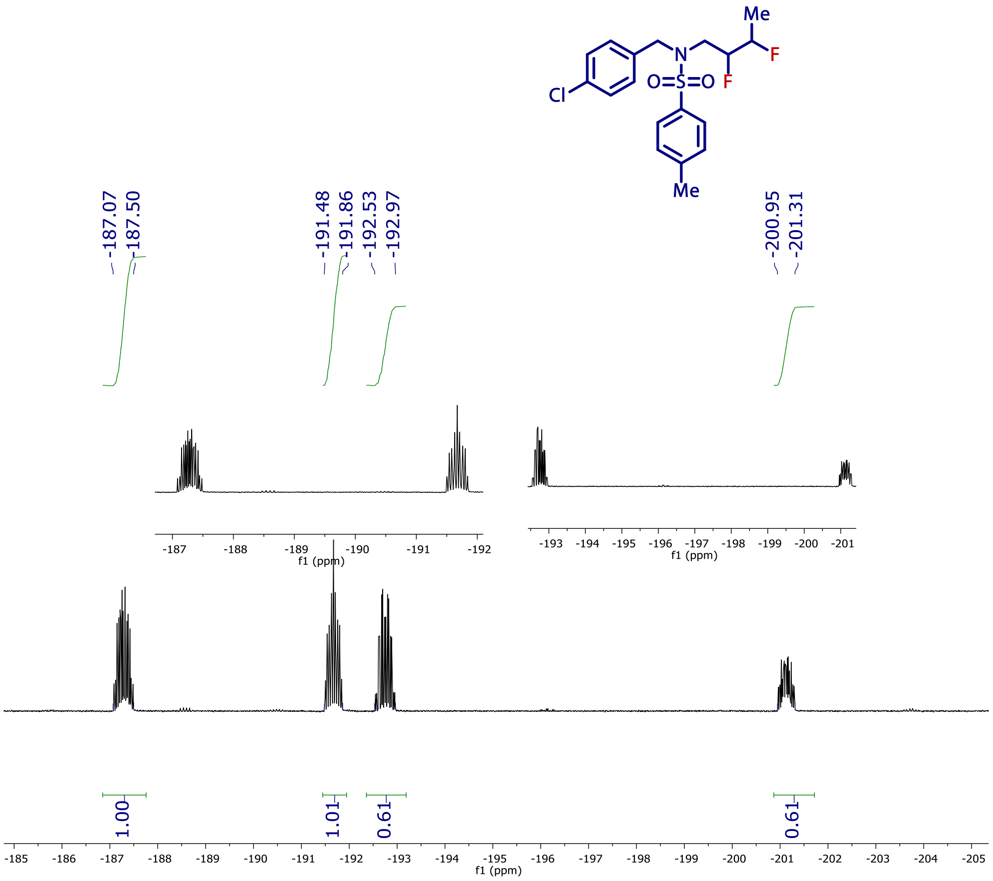

N-(4-chlorobenzyl)-N-(2,3-difluoro-3-methylbutyl)-4-methylbenzenesulfonamide, 3ap

^{1}H NMR (500 MHz, $CDCl_3$):

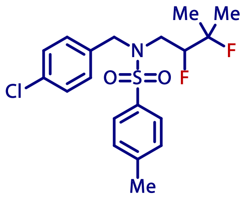

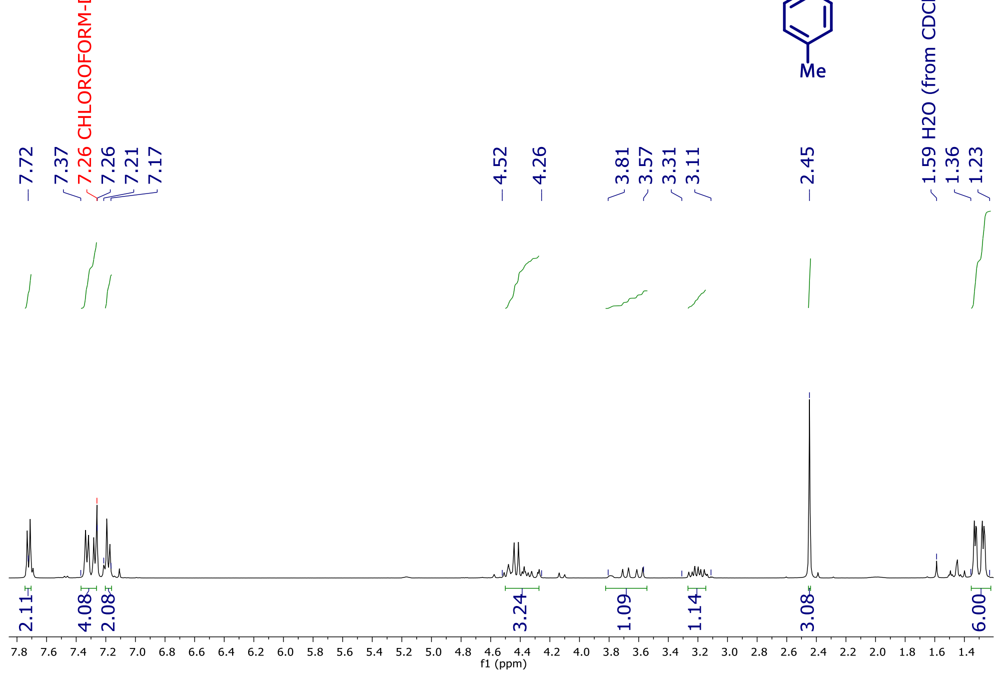

^{13}C NMR (126 MHz, $CDCl_3$):

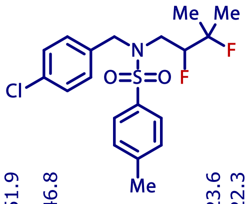

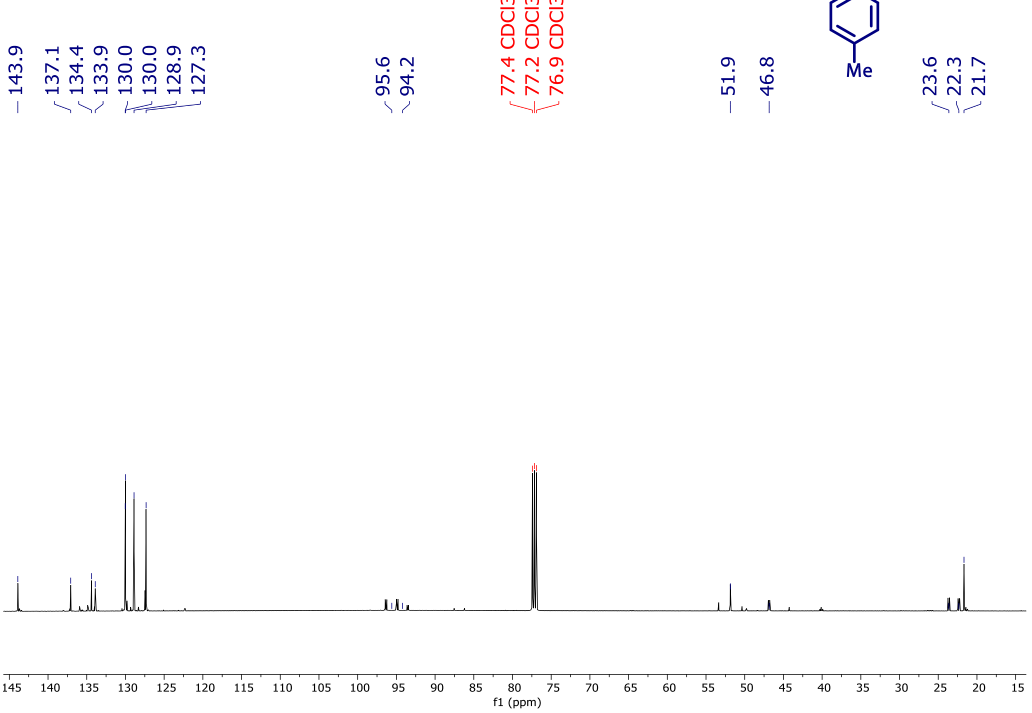

^{19}F NMR (376 MHz, CDCl$_3$):

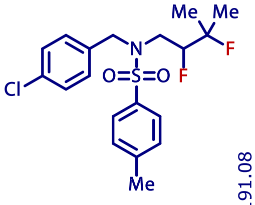

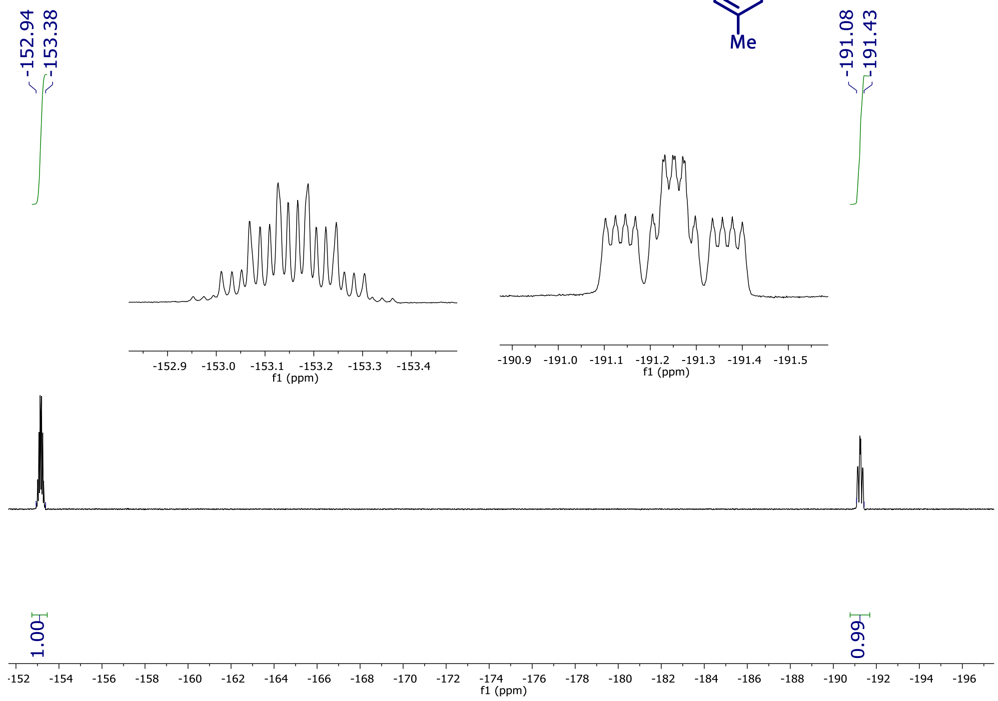

(±)-N,N-dibenzyl-3,4-difluorohexan-1-amine, 3aq

^{1}H NMR (500 MHz, CDCl$_3$):

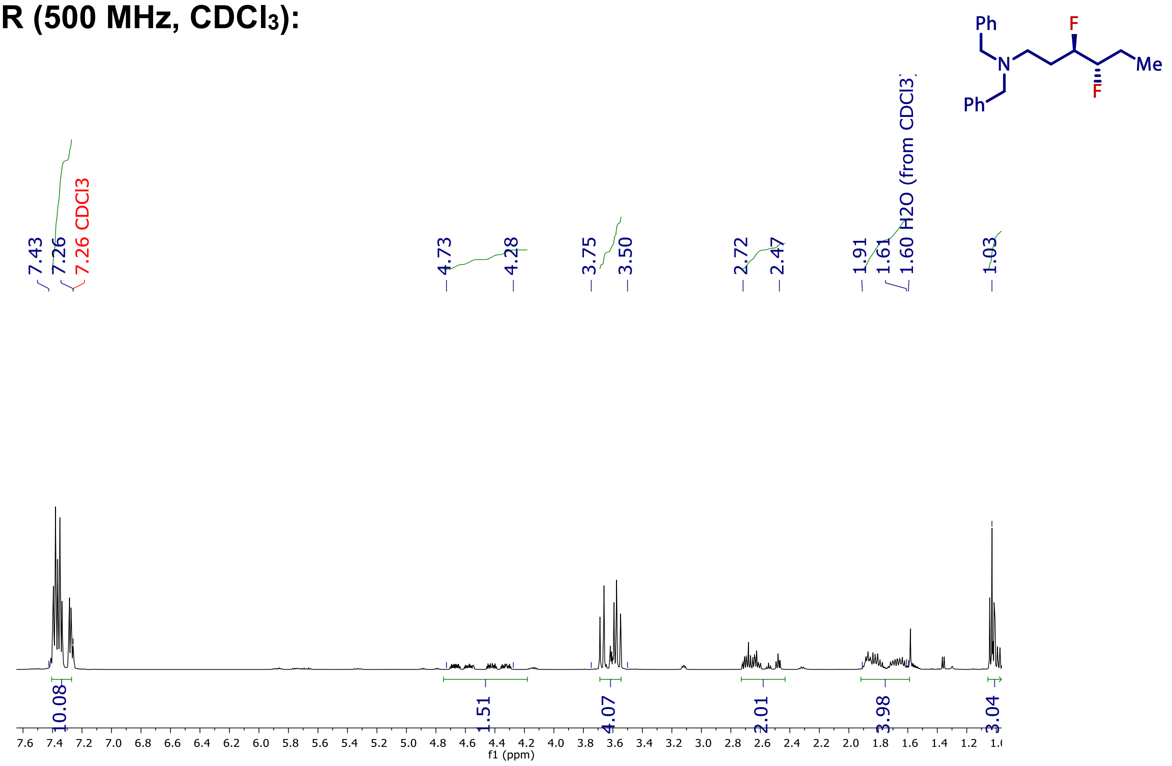

^{13}C NMR (126 MHz, CDCl$_3$):

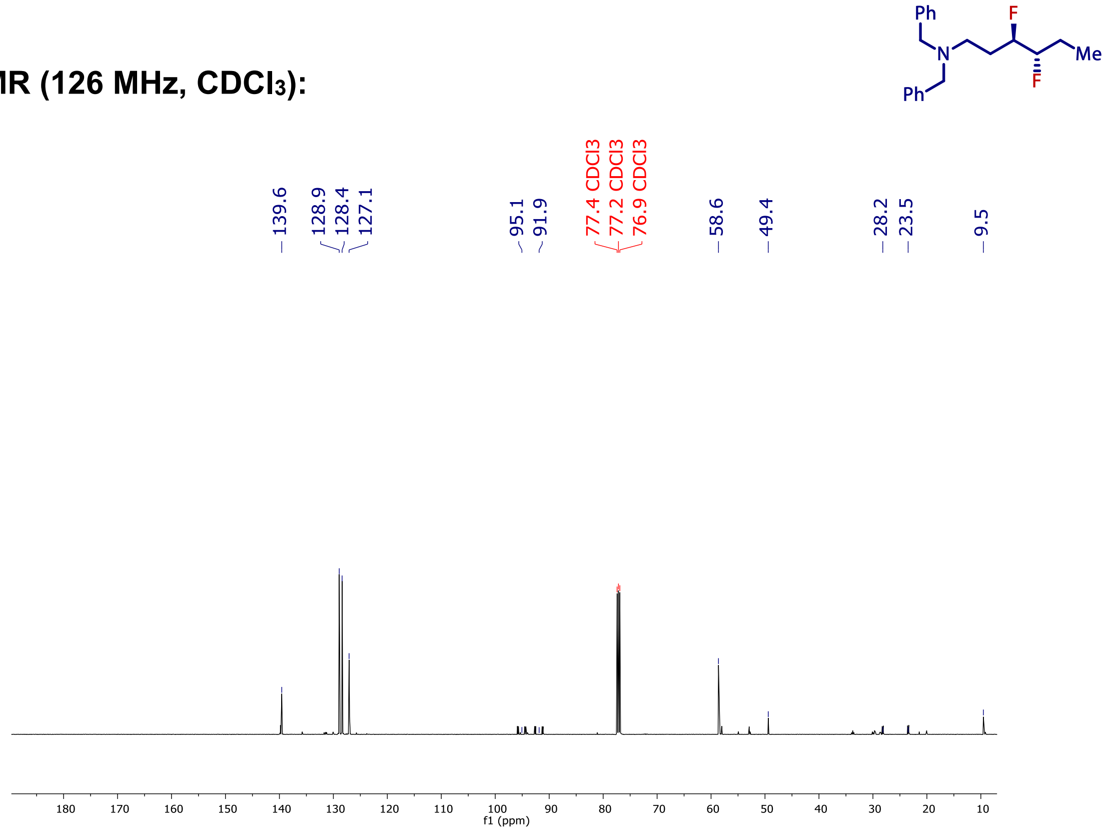

^{19}F NMR (376 MHz, $CDCl_3$):

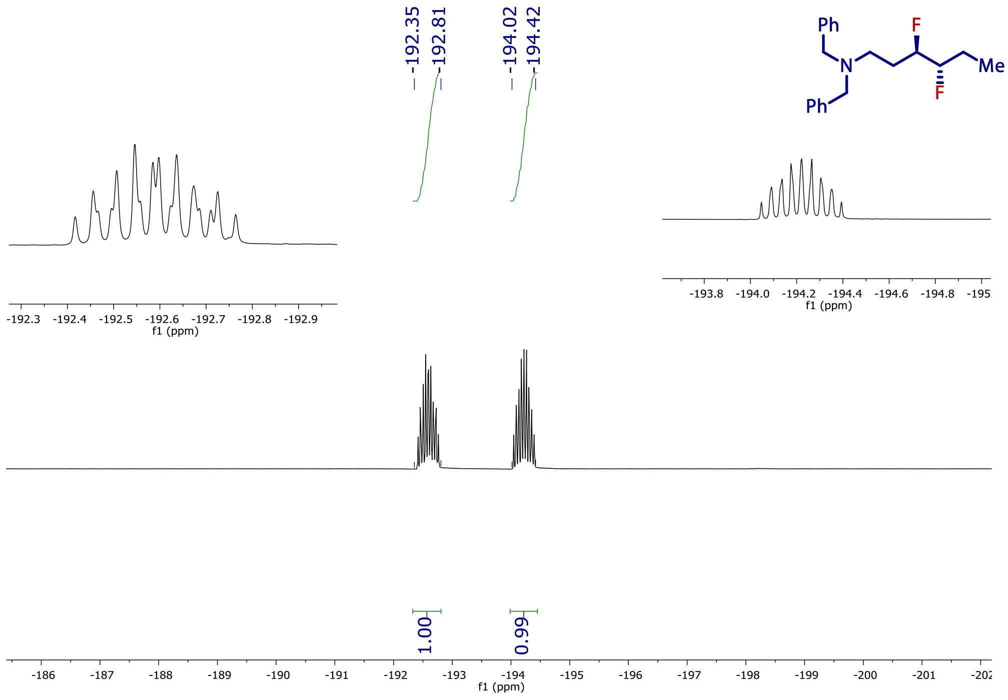

(±)-N,N-dibenzyl-3,4-difluorohexan-1-amine, 3ar

^{1}H NMR (500 MHz, CDCl$_3$):

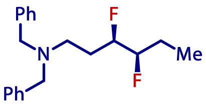

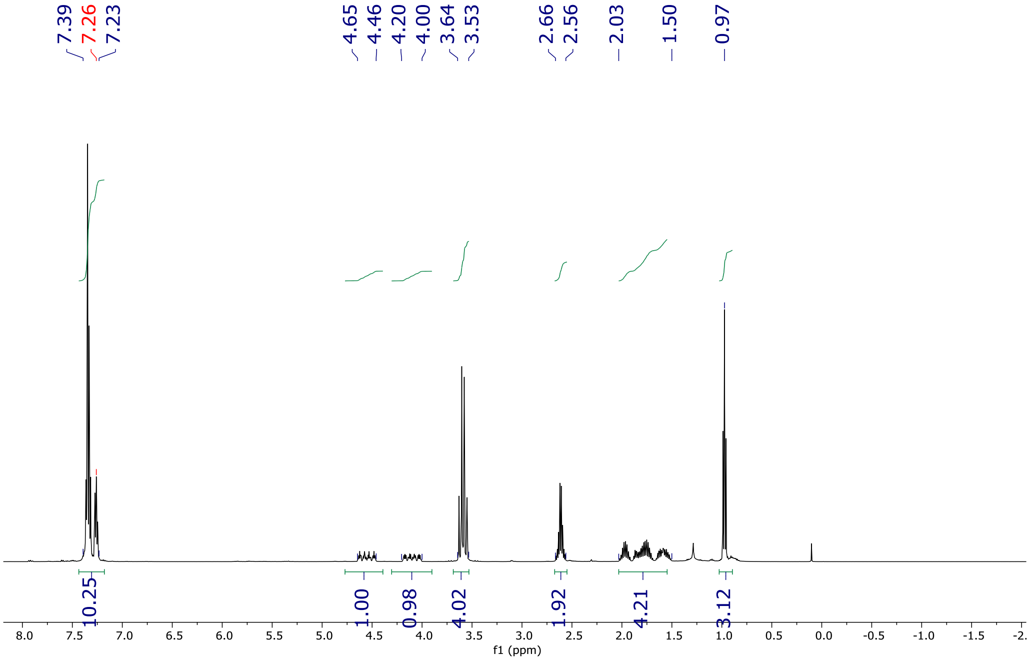

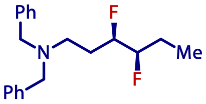

^{13}C NMR (126 MHz, CDCl$_3$):

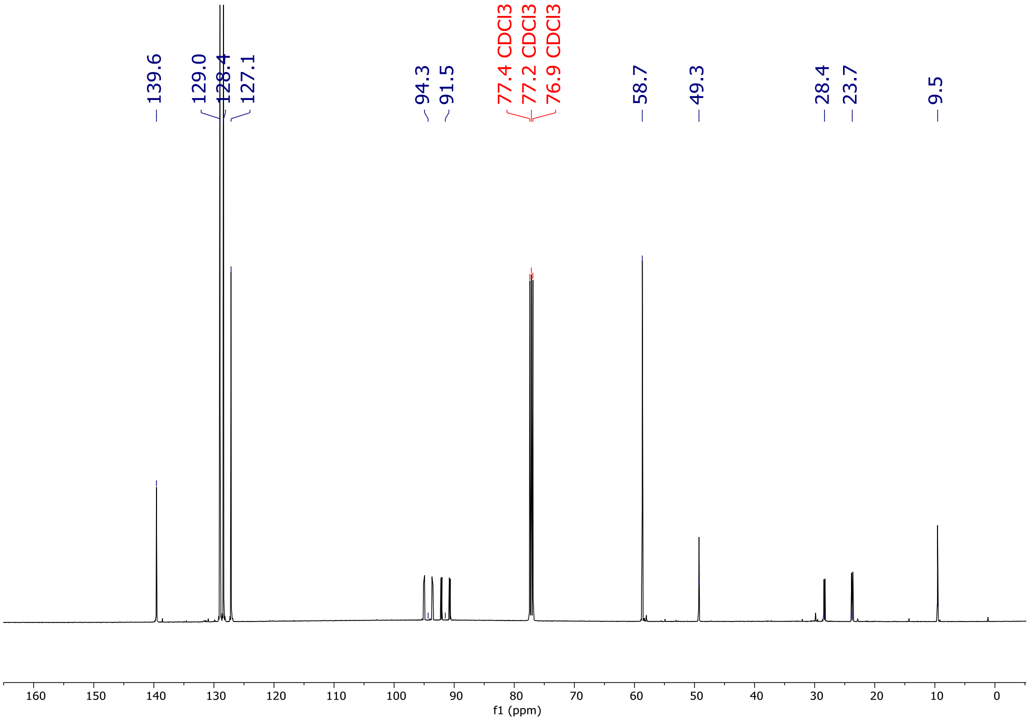

^{19}F NMR (376 MHz, $CDCl_3$):

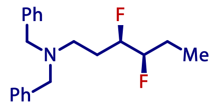

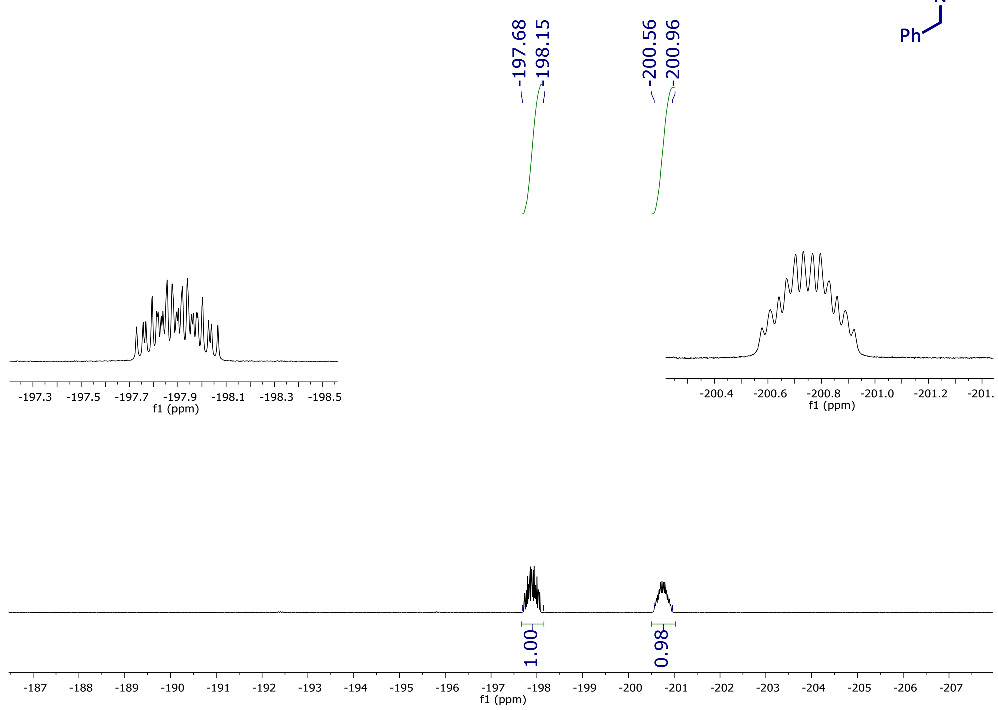

1-((2,3-difluoropropoxy)methyl)-2-methylbenzene, 3as

^{1}H NMR (500 MHz, $CDCl_3$):

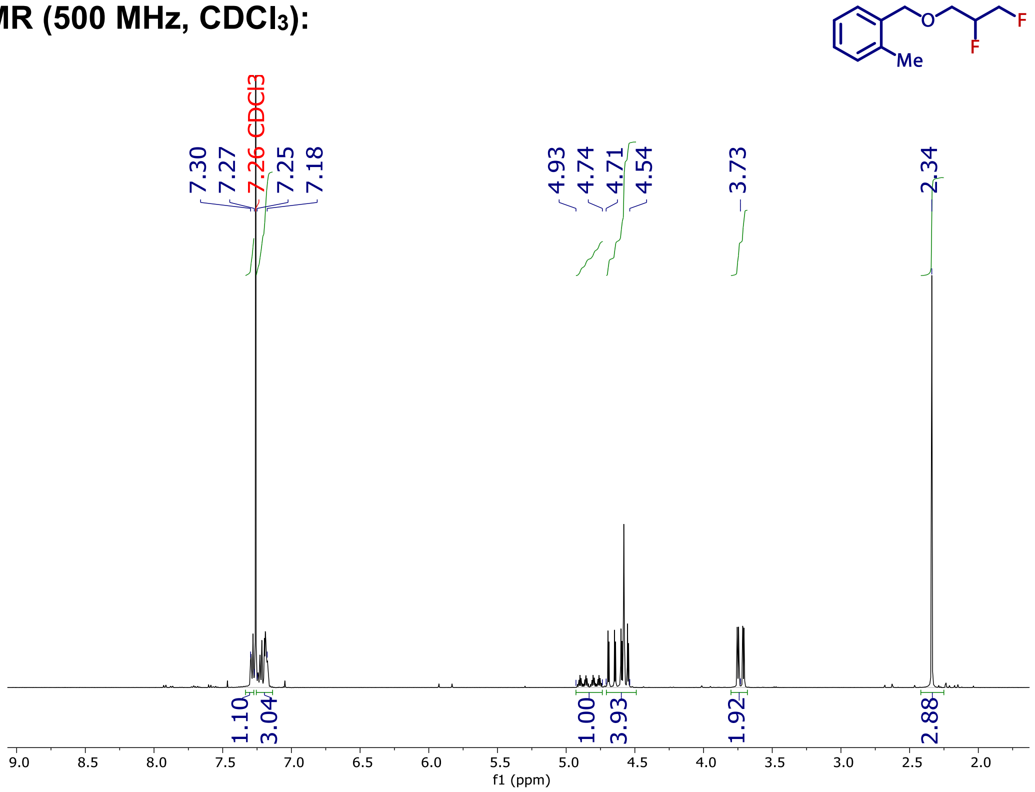

^{13}C NMR (126 MHz, $CDCl_3$):

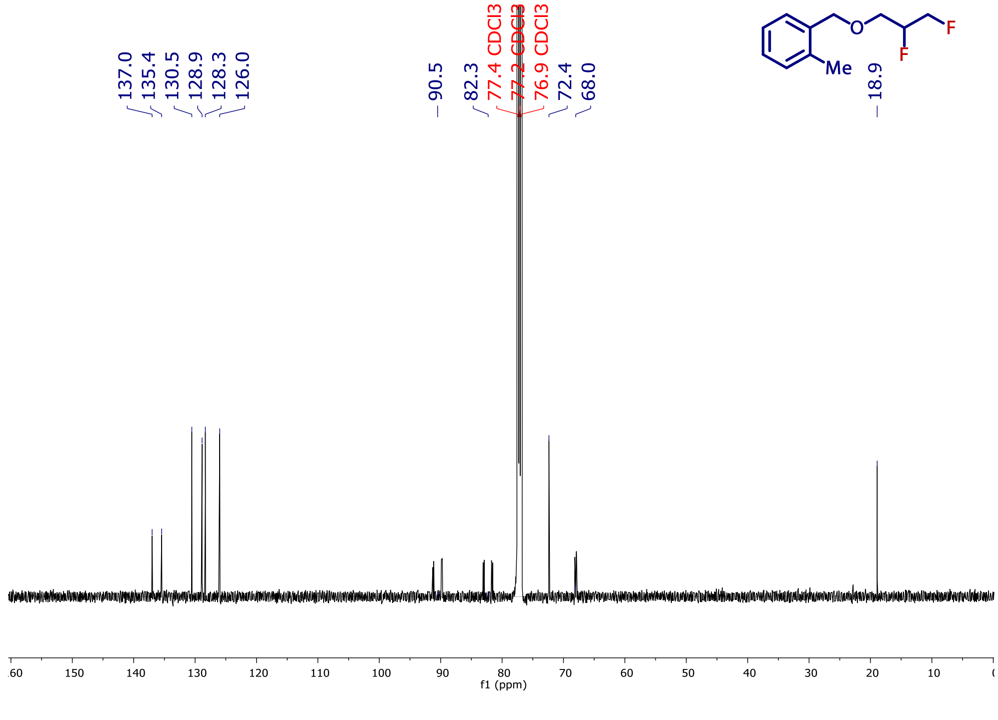

^{19}F NMR (376 MHz, $CDCl_3$):

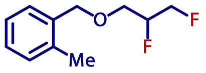

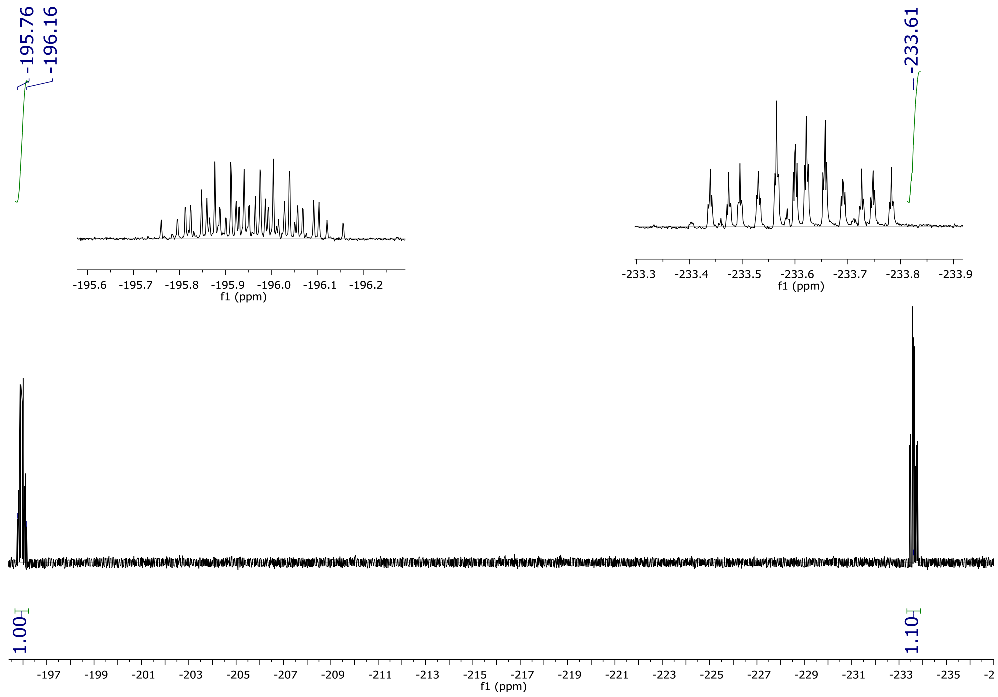

((2,3-difluoro-2-methylpropoxy)methyl)benzene, 3at

^{1}H NMR (500 MHz, $CDCl_3$):

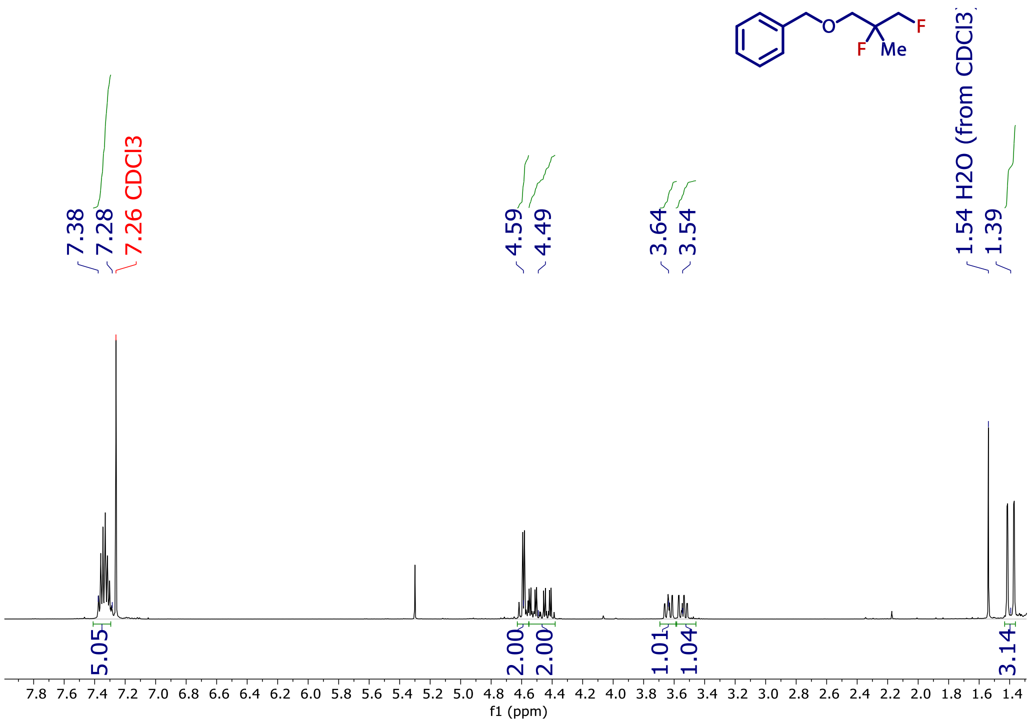

^{13}C NMR (126 MHz, $CDCl_3$):

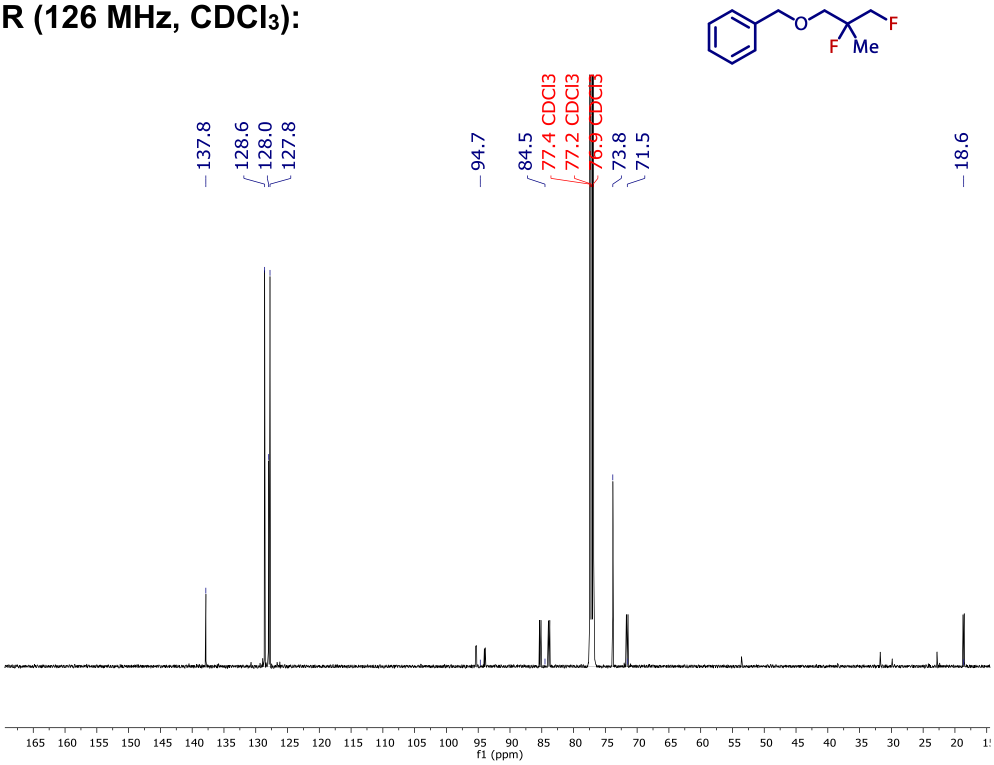

^{19}F NMR (376 MHz, $CDCl_3$):

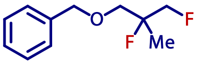

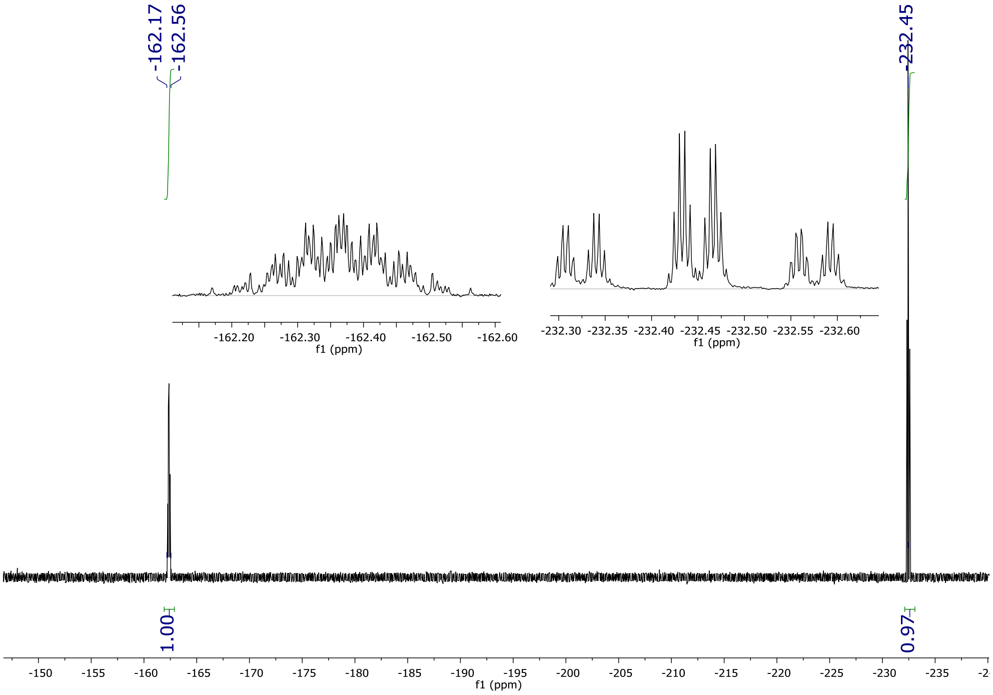

1-((2,3-difluoro-2-methylpropoxy)methyl)-4-(trifluoromethyl)benzene, 3au

^{1}H NMR (500 MHz, $CDCl_3$):

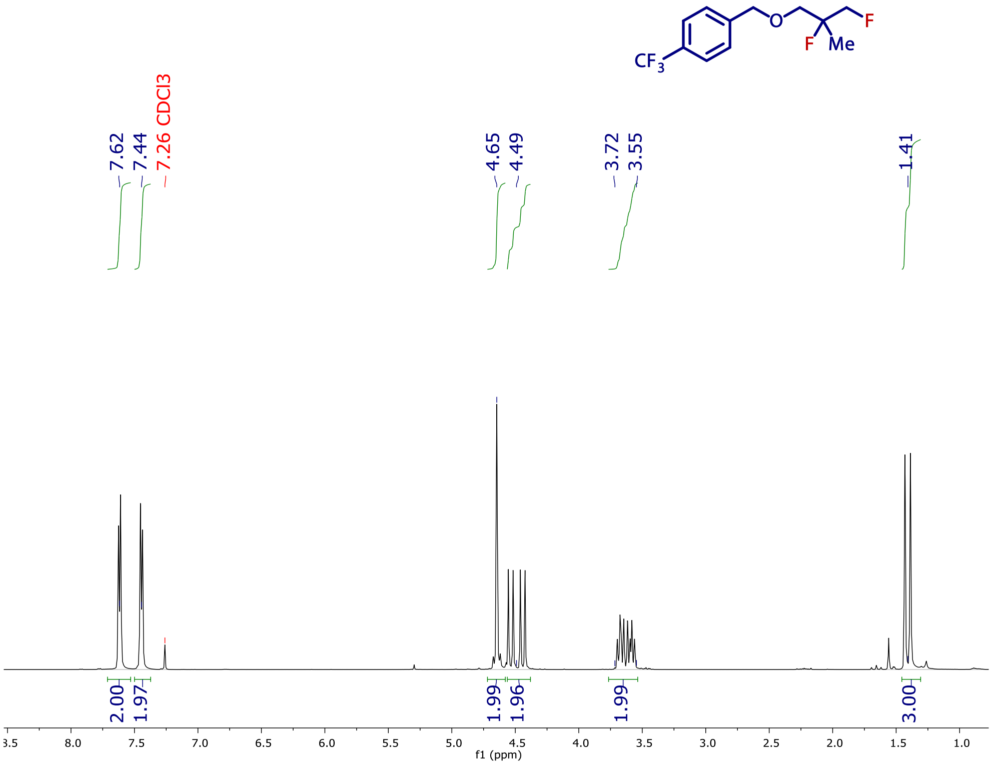

^{13}C NMR (126 MHz, $CDCl_3$):

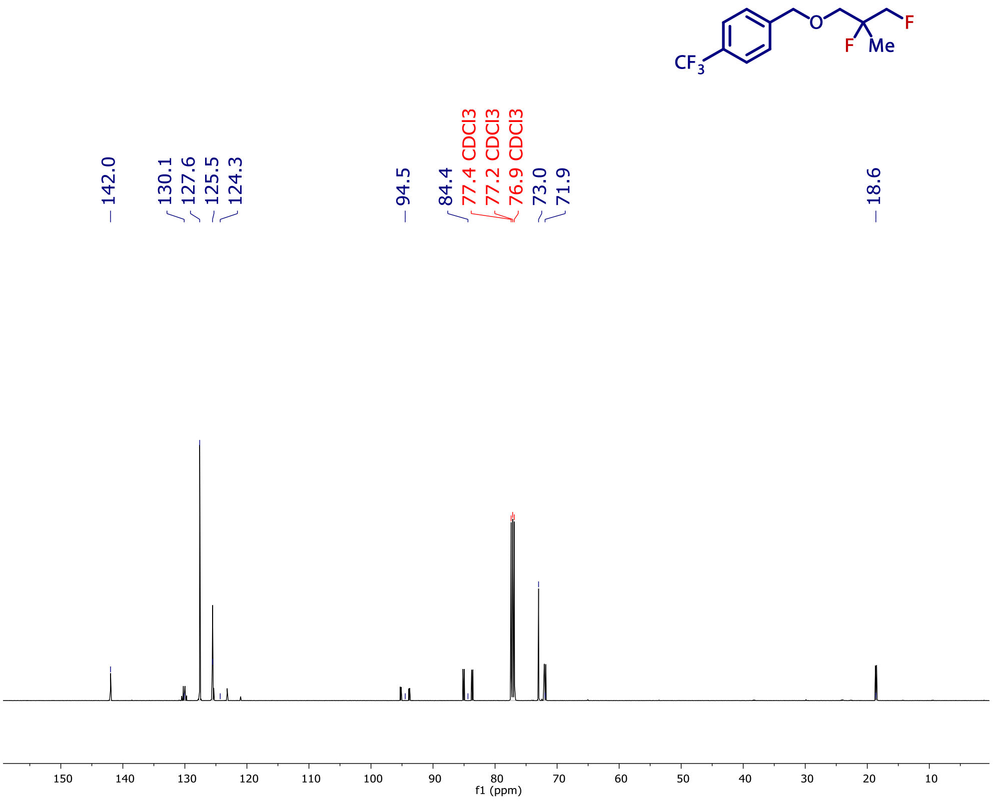

^{19}F NMR (376 MHz, $CDCl_3$):

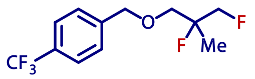

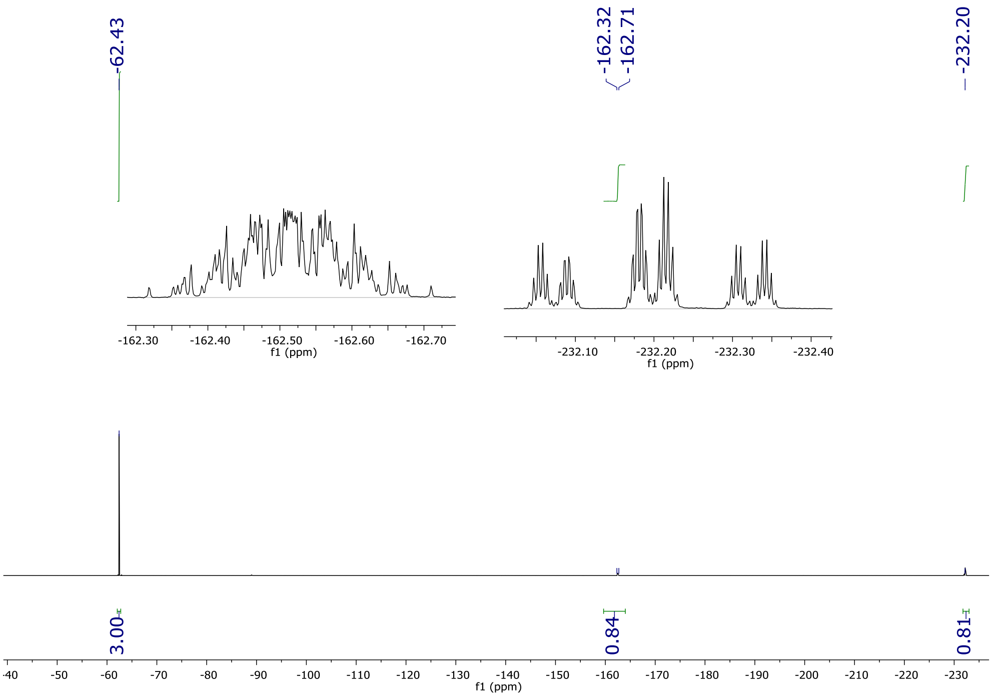

References:

[1] S. Lin, C-X. Song, G-X. Cai, W-H. Wang, Z.-J. Shi, *J. Am. Chem. Soc.,* **2008**, 130, 12901-12903

[2] I. G. Molnár, R. Gilmour, *J. Am. Chem. Soc.,* **2016**, 138, 15, 5004-5007

[3] R. Savelo and R. Leino, *Synthesis*, **2015**, 47, 1749-1760

[4] A. Braun, I. H. Cho, S. Ciblat, D. Clyne, P. Forgione, A. C. Hart, G. Huang, J. Kim, I. Modolo, L. A. Paquette, X. Peng, S. Pichlmair, C. A. Stewart, J. Wang, D. Zuev, *Collect. Czech. Chem. Commun.,* **2009**, 74, 651-769.

[5] P. D. Zeits, G. P. Rachiero, F. Hampel, J. H. Reibenspies, J. A. Gladysz, *Organometallics,* **2012,** 31, 2854-2877

[6] X. Dong, R. Sang, Q. Wang, X- Y. Tang, M. Shi, *Chem. Eur. J.*, **2013**, 19, 16910-16915

[7] J. M. Larsson, T. S. N. Zhao, K. J. Szabo, *Org. Lett.,* **2011**, 13, 1888-1891

[8] Y. He, Z. Yang, R. T. Thonbury, D. F. Toste, *J. Am. Chem. Soc.*, **2015**, 137, 12207-12210

[9] S. M. Banik, J. W. Medley, E. N. Jacobsen, *J. Am. Chem. Soc*., **2016,** 138, 5000-5003

[10] C. Tabélé, C. Curti, Y. Kabri, N. Primas, P. Vanelle, *Molecules,* **2015**, 20, 22890–22899

[11] H. J. L. Jiwan, P. L. Soumilion, *J. Phys. Chem*., **1995**, 99, 14223-14230

[12] A. J. Cresswell, S. G. Davies, J. A. Lee, M. J. Morris, P. M. Roberts, J. E. Thomson, *J. Org. Chem.,* **2012**, 77, 7262 – 7281

[13] D. C. Blakemore, A. Gilbert, *J. Chem. Soc., Perkin Trans*., **1992**, 1, 2265-2270

[14] X. Ma, H. Dang, J. A. Rose, P. Rablen, S. B. Herzon *J. Am. Chem. Soc.,* **2017**, 139, 16, 5998-6007

[15] W-C. Gao, Z-Y. Xiong, S. Pirhaghani, T. Wirth, *Synthesis,* **2019**, 51, 276–284

[16] C. Gütz, B. Klöckner, S. R. Waldvogel, *Org. Process. Res. Dev.,* **2016**, 20, 26 - 32

[17] T. Kitamura, K. Muta, J. Oyamada. *J. Org. Chem.,* **2015**, 80, 10431 - 10436

[18] F. Scheidt, M. Schäfer, J. C. Sarie, C. G. Daniliuc, J. J. Molloy, R. Gilmour, *Angew. Chem. Int. Ed.,* **2018**, 57, 16431-16435

[19] G. S. Lal, *J. Org. Chem.,* **1993**, 58, 2791 – 2796

[20] As of 23th August 2019; https://www.britishgas.co.uk/products-and-services/gas-and-electricity/our-energy-tariffs/tariff-information.html

[21] As of 23th August 2019
